# Supplementary material for: Innovative monitoring scheme adapted to remote, scattered nesting aggregation reveals a major loggerhead turtle rookery in New Caledonia, South Pacific
Source: PLoS One. 2024 Jun 18;19(6):e0299748. doi: 10.1371/journal.pone.0299748 (PMC11185463; doi:10.1371/journal.pone.0299748)
Supplement: S1 File — (PDF) [file pone.0299748.s001.pdf]

season	date_interval	islet	activities	successful_ne	mission	survey_modal
2017_2018	13/11/2017-0	atire	10	7	1	1
2017_2018	13/11/2017-0	gi	7	5	1	1
2017_2018	13/11/2017-0	ieroue	0	0	1	1
2017_2018	14/11/2017-0	kouare	4	3	1	1
2017_2018	14/11/2017-0	nda	6	5	1	1
2017_2018	13/11/2017-0	nge	8	4	1	1
2017_2018	14/11/2017-0	redika	6	2	1	1
2017_2018	14/11/2017-0	tere	2	1	1	1
2017_2018	13/11/2017-0	ua	0	0	1	1
2017_2018	13/11/2017-0	uaterembi	5	3	1	1
2017_2018	13/11/2017-0	vua	3	2	1	1
2017_2018	05/12/2017-2	atire	15	10	2	1
2017_2018	05/12/2017-2	gi	13	6	2	1
2017_2018	05/12/2017-2	ieroue	2	2	2	1
2017_2018	06/12/2017-2	kouare	9	4	2	1
2017_2018	06/12/2017-2	nda	17	5	2	1
2017_2018	05/12/2017-2	nge	6	3	2	1
2017_2018	06/12/2017-2	redika	3	3	2	1
2017_2018	06/12/2017-2	tere	0	0	2	1
2017_2018	05/12/2017-2	ua	8	3	2	1
2017_2018	05/12/2017-2	uaterembi	9	1	2	1
2017_2018	05/12/2017-2	vua	2	2	2	1
2017_2018	23/12/2017-0	gi	9	7	3	1
2017_2018	23/12/2017-0	nda	4	4	3	1
2017_2018	22/12/2017-0	nge	4	2	3	1
2017_2018	23/12/2017-0	tere	0	0	3	1
2017_2018	23/12/2017-0	ua	0	0	3	1
2017_2018	22/12/2017-0	uaterembi	15	10	3	1
2017_2018	22/12/2017-0	atire	21	12	3	1
2017_2018	22/12/2017-0	ieroue	2	2	3	1
2017_2018	23/12/2017-0	kouare	0	0	3	1
2017_2018	22/12/2017-0	redika	21	14	3	1
2017_2018	22/12/2017-0	vua	8	6	3	1
2017_2018	02/02/2018-2	atire	2	2	5	1
2017_2018	01/02/2018-2	gi	6	5	5	1
2017_2018	02/02/2018-2	ieroue	2	2	5	1
2017_2018	01/02/2018-2	kouare	0	0	5	1
2017_2018	01/02/2018-2	nda	4	2	5	1
2017_2018	01/02/2018-2	nge	0	0	5	1
2017_2018	02/02/2018-2	redika	8	4	5	1
2017_2018	01/02/2018-2	tere	1	1	5	1
2017_2018	01/02/2018-2	ua	0	0	5	1
2017_2018	02/02/2018-2	uaterembi	4	3	5	1
2017_2018	02/02/2018-2	vua	2	2	5	1
2017_2018	24/02/2018-1	atire	1	1	6	1
2017_2018	23/02/2018-1	gi	0	0	6	1
2017_2018	24/02/2018-1	ieroue	0	0	6	1
2017_2018	23/02/2018-1	kouare	0	0	6	1
2017_2018	23/02/2018-1	nda	2	2	6	1

2017_2018	23/02/2018-1 nge	1	1	6	1
2017_2018	24/02/2018-1 redika	2	2	6	1
2017_2018	23/02/2018-1 tere	0	0	6	1
2017_2018	23/02/2018-1 ua	0	0	6	1
2017_2018	24/02/2018-1 uaterembi	1	1	6	1
2017_2018	24/02/2018-1 vua	0	0	6	1
2018_2019	31/10/2018-2 atire	2	1	1	1
2018_2019	22/11/2018-2 atire	4	3	2	1
2018_2019	30/11/2018-2 atire	16	12	3	1
2018_2019	22/12/2018-0 atire	21	10	4	1
2018_2019	01/11/2018-2 gi	1	1	1	1
2018_2019	23/11/2018-2 gi	7	4	2	1
2018_2019	30/11/2018-2 gi	14	9	3	1
2018_2019	22/12/2018-1 gi	18	11	4	1
2018_2019	01/11/2018-2 ieroue	2	1	1	1
2018_2019	23/11/2018-2 ieroue	0	0	2	1
2018_2019	29/11/2018-2 ieroue	3	1	3	1
2018_2019	21/12/2018-1 ieroue	0	0	4	1
2018_2019	31/10/2018-2 kouare	0	0	1	1
2018_2019	22/11/2018-2 kouare	3	1	2	1
2018_2019	30/11/2018-2 kouare	18	7	3	1
2018_2019	22/12/2018-0 kouare	7	3	4	1
2018_2019	31/10/2018-2 nda	2	1	1	1
2018_2019	22/11/2018-2 nda	1	0	2	1
2018_2019	30/11/2018-2 nda	1	1	3	1
2018_2019	21/12/2018-0 nda	6	2	4	1
2018_2019	31/10/2018-2 nge	12	3	1	1
2018_2019	22/11/2018-2 nge	13	5	2	1
2018_2019	30/11/2018-2 nge	12	5	3	1
2018_2019	21/12/2018-0 nge	24	14	4	1
2018_2019	31/10/2018-2 redika	0	0	1	1
2018_2019	22/11/2018-2 redika	1	1	2	1
2018_2019	29/11/2018-2 redika	5	3	3	1
2018_2019	21/12/2018-1 redika	11	5	4	1
2018_2019	31/10/2018-2 ua	0	0	1	1
2018_2019	22/11/2018-2 ua	0	0	2	1
2018_2019	29/11/2018-2 ua	1	1	3	1
2018_2019	31/10/2018-2 uaterembi	0	0	1	1
2018_2019	22/11/2018-2 uaterembi	3	1	2	1
2018_2019	29/11/2018-2 uaterembi	4	2	3	1
2018_2019	21/12/2018-0 uaterembi	12	5	4	1
2018_2019	31/10/2018-2 uatio	1	1	1	1
2018_2019	22/11/2018-2 uatio	4	3	2	1
2018_2019	29/11/2018-2 uatio	8	2	3	1
2018_2019	21/12/2018-1 uatio	11	6	4	1
2018_2019	31/10/2018-2 vua	0	0	1	1
2018_2019	22/11/2018-2 vua	2	1	2	1
2018_2019	29/11/2018-2 vua	8	4	3	1
2018_2019	21/12/2018-0 vua	11	9	4	1
2018_2019	02/01/2019-2 amere	14	10	6	0

2018_2019	02/01/2019-2 du ami	2	1	6	0
2018_2019	02/01/2019-2 kie	20	19	6	0
2018_2019	03/01/2019-2 koko	10	7	6	0
2018_2019	04/01/2019-2 mato	1	1	6	0
2018_2019	03/01/2019-2 ndo	1	1	6	0
2018_2019	04/01/2019-2 noe	1	0	6	0
2018_2019	03/01/2019-2 petit koko	4	3	6	0
2018_2019	03/01/2019-2 totea	4	3	6	0
2018_2019	03/01/2019-2 uie	2	1	6	0
2018_2019	04/01/2019-2 uo	2	2	6	0
2019_2020	02/01/2020-2 mato	10	2	8	0
2019_2020	06/11/2019-2 atire	1	1	1	1
2019_2020	28/11/2019-0 atire	9	2	2	1
2019_2020	06/11/2019-2 nda	0	0	1	0
2019_2020	28/11/2019-0 nda	6	2	2	0
2019_2020	01/01/2020-2 ndo	16	11	8	0
2019_2020	05/12/2019-1 atire	15	4	3	1
2019_2020	11/12/2019-2 atire	19	6	4	1
2019_2020	07/01/2020-2 atire	7	5	9	1
2019_2020	07/11/2019-2 gi	12	5	1	1
2019_2020	29/11/2019-0 gi	2	1	2	1
2019_2020	12/12/2019-0 gi	8	5	5	1
2019_2020	03/01/2020-0 gi	1	0	6	1
2019_2020	10/01/2020-1 gi	3	2	7	1
2019_2020	07/11/2019-2 ieroue	0	0	1	1
2019_2020	29/11/2019-0 ieroue	3	1	2	1
2019_2020	19/12/2019-0 ieroue	9	1	6	1
2019_2020	10/01/2020-1 ieroue	0	0	7	1
2019_2020	18/01/2020-2 ieroue	1	1	9	1
2019_2020	06/11/2019-2 kouare	0	0	1	1
2019_2020	28/11/2019-0 kouare	4	0	2	1
2019_2020	13/12/2019-0 kouare	3	2	5	1
2019_2020	04/01/2020-1 kouare	2	2	7	1
2019_2020	17/01/2020-2 kouare	1	0	9	1
2019_2020	07/11/2019-2 nge	2	0	1	1
2019_2020	29/11/2019-0 nge	12	6	2	1
2019_2020	12/12/2019-0 nge	10	4	5	1
2019_2020	03/01/2020-1 nge	4	2	7	1
2019_2020	17/01/2020-2 nge	6	5	9	1
2019_2020	13/12/2019-0 noe	0	0	5	1
2019_2020	04/01/2020-1 noe	3	2	7	1
2019_2020	18/01/2020-2 noe	4	1	9	1
2019_2020	13/12/2019-0 puemba	4	4	5	1
2019_2020	04/01/2020-1 puemba	3	0	7	1
2019_2020	18/01/2020-2 puemba	2	2	9	1
2019_2020	07/11/2019-2 redika	2	0	1	1
2019_2020	29/11/2019-0 redika	5	0	2	1
2019_2020	12/12/2019-0 redika	40	6	5	1
2019_2020	03/01/2020-1 redika	7	6	7	1
2019_2020	17/01/2020-2 redika	8	2	9	1

2019_2020	06/11/2019-2 uaterembi	13	1	1	1
2019_2020	28/11/2019-0 uaterembi	17	1	2	1
2019_2020	12/12/2019-0 uaterembi	16	1	5	1
2019_2020	07/01/2020-2 uaterembi	8	2	9	1
2019_2020	07/11/2019-2 uatio	6	1	1	1
2019_2020	29/11/2019-0 uatio	5	1	2	1
2019_2020	12/12/2019-0 uatio	18	4	5	1
2019_2020	03/01/2020-1 uatio	1	1	7	1
2019_2020	17/01/2020-2 uatio	8	5	9	1
2019_2020	13/12/2019-0 uie	13	5	5	1
2019_2020	04/01/2020-1 uie	2	2	7	1
2019_2020	18/01/2020-2 uie	3	1	9	1
2019_2020	13/12/2019-0 uo	5	1	5	1
2019_2020	04/01/2020-1 uo	1	1	7	1
2019_2020	18/01/2020-2 uo	0	0	9	1
2019_2020	01/01/2020-2 nouare	0	0	8	0
2019_2020	02/01/2020-2 pumbo	2	0	8	0
2019_2020	06/11/2019-2 tere	0	0	1	0
2019_2020	28/11/2019-0 tere	0	0	2	0
2019_2020	01/01/2020-2 ua	7	5	8	0
2019_2020	23/01/2020-2 ua	4	3	9	0
2019_2020	01/01/2020-2 ugo	0	0	8	0
2019_2020	14/11/2019-0 vua	10	3	2	0
2020_2021	02/12/2020-2 mato	2	0	3	0
2020_2021	24/12/2020-0 mato	5	1	6	0
2020_2021	01/12/2020-2 mbore	2	2	3	0
2020_2021	23/12/2020-0 mbore	0	0	6	0
2020_2021	01/12/2020-2 ndo	2	1	3	0
2020_2021	23/12/2020-0 ndo	5	2	6	0
2020_2021	07/11/2020-2 atire	2	1	1	1
2020_2021	29/11/2020-0 atire	10	6	2	1
2020_2021	10/12/2020-2 atire	11	9	4	1
2020_2021	24/12/2020-0 atire	15	6	5	1
2020_2021	06/01/2021-2 atire	17	12	7	1
2020_2021	21/01/2021-0 atire	10	7	8	1
2020_2021	08/11/2020-2 gi	3	3	1	1
2020_2021	30/11/2020-0 gi	7	1	2	1
2020_2021	09/12/2020-2 gi	7	4	4	1
2020_2021	23/12/2020-0 gi	11	4	5	1
2020_2021	16/01/2021-0 gi	1	1	8	1
2020_2021	07/11/2020-2 ieroue	1	1	1	1
2020_2021	29/11/2020-0 ieroue	0	0	2	1
2020_2021	09/12/2020-2 ieroue	2	1	4	1
2020_2021	24/12/2020-0 ieroue	3	2	5	1
2020_2021	06/01/2021-1 ieroue	1	1	7	1
2020_2021	20/01/2021-0 ieroue	1	0	8	1
2020_2021	07/11/2020-2 kouare	5	2	1	1
2020_2021	29/11/2020-0 kouare	12	4	2	1
2020_2021	10/12/2020-2 kouare	5	3	4	1
2020_2021	24/12/2020-0 kouare	9	6	5	1

2020_2021	05/01/2021-2 kouare	8	7	7	1
2020_2021	21/01/2021-0 kouare	2	2	8	1
2020_2021	07/11/2020-2 nda	10	2	1	1
2020_2021	29/11/2020-0 nda	3	1	2	1
2020_2021	10/12/2020-2 nda	1	0	4	1
2020_2021	24/12/2020-0 nda	6	2	5	1
2020_2021	05/01/2021-2 nda	5	4	7	1
2020_2021	21/01/2021-0 nda	3	1	8	1
2020_2021	07/11/2020-2 nge	6	1	1	1
2020_2021	29/11/2020-0 nge	15	5	2	1
2020_2021	09/12/2020-2 nge	16	5	4	1
2020_2021	24/12/2020-0 nge	6	1	5	1
2020_2021	06/01/2021-1 nge	9	5	7	1
2020_2021	20/01/2021-0 nge	2	0	8	1
2020_2021	08/11/2020-2 redika	3	1	1	1
2020_2021	30/11/2020-0 redika	1	0	2	1
2020_2021	09/12/2020-2 redika	0	0	4	1
2020_2021	23/12/2020-0 redika	4	0	5	1
2020_2021	06/01/2021-1 redika	1	0	7	1
2020_2021	20/01/2021-0 redika	0	0	8	1
2020_2021	07/11/2020-2 tere	0	0	1	1
2020_2021	29/11/2020-0 tere	0	0	2	1
2020_2021	10/12/2020-2 tere	0	0	4	1
2020_2021	24/12/2020-0 tere	0	0	5	1
2020_2021	05/01/2021-2 tere	0	0	7	1
2020_2021	21/01/2021-0 tere	0	0	8	1
2020_2021	08/11/2020-2 ua	1	1	1	1
2020_2021	30/11/2020-0 ua	0	0	2	1
2020_2021	09/12/2020-2 ua	0	0	4	1
2020_2021	23/12/2020-0 ua	0	0	5	1
2020_2021	05/01/2021-1 ua	0	0	7	1
2020_2021	20/01/2021-0 ua	0	0	8	1
2020_2021	08/11/2020-2 uaterembi	0	0	1	1
2020_2021	30/11/2020-0 uaterembi	7	1	2	1
2020_2021	09/12/2020-2 uaterembi	11	2	4	1
2020_2021	23/12/2020-0 uaterembi	9	3	5	1
2020_2021	05/01/2021-1 uaterembi	9	4	7	1
2020_2021	20/01/2021-0 uaterembi	4	2	8	1
2020_2021	08/11/2020-2 uatio	5	2	1	1
2020_2021	30/11/2020-0 uatio	3	0	2	1
2020_2021	09/12/2020-2 uatio	3	0	4	1
2020_2021	23/12/2020-0 uatio	8	1	5	1
2020_2021	05/01/2021-1 uatio	4	0	7	1
2020_2021	20/01/2021-0 uatio	0	0	8	1
2020_2021	07/11/2020-2 vua	3	1	1	1
2020_2021	29/11/2020-0 vua	3	1	2	1
2020_2021	02/12/2020-2 noe	0	0	3	0
2020_2021	24/12/2020-0 noe	0	0	6	0
2020_2021	02/12/2020-2 nouare	2	0	3	0
2020_2021	01/12/2020-2 petit koko	0	0	3	0

2020_2021	23/12/2020-0 petit koko	0	0	6	0
2020_2021	02/12/2020-2 puemba	1	1	3	0
2020_2021	24/12/2020-0 puemba	2	2	6	0
2020_2021	02/12/2020-2 pumbo	1	0	3	0
2020_2021	24/12/2020-0 pumbo	2	1	6	0
2020_2021	10/12/2020-2 vua	8	5	4	1
2020_2021	23/12/2020-0 vua	13	6	5	1
2020_2021	06/01/2021-1 vua	14	6	7	1
2020_2021	21/01/2021-0 vua	2	1	8	1
2020_2021	01/12/2020-2 totea	2	1	3	0
2020_2021	23/12/2020-0 totea	3	2	6	0
2020_2021	02/12/2020-2 ugo	0	0	3	0
2020_2021	24/12/2020-0 ugo	0	0	6	0
2020_2021	01/12/2020-2 uie	2	1	3	0
2020_2021	23/12/2020-0 uie	4	2	6	0
2020_2021	01/12/2020-2 uo	0	0	3	0
2020_2021	23/12/2020-0 uo	0	0	6	0
2021_2022	25/11/2021-1 mato	4	2	3	0
2021_2022	17/12/2021-2 mato	4	1	5	0
2021_2022	26/11/2021-1 mbore	1	1	3	0
2021_2022	18/12/2021-3 mbore	0	0	5	0
2021_2022	26/11/2021-1 ndo	2	2	3	0
2021_2022	18/12/2021-3 ndo	4	1	5	0
2021_2022	25/11/2021-1 noe	0	0	3	0
2021_2022	17/12/2021-2 noe	2	2	5	0
2021_2022	25/11/2021-1 nouare	3	1	3	0
2021_2022	17/12/2021-2 nouare	0	0	5	0
2021_2022	26/11/2021-1 petit koko	1	0	3	0
2021_2022	18/12/2021-3 petit koko	1	1	5	0
2021_2022	25/11/2021-1 puemba	1	1	3	0
2021_2022	17/12/2021-2 puemba	2	2	5	0
2021_2022	25/11/2021-1 pumbo	0	0	3	0
2021_2022	17/12/2021-2 pumbo	0	1	5	0
2021_2022	27/10/2021-1 atire	2	1	1	1
2021_2022	28/10/2021-1 gi	6	4	1	1
2021_2022	27/10/2021-1 ieroue	0	0	1	1
2021_2022	27/10/2021-1 kouare	0	0	1	1
2021_2022	27/10/2021-1 nda	0	0	1	1
2021_2022	27/10/2021-1 nge	5	2	1	1
2021_2022	28/10/2021-1 redika	0	0	1	1
2021_2022	27/10/2021-1 tere	0	0	1	1
2021_2022	28/10/2021-1 ua	0	0	1	1
2021_2022	28/10/2021-1 uaterembi	0	0	1	1
2021_2022	28/10/2021-1 uatio	0	0	1	1
2021_2022	27/10/2021-1 vua	3	1	1	1
2021_2022	18/11/2021-0 atire	10	6	2	1
2021_2022	19/11/2021-0 gi	17	7	2	1
2021_2022	18/11/2021-0 ieroue	1	1	2	1
2021_2022	18/11/2021-0 kouare	3	1	2	1
2021_2022	18/11/2021-0 nda	6	3	2	1

2021_2022	18/11/2021-0 nge	19	12	2	1
2021_2022	19/11/2021-0 redika	4	4	2	1
2021_2022	18/11/2021-0 tere	0	0	2	1
2021_2022	19/11/2021-0 ua	0	0	2	1
2021_2022	19/11/2021-0 uaterembi	16	7	2	1
2021_2022	19/11/2021-0 uatio	3	3	2	1
2021_2022	26/11/2021-1 totea	8	5	3	0
2021_2022	18/12/2021-3 totea	4	0	5	0
2021_2022	25/11/2021-1 ugo	0	0	3	0
2021_2022	18/11/2021-0 vua	10	6	2	1
2021_2022	09/12/2021-2 atire	5	3	4	1
2021_2022	08/12/2021-2 gi	16	8	4	1
2021_2022	09/12/2021-2 ieroue	1	1	4	1
2021_2022	08/12/2021-2 kouare	3	2	4	1
2021_2022	08/12/2021-2 nda	3	1	4	1
2021_2022	08/12/2021-2 nge	16	11	4	1
2021_2022	09/12/2021-2 redika	4	3	4	1
2021_2022	08/12/2021-2 tere	0	0	4	1
2021_2022	09/12/2021-2 ua	2	1	4	1
2021_2022	08/12/2021-2 uaterembi	8	5	4	1
2021_2022	17/12/2021-2 ugo	0	0	5	0
2021_2022	26/11/2021-1 uie	11	6	3	0
2021_2022	18/12/2021-2 uie	1	0	5	0
2021_2022	26/11/2021-1 uo	1	1	3	0
2021_2022	18/12/2021-3 uo	3	3	5	0
2021_2022	09/12/2021-2 uatio	7	2	4	1
2021_2022	08/12/2021-2 vua	8	0	4	1
2021_2022	22/12/2021-0 atire	11	7	6	1
2021_2022	23/12/2021-0 gi	14	9	6	1
2021_2022	23/12/2021-0 ieroue	1	1	6	1
2021_2022	22/12/2021-0 kouare	3	2	6	1
2021_2022	22/12/2021-0 nda	4	3	6	1
2021_2022	23/12/2021-0 nge	11	9	6	1
2021_2022	23/12/2021-0 redika	5	3	6	1
2021_2022	22/12/2021-0 tere	0	0	6	1
2021_2022	22/12/2021-0 ua	1	1	6	1
2021_2022	22/12/2021-0 uaterembi	11	5	6	1
2021_2022	23/12/2021-0 uatio	6	2	6	1
2021_2022	22/12/2021-0 vua	9	4	6	1
2021_2022	06/01/2022-2 atire	15	10	7	1
2021_2022	06/01/2022-2 gi	21	13	7	1
2021_2022	06/01/2022-2 ieroue	2	2	7	1
2021_2022	06/01/2022-2 kouare	7	3	7	1
2021_2022	06/01/2022-2 nda	6	4	7	1
2021_2022	06/01/2022-2 nge	10	8	7	1
2021_2022	06/01/2022-2 redika	3	2	7	1
2021_2022	06/01/2022-2 tere	0	0	7	1
2021_2022	06/01/2022-2 ua	0	0	7	1
2021_2022	05/01/2022-2 uaterembi	9	5	7	1
2021_2022	06/01/2022-2 uatio	2	1	7	1

2021_2022	05/01/2022-2 vua	16	9	7	1
2021_2022	28/01/2022-1 atire	8	4	8	1
2021_2022	27/01/2022-1 gi	2	2	8	1
2021_2022	27/01/2022-1 ieroue	0	0	8	1
2021_2022	28/01/2022-1 kouare	0	0	8	1
2021_2022	28/01/2022-1 nda	0	0	8	1
2021_2022	28/01/2022-1 nge	8	6	8	1
2021_2022	28/01/2022-1 redika	0	0	8	1
2021_2022	28/01/2022-1 tere	0	0	8	1
2021_2022	27/01/2022-1 ua	1	1	8	1
2021_2022	27/01/2022-1 uaterembi	0	0	8	1
2021_2022	27/01/2022-1 uatio	0	0	8	1
2021_2022	27/01/2022-1 vua	1	0	8	1
2021_2022	16/02/2022-2 gi	2	2	10	1
2021_2022	16/02/2022-2 nge	0	0	10	1
2021_2022	18/02/2022-1 atire	0	0	11	1
2021_2022	24/02/2022-1 gi	2	2	11	1
2021_2022	17/02/2022-1 ieroue	0	0	11	1
2021_2022	18/02/2022-1 kouare	0	0	11	1
2021_2022	18/02/2022-1 nda	0	0	11	1
2021_2022	24/02/2022-1 nge	1	1	11	1
2021_2022	19/02/2022-1 redika	0	0	11	1
2021_2022	18/02/2022-1 tere	0	0	11	1
2021_2022	17/02/2022-1 ua	0	0	11	1
2021_2022	17/02/2022-1 uaterembi	0	0	11	1
2021_2022	17/02/2022-1 uatio	0	0	11	1
2021_2022	18/02/2022-1 vua	1	1	11	1
2022_2023	05/11/2022-2 atire	4	1	1	1
2022_2023	05/11/2022-2 gi	3	2	1	1
2022_2023	06/11/2022-2 ieroue	4	3	1	1
2022_2023	05/11/2022-2 kouare	1	1	1	1
2022_2023	05/11/2022-2 nda	6	2	1	1
2022_2023	06/11/2022-2 nge	2	2	1	1
2022_2023	05/11/2022-2 redika	0	0	1	1
2022_2023	05/11/2022-2 tere	0	0	1	1
2022_2023	06/11/2022-2 ua	1	1	1	1
2022_2023	06/11/2022-2 uaterembi	3	1	1	1
2022_2023	06/11/2022-2 uatio	8	4	1	1
2022_2023	05/11/2022-2 vua	2	2	1	1
2022_2023	18/11/2022-0 amere	6	5	2	0
2022_2023	18/11/2022-0 kie	7	4	2	0
2022_2023	18/11/2022-0 nouare	0	0	2	0
2022_2023	23/11/2022-0 gi	2	1	3	1
2022_2023	24/11/2022-0 ieroue	8	5	3	1
2022_2023	23/11/2022-0 kouare	9	6	3	1
2022_2023	23/11/2022-0 nda	18	4	3	1
2022_2023	24/11/2022-0 nge	8	4	3	1
2022_2023	24/11/2022-0 ua	9	3	3	1
2022_2023	24/11/2022-0 uatio	8	1	3	1
2022_2023	23/11/2022-0 vua	9	6	3	1

2022_2023	28/11/2022-1 mato	0	0	4	0
2022_2023	27/11/2022-1 mbore	3	2	4	0
2022_2023	27/11/2022-1 ndo	7	4	4	0
2022_2023	28/11/2022-1 noe	0	0	4	0
2022_2023	27/11/2022-1 petit koko	1	1	4	0
2022_2023	28/11/2022-1 puemba	5	5	4	0
2022_2023	23/11/2022-1 tere	0	0	4	1
2022_2023	27/11/2022-1 totea	14	6	4	0
2022_2023	24/11/2022-1 uaterembi	17	5	4	1
2022_2023	28/11/2022-1 ugo	0	0	4	0
2022_2023	27/11/2022-1 uie	0	0	4	0
2022_2023	27/11/2022-1 uo	1	0	4	0
2022_2023	23/11/2022-1 atire	13	9	5	1
2022_2023	09/12/2022-1 ieroue	6	2	5	1
2022_2023	09/12/2022-1 kouare	3	2	5	1
2022_2023	09/12/2022-1 nda	15	6	5	1
2022_2023	09/12/2022-1 vua	11	4	5	1
2022_2023	06/12/2022-2 amere	6	2	6	0
2022_2023	06/12/2022-2 kie	13	10	6	0
2022_2023	06/12/2022-2 nouare	0	0	6	0
2022_2023	16/12/2022-3 mato	1	0	7	0
2022_2023	15/12/2022-2 mbore	2	1	7	0
2022_2023	15/12/2022-2 ndo	9	9	7	0
2022_2023	09/12/2022-2 nge	7	4	7	1
2022_2023	16/12/2022-3 noe	0	0	7	0
2022_2023	15/12/2022-2 petit koko	1	1	7	0
2022_2023	16/12/2022-3 puemba	2	2	7	0
2022_2023	15/12/2022-2 tere	0	0	7	1
2022_2023	15/12/2022-2 totea	11	5	7	0
2022_2023	16/12/2022-3 ugo	0	0	7	0
2022_2023	15/12/2022-2 uie	0	0	7	0
2022_2023	15/12/2022-2 uo	0	0	7	0
2022_2023	09/12/2022-0 gi	8	4	8	1
2022_2023	09/12/2022-0 ua	5	2	8	1
2022_2023	16/12/2022-0 uaterembi	11	5	8	1
2022_2023	09/12/2022-0 uatio	10	6	8	1
2022_2023	20/12/2022-1 atire	15	9	9	1
2022_2023	05/01/2023-1 gi	6	4	9	1
2022_2023	20/12/2022-1 ieroue	3	2	9	1
2022_2023	20/12/2022-1 kouare	8	3	9	1
2022_2023	20/12/2022-1 nda	18	9	9	1
2022_2023	30/12/2022-1 nge	11	5	9	1
2022_2023	23/11/2022-1 redika	7	3	9	1
2022_2023	30/12/2022-1 tere	0	0	9	1
2022_2023	04/01/2023-1 uaterembi	3	2	9	1
2022_2023	05/01/2023-1 uatio	3	2	9	1
2022_2023	20/12/2022-1 vua	11	6	9	1
2022_2023	13/01/2023-2 atire	3	2	10	1
2022_2023	12/01/2023-2 gi	2	2	10	1
2022_2023	12/01/2023-2 ieroue	2	1	10	1

2022_2023	12/01/2023-2 kouare	0	0	10	1
2022_2023	12/01/2023-2 nda	7	3	10	1
2022_2023	12/01/2023-2 nge	1	1	10	1
2022_2023	12/01/2023-2 tere	2	1	10	1
2022_2023	04/01/2023-2 ua	0	0	10	1
2022_2023	13/01/2023-2 uaterembi	1	0	10	1
2022_2023	13/01/2023-2 uatio	3	2	10	1
2022_2023	13/01/2023-2 vua	6	4	10	1
2022_2023	26/01/2023-0 gi	1	1	11	1
2022_2023	26/01/2023-0 ieroue	0	0	11	1
2022_2023	27/01/2023-0 kouare	0	0	11	1
2022_2023	26/01/2023-0 nda	2	2	11	1
2022_2023	26/01/2023-0 tere	1	1	11	1
2022_2023	27/01/2023-0 ua	0	0	11	1
2022_2023	26/01/2023-0 uaterembi	1	0	11	1
2022_2023	27/01/2023-1 nge	1	1	12	1
2022_2023	27/01/2023-1 vua	0	0	12	1
