# Supplementary material for: Innovative monitoring scheme adapted to remote, scattered nesting aggregation reveals a major loggerhead turtle rookery in New Caledonia, South Pacific
Source: PLoS One. 2024 Jun 18;19(6):e0299748. doi: 10.1371/journal.pone.0299748 (PMC11185463; doi:10.1371/journal.pone.0299748)
Supplement: S2 File — (PDF) [file pone.0299748.s002.pdf]

season	date	mission	islet	successful_ne	avorted_nest	round_trip
2017_2018	04/12/2017	1	atire	1	0	0
2017_2018	04/12/2017	1	atire	1	0	0
2017_2018	04/12/2017	1	atire	1	0	0
2017_2018	04/12/2017	1	atire	1	0	0
2017_2018	04/12/2017	1	atire	1	0	0
2017_2018	04/12/2017	1	atire	0	0	1
2017_2018	04/12/2017	1	atire	0	5	0
2017_2018	04/12/2017	1	atire	1	0	0
2017_2018	04/12/2017	1	atire	1	2	0
2017_2018	04/12/2017	1	atire	0	2	0
2017_2018	04/12/2017	1	vua	0	0	1
2017_2018	04/12/2017	1	vua	1	0	0
2017_2018	04/12/2017	1	vua	1	0	0
2017_2018	04/12/2017	1	uaterembi	1	0	0
2017_2018	04/12/2017	1	uaterembi	1	0	0
2017_2018	04/12/2017	1	uaterembi	0	1	0
2017_2018	04/12/2017	1	uaterembi	0	0	1
2017_2018	04/12/2017	1	uaterembi	1	0	0
2017_2018	04/12/2017	1	gi	1	0	0
2017_2018	04/12/2017	1	gi	0	1	0
2017_2018	04/12/2017	1	gi	1	0	0
2017_2018	04/12/2017	1	gi	1	0	0
2017_2018	04/12/2017	1	gi	0	0	1
2017_2018	04/12/2017	1	gi	1	1	0
2017_2018	04/12/2017	1	gi	1	0	0
2017_2018	04/12/2017	1	nge	1	0	0
2017_2018	04/12/2017	1	nge	1	0	0
2017_2018	04/12/2017	1	nge	0	1	0
2017_2018	04/12/2017	1	nge	0	1	0
2017_2018	04/12/2017	1	nge	0	1	0
2017_2018	04/12/2017	1	nge	1	0	0
2017_2018	04/12/2017	1	nge	1	0	0
2017_2018	04/12/2017	1	nge	0	0	1
2017_2018	05/12/2017	1	kouare	1	0	0
2017_2018	05/12/2017	1	kouare	1	0	0
2017_2018	05/12/2017	1	kouare	0	0	1
2017_2018	05/12/2017	1	kouare	1	0	0
2017_2018	05/12/2017	1	nda	1	0	0
2017_2018	05/12/2017	1	nda	1	0	0
2017_2018	05/12/2017	1	nda	0	1	0
2017_2018	05/12/2017	1	nda	1	0	0
2017_2018	05/12/2017	1	nda	1	0	0
2017_2018	05/12/2017	1	nda	1	1	0
2017_2018	05/12/2017	1	tere	1	0	0
2017_2018	05/12/2017	1	tere	0	1	0
2017_2018	05/12/2017	1	redika	0	0	1
2017_2018	05/12/2017	1	redika	0	0	1
2017_2018	05/12/2017	1	redika	0	0	1
2017_2018	05/12/2017	1	redika	0	0	1

2017_2018	05/12/2017	1	redika	1	2	0
2017_2018	05/12/2017	1	redika	1	0	0
2017_2018	21/12/2017	2	atire	1	0	0
2017_2018	21/12/2017	2	atire	1	0	0
2017_2018	21/12/2017	2	atire	0	1	0
2017_2018	21/12/2017	2	atire	0	0	1
2017_2018	21/12/2017	2	atire	1	0	0
2017_2018	21/12/2017	2	atire	1	0	0
2017_2018	21/12/2017	2	atire	1	0	0
2017_2018	21/12/2017	2	atire	1	0	0
2017_2018	21/12/2017	2	atire	1	0	0
2017_2018	21/12/2017	2	atire	1	0	0
2017_2018	21/12/2017	2	atire	0	0	1
2017_2018	21/12/2017	2	atire	0	0	1
2017_2018	21/12/2017	2	atire	0	1	0
2017_2018	21/12/2017	2	atire	1	0	0
2017_2018	21/12/2017	2	atire	1	0	0
2017_2018	21/12/2017	2	redika	1	0	0
2017_2018	21/12/2017	2	redika	1	0	0
2017_2018	21/12/2017	2	redika	1	0	0
2017_2018	21/12/2017	2	vua	1	0	0
2017_2018	21/12/2017	2	vua	1	0	0
2017_2018	21/12/2017	2	ieroue	1	0	0
2017_2018	21/12/2017	2	ieroue	1	0	0
2017_2018	21/12/2017	2	uaterembi	0	0	1
2017_2018	21/12/2017	2	uaterembi	0	0	1
2017_2018	21/12/2017	2	uaterembi	0	1	0
2017_2018	21/12/2017	2	uaterembi	0	1	0
2017_2018	21/12/2017	2	uaterembi	0	0	1
2017_2018	21/12/2017	2	uaterembi	0	1	0
2017_2018	21/12/2017	2	uaterembi	0	3	0
2017_2018	21/12/2017	2	uaterembi	0	1	0
2017_2018	21/12/2017	2	uaterembi	1	0	0
2017_2018	21/12/2017	2	nge	1	0	0
2017_2018	21/12/2017	2	nge	0	1	0
2017_2018	21/12/2017	2	nge	1	1	0
2017_2018	21/12/2017	2	nge	0	1	0
2017_2018	21/12/2017	2	nge	1	1	0
2017_2018	21/12/2017	2	nge	0	2	0
2017_2018	22/12/2017	2	ua	0	1	0
2017_2018	22/12/2017	2	ua	0	1	0
2017_2018	22/12/2017	2	ua	0	1	0
2017_2018	22/12/2017	2	ua	1	0	0
2017_2018	22/12/2017	2	ua	1	0	0
2017_2018	22/12/2017	2	ua	0	1	0
2017_2018	22/12/2017	2	ua	0	2	0
2017_2018	22/12/2017	2	ua	1	0	0
2017_2018	22/12/2017	2	nda	0	1	0
2017_2018	22/12/2017	2	nda	0	0	1
2017_2018	22/12/2017	2	nda	0	0	1

2017_2018	22/12/2017	2	nda	0	0	1
2017_2018	22/12/2017	2	nda	0	2	0
2017_2018	22/12/2017	2	nda	0	0	1
2017_2018	22/12/2017	2	nda	0	3	0
2017_2018	22/12/2017	2	nda	0	0	1
2017_2018	22/12/2017	2	nda	1	0	0
2017_2018	22/12/2017	2	nda	0	2	0
2017_2018	22/12/2017	2	nda	1	0	0
2017_2018	22/12/2017	2	nda	0	2	0
2017_2018	22/12/2017	2	nda	0	2	0
2017_2018	22/12/2017	2	nda	1	0	0
2017_2018	22/12/2017	2	nda	1	0	0
2017_2018	22/12/2017	2	nda	0	1	0
2017_2018	22/12/2017	2	nda	1	0	0
2017_2018	22/12/2017	2	kouare	1	0	0
2017_2018	22/12/2017	2	kouare	1	0	0
2017_2018	22/12/2017	2	kouare	0	0	1
2017_2018	22/12/2017	2	kouare	0	0	1
2017_2018	22/12/2017	2	kouare	0	0	1
2017_2018	22/12/2017	2	kouare	1	0	0
2017_2018	22/12/2017	2	kouare	0	0	1
2017_2018	22/12/2017	2	kouare	0	0	1
2017_2018	22/12/2017	2	kouare	1	1	0
2017_2018	22/12/2017	2	gi	1	0	0
2017_2018	22/12/2017	2	gi	1	0	0
2017_2018	22/12/2017	2	gi	1	1	0
2017_2018	22/12/2017	2	gi	0	1	0
2017_2018	22/12/2017	2	gi	0	1	0
2017_2018	22/12/2017	2	gi	1	0	0
2017_2018	22/12/2017	2	gi	1	0	0
2017_2018	22/12/2017	2	gi	0	1	0
2017_2018	22/12/2017	2	gi	0	1	0
2017_2018	22/12/2017	2	gi	0	1	0
2017_2018	22/12/2017	2	gi	0	1	0
2017_2018	22/12/2017	2	gi	1	0	0
2017_2018	22/12/2017	2	gi	0	1	0
2017_2018	05/01/2018	3	nda	1	0	0
2017_2018	05/01/2018	3	nda	1	0	0
2017_2018	05/01/2018	3	nda	1	0	0
2017_2018	05/01/2018	3	nda	1	0	0
2017_2018	05/01/2018	3	gi	1	0	0
2017_2018	05/01/2018	3	gi	1	0	0
2017_2018	05/01/2018	3	gi	0	1	0
2017_2018	05/01/2018	3	gi	0	2	0
2017_2018	05/01/2018	3	gi	1	0	0
2017_2018	05/01/2018	3	gi	1	1	0
2017_2018	05/01/2018	3	gi	1	0	0
2017_2018	05/01/2018	3	gi	1	0	0
2017_2018	05/01/2018	3	gi	1	0	0
2017_2018	05/01/2018	3	nge	0	1	0

2017_2018	05/01/2018	3 nge	1	0	0
2017_2018	05/01/2018	3 nge	0	1	0
2017_2018	05/01/2018	3 nge	1	0	0
2017_2018	05/01/2018	3 uaterembi	1	0	0
2017_2018	05/01/2018	3 uaterembi	1	0	0
2017_2018	05/01/2018	3 uaterembi	1	2	0
2017_2018	05/01/2018	3 uaterembi	0	1	0
2017_2018	05/01/2018	3 uaterembi	0	1	0
2017_2018	05/01/2018	3 uaterembi	1	2	0
2017_2018	05/01/2018	3 uaterembi	0	1	0
2017_2018	05/01/2018	3 uaterembi	0	2	0
2017_2018	05/01/2018	3 uaterembi	1	0	0
2017_2018	05/01/2018	3 uaterembi	1	1	0
2017_2018	05/01/2018	3 uaterembi	1	1	0
2017_2018	05/01/2018	3 uaterembi	1	3	0
2017_2018	05/01/2018	3 uaterembi	0	1	0
2017_2018	05/01/2018	3 uaterembi	1	3	0
2017_2018	05/01/2018	3 uaterembi	1	1	0
2017_2018	09/01/2018	3 atire	0	2	0
2017_2018	09/01/2018	3 atire	1	1	0
2017_2018	09/01/2018	3 atire	1	0	0
2017_2018	09/01/2018	3 atire	0	0	1
2017_2018	09/01/2018	3 atire	1	1	0
2017_2018	09/01/2018	3 atire	1	1	0
2017_2018	09/01/2018	3 atire	1	3	0
2017_2018	09/01/2018	3 atire	1	0	0
2017_2018	09/01/2018	3 atire	1	3	0
2017_2018	09/01/2018	3 atire	1	0	0
2017_2018	09/01/2018	3 atire	1	0	0
2017_2018	09/01/2018	3 atire	0	1	0
2017_2018	09/01/2018	3 atire	0	3	0
2017_2018	09/01/2018	3 atire	1	0	0
2017_2018	09/01/2018	3 atire	1	1	0
2017_2018	09/01/2018	3 atire	0	1	0
2017_2018	09/01/2018	3 atire	0	1	0
2017_2018	09/01/2018	3 atire	0	2	0
2017_2018	09/01/2018	3 atire	0	2	0
2017_2018	09/01/2018	3 atire	1	0	0
2017_2018	09/01/2018	3 atire	0	1	0
2017_2018	09/01/2018	3 redika	1	0	0
2017_2018	09/01/2018	3 redika	1	0	0
2017_2018	09/01/2018	3 redika	1	0	0
2017_2018	09/01/2018	3 redika	1	0	0
2017_2018	09/01/2018	3 redika	0	2	0
2017_2018	09/01/2018	3 redika	0	2	0
2017_2018	09/01/2018	3 redika	0	1	0
2017_2018	09/01/2018	3 redika	0	1	0
2017_2018	09/01/2018	3 redika	1	0	0
2017_2018	09/01/2018	3 redika	1	1	0
2017_2018	09/01/2018	3 redika	0	0	1

2017_2018	09/01/2018	3	redika	1	2	0
2017_2018	09/01/2018	3	redika	1	0	0
2017_2018	09/01/2018	3	redika	1	0	0
2017_2018	09/01/2018	3	redika	0	0	1
2017_2018	09/01/2018	3	redika	0	3	0
2017_2018	09/01/2018	3	redika	1	4	0
2017_2018	09/01/2018	3	redika	1	5	0
2017_2018	09/01/2018	3	redika	1	0	0
2017_2018	09/01/2018	3	redika	1	3	0
2017_2018	09/01/2018	3	redika	1	0	0
2017_2018	09/01/2018	3	vua	0	1	0
2017_2018	09/01/2018	3	vua	1	1	0
2017_2018	09/01/2018	3	vua	1	0	0
2017_2018	09/01/2018	3	vua	1	1	0
2017_2018	09/01/2018	3	vua	1	0	0
2017_2018	09/01/2018	3	vua	1	0	0
2017_2018	09/01/2018	3	vua	1	0	0
2017_2018	09/01/2018	3	vua	0	1	0
2017_2018	09/01/2018	3	ieroue	1	0	0
2017_2018	09/01/2018	3	ieroue	1	0	0
2017_2018	22/02/2018	4	nda	0	1	0
2017_2018	22/02/2018	4	nda	1	0	0
2017_2018	22/02/2018	4	nda	1	1	0
2017_2018	22/02/2018	4	nda	0	2	0
2017_2018	22/02/2018	4	tere	1	0	0
2017_2018	22/02/2018	4	gi	1	1	0
2017_2018	22/02/2018	4	gi	1	0	0
2017_2018	22/02/2018	4	gi	1	0	0
2017_2018	22/02/2018	4	gi	1	0	0
2017_2018	22/02/2018	4	gi	1	1	0
2017_2018	22/02/2018	4	gi	0	1	0
2017_2018	23/02/2018	4	uaterembi	1	1	0
2017_2018	23/02/2018	4	uaterembi	1	3	0
2017_2018	23/02/2018	4	uaterembi	0	1	0
2017_2018	23/02/2018	4	uaterembi	1	1	0
2017_2018	23/02/2018	4	ieroue	1	0	0
2017_2018	23/02/2018	4	ieroue	1	0	0
2017_2018	23/02/2018	4	vua	1	0	0
2017_2018	23/02/2018	4	vua	1	0	0
2017_2018	23/02/2018	4	redika	0	0	1
2017_2018	23/02/2018	4	redika	0	0	1
2017_2018	23/02/2018	4	redika	0	3	0
2017_2018	23/02/2018	4	redika	1	0	0
2017_2018	23/02/2018	4	redika	1	0	0
2017_2018	23/02/2018	4	redika	1	0	0
2017_2018	23/02/2018	4	redika	1	0	0
2017_2018	23/02/2018	4	redika	0	2	0
2017_2018	23/02/2018	4	atire	1	0	0
2017_2018	23/02/2018	4	atire	1	0	0
2017_2018	13/03/2018	5	nda	1	0	0

2017_2018	13/03/2018	5	nda	1	0	0
2017_2018	13/03/2018	5	nge	1	0	0
2017_2018	13/03/2018	5	uaterembi	1	0	0
2017_2018	14/03/2018	5	redika	1	0	0
2017_2018	14/03/2018	5	redika	1	0	0
2017_2018	14/03/2018	5	atire	1	0	0
2018_2019	21/11/2018	1	atire	1	1	0
2018_2019	21/11/2018	1	atire	0	0	1
2018_2019	21/11/2018	1	uatio	1	0	0
2018_2019	21/11/2018	1	nge	1	1	0
2018_2019	21/11/2018	1	nge	1	1	0
2018_2019	21/11/2018	1	nge	0	0	1
2018_2019	21/11/2018	1	nge	0	1	0
2018_2019	21/11/2018	1	nge	1	0	0
2018_2019	21/11/2018	1	nge	0	1	0
2018_2019	21/11/2018	1	nge	0	1	0
2018_2019	21/11/2018	1	nge	0	1	0
2018_2019	21/11/2018	1	nge	0	1	0
2018_2019	21/11/2018	1	nge	0	2	0
2018_2019	21/11/2018	1	nge	0	3	0
2018_2019	21/11/2018	1	nge	0	1	0
2018_2019	21/11/2018	1	nda	0	1	0
2018_2019	21/11/2018	1	nda	1	0	0
2018_2019	22/11/2018	1	gi	1	1	0
2018_2019	22/11/2018	1	ieroue	0	1	0
2018_2019	22/11/2018	1	ieroue	1	0	0
2018_2019	28/11/2018	2	redika	1	0	0
2018_2019	28/11/2018	2	vua	1	0	0
2018_2019	28/11/2018	2	vua	0	0	1
2018_2019	28/11/2018	2	uaterembi	1	0	0
2018_2019	28/11/2018	2	uaterembi	0	1	0
2018_2019	28/11/2018	2	uaterembi	0	1	0
2018_2019	28/11/2018	2	uatio	1	2	0
2018_2019	28/11/2018	2	uatio	0	1	0
2018_2019	28/11/2018	2	uatio	1	0	0
2018_2019	28/11/2018	2	uatio	1	1	0
2018_2019	28/11/2018	2	kouare	0	0	1
2018_2019	28/11/2018	2	kouare	0	1	0
2018_2019	28/11/2018	2	kouare	1	0	0
2018_2019	28/11/2018	2	gi	1	1	0
2018_2019	28/11/2018	2	gi	0	1	0
2018_2019	28/11/2018	2	gi	1	0	0
2018_2019	28/11/2018	2	gi	1	2	0
2018_2019	28/11/2018	2	gi	1	0	0
2018_2019	28/11/2018	2	gi	0	3	0
2018_2019	28/11/2018	2	gi	0	1	0
2018_2019	29/11/2018	2	nda	0	0	1
2018_2019	29/11/2018	2	nge	0	3	0
2018_2019	29/11/2018	2	nge	1	2	0
2018_2019	29/11/2018	2	nge	0	1	0

2018_2019	29/11/2018	2 nge	0	1	0
2018_2019	29/11/2018	2 nge	0	1	0
2018_2019	29/11/2018	2 nge	1	1	0
2018_2019	29/11/2018	2 nge	1	0	0
2018_2019	29/11/2018	2 nge	1	2	0
2018_2019	29/11/2018	2 nge	1	0	0
2018_2019	29/11/2018	2 nge	0	1	0
2018_2019	29/11/2018	2 nge	0	1	0
2018_2019	29/11/2018	2 nge	0	0	1
2018_2019	29/11/2018	2 nge	0	1	0
2018_2019	29/11/2018	2 atire	1	0	0
2018_2019	29/11/2018	2 atire	0	3	0
2018_2019	29/11/2018	2 atire	1	0	0
2018_2019	29/11/2018	2 atire	1	0	0
2018_2019	20/12/2018	3 redika	1	0	0
2018_2019	20/12/2018	3 redika	0	6	0
2018_2019	20/12/2018	3 redika	0	0	1
2018_2019	20/12/2018	3 redika	1	0	0
2018_2019	20/12/2018	3 redika	1	0	0
2018_2019	20/12/2018	3 vua	0	0	1
2018_2019	20/12/2018	3 vua	0	0	1
2018_2019	20/12/2018	3 vua	0	1	0
2018_2019	20/12/2018	3 vua	1	0	0
2018_2019	20/12/2018	3 vua	1	0	0
2018_2019	20/12/2018	3 vua	1	0	0
2018_2019	20/12/2018	3 vua	0	0	1
2018_2019	20/12/2018	3 vua	1	0	0
2018_2019	20/12/2018	3 ieroue	0	0	1
2018_2019	20/12/2018	3 ieroue	0	0	1
2018_2019	20/12/2018	3 ieroue	1	0	0
2018_2019	20/12/2018	3 uaterembi	1	0	0
2018_2019	20/12/2018	3 uaterembi	0	0	1
2018_2019	20/12/2018	3 uaterembi	1	1	0
2018_2019	20/12/2018	3 uaterembi	0	0	1
2018_2019	20/12/2018	3 uatio	0	1	0
2018_2019	20/12/2018	3 uatio	0	0	1
2018_2019	20/12/2018	3 uatio	1	0	0
2018_2019	20/12/2018	3 uatio	0	1	0
2018_2019	20/12/2018	3 uatio	0	1	0
2018_2019	20/12/2018	3 uatio	0	0	1
2018_2019	20/12/2018	3 uatio	0	1	0
2018_2019	20/12/2018	3 uatio	1	0	0
2018_2019	20/12/2018	3 ua	1	0	0
2018_2019	20/12/2018	3 nge	0	2	0
2018_2019	20/12/2018	3 nge	0	1	0
2018_2019	20/12/2018	3 nge	1	0	0
2018_2019	20/12/2018	3 nge	0	1	0
2018_2019	20/12/2018	3 nge	1	0	0
2018_2019	20/12/2018	3 nge	0	0	1
2018_2019	20/12/2018	3 nge	0	0	1

2018_2019	20/12/2018	3	nge	1	0	0
2018_2019	20/12/2018	3	nge	1	0	0
2018_2019	20/12/2018	3	nge	1	1	0
2018_2019	20/12/2018	3	nge	0	0	1
2018_2019	20/12/2018	3	nge	0	3	0
2018_2019	20/12/2018	3	nda	1	0	0
2018_2019	21/12/2018	3	gi	0	1	0
2018_2019	21/12/2018	3	gi	1	1	0
2018_2019	21/12/2018	3	gi	1	3	0
2018_2019	21/12/2018	3	gi	1	1	0
2018_2019	21/12/2018	3	gi	1	0	0
2018_2019	21/12/2018	3	gi	1	0	0
2018_2019	21/12/2018	3	gi	1	0	0
2018_2019	21/12/2018	3	gi	1	0	0
2018_2019	21/12/2018	3	gi	0	0	1
2018_2019	21/12/2018	3	gi	1	0	0
2018_2019	21/12/2018	3	gi	1	0	0
2018_2019	21/12/2018	3	gi	0	0	1
2018_2019	21/12/2018	3	gi	0	3	0
2018_2019	21/12/2018	3	gi	0	0	1
2018_2019	21/12/2018	3	kouare	1	0	0
2018_2019	21/12/2018	3	kouare	0	0	1
2018_2019	21/12/2018	3	kouare	0	0	1
2018_2019	21/12/2018	3	kouare	0	0	1
2018_2019	21/12/2018	3	kouare	1	0	0
2018_2019	21/12/2018	3	kouare	1	0	0
2018_2019	21/12/2018	3	kouare	0	0	1
2018_2019	21/12/2018	3	kouare	0	0	1
2018_2019	21/12/2018	3	kouare	0	0	1
2018_2019	21/12/2018	3	kouare	1	0	0
2018_2019	21/12/2018	3	kouare	0	1	0
2018_2019	21/12/2018	3	kouare	0	0	1
2018_2019	21/12/2018	3	kouare	1	0	0
2018_2019	21/12/2018	3	kouare	1	0	0
2018_2019	21/12/2018	3	kouare	0	0	1
2018_2019	21/12/2018	3	kouare	1	0	0
2018_2019	21/12/2018	3	kouare	0	0	1
2018_2019	21/12/2018	3	kouare	0	1	0
2018_2019	21/12/2018	3	atire	1	0	0
2018_2019	21/12/2018	3	atire	1	2	0
2018_2019	21/12/2018	3	atire	1	0	0
2018_2019	21/12/2018	3	atire	1	0	0
2018_2019	21/12/2018	3	atire	0	0	1
2018_2019	21/12/2018	3	atire	0	0	1
2018_2019	21/12/2018	3	atire	1	0	0
2018_2019	21/12/2018	3	atire	1	0	0
2018_2019	21/12/2018	3	atire	1	0	0
2018_2019	21/12/2018	3	atire	1	2	0
2018_2019	21/12/2018	3	atire	1	1	0
2018_2019	21/12/2018	3	atire	0	1	0

2018_2019	21/12/2018	3	atire	0	0	1
2018_2019	21/12/2018	3	atire	1	1	0
2018_2019	21/12/2018	3	atire	1	1	0
2018_2019	21/12/2018	3	atire	1	2	0
2018_2019	03/01/2019	4	kouare	1	0	0
2018_2019	03/01/2019	4	kouare	0	0	1
2018_2019	03/01/2019	4	kouare	1	0	0
2018_2019	03/01/2019	4	kouare	0	0	1
2018_2019	03/01/2019	4	kouare	0	0	1
2018_2019	03/01/2019	4	kouare	0	0	1
2018_2019	03/01/2019	4	kouare	1	0	0
2018_2019	09/01/2019	4	atire	0	0	1
2018_2019	09/01/2019	4	atire	1	0	0
2018_2019	09/01/2019	4	atire	1	1	0
2018_2019	09/01/2019	4	atire	0	0	1
2018_2019	09/01/2019	4	atire	1	0	0
2018_2019	09/01/2019	4	atire	0	0	1
2018_2019	09/01/2019	4	atire	1	0	0
2018_2019	09/01/2019	4	atire	1	0	0
2018_2019	09/01/2019	4	atire	0	0	1
2018_2019	09/01/2019	4	atire	0	2	0
2018_2019	09/01/2019	4	atire	0	1	0
2018_2019	09/01/2019	4	atire	1	0	0
2018_2019	09/01/2019	4	atire	1	1	0
2018_2019	09/01/2019	4	atire	0	4	0
2018_2019	09/01/2019	4	atire	0	1	0
2018_2019	09/01/2019	4	atire	0	1	0
2018_2019	09/01/2019	4	atire	1	0	0
2018_2019	09/01/2019	4	atire	0	3	0
2018_2019	09/01/2019	4	atire	1	1	0
2018_2019	09/01/2019	4	atire	1	0	0
2018_2019	09/01/2019	4	atire	0	0	1
2018_2019	09/01/2019	4	vua	1	0	0
2018_2019	09/01/2019	4	vua	1	1	0
2018_2019	09/01/2019	4	vua	1	1	0
2018_2019	09/01/2019	4	vua	1	1	0
2018_2019	09/01/2019	4	vua	1	0	0
2018_2019	09/01/2019	4	vua	1	0	0
2018_2019	09/01/2019	4	vua	1	2	0
2018_2019	09/01/2019	4	vua	0	1	0
2018_2019	09/01/2019	4	vua	1	0	0
2018_2019	09/01/2019	4	vua	1	0	0
2018_2019	09/01/2019	4	vua	0	0	1
2018_2019	09/01/2019	4	uaterembi	0	0	1
2018_2019	09/01/2019	4	uaterembi	0	1	0
2018_2019	09/01/2019	4	uaterembi	1	0	0
2018_2019	09/01/2019	4	uaterembi	0	1	0
2018_2019	09/01/2019	4	uaterembi	0	1	0
2018_2019	09/01/2019	4	uaterembi	0	0	1
2018_2019	09/01/2019	4	uaterembi	1	0	0

2018_2019	09/01/2019	4	uaterembi	1	2	0
2018_2019	09/01/2019	4	uaterembi	1	0	0
2018_2019	09/01/2019	4	uaterembi	0	0	1
2018_2019	09/01/2019	4	uaterembi	0	0	1
2018_2019	09/01/2019	4	uaterembi	1	0	0
2018_2019	09/01/2019	4	nda	1	0	0
2018_2019	09/01/2019	4	nda	0	0	1
2018_2019	09/01/2019	4	nda	0	0	1
2018_2019	09/01/2019	4	nda	0	0	1
2018_2019	09/01/2019	4	nda	1	0	0
2018_2019	09/01/2019	4	nda	0	0	1
2018_2019	09/01/2019	4	nge	1	0	0
2018_2019	09/01/2019	4	nge	1	0	0
2018_2019	09/01/2019	4	nge	1	0	0
2018_2019	09/01/2019	4	nge	1	0	0
2018_2019	09/01/2019	4	nge	0	2	0
2018_2019	09/01/2019	4	nge	1	1	0
2018_2019	09/01/2019	4	nge	0	0	1
2018_2019	09/01/2019	4	nge	0	1	0
2018_2019	09/01/2019	4	nge	0	0	1
2018_2019	09/01/2019	4	nge	0	1	0
2018_2019	09/01/2019	4	nge	0	1	0
2018_2019	09/01/2019	4	nge	1	0	0
2018_2019	09/01/2019	4	nge	0	3	0
2018_2019	09/01/2019	4	nge	1	0	0
2018_2019	09/01/2019	4	nge	1	0	0
2018_2019	09/01/2019	4	nge	1	1	0
2018_2019	09/01/2019	4	nge	1	0	0
2018_2019	09/01/2019	4	nge	0	2	0
2018_2019	09/01/2019	4	nge	1	0	0
2018_2019	09/01/2019	4	nge	0	1	0
2018_2019	09/01/2019	4	nge	1	0	0
2018_2019	09/01/2019	4	nge	1	1	0
2018_2019	09/01/2019	4	nge	1	0	0
2018_2019	09/01/2019	4	nge	0	4	0
2018_2019	10/01/2019	4	gi	1	1	0
2018_2019	10/01/2019	4	gi	1	0	0
2018_2019	10/01/2019	4	gi	1	1	0
2018_2019	10/01/2019	4	gi	1	2	0
2018_2019	10/01/2019	4	gi	0	1	0
2018_2019	10/01/2019	4	gi	1	0	0
2018_2019	10/01/2019	4	gi	0	2	0
2018_2019	10/01/2019	4	gi	0	1	0
2018_2019	10/01/2019	4	gi	0	1	0
2018_2019	10/01/2019	4	gi	1	0	0
2018_2019	10/01/2019	4	gi	1	0	0
2018_2019	10/01/2019	4	gi	1	1	0
2018_2019	10/01/2019	4	gi	1	1	0
2018_2019	10/01/2019	4	gi	0	1	0
2018_2019	10/01/2019	4	gi	0	3	0

2018_2019	10/01/2019	4 gi	0	2	0
2018_2019	10/01/2019	4 gi	1	1	0
2018_2019	10/01/2019	4 gi	1	0	0
2018_2019	10/01/2019	4 uatio	0	0	1
2018_2019	10/01/2019	4 uatio	1	1	0
2018_2019	10/01/2019	4 uatio	0	0	1
2018_2019	10/01/2019	4 uatio	0	1	0
2018_2019	10/01/2019	4 uatio	1	0	0
2018_2019	10/01/2019	4 uatio	1	0	0
2018_2019	10/01/2019	4 uatio	1	3	0
2018_2019	10/01/2019	4 uatio	0	0	1
2018_2019	10/01/2019	4 uatio	1	5	0
2018_2019	10/01/2019	4 uatio	1	0	0
2018_2019	10/01/2019	4 uatio	0	1	0
2018_2019	10/01/2019	4 redika	0	1	0
2018_2019	10/01/2019	4 redika	0	0	1
2018_2019	10/01/2019	4 redika	0	1	0
2018_2019	10/01/2019	4 redika	1	0	0
2018_2019	10/01/2019	4 redika	1	1	0
2018_2019	10/01/2019	4 redika	0	0	1
2018_2019	10/01/2019	4 redika	1	0	0
2018_2019	10/01/2019	4 redika	0	0	1
2018_2019	10/01/2019	4 redika	1	0	0
2018_2019	10/01/2019	4 redika	1	0	0
2018_2019	10/01/2019	4 redika	0	2	0
2018_2019	23/01/2019	5 kie	1	0	0
2018_2019	23/01/2019	5 kie	1	0	0
2018_2019	23/01/2019	5 kie	1	1	0
2018_2019	23/01/2019	5 kie	1	0	0
2018_2019	23/01/2019	5 kie	1	0	0
2018_2019	23/01/2019	5 kie	1	0	0
2018_2019	23/01/2019	5 kie	1	1	0
2018_2019	23/01/2019	5 kie	1	0	0
2018_2019	23/01/2019	5 kie	1	0	0
2018_2019	23/01/2019	5 kie	1	0	0
2018_2019	23/01/2019	5 kie	1	0	0
2018_2019	23/01/2019	5 kie	1	0	0
2018_2019	23/01/2019	5 kie	1	0	0
2018_2019	23/01/2019	5 kie	1	0	0
2018_2019	23/01/2019	5 kie	1	0	0
2018_2019	23/01/2019	5 kie	1	1	0
2018_2019	23/01/2019	5 kie	1	0	0
2018_2019	23/01/2019	5 kie	1	0	0
2018_2019	23/01/2019	5 kie	0	1	0
2018_2019	23/01/2019	5 kie	1	0	0
2018_2019	23/01/2019	5 amere	1	0	0
2018_2019	23/01/2019	5 amere	1	0	0
2018_2019	23/01/2019	5 amere	0	0	1
2018_2019	23/01/2019	5 amere	1	0	0
2018_2019	23/01/2019	5 amere	1	1	0

2018_2019	23/01/2019	5 amere	1	0	0
2018_2019	23/01/2019	5 amere	1	0	0
2018_2019	23/01/2019	5 amere	0	1	0
2018_2019	23/01/2019	5 amere	0	1	0
2018_2019	23/01/2019	5 amere	0	1	0
2018_2019	23/01/2019	5 amere	1	0	0
2018_2019	23/01/2019	5 amere	1	0	0
2018_2019	23/01/2019	5 amere	1	0	0
2018_2019	23/01/2019	5 amere	1	3	0
2018_2019	23/01/2019	5 du ami	1	0	0
2018_2019	23/01/2019	5 du ami	0	5	0
2018_2019	24/01/2019	5 koko	1	0	0
2018_2019	24/01/2019	5 koko	1	1	0
2018_2019	24/01/2019	5 koko	1	0	0
2018_2019	24/01/2019	5 koko	0	0	1
2018_2019	24/01/2019	5 koko	1	1	0
2018_2019	24/01/2019	5 koko	0	0	1
2018_2019	24/01/2019	5 koko	1	0	0
2018_2019	24/01/2019	5 koko	0	1	0
2018_2019	24/01/2019	5 koko	1	5	0
2018_2019	24/01/2019	5 koko	1	0	0
2018_2019	24/01/2019	5 petit koko	1	0	0
2018_2019	24/01/2019	5 petit koko	1	0	0
2018_2019	24/01/2019	5 petit koko	0	1	0
2018_2019	24/01/2019	5 petit koko	1	0	0
2018_2019	24/01/2019	5 totea	1	0	0
2018_2019	24/01/2019	5 totea	1	0	0
2018_2019	24/01/2019	5 totea	1	7	0
2018_2019	24/01/2019	5 totea	0	1	0
2018_2019	24/01/2019	5 ndo	1	0	0
2018_2019	24/01/2019	5 uie	1	0	0
2018_2019	24/01/2019	5 uie	0	3	0
2018_2019	25/01/2019	5 uo	1	1	0
2018_2019	25/01/2019	5 uo	1	0	0
2018_2019	25/01/2019	5 mato	1	0	0
2018_2019	25/01/2019	5 noe	0	1	0
2019_2020	27/11/2019	1 atire	1	0	0
2019_2020	27/11/2019	1 uaterembi	0	0	1
2019_2020	27/11/2019	1 uaterembi	0	0	1
2019_2020	27/11/2019	1 uaterembi	0	0	1
2019_2020	27/11/2019	1 uaterembi	0	0	1
2019_2020	27/11/2019	1 uaterembi	0	0	1
2019_2020	27/11/2019	1 uaterembi	1	0	0
2019_2020	27/11/2019	1 uaterembi	0	4	0
2019_2020	27/11/2019	1 uaterembi	0	2	0
2019_2020	27/11/2019	1 uaterembi	0	1	0
2019_2020	27/11/2019	1 uaterembi	0	2	0
2019_2020	27/11/2019	1 uaterembi	0	1	0
2019_2020	27/11/2019	1 uaterembi	0	0	1
2019_2020	27/11/2019	1 uaterembi	0	0	1

2019_2020	28/11/2019	1	nge	0	0	1
2019_2020	28/11/2019	1	nge	0	0	1
2019_2020	28/11/2019	1	gi	1	0	0
2019_2020	28/11/2019	1	gi	1	0	0
2019_2020	28/11/2019	1	gi	1	1	0
2019_2020	28/11/2019	1	gi	0	1	0
2019_2020	28/11/2019	1	gi	0	1	0
2019_2020	28/11/2019	1	gi	0	1	0
2019_2020	28/11/2019	1	gi	1	2	0
2019_2020	28/11/2019	1	gi	0	1	0
2019_2020	28/11/2019	1	gi	1	0	0
2019_2020	28/11/2019	1	gi	0	5	0
2019_2020	28/11/2019	1	gi	0	1	0
2019_2020	28/11/2019	1	gi	0	0	1
2019_2020	28/11/2019	1	uatio	0	1	0
2019_2020	28/11/2019	1	uatio	0	1	0
2019_2020	28/11/2019	1	uatio	0	2	0
2019_2020	28/11/2019	1	uatio	1	1	0
2019_2020	28/11/2019	1	uatio	0	0	1
2019_2020	28/11/2019	1	uatio	0	1	0
2019_2020	28/11/2019	1	redika	0	0	1
2019_2020	28/11/2019	1	redika	0	1	0
2019_2020	27/11/2019	1	kouare	0	0	0
2019_2020	27/11/2019	1	nda	0	0	0
2019_2020	27/11/2019	1	tere	0	0	0
2019_2020	28/11/2019	1	ieroue	0	0	0
2019_2020	04/12/2019	2	redika	0	1	0
2019_2020	04/12/2019	2	redika	0	1	0
2019_2020	04/12/2019	2	redika	0	2	0
2019_2020	04/12/2019	2	redika	0	0	1
2019_2020	04/12/2019	2	redika	0	1	0
2019_2020	04/12/2019	2	atire	0	4	0
2019_2020	04/12/2019	2	atire	0	8	0
2019_2020	04/12/2019	2	atire	0	1	0
2019_2020	04/12/2019	2	atire	1	1	0
2019_2020	04/12/2019	2	atire	0	1	0
2019_2020	04/12/2019	2	atire	0	7	0
2019_2020	04/12/2019	2	atire	0	1	0
2019_2020	04/12/2019	2	atire	1	1	0
2019_2020	04/12/2019	2	atire	0	0	1
2019_2020	04/12/2019	2	ieroue	0	1	0
2019_2020	04/12/2019	2	ieroue	0	0	1
2019_2020	04/12/2019	2	ieroue	1	0	0
2019_2020	04/12/2019	2	nge	0	0	1
2019_2020	04/12/2019	2	nge	0	0	1
2019_2020	04/12/2019	2	nge	0	0	1
2019_2020	04/12/2019	2	nge	0	1	0
2019_2020	04/12/2019	2	nge	1	0	0
2019_2020	04/12/2019	2	nge	0	0	1
2019_2020	04/12/2019	2	nge	1	0	0

2019_2020	04/12/2019	2	nge	0	1	0
2019_2020	04/12/2019	2	nge	1	2	0
2019_2020	04/12/2019	2	nge	1	0	0
2019_2020	04/12/2019	2	nge	1	0	0
2019_2020	05/12/2019	2	nge	1	0	0
2019_2020	05/12/2019	2	gi	0	0	1
2019_2020	05/12/2019	2	gi	1	0	0
2019_2020	05/12/2019	2	nda	0	3	0
2019_2020	05/12/2019	2	nda	1	0	0
2019_2020	05/12/2019	2	nda	0	1	0
2019_2020	05/12/2019	2	nda	0	0	1
2019_2020	05/12/2019	2	nda	0	0	1
2019_2020	05/12/2019	2	nda	1	0	0
2019_2020	05/12/2019	2	kouare	0	1	0
2019_2020	05/12/2019	2	kouare	0	3	0
2019_2020	05/12/2019	2	kouare	0	3	0
2019_2020	05/12/2019	2	kouare	0	1	0
2019_2020	05/12/2019	2	uatio	0	2	0
2019_2020	05/12/2019	2	uatio	1	0	0
2019_2020	05/12/2019	2	uatio	0	0	1
2019_2020	05/12/2019	2	uatio	0	0	1
2019_2020	05/12/2019	2	uatio	0	0	1
2019_2020	05/12/2019	2	uaterembi	0	0	1
2019_2020	05/12/2019	2	uaterembi	0	2	0
2019_2020	05/12/2019	2	uaterembi	0	1	0
2019_2020	05/12/2019	2	uaterembi	0	1	0
2019_2020	05/12/2019	2	uaterembi	0	1	0
2019_2020	05/12/2019	2	uaterembi	0	1	0
2019_2020	05/12/2019	2	uaterembi	0	1	0
2019_2020	05/12/2019	2	uaterembi	0	5	0
2019_2020	05/12/2019	2	uaterembi	0	1	0
2019_2020	05/12/2019	2	uaterembi	0	1	0
2019_2020	05/12/2019	2	uaterembi	0	0	1
2019_2020	05/12/2019	2	uaterembi	0	1	0
2019_2020	05/12/2019	2	uaterembi	0	0	1
2019_2020	05/12/2019	2	uaterembi	0	0	1
2019_2020	05/12/2019	2	uaterembi	1	0	0
2019_2020	05/12/2019	2	uaterembi	0	1	0
2019_2020	05/12/2019	2	uaterembi	0	0	1
2019_2020	05/12/2019	2	vua	1	1	0
2019_2020	05/12/2019	2	vua	0	0	1
2019_2020	05/12/2019	2	vua	0	0	1
2019_2020	05/12/2019	2	vua	0	2	0
2019_2020	05/12/2019	2	vua	0	2	0
2019_2020	05/12/2019	2	vua	0	1	0
2019_2020	05/12/2019	2	vua	0	0	1
2019_2020	05/12/2019	2	vua	0	0	1
2019_2020	05/12/2019	2	vua	1	1	0
2019_2020	05/12/2019	2	vua	1	1	0
2019_2020	05/12/2019	2	tere	0	0	0

2019_2020	10/12/2019	3 atire	0	1	0
2019_2020	10/12/2019	3 atire	0	1	0
2019_2020	10/12/2019	3 atire	1	0	0
2019_2020	10/12/2019	3 atire	0	3	0
2019_2020	10/12/2019	3 atire	0	2	0
2019_2020	10/12/2019	3 atire	0	1	0
2019_2020	10/12/2019	3 atire	0	1	0
2019_2020	10/12/2019	3 atire	0	2	0
2019_2020	10/12/2019	3 atire	1	0	0
2019_2020	10/12/2019	3 atire	1	0	0
2019_2020	10/12/2019	3 atire	0	0	1
2019_2020	10/12/2019	3 atire	0	2	0
2019_2020	10/12/2019	3 atire	0	2	0
2019_2020	10/12/2019	3 atire	0	2	0
2019_2020	10/12/2019	3 atire	1	0	0
2019_2020	23/12/2019	4 atire	1	1	0
2019_2020	23/12/2019	4 atire	0	3	0
2019_2020	23/12/2019	4 atire	0	2	0
2019_2020	23/12/2019	4 atire	0	3	0
2019_2020	23/12/2019	4 atire	1	0	0
2019_2020	23/12/2019	4 atire	0	0	1
2019_2020	23/12/2019	4 atire	0	3	0
2019_2020	23/12/2019	4 atire	1	0	0
2019_2020	24/12/2019	4 atire	1	3	0
2019_2020	24/12/2019	4 atire	0	1	0
2019_2020	24/12/2019	4 atire	1	3	0
2019_2020	24/12/2019	4 atire	0	5	0
2019_2020	24/12/2019	4 atire	0	3	0
2019_2020	24/12/2019	4 atire	0	1	0
2019_2020	24/12/2019	4 atire	0	3	0
2019_2020	24/12/2019	4 atire	0	3	0
2019_2020	24/12/2019	4 atire	0	2	0
2019_2020	24/12/2019	4 atire	1	0	0
2019_2020	24/12/2019	4 atire	0	4	0
2019_2020	02/01/2020	5 redika	0	1	0
2019_2020	02/01/2020	5 redika	1	0	0
2019_2020	02/01/2020	5 redika	0	1	0
2019_2020	02/01/2020	5 redika	0	0	1
2019_2020	02/01/2020	5 redika	0	1	0
2019_2020	02/01/2020	5 redika	0	1	0
2019_2020	02/01/2020	5 redika	0	2	0
2019_2020	02/01/2020	5 redika	1	0	0
2019_2020	02/01/2020	5 redika	0	0	1
2019_2020	02/01/2020	5 redika	0	1	0
2019_2020	02/01/2020	5 redika	0	1	0
2019_2020	02/01/2020	5 redika	0	1	0
2019_2020	02/01/2020	5 redika	0	1	0
2019_2020	02/01/2020	5 redika	1	2	0
2019_2020	02/01/2020	5 redika	0	3	0
2019_2020	02/01/2020	5 redika	0	1	0

2019_2020	02/01/2020	5	redika	0	3	0
2019_2020	02/01/2020	5	redika	0	0	1
2019_2020	02/01/2020	5	redika	1	0	0
2019_2020	02/01/2020	5	redika	0	1	0
2019_2020	02/01/2020	5	redika	0	1	0
2019_2020	02/01/2020	5	redika	0	1	0
2019_2020	02/01/2020	5	redika	0	1	0
2019_2020	02/01/2020	5	redika	0	2	0
2019_2020	02/01/2020	5	redika	0	1	0
2019_2020	02/01/2020	5	redika	0	1	0
2019_2020	02/01/2020	5	redika	0	1	0
2019_2020	02/01/2020	5	redika	0	1	0
2019_2020	02/01/2020	5	redika	0	1	0
2019_2020	02/01/2020	5	redika	1	1	0
2019_2020	02/01/2020	5	redika	0	4	0
2019_2020	02/01/2020	5	redika	0	3	0
2019_2020	02/01/2020	5	redika	0	5	0
2019_2020	02/01/2020	5	redika	0	3	0
2019_2020	02/01/2020	5	redika	0	1	0
2019_2020	02/01/2020	5	redika	1	0	0
2019_2020	02/01/2020	5	redika	0	1	0
2019_2020	02/01/2020	5	redika	0	2	0
2019_2020	02/01/2020	5	redika	0	2	0
2019_2020	02/01/2020	5	uaterembi	1	0	0
2019_2020	02/01/2020	5	uaterembi	0	1	0
2019_2020	02/01/2020	5	uaterembi	0	0	1
2019_2020	02/01/2020	5	uaterembi	0	0	1
2019_2020	02/01/2020	5	uaterembi	0	0	1
2019_2020	02/01/2020	5	uaterembi	0	5	0
2019_2020	02/01/2020	5	uaterembi	0	0	1
2019_2020	02/01/2020	5	uaterembi	0	3	0
2019_2020	02/01/2020	5	uaterembi	0	0	1
2019_2020	02/01/2020	5	uaterembi	0	2	0
2019_2020	02/01/2020	5	uaterembi	0	2	0
2019_2020	02/01/2020	5	uaterembi	0	1	0
2019_2020	02/01/2020	5	uaterembi	0	3	0
2019_2020	02/01/2020	5	uaterembi	0	2	0
2019_2020	02/01/2020	5	uaterembi	0	1	0
2019_2020	02/01/2020	5	uaterembi	0	0	1
2019_2020	02/01/2020	5	uatio	0	2	0
2019_2020	02/01/2020	5	uatio	1	0	0
2019_2020	02/01/2020	5	uatio	0	2	0
2019_2020	02/01/2020	5	uatio	0	2	0
2019_2020	02/01/2020	5	uatio	0	2	0
2019_2020	02/01/2020	5	uatio	0	2	0
2019_2020	02/01/2020	5	uatio	0	2	0
2019_2020	02/01/2020	5	uatio	0	2	0
2019_2020	02/01/2020	5	uatio	1	0	0
2019_2020	02/01/2020	5	uatio	0	2	0
2019_2020	02/01/2020	5	uatio	1	0	0

2019_2020	02/01/2020	5	uatio	1	0	0
2019_2020	02/01/2020	5	uatio	0	4	0
2019_2020	02/01/2020	5	uatio	0	1	0
2019_2020	02/01/2020	5	uatio	0	1	0
2019_2020	02/01/2020	5	uatio	0	0	1
2019_2020	02/01/2020	5	uatio	0	0	1
2019_2020	02/01/2020	5	uatio	0	0	1
2019_2020	02/01/2020	5	gi	1	0	0
2019_2020	02/01/2020	5	gi	1	4	0
2019_2020	02/01/2020	5	gi	1	0	0
2019_2020	02/01/2020	5	gi	1	2	0
2019_2020	02/01/2020	5	gi	1	2	0
2019_2020	02/01/2020	5	gi	0	1	0
2019_2020	02/01/2020	5	gi	0	0	1
2019_2020	02/01/2020	5	gi	0	3	0
2019_2020	02/01/2020	5	nge	0	2	0
2019_2020	02/01/2020	5	nge	0	3	0
2019_2020	02/01/2020	5	nge	1	0	0
2019_2020	02/01/2020	5	nge	0	4	0
2019_2020	02/01/2020	5	nge	0	8	0
2019_2020	02/01/2020	5	nge	1	0	0
2019_2020	02/01/2020	5	nge	1	1	0
2019_2020	02/01/2020	5	nge	0	4	0
2019_2020	02/01/2020	5	nge	1	1	0
2019_2020	03/01/2020	5	kouare	0	0	1
2019_2020	03/01/2020	5	kouare	1	0	0
2019_2020	03/01/2020	5	kouare	1	0	0
2019_2020	03/01/2020	5	uie	0	4	0
2019_2020	03/01/2020	5	uie	0	6	0
2019_2020	03/01/2020	5	uie	0	2	0
2019_2020	03/01/2020	5	uie	0	12	0
2019_2020	03/01/2020	5	uie	1	0	0
2019_2020	03/01/2020	5	uie	1	0	0
2019_2020	03/01/2020	5	uie	1	0	0
2019_2020	03/01/2020	5	uie	0	2	0
2019_2020	03/01/2020	5	uie	1	0	0
2019_2020	03/01/2020	5	uie	1	0	0
2019_2020	03/01/2020	5	uie	0	4	0
2019_2020	03/01/2020	5	uie	0	1	0
2019_2020	03/01/2020	5	uie	0	3	0
2019_2020	03/01/2020	5	puemba	1	0	0
2019_2020	03/01/2020	5	puemba	1	0	0
2019_2020	03/01/2020	5	puemba	1	1	0
2019_2020	03/01/2020	5	puemba	1	0	0
2019_2020	03/01/2020	5	uo	0	1	0
2019_2020	03/01/2020	5	uo	0	2	0
2019_2020	03/01/2020	5	uo	0	2	0
2019_2020	03/01/2020	5	uo	1	0	0
2019_2020	03/01/2020	5	uo	0	2	0
2019_2020	02/01/2020	5	redika	0	2	0

2019_2020	02/01/2020	5	nge	0	1	0
2019_2020	03/01/2020	5	noe	0	0	0
2019_2020	09/01/2020	6	ieroue	0	1	0
2019_2020	09/01/2020	6	ieroue	0	2	0
2019_2020	09/01/2020	6	ieroue	0	2	0
2019_2020	09/01/2020	6	ieroue	0	1	0
2019_2020	09/01/2020	6	ieroue	0	1	0
2019_2020	09/01/2020	6	ieroue	0	1	0
2019_2020	09/01/2020	6	ieroue	0	3	0
2019_2020	09/01/2020	6	ieroue	1	0	0
2019_2020	09/01/2020	6	ieroue	0	2	0
2019_2020	09/01/2020	6	gi	0	0	1
2019_2020	16/01/2020	7	redika	1	0	0
2019_2020	16/01/2020	7	redika	0	1	0
2019_2020	16/01/2020	7	redika	1	0	0
2019_2020	16/01/2020	7	redika	1	0	0
2019_2020	16/01/2020	7	redika	1	0	0
2019_2020	16/01/2020	7	redika	1	0	0
2019_2020	16/01/2020	7	redika	1	0	0
2019_2020	16/01/2020	7	uatio	1	0	0
2019_2020	16/01/2020	7	kouare	1	0	0
2019_2020	16/01/2020	7	kouare	1	0	0
2019_2020	16/01/2020	7	gi	1	0	0
2019_2020	16/01/2020	7	gi	1	0	0
2019_2020	16/01/2020	7	gi	0	0	1
2019_2020	16/01/2020	7	nge	0	0	1
2019_2020	16/01/2020	7	nge	0	0	1
2019_2020	16/01/2020	7	nge	1	0	0
2019_2020	16/01/2020	7	nge	1	0	0
2019_2020	17/01/2020	7	uie	1	0	0
2019_2020	17/01/2020	7	uie	1	1	0
2019_2020	17/01/2020	7	noe	0	1	0
2019_2020	17/01/2020	7	noe	1	1	0
2019_2020	17/01/2020	7	noe	1	0	0
2019_2020	17/01/2020	7	puemba	0	0	1
2019_2020	17/01/2020	7	puemba	0	0	1
2019_2020	17/01/2020	7	puemba	0	0	1
2019_2020	17/01/2020	7	uo	1	0	0
2019_2020	17/01/2020	7	ieroue	0	0	0
2019_2020	22/01/2020	8	ndo	1	0	0
2019_2020	22/01/2020	8	ndo	1	3	0
2019_2020	22/01/2020	8	ndo	1	0	0
2019_2020	22/01/2020	8	ndo	0	1	0
2019_2020	22/01/2020	8	ndo	1	0	0
2019_2020	22/01/2020	8	ndo	0	3	0
2019_2020	22/01/2020	8	ndo	1	0	0
2019_2020	22/01/2020	8	ndo	1	0	0
2019_2020	22/01/2020	8	ndo	1	0	0
2019_2020	22/01/2020	8	ndo	1	2	0
2019_2020	22/01/2020	8	ndo	1	0	0

2019_2020	22/01/2020	8	ndo	1	2	0
2019_2020	22/01/2020	8	ndo	0	4	0
2019_2020	22/01/2020	8	ndo	0	1	0
2019_2020	22/01/2020	8	ndo	1	1	0
2019_2020	22/01/2020	8	ndo	0	2	0
2019_2020	22/01/2020	8	ua	0	1	0
2019_2020	22/01/2020	8	ua	1	2	0
2019_2020	22/01/2020	8	ua	1	3	0
2019_2020	22/01/2020	8	ua	1	0	0
2019_2020	22/01/2020	8	ua	0	1	0
2019_2020	22/01/2020	8	ua	1	0	0
2019_2020	22/01/2020	8	ua	1	0	0
2019_2020	23/01/2020	8	mato	0	2	0
2019_2020	23/01/2020	8	mato	1	2	0
2019_2020	23/01/2020	8	mato	0	2	0
2019_2020	23/01/2020	8	mato	0	1	0
2019_2020	23/01/2020	8	mato	0	1	0
2019_2020	23/01/2020	8	mato	1	0	0
2019_2020	23/01/2020	8	mato	0	1	0
2019_2020	23/01/2020	8	mato	0	1	0
2019_2020	23/01/2020	8	mato	0	1	0
2019_2020	23/01/2020	8	mato	0	2	0
2019_2020	23/01/2020	8	pumbo	0	2	0
2019_2020	23/01/2020	8	pumbo	0	1	0
2019_2020	22/01/2020	8	ugo	0	0	0
2019_2020	22/01/2020	8	nouare	0	0	0
2019_2020	28/01/2020	9	redika	1	2	0
2019_2020	28/01/2020	9	redika	0	0	1
2019_2020	28/01/2020	9	redika	0	1	0
2019_2020	28/01/2020	9	redika	0	1	0
2019_2020	28/01/2020	9	redika	0	1	0
2019_2020	28/01/2020	9	redika	0	1	0
2019_2020	28/01/2020	9	redika	0	1	0
2019_2020	28/01/2020	9	redika	1	0	0
2019_2020	28/01/2020	9	atire	0	1	0
2019_2020	28/01/2020	9	atire	1	0	0
2019_2020	28/01/2020	9	atire	1	0	0
2019_2020	28/01/2020	9	atire	1	0	0
2019_2020	28/01/2020	9	atire	1	0	0
2019_2020	28/01/2020	9	atire	1	0	0
2019_2020	28/01/2020	9	atire	0	1	0
2019_2020	28/01/2020	9	uaterembi	1	0	0
2019_2020	28/01/2020	9	uaterembi	0	2	0
2019_2020	28/01/2020	9	uaterembi	1	0	0
2019_2020	28/01/2020	9	uaterembi	0	1	0
2019_2020	28/01/2020	9	uaterembi	0	0	1
2019_2020	28/01/2020	9	uaterembi	0	0	1
2019_2020	28/01/2020	9	uaterembi	0	0	1
2019_2020	28/01/2020	9	uaterembi	0	0	1
2019_2020	28/01/2020	9	uatio	0	1	0

2019_2020	28/01/2020	9	uatio	0	0	1
2019_2020	28/01/2020	9	uatio	1	0	0
2019_2020	28/01/2020	9	uatio	1	0	0
2019_2020	28/01/2020	9	uatio	1	0	0
2019_2020	28/01/2020	9	uatio	1	0	0
2019_2020	28/01/2020	9	uatio	1	2	0
2019_2020	28/01/2020	9	uatio	0	0	1
2019_2020	28/01/2020	9	ua	1	0	0
2019_2020	28/01/2020	9	ua	1	0	0
2019_2020	28/01/2020	9	ua	0	1	0
2019_2020	28/01/2020	9	ua	1	0	0
2019_2020	28/01/2020	9	kouare	0	1	0
2019_2020	28/01/2020	9	nge	1	0	0
2019_2020	28/01/2020	9	nge	1	0	0
2019_2020	28/01/2020	9	nge	0	0	1
2019_2020	28/01/2020	9	nge	1	0	0
2019_2020	28/01/2020	9	nge	1	2	0
2019_2020	28/01/2020	9	nge	1	0	0
2019_2020	29/01/2020	9	uie	0	2	0
2019_2020	29/01/2020	9	uie	0	8	0
2019_2020	29/01/2020	9	uie	1	0	0
2019_2020	29/01/2020	9	ieroue	1	1	0
2019_2020	29/01/2020	9	noe	1	0	0
2019_2020	29/01/2020	9	noe	0	0	1
2019_2020	29/01/2020	9	noe	0	0	1
2019_2020	29/01/2020	9	noe	0	2	0
2019_2020	29/01/2020	9	puemba	1	0	0
2019_2020	29/01/2020	9	puemba	1	0	0
2019_2020	29/01/2020	9	uo	0	0	0
2020_2021	28/11/2020	1	atire	0	1	0
2020_2021	28/11/2020	1	atire	1	0	0
2020_2021	28/11/2020	1	vua	0	1	0
2020_2021	28/11/2020	1	vua	1	1	0
2020_2021	28/11/2020	1	vua	0	0	1
2020_2021	28/11/2020	1	ieroue	1	1	0
2020_2021	28/11/2020	1	kouare	0	0	1
2020_2021	28/11/2020	1	kouare	0	0	1
2020_2021	28/11/2020	1	kouare	1	4	0
2020_2021	28/11/2020	1	kouare	0	0	1
2020_2021	28/11/2020	1	kouare	1	0	0
2020_2021	28/11/2020	1	tere	0	0	0
2020_2021	28/11/2020	1	nda	0	0	1
2020_2021	28/11/2020	1	nda	0	1	0
2020_2021	28/11/2020	1	nda	0	0	1
2020_2021	28/11/2020	1	nda	0	0	1
2020_2021	28/11/2020	1	nda	1	1	0
2020_2021	28/11/2020	1	nda	0	0	1
2020_2021	28/11/2020	1	nda	0	1	0
2020_2021	28/11/2020	1	nda	1	0	0
2020_2021	28/11/2020	1	nda	0	1	0

2020_2021	28/11/2020	1	nda	0	2	0
2020_2021	28/11/2020	1	nge	0	1	0
2020_2021	28/11/2020	1	nge	0	1	0
2020_2021	28/11/2020	1	nge	0	3	0
2020_2021	28/11/2020	1	nge	0	1	0
2020_2021	28/11/2020	1	nge	1	1	0
2020_2021	28/11/2020	1	nge	0	2	0
2020_2021	29/11/2020	1	gi	1	0	0
2020_2021	29/11/2020	1	gi	1	0	0
2020_2021	29/11/2020	1	gi	1	0	0
2020_2021	29/11/2020	1	ua	1	1	0
2020_2021	29/11/2020	1	uatio	0	0	1
2020_2021	29/11/2020	1	uatio	0	0	1
2020_2021	29/11/2020	1	uatio	1	1	0
2020_2021	29/11/2020	1	uatio	0	1	0
2020_2021	29/11/2020	1	uatio	1	1	0
2020_2021	29/11/2020	1	uaterembi	0	0	0
2020_2021	29/11/2020	1	redika	0	1	0
2020_2021	29/11/2020	1	redika	0	4	0
2020_2021	29/11/2020	1	redika	1	0	0
2020_2021	08/12/2020	2	redika	0	2	0
2020_2021	08/12/2020	2	ieroue	0	0	0
2020_2021	08/12/2020	2	ua	0	0	0
2020_2021	08/12/2020	2	uatio	0	1	0
2020_2021	08/12/2020	2	uatio	0	0	1
2020_2021	08/12/2020	2	uatio	0	0	1
2020_2021	08/12/2020	2	uaterembi	0	1	0
2020_2021	08/12/2020	2	uaterembi	0	0	1
2020_2021	08/12/2020	2	uaterembi	1	1	0
2020_2021	08/12/2020	2	uaterembi	0	1	0
2020_2021	08/12/2020	2	uaterembi	0	4	0
2020_2021	08/12/2020	2	uaterembi	0	2	0
2020_2021	08/12/2020	2	uaterembi	0	0	1
2020_2021	08/12/2020	2	gi	0	0	1
2020_2021	08/12/2020	2	gi	0	1	0
2020_2021	08/12/2020	2	gi	0	6	0
2020_2021	08/12/2020	2	gi	0	1	0
2020_2021	08/12/2020	2	gi	0	1	0
2020_2021	08/12/2020	2	gi	1	0	0
2020_2021	08/12/2020	2	gi	0	3	0
2020_2021	08/12/2020	2	nge	0	2	0
2020_2021	08/12/2020	2	nge	1	0	0
2020_2021	08/12/2020	2	nge	1	0	0
2020_2021	08/12/2020	2	nge	1	3	0
2020_2021	08/12/2020	2	nge	0	2	0
2020_2021	08/12/2020	2	nge	0	5	0
2020_2021	08/12/2020	2	nge	0	3	0
2020_2021	08/12/2020	2	nge	0	4	0
2020_2021	08/12/2020	2	nge	1	0	0
2020_2021	08/12/2020	2	nge	0	2	0

2020_2021	08/12/2020	2	nge	0	1	0
2020_2021	08/12/2020	2	nge	1	1	0
2020_2021	08/12/2020	2	nge	0	4	0
2020_2021	08/12/2020	2	nge	0	4	0
2020_2021	08/12/2020	2	nge	0	1	0
2020_2021	09/12/2020	2	tere	0	0	0
2020_2021	09/12/2020	2	nda	1	0	0
2020_2021	09/12/2020	2	nda	0	1	0
2020_2021	09/12/2020	2	nda	0	1	0
2020_2021	09/12/2020	2	kouare	1	0	0
2020_2021	09/12/2020	2	kouare	0	4	0
2020_2021	09/12/2020	2	kouare	0	9	0
2020_2021	09/12/2020	2	kouare	0	0	1
2020_2021	09/12/2020	2	kouare	0	1	0
2020_2021	09/12/2020	2	kouare	1	0	0
2020_2021	09/12/2020	2	kouare	0	0	1
2020_2021	09/12/2020	2	kouare	0	0	1
2020_2021	09/12/2020	2	kouare	1	0	0
2020_2021	09/12/2020	2	kouare	1	0	0
2020_2021	09/12/2020	2	kouare	0	0	1
2020_2021	09/12/2020	2	kouare	0	0	1
2020_2021	09/12/2020	2	vua	0	0	1
2020_2021	09/12/2020	2	vua	1	0	0
2020_2021	09/12/2020	2	vua	0	0	1
2020_2021	09/12/2020	2	atire	1	0	0
2020_2021	09/12/2020	2	atire	1	0	0
2020_2021	09/12/2020	2	atire	1	0	0
2020_2021	09/12/2020	2	atire	1	0	0
2020_2021	09/12/2020	2	atire	1	1	0
2020_2021	09/12/2020	2	atire	0	2	0
2020_2021	09/12/2020	2	atire	1	1	0
2020_2021	09/12/2020	2	atire	0	1	0
2020_2021	09/12/2020	2	atire	0	1	0
2020_2021	09/12/2020	2	atire	0	1	0
2020_2021	22/12/2020	3	uo	0	0	0
2020_2021	22/12/2020	3	uie	1	2	0
2020_2021	22/12/2020	3	uie	0	1	0
2020_2021	22/12/2020	3	ndo	1	0	0
2020_2021	22/12/2020	3	ndo	0	1	0
2020_2021	22/12/2020	3	totea	1	0	0
2020_2021	22/12/2020	3	totea	0	1	0
2020_2021	22/12/2020	3	mbore	1	0	0
2020_2021	22/12/2020	3	mbore	1	1	0
2020_2021	22/12/2020	3	petit koko	0	0	0
2020_2021	23/12/2020	3	mato	0	1	0
2020_2021	23/12/2020	3	mato	0	1	0
2020_2021	23/12/2020	3	noe	0	0	0
2020_2021	23/12/2020	3	puemba	1	0	0
2020_2021	23/12/2020	3	pumbo	0	1	0
2020_2021	23/12/2020	3	ugo	0	0	0

2020_2021	23/12/2020	3	nouare	0	6	0
2020_2021	23/12/2020	3	nouare	0	1	0
2020_2021	22/12/2020	4	redika	0	0	0
2020_2021	22/12/2020	4	vua	1	0	0
2020_2021	22/12/2020	4	vua	0	1	0
2020_2021	22/12/2020	4	vua	1	0	0
2020_2021	22/12/2020	4	vua	0	1	0
2020_2021	22/12/2020	4	vua	0	1	0
2020_2021	22/12/2020	4	vua	1	1	0
2020_2021	22/12/2020	4	vua	1	1	0
2020_2021	22/12/2020	4	vua	1	0	0
2020_2021	22/12/2020	4	uaterembi	0	0	1
2020_2021	22/12/2020	4	uaterembi	0	0	1
2020_2021	22/12/2020	4	uaterembi	0	0	1
2020_2021	22/12/2020	4	uaterembi	0	0	1
2020_2021	22/12/2020	4	uaterembi	0	0	1
2020_2021	22/12/2020	4	uaterembi	0	1	0
2020_2021	22/12/2020	4	uaterembi	0	0	1
2020_2021	22/12/2020	4	uaterembi	0	1	0
2020_2021	22/12/2020	4	uaterembi	1	1	0
2020_2021	22/12/2020	4	uaterembi	1	0	0
2020_2021	22/12/2020	4	uaterembi	0	1	0
2020_2021	22/12/2020	4	uatio	0	3	0
2020_2021	22/12/2020	4	uatio	0	4	0
2020_2021	22/12/2020	4	uatio	0	2	0
2020_2021	22/12/2020	4	gi	1	2	0
2020_2021	22/12/2020	4	gi	0	2	0
2020_2021	22/12/2020	4	gi	1	6	0
2020_2021	22/12/2020	4	gi	0	2	0
2020_2021	22/12/2020	4	gi	0	1	0
2020_2021	22/12/2020	4	gi	1	0	0
2020_2021	22/12/2020	4	gi	1	0	0
2020_2021	23/12/2020	4	nge	1	0	0
2020_2021	23/12/2020	4	nge	0	4	0
2020_2021	23/12/2020	4	nge	1	0	0
2020_2021	23/12/2020	4	nge	0	1	0
2020_2021	23/12/2020	4	nge	0	4	0
2020_2021	23/12/2020	4	nge	1	1	0
2020_2021	23/12/2020	4	nge	1	0	0
2020_2021	23/12/2020	4	nge	0	4	0
2020_2021	23/12/2020	4	nge	0	6	0
2020_2021	23/12/2020	4	nge	0	2	0
2020_2021	23/12/2020	4	nge	0	1	0
2020_2021	23/12/2020	4	nge	0	2	0
2020_2021	23/12/2020	4	nge	1	2	0
2020_2021	23/12/2020	4	nge	0	1	0
2020_2021	23/12/2020	4	nge	0	1	0
2020_2021	23/12/2020	4	nge	0	2	0
2020_2021	23/12/2020	4	kouare	0	0	1
2020_2021	23/12/2020	4	kouare	1	0	0

2020_2021	23/12/2020	4	kouare	1	1	0
2020_2021	23/12/2020	4	kouare	0	0	1
2020_2021	23/12/2020	4	kouare	1	0	0
2020_2021	23/12/2020	4	nda	0	2	0
2020_2021	23/12/2020	4	ieroue	1	0	0
2020_2021	23/12/2020	4	ieroue	0	1	0
2020_2021	23/12/2020	4	atire	1	0	0
2020_2021	23/12/2020	4	atire	1	0	0
2020_2021	23/12/2020	4	atire	1	0	0
2020_2021	23/12/2020	4	atire	0	2	0
2020_2021	23/12/2020	4	atire	1	0	0
2020_2021	23/12/2020	4	atire	1	0	0
2020_2021	23/12/2020	4	atire	1	0	0
2020_2021	23/12/2020	4	atire	1	1	0
2020_2021	23/12/2020	4	atire	1	0	0
2020_2021	23/12/2020	4	atire	0	0	1
2020_2021	23/12/2020	4	atire	1	0	0
2020_2021	04/01/2021	5	uatio	1	0	0
2020_2021	04/01/2021	5	uatio	0	0	1
2020_2021	04/01/2021	5	uatio	0	1	0
2020_2021	04/01/2021	5	uatio	0	2	0
2020_2021	04/01/2021	5	uatio	0	1	0
2020_2021	04/01/2021	5	uatio	0	1	0
2020_2021	04/01/2021	5	uatio	0	0	1
2020_2021	04/01/2021	5	uatio	0	1	0
2020_2021	04/01/2021	5	ua	0	0	0
2020_2021	04/01/2021	5	uaterembi	1	0	0
2020_2021	04/01/2021	5	uaterembi	0	3	0
2020_2021	04/01/2021	5	uaterembi	0	1	0
2020_2021	04/01/2021	5	uaterembi	0	0	1
2020_2021	04/01/2021	5	uaterembi	0	3	0
2020_2021	04/01/2021	5	uaterembi	1	0	0
2020_2021	04/01/2021	5	uaterembi	1	0	0
2020_2021	04/01/2021	5	uaterembi	0	0	1
2020_2021	04/01/2021	5	uaterembi	0	0	1
2020_2021	04/01/2021	5	gi	0	3	0
2020_2021	04/01/2021	5	gi	1	0	0
2020_2021	04/01/2021	5	gi	1	0	0
2020_2021	04/01/2021	5	gi	0	2	0
2020_2021	04/01/2021	5	gi	0	2	0
2020_2021	04/01/2021	5	gi	1	1	0
2020_2021	04/01/2021	5	gi	1	0	0
2020_2021	04/01/2021	5	gi	0	0	1
2020_2021	04/01/2021	5	gi	0	1	0
2020_2021	04/01/2021	5	gi	0	1	0
2020_2021	04/01/2021	5	gi	0	1	0
2020_2021	04/01/2021	5	nda	1	0	0
2020_2021	04/01/2021	5	nda	0	0	1
2020_2021	04/01/2021	5	nda	1	0	0
2020_2021	04/01/2021	5	nda	0	0	1

2020_2021	04/01/2021	5	nda	0	1	0
2020_2021	04/01/2021	5	nda	0	1	0
2020_2021	04/01/2021	5	tere	0	0	0
2020_2021	04/01/2021	5	kouare	0	0	1
2020_2021	04/01/2021	5	kouare	1	0	0
2020_2021	04/01/2021	5	kouare	0	1	0
2020_2021	04/01/2021	5	kouare	1	1	0
2020_2021	04/01/2021	5	kouare	1	0	0
2020_2021	04/01/2021	5	kouare	1	0	0
2020_2021	04/01/2021	5	kouare	1	0	0
2020_2021	04/01/2021	5	kouare	1	0	0
2020_2021	04/01/2021	5	kouare	0	1	0
2020_2021	05/01/2021	5	nge	0	4	0
2020_2021	05/01/2021	5	nge	0	1	0
2020_2021	05/01/2021	5	nge	0	1	0
2020_2021	05/01/2021	5	nge	0	1	0
2020_2021	05/01/2021	5	nge	1	3	0
2020_2021	05/01/2021	5	nge	0	1	0
2020_2021	05/01/2021	5	ieroue	0	1	0
2020_2021	05/01/2021	5	ieroue	1	3	0
2020_2021	05/01/2021	5	ieroue	1	0	0
2020_2021	05/01/2021	5	vua	1	2	0
2020_2021	05/01/2021	5	vua	1	2	0
2020_2021	05/01/2021	5	vua	0	0	1
2020_2021	05/01/2021	5	vua	0	0	1
2020_2021	05/01/2021	5	vua	1	2	0
2020_2021	05/01/2021	5	vua	1	0	0
2020_2021	05/01/2021	5	vua	0	1	0
2020_2021	05/01/2021	5	vua	0	2	0
2020_2021	05/01/2021	5	vua	1	0	0
2020_2021	05/01/2021	5	vua	1	0	0
2020_2021	05/01/2021	5	vua	0	2	0
2020_2021	05/01/2021	5	vua	0	2	0
2020_2021	05/01/2021	5	vua	0	1	0
2020_2021	05/01/2021	5	redika	0	1	0
2020_2021	05/01/2021	5	redika	0	0	1
2020_2021	05/01/2021	5	redika	0	1	0
2020_2021	05/01/2021	5	redika	0	1	0
2020_2021	05/01/2021	5	atire	1	1	0
2020_2021	05/01/2021	5	atire	0	0	1
2020_2021	05/01/2021	5	atire	1	0	0
2020_2021	05/01/2021	5	atire	0	1	0
2020_2021	05/01/2021	5	atire	0	4	0
2020_2021	05/01/2021	5	atire	0	1	0
2020_2021	05/01/2021	5	atire	0	5	0
2020_2021	05/01/2021	5	atire	1	0	0
2020_2021	05/01/2021	5	atire	0	1	0
2020_2021	05/01/2021	5	atire	0	1	0
2020_2021	05/01/2021	5	atire	1	0	0
2020_2021	05/01/2021	5	atire	1	1	0

2020_2021	05/01/2021	5	atire	0	0	1
2020_2021	05/01/2021	5	atire	0	0	1
2020_2021	05/01/2021	5	atire	1	0	0
2020_2021	07/01/2021	6	uo	0	0	0
2020_2021	07/01/2021	6	mato	1	1	0
2020_2021	07/01/2021	6	mato	0	1	0
2020_2021	07/01/2021	6	mato	0	2	0
2020_2021	07/01/2021	6	mato	0	1	0
2020_2021	07/01/2021	6	mato	0	1	0
2020_2021	07/01/2021	6	uie	0	1	0
2020_2021	07/01/2021	6	uie	1	0	0
2020_2021	07/01/2021	6	uie	1	3	0
2020_2021	07/01/2021	6	uie	0	0	1
2020_2021	07/01/2021	6	ndo	0	1	0
2020_2021	07/01/2021	6	ndo	0	0	1
2020_2021	07/01/2021	6	ndo	1	0	0
2020_2021	07/01/2021	6	ndo	0	0	1
2020_2021	07/01/2021	6	ndo	1	0	0
2020_2021	07/01/2021	6	totea	1	1	0
2020_2021	07/01/2021	6	totea	0	2	0
2020_2021	07/01/2021	6	totea	1	1	0
2020_2021	07/01/2021	6	mbore	0	0	0
2020_2021	07/01/2021	6	petit koko	0	0	0
2020_2021	19/01/2021	7	nge	1	0	0
2020_2021	19/01/2021	7	nge	0	1	0
2020_2021	19/01/2021	7	nge	0	1	0
2020_2021	08/01/2021	6	noe	0	0	0
2020_2021	08/01/2021	6	puemba	1	0	0
2020_2021	08/01/2021	6	puemba	1	0	0
2020_2021	08/01/2021	6	pumbo	1	0	0
2020_2021	08/01/2021	6	pumbo	0	1	0
2020_2021	08/01/2021	6	ugo	0	0	0
2020_2021	19/01/2021	7	redika	0	2	0
2020_2021	19/01/2021	7	vua	1	0	0
2020_2021	19/01/2021	7	vua	1	0	0
2020_2021	19/01/2021	7	vua	1	0	0
2020_2021	19/01/2021	7	vua	0	1	0
2020_2021	19/01/2021	7	vua	0	0	1
2020_2021	19/01/2021	7	vua	1	0	0
2020_2021	19/01/2021	7	vua	0	1	0
2020_2021	19/01/2021	7	vua	0	1	0
2020_2021	19/01/2021	7	vua	0	1	0
2020_2021	19/01/2021	7	vua	0	0	1
2020_2021	19/01/2021	7	vua	1	0	0
2020_2021	19/01/2021	7	vua	1	0	0
2020_2021	19/01/2021	7	vua	0	1	0
2020_2021	19/01/2021	7	vua	0	0	1
2020_2021	19/01/2021	7	ieroue	1	0	0
2020_2021	19/01/2021	7	uaterembi	1	0	0
2020_2021	19/01/2021	7	uaterembi	1	0	0

2020_2021	19/01/2021	7	uaterembi	0	1	0
2020_2021	19/01/2021	7	uaterembi	1	0	0
2020_2021	19/01/2021	7	uaterembi	0	1	0
2020_2021	19/01/2021	7	uaterembi	0	1	0
2020_2021	19/01/2021	7	uaterembi	0	0	1
2020_2021	19/01/2021	7	uaterembi	1	0	0
2020_2021	19/01/2021	7	uaterembi	0	2	0
2020_2021	19/01/2021	7	uatio	0	0	1
2020_2021	19/01/2021	7	uatio	0	1	0
2020_2021	19/01/2021	7	uatio	0	1	0
2020_2021	19/01/2021	7	uatio	0	0	1
2020_2021	19/01/2021	7	ua	0	0	0
2020_2021	19/01/2021	7	nge	1	1	0
2020_2021	19/01/2021	7	nge	1	0	0
2020_2021	19/01/2021	7	nge	0	1	0
2020_2021	19/01/2021	7	nge	0	0	1
2020_2021	19/01/2021	7	nge	1	0	0
2020_2021	19/01/2021	7	nge	1	0	0
2020_2021	20/01/2021	7	tere	0	0	0
2020_2021	20/01/2021	7	nda	1	0	0
2020_2021	20/01/2021	7	nda	0	1	0
2020_2021	20/01/2021	7	nda	1	0	0
2020_2021	20/01/2021	7	nda	1	0	0
2020_2021	20/01/2021	7	nda	1	0	0
2020_2021	20/01/2021	7	kouare	1	0	0
2020_2021	20/01/2021	7	kouare	1	0	0
2020_2021	20/01/2021	7	kouare	1	0	0
2020_2021	20/01/2021	7	kouare	1	0	0
2020_2021	20/01/2021	7	kouare	1	0	0
2020_2021	20/01/2021	7	kouare	0	1	0
2020_2021	20/01/2021	7	kouare	1	0	0
2020_2021	20/01/2021	7	kouare	1	0	0
2020_2021	20/01/2021	7	atire	1	1	0
2020_2021	20/01/2021	7	atire	0	2	0
2020_2021	20/01/2021	7	atire	1	0	0
2020_2021	20/01/2021	7	atire	1	1	0
2020_2021	20/01/2021	7	atire	1	2	0
2020_2021	20/01/2021	7	atire	1	0	0
2020_2021	20/01/2021	7	atire	1	0	0
2020_2021	20/01/2021	7	atire	1	0	0
2020_2021	20/01/2021	7	atire	1	1	0
2020_2021	20/01/2021	7	atire	0	1	0
2020_2021	20/01/2021	7	atire	0	1	0
2020_2021	20/01/2021	7	atire	1	1	0
2020_2021	20/01/2021	7	atire	1	0	0
2020_2021	20/01/2021	7	atire	1	0	0
2020_2021	20/01/2021	7	atire	0	1	0
2020_2021	20/01/2021	7	atire	0	1	0
2020_2021	20/01/2021	7	atire	1	0	0
2020_2021	06/02/2021	8	redika	0	0	0

2020_2021	06/02/2021	8	ieroue	0	1	0
2020_2021	06/02/2021	8	uaterembi	0	1	0
2020_2021	06/02/2021	8	uaterembi	1	0	0
2020_2021	06/02/2021	8	uaterembi	0	0	1
2020_2021	06/02/2021	8	uaterembi	1	0	0
2020_2021	06/02/2021	8	ua	0	0	0
2020_2021	06/02/2021	8	nge	0	0	1
2020_2021	06/02/2021	8	nge	0	0	1
2020_2021	06/02/2021	8	gi	1	0	0
2020_2021	06/02/2021	8	tere	0	0	0
2020_2021	06/02/2021	8	nda	0	1	0
2020_2021	06/02/2021	8	nda	0	1	0
2020_2021	06/02/2021	8	nda	1	0	0
2020_2021	07/02/2021	8	kouare	1	1	0
2020_2021	07/02/2021	8	kouare	1	0	0
2020_2021	07/02/2021	8	vua	1	0	0
2020_2021	07/02/2021	8	vua	0	0	1
2020_2021	07/02/2021	8	atire	0	1	0
2020_2021	07/02/2021	8	atire	1	0	0
2020_2021	07/02/2021	8	atire	1	0	0
2020_2021	07/02/2021	8	atire	1	0	0
2020_2021	07/02/2021	8	atire	1	0	0
2020_2021	07/02/2021	8	atire	0	1	0
2020_2021	07/02/2021	8	atire	1	0	0
2020_2021	07/02/2021	8	atire	1	1	0
2020_2021	07/02/2021	8	atire	1	0	0
2020_2021	07/02/2021	8	atire	0	1	0
2021_2022	17/11/2021	1	atire	1	0	0
2021_2022	17/11/2021	1	atire	0	2	0
2021_2022	17/11/2021	1	vua	0	1	0
2021_2022	17/11/2021	1	vua	1	2	0
2021_2022	17/11/2021	1	vua	0	0	1
2021_2022	17/11/2021	1	ieroue	0	0	0
2021_2022	17/11/2021	1	kouare	0	0	0
2021_2022	17/11/2021	1	nda	0	0	0
2021_2022	17/11/2021	1	tere	0	0	0
2021_2022	17/11/2021	1	nge	0	0	1
2021_2022	17/11/2021	1	nge	1	0	0
2021_2022	17/11/2021	1	nge	1	0	0
2021_2022	17/11/2021	1	nge	0	0	1
2021_2022	17/11/2021	1	nge	0	0	1
2021_2022	18/11/2021	1	gi	1	0	0
2021_2022	18/11/2021	1	gi	1	0	0
2021_2022	18/11/2021	1	gi	0	0	1
2021_2022	18/11/2021	1	gi	1	0	0
2021_2022	18/11/2021	1	gi	1	0	0
2021_2022	18/11/2021	1	gi	0	1	0
2021_2022	18/11/2021	1	uaterembi	0	0	0
2021_2022	18/11/2021	1	uatio	0	0	0
2021_2022	18/11/2021	1	ua	0	0	0

2021_2022	18/11/2021	1	redika	0	0	0
2021_2022	07/12/2021	2	vua	1	0	0
2021_2022	07/12/2021	2	vua	0	0	1
2021_2022	07/12/2021	2	vua	1	0	0
2021_2022	07/12/2021	2	vua	1	0	0
2021_2022	07/12/2021	2	vua	0	2	0
2021_2022	07/12/2021	2	vua	0	0	1
2021_2022	07/12/2021	2	vua	1	0	0
2021_2022	07/12/2021	2	vua	1	0	0
2021_2022	07/12/2021	2	vua	0	0	1
2021_2022	07/12/2021	2	vua	1	0	0
2021_2022	07/12/2021	2	uaterembi	0	1	0
2021_2022	07/12/2021	2	uaterembi	1	1	0
2021_2022	07/12/2021	2	uaterembi	0	0	1
2021_2022	07/12/2021	2	uaterembi	1	0	0
2021_2022	07/12/2021	2	uaterembi	1	0	0
2021_2022	07/12/2021	2	uaterembi	1	0	0
2021_2022	07/12/2021	2	uaterembi	1	0	0
2021_2022	07/12/2021	2	uaterembi	0	1	0
2021_2022	07/12/2021	2	uaterembi	0	1	0
2021_2022	07/12/2021	2	uaterembi	0	4	0
2021_2022	07/12/2021	2	uaterembi	0	4	0
2021_2022	07/12/2021	2	uaterembi	0	0	1
2021_2022	07/12/2021	2	uaterembi	1	0	0
2021_2022	07/12/2021	2	uaterembi	1	1	0
2021_2022	07/12/2021	2	uaterembi	0	1	0
2021_2022	07/12/2021	2	uaterembi	0	2	0
2021_2022	07/12/2021	2	gi	0	0	1
2021_2022	07/12/2021	2	gi	0	1	0
2021_2022	07/12/2021	2	gi	0	1	0
2021_2022	07/12/2021	2	gi	1	0	0
2021_2022	07/12/2021	2	gi	0	1	0
2021_2022	07/12/2021	2	gi	1	0	0
2021_2022	07/12/2021	2	gi	0	0	1
2021_2022	07/12/2021	2	gi	0	0	1
2021_2022	07/12/2021	2	gi	1	0	0
2021_2022	07/12/2021	2	gi	0	1	0
2021_2022	07/12/2021	2	gi	0	1	0
2021_2022	07/12/2021	2	gi	0	3	0
2021_2022	07/12/2021	2	gi	0	1	0
2021_2022	07/12/2021	2	gi	1	2	0
2021_2022	07/12/2021	2	gi	1	1	0
2021_2022	07/12/2021	2	gi	1	0	0
2021_2022	07/12/2021	2	gi	1	0	0
2021_2022	07/12/2021	2	kouare	1	2	0
2021_2022	07/12/2021	2	kouare	0	0	1
2021_2022	07/12/2021	2	kouare	0	1	0
2021_2022	07/12/2021	2	nda	1	0	0
2021_2022	07/12/2021	2	nda	1	0	0
2021_2022	07/12/2021	2	nda	0	1	0

2021_2022	07/12/2021	2	nda	1	0	0
2021_2022	07/12/2021	2	nda	0	2	0
2021_2022	07/12/2021	2	nda	0	1	0
2021_2022	07/12/2021	2	tere	0	0	0
2021_2022	07/12/2021	2	nge	1	0	0
2021_2022	07/12/2021	2	nge	1	0	0
2021_2022	07/12/2021	2	nge	1	0	0
2021_2022	07/12/2021	2	nge	1	0	0
2021_2022	07/12/2021	2	nge	1	0	0
2021_2022	07/12/2021	2	nge	1	0	0
2021_2022	07/12/2021	2	nge	1	0	0
2021_2022	07/12/2021	2	nge	0	0	1
2021_2022	07/12/2021	2	nge	0	1	0
2021_2022	07/12/2021	2	nge	1	0	0
2021_2022	07/12/2021	2	nge	0	0	1
2021_2022	07/12/2021	2	nge	0	1	0
2021_2022	07/12/2021	2	nge	1	0	0
2021_2022	07/12/2021	2	nge	0	1	0
2021_2022	07/12/2021	2	nge	1	0	0
2021_2022	07/12/2021	2	nge	0	0	1
2021_2022	07/12/2021	2	nge	0	1	0
2021_2022	07/12/2021	2	nge	1	0	0
2021_2022	07/12/2021	2	nge	1	0	0
2021_2022	08/12/2021	2	ua	0	0	0
2021_2022	08/12/2021	2	uatio	1	0	0
2021_2022	08/12/2021	2	uatio	1	0	0
2021_2022	08/12/2021	2	uatio	1	0	0
2021_2022	08/12/2021	2	ieroue	1	0	0
2021_2022	08/12/2021	2	atire	0	0	1
2021_2022	08/12/2021	2	atire	1	0	0
2021_2022	08/12/2021	2	atire	1	0	0
2021_2022	08/12/2021	2	atire	0	0	1
2021_2022	08/12/2021	2	atire	0	0	1
2021_2022	08/12/2021	2	atire	1	0	0
2021_2022	08/12/2021	2	atire	1	0	0
2021_2022	08/12/2021	2	atire	1	0	0
2021_2022	08/12/2021	2	atire	1	0	0
2021_2022	08/12/2021	2	atire	0	0	1
2021_2022	08/12/2021	2	redika	1	0	0
2021_2022	08/12/2021	2	redika	1	0	0
2021_2022	08/12/2021	2	redika	1	2	0
2021_2022	08/12/2021	2	redika	1	0	0
2021_2022	16/12/2021	3	nouare	0	1	0
2021_2022	16/12/2021	3	nouare	1	0	0
2021_2022	16/12/2021	3	nouare	0	0	1
2021_2022	16/12/2021	3	ugo	0	0	0
2021_2022	16/12/2021	3	pumbo	0	0	0
2021_2022	16/12/2021	3	puemba	1	1	0
2021_2022	16/12/2021	3	noe	0	0	0
2021_2022	16/12/2021	3	mato	0	5	0

2021_2022	16/12/2021	3	mato	1	0	0
2021_2022	16/12/2021	3	mato	1	1	0
2021_2022	16/12/2021	3	mato	0	1	0
2021_2022	17/12/2021	3	uie	1	0	0
2021_2022	17/12/2021	3	uie	0	1	0
2021_2022	17/12/2021	3	uie	1	0	0
2021_2022	17/12/2021	3	uie	1	0	0
2021_2022	17/12/2021	3	uie	1	0	0
2021_2022	17/12/2021	3	uie	0	3	0
2021_2022	17/12/2021	3	uie	0	2	0
2021_2022	17/12/2021	3	uie	1	3	0
2021_2022	17/12/2021	3	uie	1	0	0
2021_2022	17/12/2021	3	uie	0	1	0
2021_2022	17/12/2021	3	uie	0	0	1
2021_2022	17/12/2021	3	ndo	1	0	0
2021_2022	17/12/2021	3	ndo	1	0	0
2021_2022	17/12/2021	3	totea	1	0	0
2021_2022	17/12/2021	3	totea	1	0	0
2021_2022	17/12/2021	3	totea	1	2	0
2021_2022	17/12/2021	3	totea	1	0	0
2021_2022	17/12/2021	3	totea	1	2	0
2021_2022	17/12/2021	3	totea	0	1	0
2021_2022	17/12/2021	3	totea	0	2	0
2021_2022	17/12/2021	3	totea	0	1	0
2021_2022	17/12/2021	3	mbore	1	0	0
2021_2022	17/12/2021	3	petit koko	0	1	0
2021_2022	17/12/2021	3	uo	1	0	0
2021_2022	21/12/2021	4	atire	0	1	0
2021_2022	21/12/2021	4	atire	1	0	0
2021_2022	21/12/2021	4	atire	0	2	0
2021_2022	21/12/2021	4	atire	1	1	0
2021_2022	21/12/2021	4	atire	1	0	0
2021_2022	21/12/2021	4	vua	0	2	0
2021_2022	21/12/2021	4	vua	0	0	1
2021_2022	21/12/2021	4	vua	0	1	0
2021_2022	21/12/2021	4	vua	0	0	1
2021_2022	21/12/2021	4	vua	0	0	1
2021_2022	21/12/2021	4	vua	0	1	0
2021_2022	21/12/2021	4	vua	0	1	0
2021_2022	21/12/2021	4	vua	0	0	1
2021_2022	21/12/2021	4	ua	0	0	1
2021_2022	21/12/2021	4	ua	1	0	0
2021_2022	21/12/2021	4	uaterembi	1	0	0
2021_2022	21/12/2021	4	uaterembi	0	1	0
2021_2022	21/12/2021	4	uaterembi	1	1	0
2021_2022	21/12/2021	4	uaterembi	1	1	0
2021_2022	21/12/2021	4	uaterembi	0	3	0
2021_2022	21/12/2021	4	uaterembi	1	0	0
2021_2022	21/12/2021	4	uaterembi	1	0	0
2021_2022	21/12/2021	4	uaterembi	0	1	0

2021_2022	21/12/2021	4	uaterembi	0	0	0
2021_2022	21/12/2021	4	uaterembi	0	0	0
2021_2022	21/12/2021	4	kouare	0	1	0
2021_2022	21/12/2021	4	kouare	1	0	0
2021_2022	21/12/2021	4	kouare	1	0	0
2021_2022	21/12/2021	4	nda	0	0	1
2021_2022	21/12/2021	4	nda	0	0	1
2021_2022	21/12/2021	4	nda	1	1	0
2021_2022	22/12/2021	4	nge	1	0	0
2021_2022	22/12/2021	4	nge	1	0	0
2021_2022	22/12/2021	4	nge	1	1	0
2021_2022	22/12/2021	4	nge	0	0	1
2021_2022	22/12/2021	4	nge	0	0	1
2021_2022	22/12/2021	4	nge	1	0	0
2021_2022	22/12/2021	4	nge	1	0	0
2021_2022	22/12/2021	4	nge	1	0	0
2021_2022	22/12/2021	4	nge	0	0	1
2021_2022	22/12/2021	4	nge	1	0	0
2021_2022	22/12/2021	4	nge	1	0	0
2021_2022	22/12/2021	4	nge	0	3	0
2021_2022	22/12/2021	4	nge	0	0	1
2021_2022	22/12/2021	4	nge	1	0	0
2021_2022	22/12/2021	4	nge	1	0	0
2021_2022	22/12/2021	4	nge	1	0	0
2021_2022	22/12/2021	4	gi	1	0	0
2021_2022	22/12/2021	4	gi	1	1	0
2021_2022	22/12/2021	4	gi	1	0	0
2021_2022	22/12/2021	4	gi	0	0	1
2021_2022	22/12/2021	4	gi	0	0	1
2021_2022	22/12/2021	4	gi	1	0	0
2021_2022	22/12/2021	4	gi	0	1	0
2021_2022	22/12/2021	4	gi	0	1	0
2021_2022	22/12/2021	4	gi	0	0	1
2021_2022	22/12/2021	4	gi	1	1	0
2021_2022	22/12/2021	4	gi	0	1	0
2021_2022	22/12/2021	4	gi	0	2	0
2021_2022	22/12/2021	4	gi	1	2	0
2021_2022	22/12/2021	4	gi	1	2	0
2021_2022	22/12/2021	4	uatio	1	0	0
2021_2022	22/12/2021	4	uatio	1	0	0
2021_2022	22/12/2021	4	uatio	0	0	1
2021_2022	22/12/2021	4	uatio	0	0	1
2021_2022	22/12/2021	4	uatio	0	0	1
2021_2022	22/12/2021	4	uatio	0	0	1
2021_2022	22/12/2021	4	uatio	0	0	1
2021_2022	22/12/2021	4	ieroue	1	0	0
2021_2022	22/12/2021	4	redika	0	0	1
2021_2022	22/12/2021	4	redika	1	0	0
2021_2022	22/12/2021	4	redika	1	2	0
2021_2022	22/12/2021	4	redika	1	0	0

2021_2022	21/12/2021	4	tere	0	0	0
2021_2022	22/12/2021	4	gi	0	1	0
2021_2022	22/12/2021	4	gi	1	0	0
2021_2022	29/12/2021	5	nouare	0	0	0
2021_2022	29/12/2021	5	ugo	0	0	0
2021_2022	29/12/2021	5	pumbo	0	0	0
2021_2022	29/12/2021	5	puemba	1	0	0
2021_2022	29/12/2021	5	puemba	1	0	0
2021_2022	29/12/2021	5	noe	1	0	0
2021_2022	29/12/2021	5	noe	1	0	0
2021_2022	29/12/2021	5	mato	0	1	0
2021_2022	29/12/2021	5	mato	1	0	0
2021_2022	29/12/2021	5	mato	0	1	0
2021_2022	29/12/2021	5	mato	0	2	0
2021_2022	29/12/2021	5	uie	0	0	1
2021_2022	30/12/2021	5	ndo	0	1	0
2021_2022	30/12/2021	5	ndo	0	1	0
2021_2022	30/12/2021	5	ndo	0	0	1
2021_2022	30/12/2021	5	ndo	1	0	0
2021_2022	30/12/2021	5	totea	0	2	0
2021_2022	30/12/2021	5	totea	0	0	1
2021_2022	30/12/2021	5	totea	0	1	0
2021_2022	30/12/2021	5	totea	1	0	0
2021_2022	30/12/2021	5	mbore	0	0	0
2021_2022	30/12/2021	5	petit koko	1	0	0
2021_2022	30/12/2021	5	uo	1	0	0
2021_2022	30/12/2021	5	uo	1	0	0
2021_2022	30/12/2021	5	uo	1	1	0
2021_2022	04/01/2022	6	atire	1	0	0
2021_2022	04/01/2022	6	atire	1	0	0
2021_2022	04/01/2022	6	atire	1	0	0
2021_2022	04/01/2022	6	atire	0	1	0
2021_2022	04/01/2022	6	atire	1	0	0
2021_2022	04/01/2022	6	atire	0	2	0
2021_2022	04/01/2022	6	atire	1	0	0
2021_2022	04/01/2022	6	atire	0	2	0
2021_2022	04/01/2022	6	atire	1	1	0
2021_2022	04/01/2022	6	atire	0	5	0
2021_2022	04/01/2022	6	atire	1	0	0
2021_2022	04/01/2022	6	vua	1	0	0
2021_2022	04/01/2022	6	vua	1	0	0
2021_2022	04/01/2022	6	vua	0	0	1
2021_2022	04/01/2022	6	vua	1	0	0
2021_2022	04/01/2022	6	vua	1	0	0
2021_2022	04/01/2022	6	vua	0	1	0
2021_2022	04/01/2022	6	vua	0	1	0
2021_2022	04/01/2022	6	vua	0	0	1
2021_2022	04/01/2022	6	vua	0	1	0
2021_2022	04/01/2022	6	uaterembi	1	0	0
2021_2022	04/01/2022	6	uaterembi	1	0	0

2021_2022	04/01/2022	6 uaterembi	1	0	0
2021_2022	04/01/2022	6 uaterembi	0	3	0
2021_2022	04/01/2022	6 uaterembi	1	0	0
2021_2022	04/01/2022	6 uaterembi	1	1	0
2021_2022	04/01/2022	6 uaterembi	0	1	0
2021_2022	04/01/2022	6 uaterembi	0	1	0
2021_2022	04/01/2022	6 uaterembi	0	2	0
2021_2022	04/01/2022	6 uaterembi	0	0	1
2021_2022	04/01/2022	6 uaterembi	0	1	0
2021_2022	04/01/2022	6 kouare	1	0	0
2021_2022	04/01/2022	6 kouare	1	0	0
2021_2022	04/01/2022	6 kouare	0	1	0
2021_2022	04/01/2022	6 nda	0	1	0
2021_2022	04/01/2022	6 nda	1	0	0
2021_2022	04/01/2022	6 nda	1	0	0
2021_2022	04/01/2022	6 nda	1	0	0
2021_2022	04/01/2022	6 tere	0	0	0
2021_2022	04/01/2022	6 nge	1	0	0
2021_2022	04/01/2022	6 nge	1	0	0
2021_2022	04/01/2022	6 nge	0	1	0
2021_2022	04/01/2022	6 nge	1	0	0
2021_2022	04/01/2022	6 nge	1	3	0
2021_2022	04/01/2022	6 nge	0	0	0
2021_2022	04/01/2022	6 nge	1	0	0
2021_2022	04/01/2022	6 nge	1	0	0
2021_2022	04/01/2022	6 nge	1	0	0
2021_2022	04/01/2022	6 nge	0	1	0
2021_2022	04/01/2022	6 nge	1	0	0
2021_2022	04/01/2022	6 nge	1	0	0
2021_2022	05/01/2022	6 gi	1	0	0
2021_2022	05/01/2022	6 gi	1	0	0
2021_2022	05/01/2022	6 gi	0	1	0
2021_2022	05/01/2022	6 gi	0	0	0
2021_2022	05/01/2022	6 gi	0	1	0
2021_2022	05/01/2022	6 gi	0	0	0
2021_2022	05/01/2022	6 gi	0	0	0
2021_2022	05/01/2022	6 gi	0	1	0
2021_2022	05/01/2022	6 gi	1	0	0
2021_2022	05/01/2022	6 gi	0	1	0
2021_2022	05/01/2022	6 gi	1	0	0
2021_2022	05/01/2022	6 gi	1	0	0
2021_2022	05/01/2022	6 gi	0	0	0
2021_2022	05/01/2022	6 gi	0	2	0
2021_2022	05/01/2022	6 gi	1	0	0
2021_2022	05/01/2022	6 gi	1	2	0
2021_2022	05/01/2022	6 gi	1	0	0
2021_2022	05/01/2022	6 gi	1	0	0
2021_2022	05/01/2022	6 uatio	0	0	1
2021_2022	05/01/2022	6 uatio	1	2	0
2021_2022	05/01/2022	6 uatio	1	0	0

2021_2022	05/01/2022	6	uatio	0	1	0
2021_2022	05/01/2022	6	uatio	0	0	1
2021_2022	05/01/2022	6	uatio	0	0	1
2021_2022	05/01/2022	6	ua	1	0	0
2021_2022	05/01/2022	6	ieroue	1	0	0
2021_2022	05/01/2022	6	redika	0	1	0
2021_2022	05/01/2022	6	redika	0	0	0
2021_2022	05/01/2022	6	redika	1	1	0
2021_2022	05/01/2022	6	redika	0	1	0
2021_2022	05/01/2022	6	redika	1	0	0
2021_2022	05/01/2022	6	redika	1	0	0
2021_2022	26/01/2022	7	vua	1	0	0
2021_2022	26/01/2022	7	vua	1	0	0
2021_2022	26/01/2022	7	vua	1	0	0
2021_2022	26/01/2022	7	vua	0	0	1
2021_2022	26/01/2022	7	vua	1	0	0
2021_2022	26/01/2022	7	vua	1	0	0
2021_2022	26/01/2022	7	vua	0	0	1
2021_2022	26/01/2022	7	vua	0	0	0
2021_2022	26/01/2022	7	vua	0	1	0
2021_2022	26/01/2022	7	vua	0	0	1
2021_2022	26/01/2022	7	vua	0	1	0
2021_2022	26/01/2022	7	vua	0	0	0
2021_2022	26/01/2022	7	vua	1	0	0
2021_2022	26/01/2022	7	vua	1	2	0
2021_2022	26/01/2022	7	vua	0	1	0
2021_2022	26/01/2022	7	vua	0	1	0
2021_2022	26/01/2022	7	vua	1	0	0
2021_2022	26/01/2022	7	vua	1	0	0
2021_2022	26/01/2022	7	ieroue	1	0	0
2021_2022	26/01/2022	7	ieroue	1	0	0
2021_2022	26/01/2022	7	uaterembi	1	0	0
2021_2022	26/01/2022	7	uaterembi	0	1	0
2021_2022	26/01/2022	7	uaterembi	1	0	0
2021_2022	26/01/2022	7	uaterembi	0	2	0
2021_2022	26/01/2022	7	uaterembi	0	0	0
2021_2022	26/01/2022	7	uaterembi	0	1	0
2021_2022	26/01/2022	7	uaterembi	1	0	0
2021_2022	26/01/2022	7	uaterembi	0	0	0
2021_2022	26/01/2022	7	uaterembi	0	1	0
2021_2022	26/01/2022	7	uaterembi	1	0	0
2021_2022	26/01/2022	7	uaterembi	1	0	0
2021_2022	26/01/2022	7	uatio	1	0	0
2021_2022	26/01/2022	7	uatio	0	2	0
2021_2022	26/01/2022	7	ua	0	0	0
2021_2022	26/01/2022	7	gi	1	0	0
2021_2022	26/01/2022	7	gi	0	2	0
2021_2022	26/01/2022	7	gi	1	0	0
2021_2022	26/01/2022	7	gi	1	0	0
2021_2022	26/01/2022	7	gi	1	0	0

2021_2022	26/01/2022	7	gi	1	0	0
2021_2022	26/01/2022	7	gi	1	0	0
2021_2022	26/01/2022	7	gi	1	0	0
2021_2022	26/01/2022	7	gi	1	0	0
2021_2022	26/01/2022	7	gi	1	1	0
2021_2022	26/01/2022	7	gi	1	2	0
2021_2022	26/01/2022	7	gi	1	1	0
2021_2022	26/01/2022	7	gi	1	0	0
2021_2022	26/01/2022	7	gi	0	2	0
2021_2022	26/01/2022	7	gi	0	1	0
2021_2022	26/01/2022	7	gi	0	1	0
2021_2022	26/01/2022	7	gi	0	2	0
2021_2022	26/01/2022	7	gi	0	1	0
2021_2022	26/01/2022	7	gi	0	1	0
2021_2022	26/01/2022	7	gi	1	0	0
2021_2022	26/01/2022	7	gi	0	2	0
2021_2022	27/01/2022	7	nge	0	1	0
2021_2022	27/01/2022	7	nge	1	0	0
2021_2022	27/01/2022	7	nge	1	0	0
2021_2022	27/01/2022	7	nge	0	1	0
2021_2022	27/01/2022	7	nge	1	0	0
2021_2022	27/01/2022	7	nge	1	0	0
2021_2022	27/01/2022	7	nge	1	0	0
2021_2022	27/01/2022	7	nge	1	0	0
2021_2022	27/01/2022	7	nge	1	0	0
2021_2022	27/01/2022	7	nge	1	0	0
2021_2022	27/01/2022	7	kouare	1	0	0
2021_2022	27/01/2022	7	kouare	0	0	0
2021_2022	27/01/2022	7	kouare	0	0	1
2021_2022	27/01/2022	7	kouare	1	0	0
2021_2022	27/01/2022	7	kouare	0	0	1
2021_2022	27/01/2022	7	kouare	0	0	1
2021_2022	27/01/2022	7	kouare	1	0	0
2021_2022	27/01/2022	7	kouare	0	1	0
2021_2022	27/01/2022	7	tere	0	0	0
2021_2022	27/01/2022	7	nda	1	1	0
2021_2022	27/01/2022	7	nda	1	0	0
2021_2022	27/01/2022	7	nda	0	1	0
2021_2022	27/01/2022	7	nda	1	0	0
2021_2022	27/01/2022	7	nda	1	0	0
2021_2022	27/01/2022	7	nda	0	1	0
2021_2022	27/01/2022	7	redika	1	0	0
2021_2022	27/01/2022	7	redika	0	0	1
2021_2022	27/01/2022	7	redika	1	0	0
2021_2022	27/01/2022	7	atire	0	2	0
2021_2022	27/01/2022	7	atire	0	1	0
2021_2022	27/01/2022	7	atire	0	0	1
2021_2022	27/01/2022	7	atire	0	0	0
2021_2022	27/01/2022	7	atire	1	1	0
2021_2022	27/01/2022	7	atire	0	1	0

2021_2022	27/01/2022	7	atire	0	3	0
2021_2022	27/01/2022	7	atire	1	0	0
2021_2022	27/01/2022	7	atire	1	0	0
2021_2022	27/01/2022	7	atire	1	0	0
2021_2022	27/01/2022	7	atire	1	0	0
2021_2022	27/01/2022	7	atire	1	0	0
2021_2022	27/01/2022	7	atire	1	0	0
2021_2022	27/01/2022	7	atire	1	0	0
2021_2022	27/01/2022	7	atire	1	0	0
2021_2022	27/01/2022	7	atire	1	3	0
2021_2022	14/02/2022	8	atire	0	1	0
2021_2022	14/02/2022	8	atire	1	0	0
2021_2022	14/02/2022	8	atire	1	0	0
2021_2022	14/02/2022	8	atire	0	1	0
2021_2022	14/02/2022	8	atire	1	0	0
2021_2022	14/02/2022	8	atire	0	1	0
2021_2022	14/02/2022	8	atire	1	0	0
2021_2022	14/02/2022	8	atire	0	4	0
2021_2022	14/02/2022	8	vua	0	1	0
2021_2022	14/02/2022	8	ieroue	0	0	0
2021_2022	14/02/2022	8	kouare	0	0	0
2021_2022	14/02/2022	8	nda	0	0	0
2021_2022	14/02/2022	8	tere	0	0	0
2021_2022	15/02/2022	8	gi	0	0	0
2021_2022	15/02/2022	8	gi	1	0	0
2021_2022	15/02/2022	8	gi	1	0	0
2021_2022	15/02/2022	8	uaterembi	0	0	0
2021_2022	15/02/2022	8	uatio	0	0	0
2021_2022	15/02/2022	8	ua	1	0	0
2021_2022	16/02/2022	8	nge	1	0	0
2021_2022	16/02/2022	8	nge	1	0	0
2021_2022	16/02/2022	8	nge	1	0	0
2021_2022	16/02/2022	8	nge	0	1	0
2021_2022	16/02/2022	8	nge	1	0	0
2021_2022	16/02/2022	8	nge	1	0	0
2021_2022	16/02/2022	8	nge	0	0	0
2021_2022	16/02/2022	8	nge	0	1	0
2021_2022	16/02/2022	8	nge	1	0	0
2021_2022	18/02/2022	8	redika	0	0	0
2021_2022	23/02/2022	10	gi	1	0	0
2021_2022	23/02/2022	10	gi	1	0	0
2021_2022	23/02/2022	10	nge	0	0	0
2021_2022	10/03/2022	11	ieroue	0	0	0
2021_2022	10/03/2022	11	uaterembi	0	0	0
2021_2022	10/03/2022	11	uatio	0	0	0
2021_2022	10/03/2022	11	ua	0	0	0
2021_2022	10/03/2022	11	nge	1	0	0
2021_2022	10/03/2022	11	nge	0	0	0
2021_2022	10/03/2022	11	nge	0	0	0
2021_2022	10/03/2022	11	gi	1	0	0

2021_2022	10/03/2022	11	gi	1	0	0
2021_2022	10/03/2022	11	gi	0	0	0
2021_2022	10/03/2022	11	gi	0	0	0
2021_2022	10/03/2022	11	gi	0	0	0
2021_2022	11/03/2022	11	nda	0	0	0
2021_2022	11/03/2022	11	kouare	0	0	0
2021_2022	11/03/2022	11	tere	0	0	0
2021_2022	11/03/2022	11	vua	1	0	0
2021_2022	11/03/2022	11	redika	0	0	0
2021_2022	11/03/2022	11	atire	0	0	0
2022_2023	22/11/2022	1	atire	0	0	1
2022_2023	22/11/2022	1	atire	1	0	0
2022_2023	22/11/2022	1	atire	0	0	1
2022_2023	22/11/2022	1	atire	0	2	0
2022_2023	22/11/2022	1	redika	0	0	0
2022_2023	22/11/2022	1	vua	1	0	0
2022_2023	22/11/2022	1	vua	1	0	0
2022_2023	22/11/2022	1	tere	0	0	0
2022_2023	22/11/2022	1	nda	0	0	1
2022_2023	22/11/2022	1	nda	0	0	1
2022_2023	22/11/2022	1	nda	1	0	0
2022_2023	22/11/2022	1	nda	0	1	0
2022_2023	22/11/2022	1	nda	0	1	0
2022_2023	22/11/2022	1	nda	1	0	0
2022_2023	22/11/2022	1	kouare	1	0	0
2022_2023	22/11/2022	1	gi	1	0	0
2022_2023	22/11/2022	1	gi	1	0	0
2022_2023	22/11/2022	1	gi	0	3	0
2022_2023	23/11/2022	1	uaterembi	0	1	0
2022_2023	23/11/2022	1	uaterembi	1	0	0
2022_2023	23/11/2022	1	uaterembi	0	0	1
2022_2023	23/11/2022	1	uatio	1	0	0
2022_2023	23/11/2022	1	uatio	0	2	0
2022_2023	23/11/2022	1	uatio	1	0	0
2022_2023	23/11/2022	1	uatio	0	2	0
2022_2023	23/11/2022	1	uatio	1	0	0
2022_2023	23/11/2022	1	uatio	1	3	0
2022_2023	23/11/2022	1	uatio	0	0	1
2022_2023	23/11/2022	1	uatio	0	0	1
2022_2023	23/11/2022	1	ua	1	0	0
2022_2023	23/11/2022	1	nge	1	1	0
2022_2023	23/11/2022	1	nge	1	0	0
2022_2023	23/11/2022	1	ieroue	1	0	0
2022_2023	23/11/2022	1	ieroue	0	0	1
2022_2023	23/11/2022	1	ieroue	1	0	0
2022_2023	23/11/2022	1	ieroue	1	0	0
2022_2023	05/12/2022	2	kie	0	0	1
2022_2023	05/12/2022	2	kie	1	0	0
2022_2023	05/12/2022	2	kie	1	2	0
2022_2023	05/12/2022	2	kie	0	1	0

2022_2023	05/12/2022	2	kie	1	0	0
2022_2023	05/12/2022	2	kie	1	0	0
2022_2023	05/12/2022	2	kie	0	1	0
2022_2023	05/12/2022	2	amere	1	0	0
2022_2023	05/12/2022	2	amere	0	1	0
2022_2023	05/12/2022	2	amere	1	0	0
2022_2023	05/12/2022	2	amere	1	1	0
2022_2023	05/12/2022	2	amere	1	0	0
2022_2023	05/12/2022	2	amere	1	0	0
2022_2023	05/12/2022	2	nouare	0	0	0
2022_2023	07/12/2022	3	vua	1	0	0
2022_2023	07/12/2022	3	vua	1	0	0
2022_2023	07/12/2022	3	vua	1	1	0
2022_2023	07/12/2022	3	vua	0	0	1
2022_2023	07/12/2022	3	vua	0	1	0
2022_2023	07/12/2022	3	vua	1	0	0
2022_2023	07/12/2022	3	vua	1	0	0
2022_2023	07/12/2022	3	vua	1	1	0
2022_2023	07/12/2022	3	vua	0	0	1
2022_2023	07/12/2022	3	uatio	0	1	0
2022_2023	07/12/2022	3	uatio	0	1	0
2022_2023	07/12/2022	3	uatio	0	1	0
2022_2023	07/12/2022	3	uatio	0	1	0
2022_2023	07/12/2022	3	uatio	0	0	1
2022_2023	07/12/2022	3	uatio	0	3	0
2022_2023	07/12/2022	3	uatio	1	0	0
2022_2023	07/12/2022	3	uatio	0	3	0
2022_2023	07/12/2022	3	kouare	0	0	1
2022_2023	07/12/2022	3	kouare	1	0	0
2022_2023	07/12/2022	3	kouare	1	1	0
2022_2023	07/12/2022	3	kouare	1	0	0
2022_2023	07/12/2022	3	kouare	1	0	0
2022_2023	07/12/2022	3	kouare	1	0	0
2022_2023	07/12/2022	3	kouare	0	1	0
2022_2023	07/12/2022	3	kouare	1	0	0
2022_2023	07/12/2022	3	kouare	0	1	0
2022_2023	07/12/2022	3	nda	0	6	0
2022_2023	07/12/2022	3	nda	0	1	0
2022_2023	07/12/2022	3	nda	1	1	0
2022_2023	07/12/2022	3	nda	0	0	1
2022_2023	07/12/2022	3	nda	0	0	1
2022_2023	07/12/2022	3	nda	0	0	1
2022_2023	07/12/2022	3	nda	0	1	0
2022_2023	07/12/2022	3	nda	0	2	0
2022_2023	07/12/2022	3	nda	0	2	0
2022_2023	07/12/2022	3	nda	0	1	0
2022_2023	07/12/2022	3	nda	1	0	0
2022_2023	07/12/2022	3	nda	0	1	0
2022_2023	07/12/2022	3	nda	0	0	1
2022_2023	07/12/2022	3	nda	1	0	0

2022_2023	07/12/2022	3	nda	1	0	0
2022_2023	07/12/2022	3	nda	0	2	0
2022_2023	07/12/2022	3	nda	0	2	0
2022_2023	07/12/2022	3	nda	0	0	1
2022_2023	08/12/2022	3	nge	1	0	0
2022_2023	08/12/2022	3	nge	0	1	0
2022_2023	08/12/2022	3	nge	1	0	0
2022_2023	08/12/2022	3	nge	0	1	0
2022_2023	08/12/2022	3	nge	1	0	0
2022_2023	08/12/2022	3	nge	0	2	0
2022_2023	08/12/2022	3	nge	1	0	0
2022_2023	08/12/2022	3	nge	0	2	0
2022_2023	08/12/2022	3	gi	0	1	0
2022_2023	08/12/2022	3	gi	1	0	0
2022_2023	08/12/2022	3	ua	0	0	1
2022_2023	08/12/2022	3	ua	1	1	0
2022_2023	08/12/2022	3	ua	0	1	0
2022_2023	08/12/2022	3	ua	1	1	0
2022_2023	08/12/2022	3	ua	0	1	0
2022_2023	08/12/2022	3	ua	0	5	0
2022_2023	08/12/2022	3	ua	1	0	0
2022_2023	08/12/2022	3	ua	0	4	0
2022_2023	08/12/2022	3	ua	0	2	0
2022_2023	08/12/2022	3	ieroue	1	0	0
2022_2023	08/12/2022	3	ieroue	0	1	0
2022_2023	08/12/2022	3	ieroue	1	0	0
2022_2023	08/12/2022	3	ieroue	1	1	0
2022_2023	08/12/2022	3	ieroue	0	1	0
2022_2023	08/12/2022	3	ieroue	0	1	0
2022_2023	08/12/2022	3	ieroue	1	0	0
2022_2023	08/12/2022	3	ieroue	1	0	0
2022_2023	14/12/2022	4	uo	0	1	0
2022_2023	14/12/2022	4	ndo	1	0	0
2022_2023	14/12/2022	4	ndo	0	0	1
2022_2023	14/12/2022	4	ndo	0	1	0
2022_2023	14/12/2022	4	ndo	1	0	0
2022_2023	14/12/2022	4	ndo	1	3	0
2022_2023	14/12/2022	4	ndo	1	0	0
2022_2023	14/12/2022	4	ndo	0	1	0
2022_2023	14/12/2022	4	totea	1	0	0
2022_2023	14/12/2022	4	totea	0	2	0
2022_2023	14/12/2022	4	totea	0	1	0
2022_2023	14/12/2022	4	totea	0	1	0
2022_2023	14/12/2022	4	totea	1	0	0
2022_2023	14/12/2022	4	totea	0	1	0
2022_2023	14/12/2022	4	totea	1	2	0
2022_2023	14/12/2022	4	totea	0	2	0
2022_2023	14/12/2022	4	totea	0	1	0
2022_2023	14/12/2022	4	totea	0	1	0
2022_2023	14/12/2022	4	totea	1	0	0

2022_2023	14/12/2022	4	totea	1	1	0
2022_2023	14/12/2022	4	totea	0	1	0
2022_2023	14/12/2022	4	totea	1	0	0
2022_2023	14/12/2022	4	uie	0	0	0
2022_2023	14/12/2022	4	mbore	1	0	0
2022_2023	14/12/2022	4	mbore	1	0	0
2022_2023	14/12/2022	4	mbore	0	1	0
2022_2023	14/12/2022	4	petit koko	1	0	0
2022_2023	14/12/2022	4	tere	0	0	0
2022_2023	15/12/2022	4	uaterembi	1	0	0
2022_2023	15/12/2022	4	uaterembi	0	0	1
2022_2023	15/12/2022	4	uaterembi	0	1	0
2022_2023	15/12/2022	4	uaterembi	1	0	0
2022_2023	15/12/2022	4	uaterembi	0	1	0
2022_2023	15/12/2022	4	uaterembi	1	1	0
2022_2023	15/12/2022	4	uaterembi	0	1	0
2022_2023	15/12/2022	4	uaterembi	0	1	0
2022_2023	15/12/2022	4	uaterembi	0	0	1
2022_2023	15/12/2022	4	uaterembi	0	1	0
2022_2023	15/12/2022	4	uaterembi	1	0	0
2022_2023	15/12/2022	4	uaterembi	0	0	1
2022_2023	15/12/2022	4	uaterembi	0	0	1
2022_2023	15/12/2022	4	uaterembi	0	1	0
2022_2023	15/12/2022	4	uaterembi	0	1	0
2022_2023	15/12/2022	4	uaterembi	0	0	1
2022_2023	15/12/2022	4	mato	0	0	0
2022_2023	15/12/2022	4	noe	0	0	0
2022_2023	15/12/2022	4	puemba	1	0	0
2022_2023	15/12/2022	4	puemba	1	0	0
2022_2023	15/12/2022	4	puemba	1	0	0
2022_2023	15/12/2022	4	puemba	1	0	0
2022_2023	15/12/2022	4	puemba	1	0	0
2022_2023	15/12/2022	4	ugo	0	0	0
2022_2023	19/12/2022	5	ieroue	1	0	0
2022_2023	19/12/2022	5	ieroue	0	1	0
2022_2023	19/12/2022	5	ieroue	1	0	0
2022_2023	19/12/2022	5	ieroue	0	1	0
2022_2023	19/12/2022	5	ieroue	0	3	0
2022_2023	19/12/2022	5	ieroue	0	1	0
2022_2023	19/12/2022	5	kouare	0	1	0
2022_2023	19/12/2022	5	kouare	1	0	0
2022_2023	19/12/2022	5	kouare	1	0	0
2022_2023	19/12/2022	5	nda	1	0	0
2022_2023	19/12/2022	5	nda	1	0	0
2022_2023	19/12/2022	5	nda	1	0	0
2022_2023	19/12/2022	5	nda	0	0	1
2022_2023	19/12/2022	5	nda	1	1	0
2022_2023	19/12/2022	5	nda	0	0	1
2022_2023	19/12/2022	5	nda	0	0	1
2022_2023	19/12/2022	5	nda	0	1	0

2022_2023	19/12/2022	5 nda	1	0	0
2022_2023	19/12/2022	5 nda	0	0	1
2022_2023	19/12/2022	5 nda	0	2	0
2022_2023	19/12/2022	5 nda	0	1	0
2022_2023	19/12/2022	5 nda	0	1	0
2022_2023	19/12/2022	5 nda	0	0	1
2022_2023	19/12/2022	5 nda	1	0	0
2022_2023	19/12/2022	5 vua	0	0	1
2022_2023	19/12/2022	5 vua	0	1	0
2022_2023	19/12/2022	5 vua	0	0	1
2022_2023	19/12/2022	5 vua	1	3	0
2022_2023	19/12/2022	5 vua	1	0	0
2022_2023	19/12/2022	5 vua	1	0	0
2022_2023	19/12/2022	5 vua	0	2	0
2022_2023	19/12/2022	5 vua	0	0	1
2022_2023	19/12/2022	5 vua	0	1	0
2022_2023	19/12/2022	5 vua	1	0	0
2022_2023	19/12/2022	5 vua	0	1	0
2022_2023	19/12/2022	5 atire	0	1	0
2022_2023	19/12/2022	5 atire	1	0	0
2022_2023	19/12/2022	5 atire	1	2	0
2022_2023	19/12/2022	5 atire	1	3	0
2022_2023	19/12/2022	5 atire	1	0	0
2022_2023	19/12/2022	5 atire	1	1	0
2022_2023	19/12/2022	5 atire	1	0	0
2022_2023	19/12/2022	5 atire	0	1	0
2022_2023	19/12/2022	5 atire	0	0	1
2022_2023	19/12/2022	5 atire	1	0	0
2022_2023	19/12/2022	5 atire	0	3	0
2022_2023	19/12/2022	5 atire	1	0	0
2022_2023	19/12/2022	5 atire	1	0	0
2022_2023	22/12/2022	6 kie	1	0	0
2022_2023	22/12/2022	6 kie	1	0	0
2022_2023	22/12/2022	6 kie	0	0	1
2022_2023	22/12/2022	6 kie	1	0	0
2022_2023	22/12/2022	6 kie	0	0	1
2022_2023	22/12/2022	6 kie	1	0	0
2022_2023	22/12/2022	6 kie	1	0	0
2022_2023	22/12/2022	6 kie	0	0	1
2022_2023	22/12/2022	6 kie	1	0	0
2022_2023	22/12/2022	6 kie	1	0	0
2022_2023	22/12/2022	6 kie	1	0	0
2022_2023	22/12/2022	6 kie	1	0	0
2022_2023	22/12/2022	6 kie	1	0	0
2022_2023	22/12/2022	6 amere	1	0	0
2022_2023	22/12/2022	6 amere	0	0	1
2022_2023	22/12/2022	6 amere	0	1	0
2022_2023	22/12/2022	6 amere	0	1	0
2022_2023	22/12/2022	6 amere	0	0	1
2022_2023	22/12/2022	6 amere	1	0	0

2022_2023	22/12/2022	6	nouare	0	0	0
2022_2023	29/12/2022	7	uo	0	0	0
2022_2023	29/12/2022	7	ndo	1	0	0
2022_2023	29/12/2022	7	ndo	1	0	0
2022_2023	29/12/2022	7	ndo	1	0	0
2022_2023	29/12/2022	7	ndo	1	0	0
2022_2023	29/12/2022	7	ndo	1	0	0
2022_2023	29/12/2022	7	ndo	1	0	0
2022_2023	29/12/2022	7	ndo	1	0	0
2022_2023	29/12/2022	7	ndo	1	0	0
2022_2023	29/12/2022	7	ndo	1	1	0
2022_2023	29/12/2022	7	totea	1	2	0
2022_2023	29/12/2022	7	totea	1	1	0
2022_2023	29/12/2022	7	totea	1	0	0
2022_2023	29/12/2022	7	totea	1	0	0
2022_2023	29/12/2022	7	totea	0	1	0
2022_2023	29/12/2022	7	totea	0	0	1
2022_2023	29/12/2022	7	totea	0	2	0
2022_2023	29/12/2022	7	totea	0	2	0
2022_2023	29/12/2022	7	totea	0	3	0
2022_2023	29/12/2022	7	totea	1	1	0
2022_2023	29/12/2022	7	totea	0	3	0
2022_2023	29/12/2022	7	uie	0	0	0
2022_2023	29/12/2022	7	mbore	1	0	0
2022_2023	29/12/2022	7	mbore	0	1	0
2022_2023	29/12/2022	7	petit koko	1	0	0
2022_2023	29/12/2022	7	tere	0	0	0
2022_2023	29/12/2022	7	nge	1	0	0
2022_2023	29/12/2022	7	nge	0	1	0
2022_2023	29/12/2022	7	nge	1	0	0
2022_2023	29/12/2022	7	nge	0	2	0
2022_2023	29/12/2022	7	nge	0	0	1
2022_2023	29/12/2022	7	nge	1	0	0
2022_2023	29/12/2022	7	nge	1	0	0
2022_2023	30/12/2022	7	mato	0	1	0
2022_2023	30/12/2022	7	noe	0	0	0
2022_2023	30/12/2022	7	puemba	1	0	0
2022_2023	30/12/2022	7	puemba	1	0	0
2022_2023	30/12/2022	7	ugo	0	0	0
2022_2023	03/01/2023	8	ua	0	1	0
2022_2023	03/01/2023	8	ua	1	0	0
2022_2023	03/01/2023	8	ua	0	0	1
2022_2023	03/01/2023	8	ua	1	0	0
2022_2023	03/01/2023	8	ua	0	1	0
2022_2023	03/01/2023	8	uaterembi	1	0	0
2022_2023	03/01/2023	8	uaterembi	1	1	0
2022_2023	03/01/2023	8	uaterembi	1	1	0
2022_2023	03/01/2023	8	uaterembi	0	2	0
2022_2023	03/01/2023	8	uaterembi	0	2	0
2022_2023	03/01/2023	8	uaterembi	0	1	0

2022_2023	03/01/2023	8	uaterembi	1	0	0
2022_2023	03/01/2023	8	uaterembi	0	0	1
2022_2023	03/01/2023	8	uaterembi	0	1	0
2022_2023	03/01/2023	8	uaterembi	0	1	0
2022_2023	03/01/2023	8	uaterembi	1	0	0
2022_2023	04/01/2023	8	gi	1	0	0
2022_2023	04/01/2023	8	gi	0	0	1
2022_2023	04/01/2023	8	gi	0	2	0
2022_2023	04/01/2023	8	gi	1	0	0
2022_2023	04/01/2023	8	gi	0	2	0
2022_2023	04/01/2023	8	gi	1	1	0
2022_2023	04/01/2023	8	gi	1	0	0
2022_2023	04/01/2023	8	gi	0	0	1
2022_2023	04/01/2023	8	uatio	1	0	0
2022_2023	04/01/2023	8	uatio	0	2	0
2022_2023	04/01/2023	8	uatio	1	0	0
2022_2023	04/01/2023	8	uatio	0	1	0
2022_2023	04/01/2023	8	uatio	1	0	0
2022_2023	04/01/2023	8	uatio	1	0	0
2022_2023	04/01/2023	8	uatio	1	2	0
2022_2023	04/01/2023	8	uatio	0	1	0
2022_2023	04/01/2023	8	uatio	0	0	1
2022_2023	04/01/2023	8	uatio	1	1	0
2022_2023	11/01/2023	9	ieroue	0	1	0
2022_2023	11/01/2023	9	ieroue	1	0	0
2022_2023	11/01/2023	9	ieroue	1	0	0
2022_2023	11/01/2023	9	nge	1	0	0
2022_2023	11/01/2023	9	nge	0	0	1
2022_2023	11/01/2023	9	nge	0	0	1
2022_2023	11/01/2023	9	nge	0	0	1
2022_2023	11/01/2023	9	nge	0	0	1
2022_2023	11/01/2023	9	nge	1	0	0
2022_2023	11/01/2023	9	nge	0	0	1
2022_2023	11/01/2023	9	nge	1	0	0
2022_2023	11/01/2023	9	nge	0	1	0
2022_2023	11/01/2023	9	nge	1	0	0
2022_2023	11/01/2023	9	nge	1	0	0
2022_2023	11/01/2023	9	gi	1	1	0
2022_2023	11/01/2023	9	gi	0	0	1
2022_2023	11/01/2023	9	gi	0	4	0
2022_2023	11/01/2023	9	gi	1	1	0
2022_2023	11/01/2023	9	gi	1	0	0
2022_2023	11/01/2023	9	gi	1	0	0
2022_2023	11/01/2023	9	tere	0	0	0
2022_2023	11/01/2023	9	nda	1	0	0
2022_2023	11/01/2023	9	nda	1	0	0
2022_2023	11/01/2023	9	nda	1	2	0
2022_2023	11/01/2023	9	nda	1	0	0
2022_2023	11/01/2023	9	nda	1	0	0
2022_2023	11/01/2023	9	nda	0	4	0

2022_2023	11/01/2023	9 nda	0	3	0
2022_2023	11/01/2023	9 nda	0	1	0
2022_2023	11/01/2023	9 nda	1	0	0
2022_2023	11/01/2023	9 nda	0	1	0
2022_2023	11/01/2023	9 nda	1	0	0
2022_2023	11/01/2023	9 nda	1	0	0
2022_2023	11/01/2023	9 nda	0	0	1
2022_2023	11/01/2023	9 nda	0	1	0
2022_2023	11/01/2023	9 nda	0	1	0
2022_2023	11/01/2023	9 nda	0	0	1
2022_2023	11/01/2023	9 nda	1	0	0
2022_2023	11/01/2023	9 nda	0	2	0
2022_2023	11/01/2023	9 kouare	0	0	1
2022_2023	11/01/2023	9 kouare	0	0	1
2022_2023	11/01/2023	9 kouare	1	0	0
2022_2023	11/01/2023	9 kouare	1	0	0
2022_2023	11/01/2023	9 kouare	1	0	0
2022_2023	11/01/2023	9 kouare	0	1	0
2022_2023	11/01/2023	9 kouare	0	1	0
2022_2023	11/01/2023	9 kouare	0	0	1
2022_2023	12/01/2023	9 uatio	1	0	0
2022_2023	12/01/2023	9 uatio	0	1	0
2022_2023	12/01/2023	9 uatio	1	1	0
2022_2023	12/01/2023	9 uaterembi	0	1	0
2022_2023	12/01/2023	9 uaterembi	1	1	0
2022_2023	12/01/2023	9 uaterembi	1	0	0
2022_2023	12/01/2023	9 vua	1	0	0
2022_2023	12/01/2023	9 vua	0	1	0
2022_2023	12/01/2023	9 vua	1	1	0
2022_2023	12/01/2023	9 vua	1	0	0
2022_2023	12/01/2023	9 vua	0	1	0
2022_2023	12/01/2023	9 vua	0	1	0
2022_2023	12/01/2023	9 vua	1	0	0
2022_2023	12/01/2023	9 vua	1	0	0
2022_2023	12/01/2023	9 vua	0	1	0
2022_2023	12/01/2023	9 vua	0	1	0
2022_2023	12/01/2023	9 vua	1	0	0
2022_2023	12/01/2023	9 redika	1	0	0
2022_2023	12/01/2023	9 redika	1	0	0
2022_2023	12/01/2023	9 redika	0	1	0
2022_2023	12/01/2023	9 redika	0	2	0
2022_2023	12/01/2023	9 redika	0	1	0
2022_2023	12/01/2023	9 redika	0	1	0
2022_2023	12/01/2023	9 redika	1	0	0
2022_2023	12/01/2023	9 atire	0	1	0
2022_2023	12/01/2023	9 atire	0	1	0
2022_2023	12/01/2023	9 atire	0	2	0
2022_2023	12/01/2023	9 atire	0	0	1
2022_2023	12/01/2023	9 atire	1	2	0
2022_2023	12/01/2023	9 atire	1	0	0

2022_2023	12/01/2023	9	atire	0	0	1
2022_2023	12/01/2023	9	atire	1	1	0
2022_2023	12/01/2023	9	atire	1	0	0
2022_2023	12/01/2023	9	atire	0	0	1
2022_2023	12/01/2023	9	atire	1	0	0
2022_2023	12/01/2023	9	atire	1	1	0
2022_2023	12/01/2023	9	atire	1	0	0
2022_2023	12/01/2023	9	atire	1	0	0
2022_2023	12/01/2023	9	atire	1	0	0
2022_2023	25/01/2023	10	ieroue	1	0	0
2022_2023	25/01/2023	10	ieroue	0	1	0
2022_2023	25/01/2023	10	uaterembi	0	1	0
2022_2023	15/12/2022	4	uaterembi	1	0	0
2022_2023	25/01/2023	10	uatio	0	1	0
2022_2023	25/01/2023	10	uatio	1	0	0
2022_2023	25/01/2023	10	uatio	1	0	0
2022_2023	25/01/2023	10	gi	1	0	0
2022_2023	25/01/2023	10	gi	1	0	0
2022_2023	25/01/2023	10	tere	1	0	0
2022_2023	25/01/2023	10	tere	0	0	1
2022_2023	25/01/2023	10	nda	1	0	0
2022_2023	25/01/2023	10	nda	0	0	1
2022_2023	25/01/2023	10	nda	0	1	0
2022_2023	25/01/2023	10	nda	0	0	1
2022_2023	25/01/2023	10	nda	1	0	0
2022_2023	25/01/2023	10	nda	0	1	0
2022_2023	25/01/2023	10	nda	1	0	0
2022_2023	26/01/2023	10	nge	1	0	0
2022_2023	26/01/2023	10	kouare	0	0	0
2022_2023	26/01/2023	10	ua	0	0	0
2022_2023	26/01/2023	10	vua	1	0	0
2022_2023	26/01/2023	10	vua	0	0	1
2022_2023	26/01/2023	10	vua	1	0	0
2022_2023	26/01/2023	10	vua	1	0	0
2022_2023	26/01/2023	10	vua	0	1	0
2022_2023	26/01/2023	10	vua	1	1	0
2022_2023	26/01/2023	10	atire	1	0	0
2022_2023	26/01/2023	10	atire	0	1	0
2022_2023	26/01/2023	10	atire	1	0	0
2022_2023	08/02/2023	11	ieroue	0	0	0
2022_2023	08/02/2023	11	uaterembi	0	0	1
2022_2023	08/02/2023	11	gi	1	0	0
2022_2023	08/02/2023	11	tere	1	0	0
2022_2023	08/02/2023	11	nda	1	1	0
2022_2023	08/02/2023	11	nda	1	0	0
2022_2023	08/02/2023	11	kouare	0	0	0
2022_2023	08/02/2023	11	ua	0	0	0
2022_2023	15/02/2023	12	nge	1	0	0
2022_2023	15/02/2023	12	vua	0	0	0

avorted_activi	crawl_up	crawl_down	latitude	longitude	survey_modal
0	1	1	-22,521146	166,556532	1
0	1	1	-22,5212356	166,556682	1
0	1	1	-22,5212357	166,556684	1
0	0	0	-22,5212176	166,556867	1
0	0	0	-22,5212176	166,556867	1
1	1	1	-22,5212145	166,557147	1
1	0	0	-22,5203091	166,556895	1
0	0	0	-22,5202679	166,556858	1
0	0	0	-22,5202489	166,556802	1
1	1	1	-22,5201409	166,556565	1
1	1	1	-22,5760045	166,745699	1
0	1	0	-22,5761425	166,745381	1
0	1	0	-22,5761425	166,745381	1
0	1	1	-22,6789856	166,81014	1
0	1	1	-22,6802163	166,811395	1
1	0	0	-22,6802419	166,811497	1
1	1	1	-22,6803648	166,81255	1
0	1	1	-22,6800181	166,812755	1
0	1	1	-22,7208601	166,850629	1
1	1	1	-22,7212662	166,850524	1
0	0	0	-22,7238915	166,851888	1
0	1	1	-22,7236185	166,852688	1
1	1	1	-22,7208624	166,851322	1
0	1	1	-22,7206159	166,851209	1
0	1	1	-22,7206159	166,851209	1
0	1	1	-22,6923969	166,850132	1
0	0	0	-22,6924385	166,850122	1
1	1	1	-22,6937899	166,851413	1
1	1	1	-22,6919316	166,850957	1
1	1	1	-22,6919085	166,850791	1
0	0	0	-22,6918691	166,850742	1
0	1	1	-22,6919746	166,850432	1
1	1	1	-22,6921363	166,850237	1
0	0	0	-22,7756541	166,799683	1
0	1	0	-22,7757935	166,799737	1
1	1	1	-22,7761731	166,800056	1
0	0	0	-22,7752345	166,799674	1
0	0	0	-22,8466004	166,879177	1
0	0	0	-22,8461518	166,879059	1
1	1	1	-22,8458954	166,878876	1
0	0	0	-22,8453907	166,877523	1
0	1	1	-22,8454286	166,877368	1
0	1	1	-22,8456733	166,877035	1
0	0	0	-22,7887775	166,847332	1
1	1	1	-22,7889181	166,847308	1
1	0	0	-22,5171306	166,612099	1
1	1	1	-22,5150371	166,612499	1
1	1	1	-22,5149308	166,612479	1
1	1	1	-22,5145543	166,612274	1

0	1	1	-22,5137985	166,61161	1
0	1	1	-22,5139951	166,611202	1
0	1	1	-22,5203202	166,555883	1
0	1	1	-22,5204973	166,55588	1
1	1	1	-22,5207494	166,555962	1
1	1	1	-22,5208518	166,556048	1
0	1	1	-22,5210605	166,556231	1
0	1	1	-22,5210992	166,556403	1
0	1	1	-22,5211329	166,556697	1
0	1	1	-22,5212232	166,556959	1
0	0	0	-22,5212123	166,557003	1
0	0	0	-22,5210776	166,557265	1
1	1	1	-22,5211548	166,557432	1
1	1	1	-22,5211352	166,557702	1
1	0	0	-22,5203622	166,55701	1
0	1	0	-22,5200997	166,556428	1
0	1	1	-22,5201737	166,55599	1
0	0	0	-22,5155411	166,609671	1
0	1	0	-22,5169019	166,612467	1
0	0	0	-22,514001	166,611884	1
0	1	1	-22,5774977	166,749216	1
0	1	1	-22,5759283	166,746108	1
0	1	0	-22,6047195	166,826358	1
0	0	0	-22,6043423	166,827635	1
1	1	1	-22,6795303	166,810459	1
1	1	1	-22,6796554	166,810551	1
1	1	1	-22,6797907	166,810687	1
1	1	1	-22,6799446	166,810864	1
1	1	1	-22,6803765	166,812564	1
1	1	1	-22,6803063	166,812615	1
1	1	1	-22,6802587	166,812669	1
1	1	1	-22,680029	166,812779	1
0	1	1	-22,6789291	166,812681	1
0	1	1	-22,6923914	166,850152	1
1	1	0	-22,6925868	166,849975	1
0	1	1	-22,6940839	166,851246	1
1	1	1	-22,693467	166,851561	1
0	0	0	-22,6928702	166,851553	1
1	0	0	-22,6919095	166,85091	1
1	0	0	-22,7070369	166,812388	1
1	0	0	-22,7070913	166,812435	1
1	0	0	-22,7071504	166,812364	1
0	0	0	-22,7075928	166,811943	1
0	0	0	-22,7085326	166,810362	1
1	1	0	-22,7088001	166,809836	1
1	1	1	-22,7088291	166,809878	1
0	0	0	-22,7090343	166,81019	1
1	0	0	-22,8468702	166,876767	1
1	1	1	-22,8472786	166,876988	1
1	1	1	-22,8473419	166,877107	1

1	1	1	-22,8461211	166,879036	1
1	0	0	-22,8457466	166,878696	1
1	1	1	-22,8455779	166,878468	1
1	1	1	-22,8455538	166,87836	1
1	1	1	-22,8453394	166,877431	1
0	1	1	-22,8453738	166,877379	1
1	0	0	-22,8454112	166,877396	1
0	1	1	-22,8453346	166,877341	1
1	1	1	-22,8454686	166,877289	1
1	1	1	-22,8456546	166,877117	1
0	0	0	-22,8459931	166,876927	1
0	1	0	-22,8460715	166,876947	1
1	1	1	-22,8462985	166,87688	1
0	0	0	-22,8465479	166,876819	1
0	1	1	-22,7784332	166,802271	1
0	0	0	-22,7777888	166,802741	1
1	1	1	-22,7771948	166,802819	1
1	1	1	-22,7759226	166,801082	1
1	1	1	-22,7753716	166,799486	1
0	1	1	-22,7758351	166,799822	1
1	1	1	-22,7759788	166,799904	1
1	1	1	-22,7761823	166,800078	1
0	1	1	-22,7784131	166,801888	1
0	1	0	-22,7201292	166,850948	1
0	0	0	-22,7211706	166,850524	1
0	0	0	-22,721346	166,850494	1
1	0	0	-22,7237711	166,852724	1
1	0	0	-22,7236872	166,852684	1
0	1	1	-22,7234937	166,852667	1
0	1	1	-22,7232057	166,852566	1
1	0	0	-22,7208777	166,851269	1
1	0	0	-22,7207721	166,851259	1
1	1	1	-22,7206803	166,851203	1
1	1	1	-22,7205008	166,851146	1
0	1	1	-22,7203486	166,851098	1
1	0	0	-22,7202986	166,851005	1
0	0	0	-22,8454047	166,877405	1
0	1	0	-22,8454225	166,877246	1
0	1	1	-22,8455204	166,877225	1
0	0	0	-22,8466728	166,876825	1
0	0	0	-22,7209235	166,850617	1
0	0	0	-22,7213091	166,850535	1
1	0	0	-22,7215477	166,850431	1
1	1	1	-22,7237526	166,852703	1
0	0	0	-22,7230141	166,852516	1
0	0	0	-22,7205391	166,851135	1
0	1	0	-22,7203281	166,851127	1
0	0	0	-22,7201784	166,851047	1
0	0	0	-22,7202034	166,850971	1
1	0	0	-22,6921627	166,850246	1

0	0	0	-22,692458	166,850051	1
1	0	0	-22,6934511	166,851553	1
0	1	1	-22,6920266	166,851077	1
0	0	0	-22,6775197	166,810263	1
0	0	0	-22,6795698	166,810478	1
0	0	0	-22,6796088	166,810513	1
1	0	0	-22,6797806	166,810695	1
1	0	0	-22,6799214	166,810883	1
0	0	0	-22,6802511	166,811377	1
1	0	0	-22,6803971	166,812299	1
1	0	0	-22,6804019	166,812427	1
0	1	1	-22,6803711	166,812574	1
0	0	0	-22,680306	166,812647	1
0	0	0	-22,6802554	166,812624	1
0	1	1	-22,6802142	166,812645	1
1	1	1	-22,6799297	166,812864	1
0	1	1	-22,6798321	166,812862	1
0	1	1	-22,6796678	166,812917	1
1	1	1	-22,5201276	166,556059	1
0	0	0	-22,520156	166,55603	1
0	0	0	-22,5201895	166,555977	1
1	1	1	-22,520429	166,555853	1
0	0	0	-22,5207569	166,555938	1
0	0	0	-22,5209854	166,556187	1
0	0	0	-22,5209878	166,556218	1
0	1	1	-22,5211088	166,556449	1
0	0	0	-22,5211833	166,556506	1
0	0	0	-22,5211186	166,556578	1
0	0	0	-22,5212284	166,556725	1
1	1	1	-22,5211229	166,556805	1
1	0	0	-22,5211631	166,557051	1
0	1	1	-22,5211341	166,557071	1
0	1	1	-22,5211827	166,557382	1
1	0	0	-22,5211314	166,557541	1
1	1	1	-22,5210502	166,55754	1
1	1	1	-22,5210069	166,557688	1
1	0	0	-22,5204129	166,557092	1
0	0	0	-22,5203733	166,55703	1
1	0	0	-22,5201193	166,556507	1
0	0	0	-22,5142176	166,6106	1
0	0	0	-22,5172435	166,610008	1
0	0	0	-22,5172435	166,610008	1
0	0	0	-22,5173264	166,610869	1
1	0	0	-22,516955	166,612427	1
1	0	0	-22,5152003	166,612546	1
1	0	0	-22,5148982	166,612416	1
1	0	0	-22,5148171	166,612389	1
0	0	0	-22,5147355	166,612407	1
0	0	1	-22,514457	166,612159	1
1	1	1	-22,5143165	166,612129	1

0	0	0	-22,5141735	166,611969	1
0	1	1	-22,5139517	166,611917	1
0	1	1	-22,5139517	166,611917	1
1	1	1	-22,5138839	166,611849	1
1	0	0	-22,5138885	166,61174	1
0	0	0	-22,5138866	166,611655	1
0	0	0	-22,5138866	166,611655	1
0	1	0	-22,5137579	166,611523	1
0	0	0	-22,5138021	166,611511	1
0	0	0	-22,5137605	166,611483	1
1	0	0	-22,57618	166,74533	1
0	0	0	-22,5763967	166,74515	1
0	0	0	-22,5770218	166,745302	1
0	0	0	-22,5771371	166,745559	1
0	0	0	-22,5776198	166,749257	1
0	0	0	-22,5759803	166,747164	1
0	0	0	-22,5759181	166,746704	1
1	0	0	-22,5758987	166,746647	1
0	0	0	-22,6049459	166,826446	1
0	0	0	-22,6049906	166,826532	1
1	0	0	-22,8456207	166,878478	1
0	0	0	-22,8454995	166,878247	1
0	0	0	-22,8453565	166,877728	1
1	0	0	-22,8460223	166,87698	1
0	0	0	-22,7889207	166,847331	1
0	0	0	-22,7237592	166,852701	1
0	0	0	-22,7237592	166,852701	1
0	0	0	-22,7236975	166,852643	1
0	0	0	-22,7236347	166,852699	1
0	1	1	-22,7236151	166,852674	1
1	0	0	-22,7204349	166,851102	1
0	0	0	-22,6801237	166,812731	1
0	0	0	-22,6800383	166,812812	1
1	0	0	-22,6798767	166,812877	1
0	0	0	-22,6797281	166,812917	1
0	0	0	-22,6049845	166,826551	1
0	0	0	-22,6043439	166,827631	1
0	0	0	-22,5775443	166,74925	1
0	0	0	-22,5764581	166,74823	1
1	1	1	-22,5140192	166,610944	1
1	1	1	-22,5170587	166,612354	1
1	0	0	-22,5147739	166,612355	1
0	0	0	-22,5144999	166,612206	1
0	0	0	-22,514497	166,612136	1
0	1	1	-22,5142324	166,612032	1
0	0	0	-22,51385	166,611626	1
1	0	0	-22,5138988	166,6118	1
0	0	0	-22,5211652	166,55694	1
0	0	0	-22,5204462	166,557078	1
0	0	0	-22,8455537	166,878342	1

0	0	0	-22,845956	166,876981	1
0	0	0	-22,6936499	166,849777	1
0	0	0	-22,6802388	166,812568	1
0	0	0	-22,5137452	166,611644	1
0	0	0	-22,5137974	166,611538	1
0	0	0	-22,5210807	166,557251	1
0	1	1	-22,5211852	166,556791	1
1	1	1	-22,5212473	166,556891	1
0	1	1	-22,7095996	166,795891	1
0	1	1	-22,6922183	166,850189	1
0	0	0	-22,6931636	166,851602	1
1	1	1	-22,6927062	166,851505	1
1	1	1	-22,6925215	166,851447	1
0	1	1	-22,6925027	166,851408	1
1	1	1	-22,6923824	166,85138	1
1	1	1	-22,6919887	166,851048	1
1	1	1	-22,6919355	166,850993	1
1	0	0	-22,6918767	166,850825	1
1	1	1	-22,6919234	166,850727	1
1	0	0	-22,6919358	166,85066	1
1	1	1	-22,6919339	166,850605	1
1	1	1	-22,8476805	166,878461	1
0	1	1	-22,8476462	166,878471	1
0	1	1	-22,7205291	166,851167	1
1	1	1	-22,6022284	166,827443	1
0	0	0	-22,6020384	166,827757	1
0	0	0	-22,5147698	166,612351	1
0	0	0	-22,5776703	166,749078	1
1	0	1	-22,577252	166,748996	1
0	0	0	-22,6803905	166,812167	1
1	1	1	nd	nd	1
1	0	0	nd	nd	1
0	0	0	-22,7099274	166,79658	1
1	0	0	-22,7113335	166,794782	1
0	1	1	-22,710652	166,797379	1
0	1	1	-22,7105274	166,797477	1
1	1	1	-22,7784953	166,802292	1
1	1	1	-22,7783031	166,802385	1
0	1	1	-22,776343	166,800192	1
0	1	1	-22,7203773	166,851069	1
1	1	1	-22,7202238	166,851098	1
0	1	0	-22,7201621	166,85102	1
0	1	1	-22,7201983	166,850975	1
0	1	1	-22,7237565	166,852758	1
1	1	1	nd	nd	1
1	0	0	-22,7214174	166,851611	1
1	1	1	-22,8464331	166,876846	1
1	0	0	-22,6918629	166,85081	1
0	0	0	-22,6918919	166,850728	1
1	0	0	-22,6919342	166,850677	1

1	0	0	-22,6918951	166,850643	1
1	0	0	-22,6918908	166,850614	1
0	1	1	-22,6935588	166,851457	1
0	0	0	-22,6932885	166,851607	1
0	1	1	-22,6923234	166,851317	1
0	0	0	-22,6922912	166,851324	1
1	0	0	-22,6921893	166,851271	1
1	1	1	-22,6921375	166,851273	1
1	1	1	-22,6920332	166,851222	1
1	0	0	-22,6918617	166,850813	1
0	0	0	-22,5203669	166,555877	1
1	0	0	-22,5208138	166,555994	1
0	0	0	-22,5211084	166,556366	1
0	1	0	-22,5209838	166,557762	1
0	1	1	-22,5142233	166,610677	1
1	1	1	-22,5143077	166,610582	1
1	1	1	-22,5169811	166,612434	1
0	0	0	-22,513966	166,611204	1
0	0	0	-22,513966	166,611204	1
1	1	1	-22,5769011	166,7452	1
1	1	1	-22,5774095	166,749226	1
1	0	0	-22,5761413	166,747802	1
0	0	0	-22,5759157	166,746825	1
0	0	0	-22,5759134	166,746821	1
0	1	1	-22,5758644	166,746391	1
1	1	1	-22,5759457	166,745881	1
0	0	0	-22,57616	166,745294	1
1	1	1	-22,6024149	166,827116	1
1	1	1	-22,6024601	166,827052	1
0	0	0	-22,6025038	166,826962	1
0	1	1	-22,6803613	166,812653	1
1	1	1	-22,6800851	166,812795	1
0	1	1	-22,6798976	166,812892	1
1	1	1	-22,6775411	166,810563	1
1	0	0	-22,7112164	166,796478	1
1	1	1	-22,7108737	166,797038	1
0	1	1	-22,7106697	166,797415	1
1	1	1	-22,7106335	166,797459	1
1	1	1	-22,7095114	166,795783	1
1	1	1	-22,709387	166,795158	1
1	0	0	-22,7095888	166,794574	1
0	0	0	-22,5759157	166,746825	1
0	1	0	-22,7083739	166,810725	1
1	0	0	-22,6918515	166,850777	1
1	0	0	-22,691966	166,851054	1
0	0	0	-22,692066	166,851182	1
1	0	0	-22,6923861	166,851383	1
0	1	1	-22,6924997	166,851408	1
1	1	1	-22,6925317	166,851427	1
1	0	0	-22,6935465	166,851497	1

0	0	0	-22,6935763	166,851494	1
0	1	1	-22,6921006	166,850282	1
0	0	0	-22,6920223	166,850388	1
1	1	0	-22,6918733	166,850542	1
1	0	0	-22,6918933	166,85065	1
0	1	1	-22,8472996	166,876988	1
1	0	0	-22,7214682	166,851649	1
0	0	0	-22,7213779	166,851588	1
0	1	1	-22,720684	166,851272	1
0	1	1	-22,7206155	166,851196	1
0	1	0	-22,7205508	166,851221	1
0	0	0	-22,7203773	166,851069	1
0	1	1	-22,7201122	166,850957	1
0	1	1	-22,7214378	166,850478	1
1	1	1	-22,7239604	166,852256	1
0	0	0	-22,7238821	166,852643	1
0	0	0	-22,723815	166,852929	1
1	1	1	-22,7236646	166,85278	1
1	0	0	-22,7234895	166,852652	1
1	1	1	-22,7232518	166,852608	1
0	1	1	-22,7785229	166,802181	1
1	1	1	-22,7784738	166,802346	1
1	1	1	-22,7783048	166,802426	1
1	1	1	-22,7782844	166,802457	1
0	0	0	-22,7780674	166,80254	1
0	1	1	-22,7780612	166,802571	1
1	1	1	-22,7772726	166,802822	1
1	1	1	-22,776896	166,802806	1
1	1	1	-22,7763644	166,802429	1
0	0	0	-22,775963	166,801114	1
1	1	1	-22,775737	166,800646	1
1	1	0	-22,7756329	166,79964	1
0	1	1	-22,7759556	166,79988	1
0	1	1	-22,7761799	166,800092	1
1	1	1	-22,7773787	166,800938	1
0	0	0	-22,7775571	166,801102	1
1	1	1	-22,777768	166,801213	1
1	1	1	-22,7777867	166,801282	1
0	1	1	-22,5204495	166,55588	1
0	0	0	-22,5208125	166,556008	1
0	1	1	-22,521162	166,556389	1
0	0	0	-22,5211852	166,556791	1
1	1	1	-22,5212706	166,556569	1
1	1	1	-22,5212599	166,556846	1
0	1	1	-22,5211973	166,557111	1
0	1	1	-22,5211994	166,557229	1
0	0	0	-22,5211063	166,557235	1
0	1	1	-22,5211082	166,557447	1
0	1	1	-22,5211094	166,55748	1
1	1	1	-22,5211774	166,557556	1

1	1	1	-22,5206998	166,557569	1
0	1	1	-22,5205016	166,557315	1
0	1	1	-22,5202282	166,55669	1
0	1	1	-22,5202362	166,556416	1
0	1	1	-22,7758	166,799767	1
1	1	0	-22,7757333	166,799683	1
0	1	0	-22,7755	166,79965	1
1	1	1	-22,77525	166,799533	1
1	1	1	-22,7758167	166,80085	1
1	1	0	-22,7767167	166,80275	1
0	0	0	-22,7780167	166,802567	1
1	1	1	-22,5204368	166,555876	1
0	1	1	-22,5206064	166,555933	1
0	1	1	-22,5209713	166,556183	1
1	1	0	-22,5211981	166,556591	1
0	1	1	-22,5212162	166,556654	1
1	1	1	-22,5212398	166,556863	1
0	0	0	-22,5212166	166,556835	1
0	0	0	-22,5212336	166,556928	1
1	1	1	-22,5212693	166,556975	1
1	0	0	-22,5211258	166,557039	1
1	0	0	-22,521208	166,557355	1
0	0	0	-22,5211356	166,557442	1
0	0	0	-22,5210362	166,557547	1
1	0	0	-22,5209232	166,557684	1
1	1	1	-22,5204106	166,557036	1
1	1	1	-22,5202433	166,556686	1
0	1	1	-22,520168	166,556555	1
1	1	1	-22,5201609	166,556418	1
0	1	1	-22,5201701	166,556358	1
0	1	1	-22,5201721	166,556128	1
1	1	1	-22,5201653	166,556063	1
0	1	1	-22,5759748	166,745834	1
0	1	1	-22,5760025	166,745765	1
0	1	1	-22,5760125	166,745713	1
0	1	1	-22,5760395	166,745554	1
0	1	1	-22,5761047	166,745426	1
0	1	1	-22,5761433	166,74538	1
0	0	0	-22,5761537	166,74535	1
1	1	1	-22,5774775	166,749209	1
0	0	0	-22,5759182	166,746708	1
0	0	0	-22,5759095	166,746662	1
1	1	0	-22,57596	166,745963	1
1	1	1	-22,679125	166,810151	1
1	1	0	-22,6791891	166,810222	1
0	0	0	-22,6802543	166,811444	1
1	0	0	-22,6803886	166,812163	1
1	0	0	-22,6804091	166,812274	1
1	1	1	-22,6803956	166,812555	1
0	1	0	-22,6802288	166,812663	1

0	0	0	-22,6802108	166,812764	1
0	1	1	-22,6801168	166,812776	1
1	1	1	-22,6793162	166,812877	1
1	1	1	-22,6790623	166,812762	1
0	1	1	-22,6789076	166,812681	1
0	1	1	-22,84697	166,876731	1
1	1	1	-22,8473583	166,877084	1
1	1	1	-22,8476984	166,878294	1
1	1	1	-22,8476854	166,878343	1
0	1	1	-22,8476593	166,878404	1
1	1	1	-22,8458792	166,878913	1
0	1	1	-22,6919484	166,850542	1
0	1	1	-22,6919484	166,850542	1
0	0	0	-22,6919401	166,850457	1
0	1	1	-22,6919641	166,850449	1
1	1	1	-22,6919853	166,850388	1
0	1	1	-22,6920427	166,850299	1
1	1	1	-22,6921385	166,850249	1
1	1	1	-22,6922124	166,850206	1
1	1	1	-22,6923447	166,850109	1
1	1	1	-22,6924195	166,850093	1
1	1	1	-22,6924276	166,850109	1
0	1	1	-22,6926665	166,849982	1
1	1	1	-22,6941325	166,851266	1
0	1	1	-22,6940349	166,851324	1
0	1	1	-22,6940349	166,851324	1
0	1	1	-22,6939101	166,851339	1
0	1	1	-22,6938752	166,851367	1
1	1	1	-22,6934682	166,851579	1
0	0	0	-22,6920678	166,851162	1
1	0	0	-22,6919581	166,851043	1
0	1	1	-22,6919178	166,851017	1
0	1	0	-22,6919346	166,850914	1
0	0	0	-22,691927	166,850734	1
1	0	0	-22,6922912	166,851324	1
0	1	1	-22,6919288	166,850487	1
0	1	1	-22,7207175	166,850663	1
0	1	0	-22,7208685	166,850649	1
0	0	0	-22,7210012	166,850612	1
1	1	1	-22,7213554	166,8505	1
0	1	1	-22,7237053	166,852749	1
1	1	1	-22,723623	166,852728	1
1	1	1	-22,7233689	166,852632	1
1	0	0	-22,7229777	166,852514	1
0	0	0	-22,721333	166,851581	1
0	1	1	-22,7209418	166,851387	1
0	1	1	-22,7209253	166,85135	1
0	0	0	-22,7208533	166,851326	1
1	1	0	-22,720786	166,851277	1
1	0	0	-22,7207174	166,851242	1

1	0	0	-22,720697	166,85123	1
0	0	0	-22,7206281	166,851245	1
0	0	0	-22,720312	166,851132	1
1	0	0	-22,709783	166,796342	1
0	0	0	-22,7097919	166,796294	1
1	1	0	-22,7095374	166,795797	1
1	1	1	-22,7093994	166,795172	1
0	1	1	-22,7094482	166,794896	1
0	1	1	-22,7094525	166,794862	1
0	1	1	-22,7094729	166,794764	1
1	1	1	-22,7112944	166,794584	1
0	0	1	-22,7114318	166,795088	1
0	1	1	-22,7107253	166,797339	1
1	0	0	-22,7106491	166,797465	1
1	0	0	-22,5163072	166,609506	1
1	1	1	-22,5155028	166,612635	1
1	0	0	-22,5151978	166,612533	1
0	0	0	-22,5151355	166,612508	1
0	1	1	-22,5150023	166,61249	1
1	1	1	-22,514598	166,612307	1
0	0	0	-22,5145322	166,612165	1
1	1	1	-22,5140565	166,611914	1
0	0	0	-22,5138196	166,611685	1
0	1	0	-22,513746	166,611531	1
1	0	0	-22,513966	166,611204	1
0	1	1	-22,3778569	167,062011	0
0	0	0	-22,3778573	167,062033	0
0	0	0	-22,3779196	167,06202	0
0	0	0	-22,3779509	167,062007	0
0	0	0	-22,3779818	167,062088	0
0	0	0	-22,3783324	167,064109	0
0	0	0	-22,376114	167,065456	0
0	0	0	-22,3758068	167,065567	0
0	0	0	-22,3757812	167,065589	0
0	0	0	-22,3757183	167,065601	0
0	0	0	-22,3753976	167,065663	0
0	0	0	-22,3752931	167,065695	0
0	0	0	-22,3751902	167,065671	0
0	0	0	-22,3751705	167,065638	0
0	0	0	-22,3751765	167,065597	0
0	0	0	-22,3751785	167,065487	0
0	0	0	-22,3752411	167,065452	0
0	0	0	-22,3753147	167,065061	0
1	0	0	-22,3753414	167,064945	0
0	0	0	-22,3767685	167,062802	0
0	1	1	-22,4440577	167,094855	0
0	1	1	-22,4440964	167,094824	0
1	0	0	-22,4441169	167,094762	0
0	1	1	-22,4446622	167,094114	0
0	0	0	-22,4454771	167,09362	0

0	1	1	-22,4453588	167,093816	0
0	0	0	-22,4453511	167,093949	0
1	0	0	-22,4441981	167,098383	0
1	0	0	-22,4438128	167,098375	0
1	0	0	-22,4437282	167,09836	0
0	0	0	-22,44345	167,09566	0
0	0	0	-22,4436558	167,095095	0
0	0	0	-22,4437138	167,095077	0
0	0	0	-22,4438451	167,094966	0
0	1	0	-22,6250116	167,27939	0
1	0	0	-22,6254158	167,280017	0
0	0	0	-22,8799409	166,933012	0
0	1	1	-22,8801641	166,933296	0
0	0	0	-22,8807247	166,933684	0
1	1	1	-22,8814732	166,933325	0
0	1	1	-22,8816292	166,932786	0
1	1	1	-22,8803715	166,932541	0
0	0	0	-22,8800627	166,932648	0
1	1	1	-22,8800586	166,932683	0
0	0	0	-22,8800114	166,932813	0
0	1	1	-22,8799291	166,932801	0
0	1	1	-22,8584523	166,94387	0
0	1	1	-22,8586735	166,9441	0
1	1	1	-22,8586202	166,944119	0
0	0	0	-22,8582403	166,943793	0
0	1	1	-22,7202671	166,97328	0
0	1	1	-22,7206044	166,972862	0
0	1	1	-22,720881	166,972858	0
1	1	1	-22,7195909	166,973968	0
0	0	0	-22,6830645	166,968837	0
0	1	1	-22,7218137	166,917233	0
1	0	0	-22,7219656	166,918049	0
0	0	0	-22,5162509	166,769	0
0	0	0	-22,5168191	166,770049	0
0	0	0	-22,5496931	166,793229	0
1	0	0	-22,5387024	166,821782	0
0	1	1	-22,5202188	166,556509	1
1	1	1	-22,6796033	166,810509	1
1	1	1	-22,6798919	166,810834	1
1	1	1	-22,6799217	166,81087	1
1	1	1	-22,680015	166,810976	1
1	1	1	-22,6803967	166,812437	1
0	1	1	-22,6801388	166,812769	1
1	1	1	-22,6800341	166,812804	1
1	1	1	-22,6799569	166,812891	1
1	1	1	-22,6798467	166,812875	1
1	1	1	-22,6797979	166,812916	1
1	1	1	-22,6796215	166,812911	1
1	1	1	-22,6794142	166,812925	1
1	1	1	-22,6775613	166,810424	1

1	1	1	-22,6921547	166,850205	1
1	1	1	-22,6923952	166,850129	1
0	1	1	-22,720179	166,850998	1
0	1	1	-22,7201162	166,85097	1
0	1	1	-22,7201466	166,850954	1
1	1	1	-22,7206741	166,850723	1
1	1	1	-22,7208096	166,850679	1
1	1	1	-22,7237599	166,852804	1
0	1	1	-22,7230461	166,852529	1
1	1	1	-22,7209279	166,851338	1
0	1	1	-22,7208089	166,851308	1
1	0	1	-22,720685	166,851231	1
1	1	1	-22,7205675	166,851174	1
1	0	1	-22,7204031	166,851148	1
1	1	1	-22,7101101	166,796985	1
1	1	0	-22,7098767	166,796519	1
1	1	1	-22,7098252	166,796342	1
0	0	0	-22,7094254	166,795053	1
1	1	1	-22,7096612	166,794494	1
1	1	1	-22,7111602	166,794385	1
1	1	1	-22,5141254	166,610823	1
1	1	1	-22,5170562	166,609688	1
0	0	0	nd	nd	1
0	0	0	nd	nd	0
0	0	0	nd	nd	0
0	0	0	nd	nd	1
1	1	0	-22,5145035	166,610324	1
1	1	1	-22,5166631	166,612619	1
1	1	1	-22,5153444	166,612612	1
1	1	1	-22,5149915	166,612438	1
1	1	1	-22,5140264	166,611862	1
1	1	1	-22,5201728	166,556492	1
1	1	1	-22,5201872	166,556428	1
1	1	1	-22,5203897	166,555889	1
0	1	1	-22,5205425	166,555869	1
1	1	1	-22,5211766	166,556444	1
1	1	1	-22,5212237	166,557222	1
1	1	1	-22,5211369	166,557166	1
0	1	1	-22,5210398	166,557566	1
1	1	1	-22,5210179	166,55788	1
1	1	1	-22,6025	166,826976	1
1	1	1	-22,6046297	166,826205	1
0	1	1	-22,6921385	166,850224	1
1	1	1	-22,6921385	166,850224	1
1	1	1	-22,6921999	166,850179	1
1	1	1	-22,6922483	166,850171	1
1	1	1	-22,6923004	166,850154	1
0	1	1	-22,692358	166,850128	1
1	1	1	-22,6923986	166,85011	1
0	1	1	-22,6924524	166,8501	1

1	0	0	-22,6926246	166,85001	1
0	1	1	-22,6938092	166,851367	1
0	1	1	-22,6924791	166,851403	1
0	1	1	-22,6922158	166,850194	1
0	1	1	-22,6922178	166,850236	1
1	1	1	-22,7234522	166,852683	1
0	1	0	-22,7209344	166,851328	1
1	1	1	-22,8470292	166,876759	0
0	1	1	-22,8470872	166,876753	0
1	1	1	-22,8471974	166,876843	0
1	1	1	-22,8462103	166,879083	0
1	1	1	-22,8460064	166,878955	0
0	1	1	-22,8454019	166,877856	0
1	1	1	-22,7753158	166,799456	1
1	1	1	-22,7756961	166,799671	1
1	1	1	-22,775952	166,799874	1
1	1	1	-22,7760301	166,799925	1
1	1	1	-22,7094598	166,795555	1
0	1	1	-22,7096497	166,794458	1
1	1	1	-22,7107423	166,794072	1
1	1	1	-22,7110256	166,794188	1
1	1	1	-22,7104412	166,797644	1
1	1	1	-22,6790007	166,810133	1
1	1	1	-22,6790836	166,810179	1
1	1	1	-22,679513	166,810475	1
1	1	1	-22,6800823	166,811075	1
1	1	1	-22,680241	166,812704	1
1	1	1	-22,6802108	166,81273	1
1	1	1	-22,6801419	166,812763	1
1	1	1	-22,6800627	166,812794	1
1	1	1	-22,6800053	166,812864	1
1	1	1	-22,679587	166,812943	1
1	1	1	-22,6794187	166,812918	1
1	1	1	-22,6792784	166,812857	1
1	1	1	-22,6789861	166,81273	1
1	1	1	-22,6775503	166,810492	1
0	1	1	-22,6775425	166,810354	1
1	1	1	-22,6774924	166,810189	1
1	1	1	-22,6774844	166,810079	1
0	1	1	-22,5759818	166,745639	0
1	1	1	-22,5766959	166,745133	0
1	1	1	-22,5767786	166,745126	0
1	1	1	-22,5770665	166,745317	0
1	1	1	-22,5771873	166,745539	0
1	1	1	-22,5773766	166,749325	0
1	1	1	-22,576931	166,748724	0
1	1	1	-22,5765283	166,748301	0
0	1	1	-22,5758492	166,746383	0
0	1	1	-22,5759213	166,746108	0
0	0	0	nd	nd	0

1	0	1	-22,5204311	166,555864	1
1	1	1	-22,5211786	166,556528	1
0	1	1	-22,5212346	166,556795	1
1	1	1	-22,5212019	166,557136	1
1	1	1	-22,5212114	166,557126	1
1	1	1	-22,5211987	166,557162	1
1	1	1	-22,5211843	166,55728	1
1	1	1	-22,5212214	166,55732	1
0	1	1	-22,5210913	166,557491	1
0	1	1	-22,5211498	166,55766	1
1	1	1	-22,5209998	166,557925	1
1	1	1	-22,5208775	166,557701	1
1	1	1	-22,5207877	166,557615	1
1	1	1	-22,5203628	166,556881	1
0	1	1	-22,5211263	166,557797	1
0	1	1	-22,520221	166,556726	1
1	1	1	-22,5202379	166,556782	1
1	1	0	-22,5202258	166,556701	1
1	1	1	-22,5201806	166,556575	1
0	1	1	-22,520131	166,556376	1
1	1	1	-22,5202778	166,555902	1
1	1	1	-22,521129	166,556458	1
0	1	1	-22,5212139	166,556823	1
0	0	0	-22,5211958	166,556814	1
1	1	1	-22,5211824	166,557207	1
0	1	1	-22,5211148	166,55734	1
1	1	1	-22,5210403	166,557469	1
1	1	0	-22,5211195	166,557504	1
1	1	1	-22,5210294	166,557598	1
1	1	1	-22,5209743	166,557755	1
1	1	1	-22,5208185	166,557571	1
1	1	1	-22,5204623	166,557131	1
0	1	1	-22,5203088	166,556915	1
1	1	1	-22,5202592	166,556819	1
1	1	1	-22,5140099	166,610932	1
0	1	1	-22,5140622	166,610902	1
1	1	1	-22,5140412	166,610869	1
1	1	1	-22,5140234	166,610862	1
1	1	1	-22,5141163	166,610833	1
1	1	1	-22,5141073	166,610795	1
1	1	1	-22,5141404	166,610773	1
0	1	1	-22,5141395	166,610741	1
1	1	1	-22,5141215	166,610694	1
1	1	1	-22,5141777	166,61063	1
1	1	1	-22,5142596	166,610604	1
1	1	1	-22,5142889	166,610498	1
1	0	0	-22,5173071	166,610567	1
0	1	1	-22,5173224	166,610804	1
1	1	1	-22,5173205	166,610876	1
1	1	1	-22,5173307	166,610973	1

1	1	1	-22,517338	166,611039	1
1	1	1	-22,5173629	166,611148	1
0	0	0	-22,5173138	166,611401	1
1	1	1	-22,5169747	166,612437	1
1	0	0	-22,5167925	166,61263	1
1	1	0	-22,5164813	166,612672	1
1	1	1	-22,5150195	166,612513	1
1	0	0	-22,5149164	166,612419	1
1	0	0	-22,5146289	166,612347	1
1	1	1	-22,5143344	166,612132	1
1	1	1	-22,5141324	166,612023	1
1	1	1	-22,5140727	166,611971	1
1	1	1	-22,5139384	166,611841	1
0	1	1	-22,5137979	166,611666	1
1	1	1	-22,513782	166,61154	1
1	1	1	-22,5138884	166,611224	1
1	1	1	-22,5137624	166,611403	1
1	1	1	-22,5138203	166,611342	1
1	1	1	-22,5138461	166,611285	1
0	0	0	-22,5138943	166,611292	1
1	1	1	-22,5139265	166,611168	1
1	1	1	-22,5139393	166,611123	1
1	1	1	-22,5140078	166,611054	1
0	1	0	-22,6778326	166,80996	1
1	0	0	-22,6799165	166,810875	1
1	1	1	-22,6802077	166,811372	1
1	1	1	-22,6803016	166,811603	1
1	1	1	-22,680383	166,811955	1
1	1	1	-22,6803829	166,812528	1
1	1	1	-22,680131	166,81283	1
1	1	1	-22,6802992	166,812687	1
1	1	1	-22,6802687	166,812685	1
1	0	1	-22,6801886	166,812725	1
1	1	1	-22,6801173	166,81279	1
1	1	1	-22,680074	166,812835	1
1	1	0	-22,6800451	166,812872	1
1	1	1	-22,6799357	166,812893	1
1	0	0	-22,6792623	166,812831	1
1	1	1	-22,6775306	166,810171	1
1	1	1	-22,7099846	166,796721	1
0	0	1	-22,7097957	166,796295	1
1	0	0	-22,7096586	166,796057	1
1	1	1	-22,7096147	166,795856	1
1	1	1	-22,7095937	166,795813	1
1	1	1	-22,7095175	166,795653	1
1	1	1	-22,7094517	166,795649	1
1	1	1	-22,7093634	166,795168	1
0	1	1	-22,7093873	166,794973	1
1	1	1	-22,7094198	166,794874	1
0	1	1	-22,7094297	166,794837	1

0	0	0	-22,709519	166,794713	1
1	1	1	-22,7095493	166,794636	1
1	1	0	-22,7095806	166,794577	1
1	1	1	-22,7096978	166,794466	1
1	1	1	-22,7096939	166,794442	1
1	1	1	-22,7111285	166,794316	1
1	1	1	-22,7103226	166,797416	1
0	0	0	-22,7201817	166,850921	1
0	0	0	-22,7202271	166,851023	1
0	0	0	-22,72043	166,851149	1
0	1	1	-22,7233351	166,852594	1
0	1	1	-22,7234896	166,852687	1
1	0	0	-22,7216589	166,850402	1
1	1	1	-22,7212532	166,85052	1
1	0	0	-22,7202653	166,850942	1
1	0	0	-22,6920582	166,851143	1
1	0	0	-22,6922526	166,851293	1
0	0	0	-22,6925121	166,85142	1
1	0	0	-22,693692	166,851432	1
1	0	0	-22,6937824	166,851368	1
0	0	0	-22,6938698	166,8514	1
0	0	0	-22,6939366	166,851309	1
1	0	0	-22,6941126	166,851243	1
0	0	0	-22,6921372	166,850238	1
1	1	1	-22,775847	166,800844	1
0	0	0	-22,7753177	166,79948	1
0	0	0	-22,7756708	166,799625	1
1	0	0	-22,7222499	166,91803	1
1	0	0	-22,7221631	166,918039	1
1	0	0	-22,7220671	166,91807	1
1	0	0	-22,7217536	166,91785	1
0	0	0	-22,7219712	166,91805	1
0	0	1	-22,7216539	166,917767	1
0	1	1	-22,7216809	166,917756	1
1	1	1	-22,7217277	166,917385	1
0	0	0	-22,7218288	166,917248	1
0	0	0	-22,7221206	166,91702	1
1	0	0	-22,722224	166,916871	1
1	0	0	-22,7223999	166,916681	1
1	0	0	-22,7229145	166,916273	1
0	1	1	-22,53203	166,828184	1
0	1	1	-22,5321967	166,828167	1
0	1	1	-22,5327137	166,828199	1
0	0	0	-22,5328147	166,831167	1
1	0	0	-22,5162196	166,769515	1
1	0	0	-22,5162565	166,76972	1
1	0	0	-22,5161809	166,769918	1
0	0	0	-22,5182614	166,76968	1
1	0	0	-22,5186791	166,769587	1
1	1	1	nd	nd	1

1	0	0	-22,6937824	166,851368	1
0	0	0	nd	nd	1
1	0	0	nd	nd	1
1	0	1	nd	nd	1
1	0	0	nd	nd	1
1	0	0	nd	nd	1
1	0	0	nd	nd	1
1	0	0	nd	nd	1
1	1	1	nd	nd	1
0	1	1	nd	nd	1
1	1	1	nd	nd	1
1	1	1	nd	nd	1
0	1	1	-22,5139887	166,61099	1
1	1	1	-22,5140236	166,610929	1
0	1	1	-22,5144689	166,610326	1
0	0	0	-22,5150314	166,612505	1
0	0	0	-22,5144692	166,612181	1
0	0	0	-22,5140771	166,611951	1
0	0	0	-22,5139269	166,611842	1
0	0	0	-22,7098378	166,796369	1
0	0	0	-22,7780625	166,802538	1
0	0	0	-22,77612	166,800024	1
0	0	0	-22,7204768	166,851097	1
0	1	1	-22,7202283	166,850984	1
1	1	1	-22,7202548	166,850875	1
1	1	1	-22,6919985	166,850366	1
1	1	1	-22,6921173	166,850235	1
0	0	0	-22,6936469	166,849762	1
0	0	0	-22,6939497	166,851287	1
0	0	0	-22,7217891	166,917849	1
0	1	1	-22,7218025	166,917803	1
1	0	0	-22,5399883	166,821243	1
0	0	0	-22,5383365	166,822325	1
0	0	0	-22,5383459	166,822258	1
1	1	1	-22,5312613	166,829087	1
1	1	1	-22,5312045	166,829348	1
1	1	1	-22,5327324	166,828256	1
0	1	1	-22,518381	166,769691	1
0	0	0	nd	nd	1
0	1	1	-22,6833687	166,96969	0
0	0	0	-22,6833682	166,969616	0
0	0	0	-22,6832899	166,969477	0
1	0	0	-22,6832585	166,969406	0
0	0	0	-22,6831803	166,969237	0
1	0	0	-22,6830668	166,968752	0
0	0	0	-22,6830013	166,968751	0
0	0	0	-22,6830205	166,968711	0
0	0	0	-22,6831645	166,968018	0
0	0	0	-22,6835313	166,96764	0
0	0	0	-22,6843663	166,966918	0

0	0	0	-22,6843716	166,966856	0
1	0	0	-22,6847924	166,966065	0
1	0	0	-22,6847189	166,966657	0
0	0	0	-22,6846843	166,967069	0
1	0	0	-22,683555	166,971218	0
1	0	0	-22,7069886	166,812491	0
0	0	0	-22,7079839	166,81131	0
0	0	0	-22,7081618	166,811015	0
0	0	0	-22,7083035	166,810739	0
1	0	0	-22,7086951	166,80993	0
0	0	0	-22,7067663	166,812945	0
0	0	0	-22,7068122	166,812562	0
1	0	0	-22,5497453	166,793244	0
0	0	0	-22,5497749	166,793195	0
1	0	0	-22,5498893	166,793162	0
1	0	0	-22,5499764	166,793164	0
1	0	0	-22,5495722	166,793361	0
0	0	0	-22,5495295	166,79331	0
1	0	0	-22,5494738	166,793244	0
1	0	0	-22,5491773	166,792549	0
1	0	0	-22,5515133	166,791616	0
1	0	0	-22,5516832	166,791319	0
1	0	0	-22,5205626	166,835519	0
1	0	0	-22,5202785	166,835624	0
0	0	0	nd	nd	0
0	0	0	nd	nd	0
0	1	1	-22,5138301	166,611297	1
1	1	1	-22,5134679	166,611208	1
1	1	1	-22,5139921	166,610979	1
1	1	1	-22,5140118	166,610954	1
1	1	1	-22,5140695	166,610833	1
1	1	1	-22,5140739	166,610799	1
1	0	0	-22,5139562	166,611844	1
0	1	1	-22,5137433	166,611519	1
1	0	0	-22,5212234	166,556994	1
0	0	0	-22,5212381	166,557046	1
0	0	0	-22,5212057	166,557122	1
0	1	1	-22,5211223	166,557426	1
0	1	1	-22,5211821	166,557738	1
0	1	1	-22,5209833	166,557745	1
1	1	1	-22,5208906	166,557726	1
0	0	0	-22,6802111	166,811382	1
1	0	0	-22,6802541	166,812642	1
0	1	1	-22,6798363	166,812899	1
1	1	1	-22,6798404	166,812864	1
1	1	1	-22,6790121	166,812746	1
1	0	0	-22,6784718	166,812475	1
1	1	1	-22,6775261	166,810495	1
1	1	1	-22,6774969	166,809997	1
1	1	1	-22,7103659	166,797457	1

1	1	1	-22,7101293	166,797065	1
0	0	0	-22,7098183	166,796358	1
0	1	1	-22,7093581	166,795108	1
0	0	0	-22,7093759	166,794998	1
0	0	0	-22,7094884	166,794725	1
0	1	1	-22,7095468	166,794616	1
1	1	1	-22,7096464	166,794499	1
0	0	0	-22,7088376	166,809868	0
0	0	0	-22,7068122	166,81257	0
1	0	0	-22,7079684	166,811323	0
0	0	0	-22,7081598	166,810995	0
1	0	0	-22,7782212	166,801719	1
0	1	1	-22,6935421	166,851526	1
0	1	0	-22,6934983	166,849769	1
1	1	1	-22,6926853	166,849983	1
0	1	0	-22,692503	166,850105	1
0	1	0	-22,692375	166,850143	1
0	0	0	-22,6918739	166,850626	1
1	1	1	-22,7218231	166,917198	1
1	1	1	-22,7219943	166,918059	1
0	0	0	-22,7223628	166,917999	1
0	0	0	-22,6021809	166,827435	1
0	1	1	-22,5401113	166,821227	1
1	1	1	-22,5402044	166,821226	1
1	1	1	-22,5393978	166,823234	1
1	1	1	-22,5385423	166,82193	1
0	1	1	-22,5314604	166,828636	1
0	1	1	-22,5316404	166,828504	1
0	0	0	nd	nd	1
1	1	1	-22,5212246	166,557047	1
0	1	1	-22,5212534	166,557296	1
1	1	1	-22,5762431	166,745291	1
0	1	1	-22,5767859	166,745148	1
1	1	1	-22,5769415	166,745164	1
0	1	1	-22,6019942	166,827701	1
1	1	1	-22,7756793	166,800397	1
1	1	1	-22,7756014	166,800332	1
0	0	1	-22,7753325	166,799637	1
1	1	1	-22,7759198	166,799825	1
0	0	1	-22,777333	166,800956	1
0	0	0	nd	nd	1
1	1	1	-22,8471827	166,876793	1
1	1	1	-22,8476996	166,878068	1
1	1	1	-22,8476798	166,878245	1
1	1	1	-22,84687	166,879147	1
0	1	1	-22,8453795	166,87779	1
1	1	1	-22,8453979	166,877789	1
1	1	1	-22,845457	166,877809	1
0	1	1	-22,8453783	166,877688	1
1	1	1	-22,8453872	166,877633	1

1	1	1	-22,8454132	166,877481	1
1	1	0	-22,6939741	166,849687	1
1	1	1	-22,6919589	166,850455	1
1	1	1	-22,6919187	166,850758	1
1	1	1	-22,6918524	166,850786	1
0	1	1	-22,6918774	166,850901	1
1	1	1	-22,6934532	166,851546	1
0	1	1	-22,720393	166,850841	1
0	1	1	-22,7200951	166,85094	1
0	1	0	-22,7204439	166,851124	1
0	1	1	-22,7067047	166,812789	1
1	1	1	-22,7105356	166,79748	1
1	1	1	-22,7107524	166,797293	1
0	1	1	-22,7095222	166,794662	1
1	1	1	-22,7097934	166,796378	1
0	1	1	-22,7099212	166,796615	1
0	0	0	nd	nd	1
1	1	1	-22,5150047	166,612456	1
1	1	1	-22,5140362	166,611765	1
0	1	1	-22,5137948	166,611545	1
1	1	1	nd	nd	1
0	0	0	nd	nd	1
0	0	0	nd	nd	1
1	1	1	nd	nd	1
1	1	1	nd	nd	1
1	1	1	nd	nd	1
1	1	1	nd	nd	1
1	1	1	nd	nd	1
0	1	1	nd	nd	1
1	1	1	nd	nd	1
1	0	0	nd	nd	1
1	0	0	nd	nd	1
1	1	1	nd	nd	1
1	1	1	nd	nd	1
1	1	1	nd	nd	1
1	1	1	nd	nd	1
1	0	0	nd	nd	1
1	0	0	nd	nd	1
0	1	1	nd	nd	1
1	1	0	nd	nd	1
1	1	1	nd	nd	1
0	1	1	nd	nd	1
0	1	1	nd	nd	1
0	1	1	nd	nd	1
1	1	0	nd	nd	1
1	1	1	nd	nd	1
1	1	1	nd	nd	1
1	0	1	nd	nd	1
0	1	1	nd	nd	1
1	1	1	nd	nd	1

1	1	1	nd	nd	1
0	1	1	nd	nd	1
1	1	1	nd	nd	1
1	1	1	nd	nd	1
1	1	1	nd	nd	1
0	0	0	nd	nd	1
0	1	1	nd	nd	1
1	1	0	nd	nd	1
1	1	1	nd	nd	1
0	0	0	nd	nd	1
1	1	1	nd	nd	1
1	1	0	nd	nd	1
1	1	1	nd	nd	1
1	1	1	nd	nd	1
0	1	1	nd	nd	1
1	1	1	nd	nd	1
1	1	0	nd	nd	1
0	1	1	nd	nd	1
0	0	0	nd	nd	1
1	1	0	nd	nd	1
1	1	1	nd	nd	1
1	1	1	-22,5767113	166,745151	1
0	1	1	-22,5770662	166,745319	1
1	1	1	-22,5760784	166,745656	1
0	1	1	-22,5203928	166,555913	1
0	0	0	-22,5205882	166,555928	1
0	1	1	-22,5213168	166,55687	1
0	1	1	-22,5212712	166,556964	1
0	1	1	-22,5211202	166,557513	1
1	1	1	-22,5207123	166,557665	1
0	1	1	-22,5204762	166,557097	1
1	1	1	-22,5202639	166,556837	1
1	1	1	-22,5202008	166,556236	1
1	1	1	-22,5201732	166,556376	1
0	0	0	nd	nd	0
0	1	1	-22,721913	166,917352	0
1	1	0	-22,7216595	166,917491	0
0	0	0	-22,6845794	166,966493	0
1	1	0	-22,6833893	166,967379	0
0	0	0	-22,7196325	166,97405	0
1	0	0	-22,720209	166,973346	0
0	1	1	-22,8032114	166,915858	0
0	1	1	-22,8032114	166,915858	0
0	0	0	nd	nd	0
1	0	0	-22,5516576	166,791409	0
1	0	0	-22,551656	166,791312	0
0	0	0	nd	nd	0
0	1	1	-22,5314837	166,828592	0
1	0	0	-22,5206824	166,835478	0
0	0	0	nd	nd	0

1	0	0	-22,4256693	167,011575	0
1	0	0	-22,4255568	167,011684	0
0	0	0	nd	nd	1
0	0	0	nd	nd	1
1	0	0	nd	nd	1
0	1	1	nd	nd	1
1	1	1	nd	nd	1
1	0	0	nd	nd	1
0	0	0	nd	nd	1
0	1	0	nd	nd	1
0	1	1	nd	nd	1
1	1	1	nd	nd	1
1	1	1	-22,679403	166,810378	1
1	1	1	-22,679458	166,810406	1
1	1	1	-22,679921	166,810839	1
1	1	1	-22,680352	166,81183	1
1	0	0	-22,680407	166,812101	1
1	1	1	-22,680438	166,81226	1
1	0	0	-22,680015	166,81282	1
0	1	1	-22,679914	166,812896	1
0	1	1	-22,679668	166,81296	1
1	1	1	-22,679577	166,812943	1
1	1	1	-22,710535	166,797515	1
1	0	0	-22,710214	166,797131	1
1	1	1	-22,709827	166,796432	1
0	1	1	-22,720174	166,850759	1
1	0	0	-22,718515	166,849304	1
0	1	0	-22,710372	166,857894	1
1	1	0	-22,710372	166,857894	1
1	0	0	-22,710372	166,857894	1
0	0	0	-22,710372	166,857894	1
0	0	0	-22,710372	166,857894	1
0	1	1	-22,691857	166,850838	1
1	0	0	-22,691869	166,850741	1
0	0	0	-22,691908	166,850711	1
1	1	1	-22,691914	166,850661	1
1	1	1	-22,69234	166,850108	1
0	1	1	-22,692474	166,850017	1
0	1	1	-22,692589	166,849991	1
1	0	0	-22,693595	166,851462	1
1	1	1	-22,693447	166,851531	1
1	0	0	-22,693027	166,8516	1
1	1	1	-22,692798	166,851558	1
1	1	1	-22,692762	166,851552	1
0	1	1	-22,692309	166,851288	1
1	1	1	-22,692049	166,851111	1
1	0	0	-22,691896	166,851007	1
1	1	1	-22,691906	166,850953	1
1	1	1	-22,776307	166,802268	1
0	0	1	-22,775861	166,800864	1

0	1	1	-22,775541	166,800185	1
1	1	1	-22,775446	166,800013	1
0	0	1	-22,775598	166,799607	1
1	0	0	-22,846688	166,876794	1
0	0	0	-22,602205	166,827453	1
1	0	0	-22,602239	166,827366	1
0	1	1	-22,520466	166,555962	1
0	1	1	-22,521132	166,556422	1
0	1	1	-22,521173	166,556511	1
1	1	1	-22,521197	166,556596	1
0	0	0	-22,52116	166,5566	1
0	1	1	-22,521237	166,556698	1
0	1	1	-22,520961	166,557734	1
0	1	1	-22,520482	166,557111	1
0	1	1	-22,520331	166,556855	1
1	1	1	-22,520217	166,556733	1
0	1	1	-22,520274	166,556602	1
0	1	0	-22,7105533	166,79751	1
1	0	1	-22,7110333	166,796755	1
1	1	1	-22,7102566	166,794168	1
1	0	1	-22,7100927	166,794173	1
1	0	1	-22,7099411	166,794274	1
1	1	1	-22,709539	166,794621	1
1	1	1	-22,7098154	166,796436	1
1	1	0	-22,7102196	166,797108	1
0	0	0	nd	nd	1
0	0	0	-22,6788268	166,810097	1
1	1	1	-22,6790394	166,810174	1
1	1	1	-22,6792747	166,81026	1
1	1	1	-22,6794008	166,810369	1
1	1	1	-22,6804374	166,81222	1
0	0	0	-22,6801694	166,81276	1
0	1	1	-22,6800526	166,812788	1
1	0	1	-22,6795733	166,812943	1
1	1	1	-22,6793825	166,812914	1
1	0	0	-22,7202545	166,850986	1
0	0	0	-22,7201409	166,850921	1
0	0	0	-22,7210328	166,851388	1
1	0	0	-22,72323	166,852623	1
1	1	1	-22,7233637	166,852658	1
0	1	1	-22,7237973	166,852763	1
0	1	1	-22,7238921	166,852627	1
1	1	1	-22,7239433	166,85252	1
1	0	0	-22,7239633	166,852303	1
1	1	0	-22,723912	166,851919	1
1	1	0	-22,7211848	166,850553	1
0	1	1	-22,8455822	166,878416	1
1	1	1	-22,8454768	166,878103	1
0	1	1	-22,845383	166,87764	1
1	1	1	-22,8453788	166,877586	1

1	1	0	-22,8453652	166,877105	1
1	1	0	-22,8457179	166,876857	1
0	0	0	nd	nd	1
1	1	1	-22,7764411	166,802555	1
0	1	1	-22,7759146	166,800973	1
1	1	0	-22,7758729	166,80084	1
0	1	1	-22,7755463	166,800198	1
0	0	0	-22,7762935	166,800231	1
0	0	0	-22,7771343	166,800781	1
0	0	0	-22,7775672	166,801148	1
0	1	1	-22,7776666	166,801253	1
1	1	1	-22,7782335	166,801685	1
1	0	0	-22,691872	166,850686	1
1	0	0	-22,6920111	166,851152	1
1	1	0	-22,6924996	166,851396	1
1	1	1	-22,6926692	166,851466	1
0	1	0	-22,6938914	166,851314	1
1	0	0	-22,694468	166,850937	1
1	0	0	-22,6022548	166,827383	1
0	0	1	-22,6049605	166,826477	1
0	0	0	-22,6042749	166,827629	1
0	0	0	-22,5761985	166,745314	1
0	1	1	-22,5764368	166,745216	1
1	1	1	-22,5767662	166,745139	1
1	1	1	-22,5769412	166,745188	1
0	1	1	-22,5772033	166,745571	1
0	0	0	-22,5772739	166,749047	1
1	0	0	-22,5771764	166,748948	1
1	0	0	-22,5765669	166,748343	1
0	1	1	-22,575993	166,747077	1
0	1	0	-22,5758788	166,746682	1
1	1	1	-22,5758427	166,746538	1
1	0	0	-22,5758738	166,746348	1
1	1	1	-22,5760607	166,745658	1
1	0	0	-22,5145574	166,610376	1
1	1	1	-22,5147367	166,61018	1
1	0	0	-22,5158896	166,609644	1
1	1	1	-22,5170345	166,609736	1
0	1	1	-22,5204708	166,555964	1
1	1	1	-22,5206969	166,555921	1
0	1	1	-22,5210352	166,556229	1
1	1	1	-22,521205	166,556518	1
1	0	0	-22,5212149	166,556588	1
1	1	1	-22,5212611	166,556712	1
1	0	0	-22,521278	166,556915	1
0	0	0	-22,5212869	166,557057	1
1	1	1	-22,5212239	166,557108	1
1	0	0	-22,521204	166,557224	1
0	1	1	-22,5211962	166,557385	1
0	1	1	-22,5208181	166,557689	1

1	0	0	-22,5205585	166,557299	1
1	1	1	-22,5201819	166,556252	1
0	0	1	-22,5202286	166,556162	1
0	0	0	nd	nd	0
0	0	0	-22,5515112	166,791583	0
1	0	0	-22,5516527	166,791412	0
1	1	1	-22,5497521	166,793229	0
1	0	0	-22,5493188	166,793023	0
1	0	0	-22,5492372	166,792801	0
1	0	0	-22,7217662	166,917333	0
0	1	1	-22,7216858	166,917464	0
0	1	1	-22,7217444	166,917684	0
1	1	1	-22,7225953	166,916454	0
1	0	0	-22,6835162	166,970165	0
1	1	0	-22,6848561	166,965926	0
0	0	0	-22,6845626	166,966466	0
1	1	0	-22,6842295	166,967052	0
0	1	1	-22,6832449	166,967605	0
0	1	1	-22,7207744	166,972727	0
1	1	1	-22,7208197	166,972734	0
0	0	0	-22,7195781	166,97397	0
0	0	0	nd	nd	0
0	0	0	nd	nd	0
0	0	0	-22,6922454	166,851356	1
1	0	0	-22,6930781	166,851667	1
1	1	1	-22,6919939	166,850346	1
0	0	0	nd	nd	0
0	0	0	-22,5328917	166,828339	0
0	0	0	-22,5328248	166,828313	0
0	0	0	-22,5206824	166,835489	0
1	0	0	-22,520586	166,835493	0
0	0	0	nd	nd	0
1	1	1	-22,5137959	166,611545	1
0	1	1	-22,5767751	166,745184	1
0	1	1	-22,5771444	166,745478	1
0	1	1	-22,5773766	166,749283	1
1	1	0	-22,5772206	166,749056	1
1	1	1	-22,5769709	166,748803	1
0	1	1	-22,5760213	166,747317	1
1	1	0	-22,5760132	166,747314	1
1	1	0	-22,5759937	166,747238	1
1	1	1	-22,5759339	166,746997	1
1	1	0	-22,5759482	166,746914	1
0	1	1	-22,575827	166,746178	1
0	1	1	-22,5761414	166,74545	1
1	0	0	-22,576704	166,745159	1
1	0	0	-22,5772135	166,745568	1
0	1	1	-22,602428	166,827015	1
0	0	0	-22,6782761	166,809958	1
0	1	1	-22,6795784	166,810474	1

1	1	1	-22,6800334	166,810964	1
0	0	0	-22,6801347	166,811107	1
1	0	1	-22,6804525	166,812336	1
1	1	1	-22,6804516	166,812432	1
1	1	1	-22,680417	166,812545	1
0	1	1	-22,6798452	166,812942	1
1	1	1	-22,6796429	166,812971	1
1	1	1	-22,7097554	166,796225	1
1	1	0	-22,7096409	166,79457	1
1	1	1	-22,7097094	166,794528	1
1	1	1	-22,7108956	166,797127	1
0	0	0	nd	nd	1
0	0	0	-22,6918769	166,850724	1
0	1	1	-22,6923644	166,851431	1
1	1	1	-22,6926773	166,851594	1
1	1	1	-22,6926127	166,849956	1
0	1	1	-22,6923675	166,850068	1
0	1	1	-22,6922522	166,851321	1
0	0	0	nd	nd	1
0	0	0	nd	nd	1
1	0	0	nd	nd	1
0	1	1	nd	nd	1
0	0	0	nd	nd	1
0	0	0	nd	nd	1
0	1	1	nd	nd	1
0	0	0	nd	nd	1
0	1	0	nd	nd	1
0	1	1	nd	nd	1
0	1	1	nd	nd	1
1	0	0	nd	nd	1
0	0	0	nd	nd	1
0	0	0	nd	nd	1
0	1	1	-22,692208	166,851313	1
1	1	1	-22,5201286	166,556328	1
0	0	0	-22,5203703	166,555971	1
0	1	1	-22,520437	166,555922	1
0	0	0	-22,5204469	166,555968	1
0	0	0	-22,5208041	166,555993	1
0	0	0	-22,52124	166,556501	1
0	1	1	-22,5212036	166,556516	1
0	1	1	-22,5212151	166,556668	1
1	1	1	-22,5212589	166,556762	1
1	0	0	-22,521289	166,556821	1
0	0	0	-22,5211844	166,557351	1
0	1	1	-22,5211613	166,557498	1
0	0	0	-22,5209792	166,557692	1
1	0	0	-22,5205166	166,557212	1
1	0	0	-22,5203063	166,556842	1
0	0	0	-22,5202566	166,556742	1
0	0	0	nd	nd	1

1	0	0	-22,6022008	166,827435	1
1	0	0	-22,677825	166,809945	1
0	0	0	-22,6776481	166,809981	1
1	1	1	-22,6794033	166,81038	1
0	1	1	-22,6790844	166,810199	1
0	0	0	nd	nd	1
1	1	1	-22,692815	166,851588	1
1	1	1	-22,6939054	166,851319	1
0	1	0	-22,7205947	166,850744	1
0	0	0	nd	nd	1
1	1	1	-22,8457046	166,877017	1
1	1	1	-22,845628	166,877071	1
0	1	1	-22,8459704	166,876907	1
0	0	0	-22,7784762	166,802014	1
0	0	0	-22,7753042	166,799636	1
0	0	0	-22,5758899	166,746407	1
1	1	1	-22,5759036	166,746664	1
1	0	0	-22,5202265	166,556448	1
0	0	0	-22,5203982	166,555971	1
0	0	0	-22,5212002	166,556445	1
0	0	0	-22,5211504	166,55645	1
0	0	0	-22,5211861	166,556604	1
1	0	0	-22,5212906	166,556826	1
0	0	0	-22,5212734	166,556832	1
0	0	0	-22,5210821	166,557502	1
0	0	0	-22,5210795	166,557651	1
1	0	0	-22,5205861	166,557211	1
0	1	0	-22,520565	166,555867	1
1	1	1	-22,52035	166,556845	1
1	0	0	-22,576033	166,747202	1
0	1	1	-22,575922	166,746851	1
1	0	0	-22,575834	166,74627	1
0	0	0	nd	nd	1
0	0	0	nd	nd	1
0	0	0	nd	nd	1
0	0	0	nd	nd	1
1	1	1	-22,692299	166,850135	1
0	1	1	-22,69244	166,850099	1
0	1	1	-22,691928	166,850599	1
1	1	1	-22,691923	166,850867	1
1	1	1	-22,69403	166,851343	1
0	0	0	-22,721455	166,850494	1
0	1	1	-22,723817	166,85279	1
1	1	1	-22,723371	166,852658	1
0	1	1	-22,721492	166,851607	1
0	0	0	-22,721094	166,851432	1
1	1	1	-22,720935	166,851335	1
0	0	0	nd	nd	1
0	0	0	nd	nd	1
0	0	0	nd	nd	1

0	0	0	nd	nd	1
0	1	1	-22,575952	166,745696	1
1	1	1	-22,576128	166,745431	1
0	1	1	-22,576724	166,745171	1
0	1	1	-22,577089	166,745343	1
1	1	1	-22,577224	166,745667	1
1	1	1	-22,577629	166,749181	1
0	1	1	-22,577434	166,749245	1
0	1	1	-22,577149	166,748938	1
1	1	0	-22,576004	166,747341	1
0	1	1	-22,575878	166,74632	1
1	1	1	-22,678552	166,809997	1
0	1	1	-22,679256	166,810237	1
1	1	1	-22,679256	166,810237	1
0	1	1	-22,68013	166,811152	1
0	0	0	-22,680057	166,811074	1
0	1	1	-22,68035	166,81178	1
0	1	1	-22,680392	166,812335	1
1	1	1	-22,680294	166,812561	1
1	1	1	-22,680338	166,812519	1
1	1	1	-22,67962	166,812949	1
1	1	1	-22,679405	166,812908	1
1	1	1	-22,679025	166,812765	1
0	1	1	-22,677583	166,810477	1
0	1	1	-22,677549	166,810308	1
1	0	0	-22,677538	166,809994	1
1	0	0	-22,677567	166,809987	1
1	1	0	-22,720894	166,850633	1
1	0	0	-22,720991	166,85066	1
1	1	1	-22,721104	166,85056	1
0	0	0	-22,721283	166,850532	1
1	0	0	-22,721402	166,850506	1
0	0	0	-22,723844	166,851621	1
1	1	1	-22,723936	166,852382	1
1	1	1	-22,723886	166,852729	1
0	1	1	-22,723501	166,852688	1
1	1	1	-22,721577	166,851708	1
1	0	0	-22,721481	166,851637	1
1	1	1	-22,721321	166,851556	1
1	0	0	-22,721059	166,851418	1
0	1	1	-22,720927	166,851363	1
0	1	0	-22,7203	166,851115	1
0	0	0	-22,720343	166,851081	1
0	1	1	-22,720219	166,850998	1
0	1	1	-22,775824	166,799737	1
1	1	1	-22,776365	166,802482	1
1	0	0	-22,77551	166,799941	1
0	1	1	-22,845754	166,87877	1
0	1	1	-22,845404	166,877924	1
1	0	0	-22,845381	166,877687	1

0	1	1	-22,845516	166,877173	1
1	1	1	-22,845628	166,87705	1
1	0	0	-22,846241	166,876845	1
0	0	0	nd	nd	1
0	1	1	-22,691916	166,850688	1
0	1	1	-22,692256	166,851393	1
0	0	0	-22,693972	166,851334	1
0	0	0	-22,694083	166,85132	1
0	0	0	-22,694455	166,850988	1
0	1	0	-22,693676	166,849779	1
0	0	0	-22,692897	166,849901	1
1	1	1	-22,692812	166,849902	1
1	1	1	-22,692706	166,849962	1
0	1	1	-22,692584	166,850003	1
1	1	1	-22,692554	166,84999	1
1	1	1	-22,692509	166,850034	1
0	1	1	-22,692506	166,850101	1
1	1	1	-22,69245	166,85013	1
0	1	1	-22,692362	166,850106	1
1	1	1	-22,69212	166,850226	1
1	1	1	-22,69202	166,850364	1
0	1	1	-22,691967	166,85044	1
0	1	1	-22,691911	166,850519	1
0	0	0	nd	nd	1
0	1	1	-22,708929	166,80989	1
0	1	1	-22,709528	166,794978	1
0	1	1	-22,709449	166,794823	1
0	1	1	-22,605056	166,826551	1
1	1	1	-22,605033	166,826553	1
0	1	1	-22,605033	166,826553	1
0	0	0	-22,520252	166,556837	1
1	1	1	-22,520252	166,556837	1
1	0	0	-22,520252	166,556837	1
0	1	1	-22,520252	166,556837	1
0	0	0	-22,52113	166,556797	1
0	1	1	-22,52113	166,556797	1
0	1	0	-22,52113	166,556797	1
1	1	1	-22,52113	166,556797	1
0	1	1	-22,517335	166,611061	1
0	0	0	-22,513902	166,611252	1
0	0	0	-22,51404	166,611032	1
0	1	1	-22,514215	166,610648	1
1	0	0	-22,422627	167,0142	0
0	1	1	-22,422355	167,013803	0
1	1	1	-22,423066	167,01293	0
0	0	0	nd	nd	0
0	0	0	nd	nd	0
0	1	1	-22,528734	166,827098	0
0	0	0	nd	nd	0
1	0	0	-22,549301	166,792789	0

0	0	0	-22,551614	166,791493	0
0	0	0	-22,551674	166,791252	0
1	0	0	-22,551633	166,791107	0
0	1	1	-22,709448	166,794923	0
1	0	0	-22,721794	166,917229	0
0	1	1	-22,721756	166,917285	0
0	1	1	-22,721699	166,917597	0
0	1	0	-22,721689	166,917604	0
1	1	1	-22,721722	166,917697	0
1	0	0	-22,721797	166,917867	0
0	0	0	-22,722026	166,918065	0
0	0	0	-22,722172	166,91802	0
1	0	0	-22,72231	166,917981	0
1	1	1	-22,722549	166,91793	0
0	0	0	-22,683611	166,97049	0
0	1	1	-22,683651	166,967508	0
0	0	0	-22,719776	166,973902	0
0	0	0	-22,720016	166,973656	0
0	0	0	-22,720127	166,97352	0
0	0	0	-22,720167	166,973474	0
0	1	1	-22,720296	166,973366	0
1	0	0	-22,720364	166,973279	0
1	0	0	-22,720411	166,97317	0
1	0	0	-22,720624	166,972971	0
0	0	0	-22,803169	166,915879	0
1	0	0	-22,858456	166,944145	0
0	0	0	-22,516253	166,769752	0
1	1	1	-22,516159	166,76965	1
0	0	0	-22,520528	166,555867	1
1	0	0	-22,52066	166,555937	1
0	0	0	-22,521233	166,55678	1
0	1	1	-22,520833	166,557646	1
1	1	1	-22,576085	166,745587	1
1	1	1	-22,576147	166,745432	1
1	1	1	-22,576875	166,745198	1
1	1	1	-22,577212	166,745625	1
1	1	1	-22,577242	166,745691	1
1	0	0	-22,577212	166,749002	1
1	0	0	-22,576043	166,747326	1
1	0	0	-22,575953	166,747075	1
1	1	1	nd	nd	1
0	1	1	-22,708769	166,809967	1
0	1	1	-22,677643	166,80996	1
1	0	0	-22,677744	166,809991	1
0	0	0	-22,680015	166,810987	1
0	0	0	-22,680409	166,812291	1
1	0	0	-22,679994	166,812809	1
0	1	1	-22,679597	166,81296	1
0	0	0	-22,677575	166,810545	1
1	0	0	-22,677566	166,810383	1

0	0	0	-22,677544	166,81037	1
0	0	0	-22,677584	166,80999	1
1	0	0	-22,77841	166,802347	1
0	0	0	-22,776794	166,802698	1
0	0	0	-22,778417	166,801909	1
1	1	1	-22,846797	166,879196	1
1	1	1	-22,84658	166,879189	1
0	1	1	-22,845586	166,878403	1
0	1	1	-22,692015	166,850343	1
0	1	1	-22,691974	166,850378	1
0	0	0	-22,69209	166,850232	1
1	1	1	-22,692	166,850376	1
1	1	1	-22,692692	166,849963	1
0	1	1	-22,693021	166,849865	1
0	1	1	-22,693294	166,84982	1
0	0	0	-22,693476	166,849791	1
1	1	1	-22,693781	166,849717	1
0	0	0	-22,694013	166,851278	1
0	0	0	-22,693929	166,851296	1
1	0	0	-22,693755	166,851401	1
1	1	1	-22,692649	166,851586	1
0	0	0	-22,692602	166,851499	1
0	1	1	-22,692087	166,851193	1
0	1	1	-22,691879	166,850866	1
0	1	1	-22,720187	166,850868	1
0	1	1	-22,720461	166,850798	1
0	1	1	-22,720713	166,850708	1
1	0	0	-22,721016	166,850623	1
1	1	1	-22,721087	166,850604	1
0	1	1	-22,721566	166,850441	1
1	1	1	-22,723853	166,851727	1
1	1	0	-22,72391	166,852425	1
1	0	0	-22,721357	166,851593	1
0	0	0	-22,720816	166,851314	1
1	0	0	-22,720693	166,851255	1
1	0	0	-22,720606	166,851214	1
0	0	0	-22,720487	166,85117	1
0	0	0	-22,720143	166,851017	1
0	1	1	-22,709434	166,794899	1
0	1	1	-22,709379	166,795255	1
1	1	1	-22,709394	166,795023	1
1	1	1	-22,710124	166,794225	1
1	1	1	-22,710898	166,794156	1
1	1	1	-22,711026	166,796787	1
1	1	1	-22,711003	166,796835	1
0	0	0	-22,605032	166,826555	1
1	1	1	-22,515327	166,612635	1
0	1	1	-22,514968	166,612469	1
0	0	0	-22,513958	166,611663	1
0	0	0	-22,514324	166,610628	1

0	0	0	nd	nd	1
1	0	0	-22,721151	166,851434	1
0	0	0	-22,721283	166,850532	1
0	0	0	nd	nd	0
0	0	0	nd	nd	0
0	0	0	nd	nd	0
0	1	1	-22,531389	166,828772	0
0	1	1	-22,53257	166,828121	0
0	1	1	-22,539928	166,821188	0
0	1	1	-22,540397	166,821647	0
1	1	1	-22,549207	166,792664	0
0	0	0	-22,549211	166,792717	0
1	1	1	-22,549236	166,792785	0
1	1	1	-22,551598	166,791081	0
1	1	1	-22,692257	166,851486	0
1	1	1	-22,692257	166,851486	0
1	1	1	-22,684565	166,968114	0
1	1	1	-22,684774	166,966445	0
0	1	1	-22,684775	166,96615	0
1	1	1	-22,720188	166,973497	0
1	1	1	-22,720006	166,973529	0
1	1	1	-22,720628	166,974147	0
0	1	1	-22,720909	166,972816	0
0	0	0	nd	nd	0
0	1	1	-22,858627	166,944015	0
0	0	0	-22,516244	166,768982	0
0	0	0	-22,516224	166,769734	0
0	1	1	-22,517896	166,769836	0
0	0	0	-22,521121	166,556374	1
0	1	1	-22,521152	166,556399	1
0	0	0	-22,521177	166,556563	1
1	0	0	-22,521228	166,556572	1
0	0	0	-22,521235	166,556653	1
1	0	0	-22,521247	166,556869	1
0	0	0	-22,521255	166,556882	1
1	0	0	-22,521263	166,557013	1
0	1	1	-22,520811	166,557638	1
1	0	0	-22,520497	166,557132	1
0	1	1	-22,52044	166,55704	1
0	1	1	-22,576772	166,745135	1
0	1	1	-22,576967	166,745292	1
1	1	1	-22,577198	166,745621	1
0	0	0	-22,577576	166,749269	1
0	1	1	-22,577399	166,749245	1
1	1	1	-22,5773	166,749066	1
1	0	0	-22,576025	166,747228	1
1	1	1	-22,575962	166,745815	1
1	0	0	-22,576444	166,745225	1
0	1	1	-22,678965	166,810112	1
0	1	1	-22,679862	166,810779	1

0	0	0	-22,679978	166,810946	1
1	0	0	-22,680344	166,811821	1
0	0	0	-22,68039	166,812072	1
0	1	1	-22,680416	166,812344	1
1	1	1	-22,680381	166,812361	1
1	1	1	-22,680312	166,812554	1
1	1	1	-22,680281	166,812565	1
1	1	1	-22,680007	166,8128	1
1	0	0	-22,677532	166,810305	1
0	0	0	-22,777452	166,802792	1
0	0	0	-22,776157	166,800013	1
1	0	0	-22,77792	166,801356	1
1	1	1	-22,84653	166,879169	1
0	1	0	-22,846189	166,879074	1
0	0	0	-22,845442	166,87802	1
0	1	1	-22,845894	166,876963	1
0	0	0	nd	nd	1
0	0	0	-22,69219	166,850208	1
0	0	0	-22,691934	166,850469	1
1	0	0	-22,691895	166,851003	1
0	1	1	-22,691947	166,851102	1
0	0	0	-22,69249	166,851455	1
0	0	0	-22,693722	166,851404	1
0	0	0	-22,694307	166,851197	1
0	1	1	-22,692589	166,849994	1
0	1	1	-22,692519	166,85002	1
1	0	0	-22,692484	166,85006	1
0	0	0	-22,692408	166,850108	1
0	1	1	-22,692345	166,850098	1
0	0	0	-22,720141	166,851013	1
0	1	1	-22,720301	166,850923	1
1	1	1	-22,720755	166,850715	1
0	0	0	-22,721037	166,850638	1
1	0	0	-22,723841	166,851722	1
0	0	0	-22,723906	166,852346	1
0	0	0	-22,721037	166,850638	1
1	0	0	-22,723858	166,852722	1
0	0	0	-22,723545	166,852715	1
1	1	1	-22,720182	166,851028	1
0	0	0	-22,720174	166,85105	1
0	1	1	-22,720204	166,851043	1
0	0	0	-22,721037	166,850638	1
1	0	0	-22,720604	166,851217	1
0	1	1	-22,720661	166,851245	1
0	1	1	-22,720787	166,851324	1
0	1	1	-22,721067	166,851434	1
0	1	1	-22,721372	166,851588	1
1	1	1	-22,709613	166,796062	1
0	0	0	-22,709398	166,794962	1
0	0	0	-22,709428	166,794869	1

1	1	1	-22,70991	166,794319	1
1	1	1	-22,710433	166,794136	1
1	1	1	-22,710975	166,796825	1
0	0	0	-22,708642	166,810042	1
0	1	1	-22,602263	166,82732	1
1	0	0	-22,515833	166,612683	1
0	0	0	-22,721037	166,850638	1
0	0	0	-22,514486	166,612217	1
1	0	0	-22,514013	166,611861	1
0	0	0	-22,513885	166,611337	1
0	0	0	-22,514064	166,610913	1
0	0	0	-22,575871	166,746127	1
0	0	0	-22,57592	166,745911	1
0	1	1	-22,57591	166,745895	1
1	1	1	-22,57585	166,745874	1
0	1	1	-22,57599	166,745686	1
0	0	0	-22,576095	166,745442	1
1	1	1	-22,576129	166,745348	1
0	0	0	-22,576434	166,745212	1
1	0	0	-22,57654	166,745171	1
1	1	1	-22,57693	166,745222	1
1	1	1	-22,57709	166,745475	1
0	0	0	-22,577531	166,749254	1
0	1	1	-22,57746	166,749296	1
0	0	0	-22,577421	166,749297	1
1	0	0	-22,577227	166,749012	1
1	0	0	-22,575899	166,746843	1
0	0	0	-22,575874	166,746617	1
0	0	0	-22,575881	166,746362	1
0	0	0	-22,602327	166,827129	1
0	1	1	-22,602359	166,827104	1
0	0	0	-22,677779	166,809969	1
1	0	0	-22,680229	166,811381	1
0	0	0	-22,680452	166,812246	1
1	1	1	-22,68043	166,812311	1
0	0	0	-22,680434	166,812378	1
1	1	1	-22,680093	166,812787	1
0	0	0	-22,679826	166,812959	1
0	0	0	-22,679597	166,812944	1
1	0	0	-22,679053	166,812736	1
0	0	0	-22,677616	166,810476	1
0	0	0	-22,6776	166,810383	1
0	1	1	-22,709384	166,795196	1
1	0	0	-22,710439	166,794138	1
0	0	0	nd	nd	1
0	1	1	-22,72008	166,851021	1
1	0	0	-22,720175	166,851021	1
0	1	1	-22,720409	166,851135	1
0	1	1	-22,720535	166,851204	1
0	0	0	-22,720609	166,851234	1

0	1	1	-22,720748	166,851279	1
0	0	0	-22,720774	166,85127	1
0	0	0	-22,721237	166,851532	1
0	1	1	-22,723288	166,852616	1
0	1	1	-22,72358	166,852742	1
0	1	1	-22,723705	166,852801	1
0	0	0	-22,723773	166,852794	1
0	0	0	-22,723865	166,85277	1
1	0	0	-22,723882	166,85245	1
1	0	0	-22,723895	166,852266	1
1	0	0	-22,721375	166,850503	1
1	1	1	-22,72083	166,850691	1
1	1	1	-22,720709	166,850691	1
1	0	0	-22,72067	166,850724	1
0	1	1	-22,720279	166,850857	1
1	0	0	-22,720169	166,850904	1
1	0	0	-22,69234	166,8502	1
0	1	1	-22,692054	166,850321	1
0	1	1	-22,691945	166,850626	1
1	1	1	-22,691874	166,850843	1
0	1	1	-22,692458	166,851404	1
0	0	0	-22,693768	166,85139	1
0	1	1	-22,694036	166,851287	1
0	0	0	-22,693609	166,849734	1
0	0	0	-22,692678	166,849961	1
0	0	0	-22,692599	166,850012	1
0	1	1	-22,77721	166,80092	1
0	0	0	-22,777866	166,801384	1
1	1	0	-22,778093	166,801581	1
0	0	0	-22,778337	166,801858	1
1	1	1	-22,778234	166,802452	1
1	1	1	-22,77729	166,802808	1
0	0	0	-22,7761	166,800007	1
1	0	0	-22,776286	166,800186	1
0	0	0	nd	nd	1
0	1	1	-22,847146	166,876779	1
0	1	1	-22,846204	166,879097	1
1	1	0	-22,845825	166,878849	1
0	0	0	-22,845416	166,877835	1
0	1	1	-22,845388	166,877343	1
1	1	1	-22,845964	166,876952	1
0	0	0	-22,514411	166,610288	1
1	1	1	-22,513945	166,611735	1
0	1	1	-22,513855	166,611483	1
1	1	1	-22,5202	166,556295	1
1	0	0	-22,520372	166,556912	1
1	1	1	-22,520266	166,556816	1
0	0	0	-22,520526	166,55715	1
0	1	1	-22,520857	166,557928	1
1	1	1	-22,521125	166,557592	1

1	1	1	-22,521247	166,556816	1
0	1	1	-22,521173	166,556532	1
0	0	0	-22,521179	166,556525	1
0	1	1	-22,521185	166,55648	1
0	0	0	-22,521113	166,556342	1
0	0	0	-22,521021	166,55625	1
0	0	0	-22,521002	166,556215	1
0	0	0	-22,520509	166,555899	1
0	1	0	-22,520403	166,555909	1
0	0	0	-22,520432	166,555926	1
1	0	0	-22,520874	166,556069	1
0	0	0	-22,520922	166,556177	1
0	0	0	-22,521078	166,556411	1
1	1	1	-22,521162	166,556624	1
0	0	0	-22,521135	166,55669	1
1	1	1	-22,521211	166,556911	1
0	0	0	-22,520903	166,55769	1
1	0	0	-22,520576	166,557318	1
1	0	0	-22,576096	166,745528	1
0	0	0	nd	nd	1
0	0	0	nd	nd	1
0	0	0	nd	nd	1
0	0	0	nd	nd	1
0	0	0	-22,723911	166,852454	1
0	0	0	-22,723776	166,852781	1
0	1	1	-22,723573	166,852728	1
0	0	0	nd	nd	1
0	0	0	nd	nd	1
0	1	1	-22,707263	166,813059	1
0	0	0	-22,691919	166,851002	1
0	0	0	-22,692013	166,851142	1
0	0	0	-22,692451	166,851409	1
1	0	0	-22,692773	166,851565	1
0	0	0	-22,693416	166,851514	1
0	0	0	-22,693561	166,851513	1
0	0	0	-22,693767	166,851388	1
1	0	0	-22,693903	166,851347	1
0	0	0	-22,693952	166,851351	1
0	0	0	nd	nd	1
0	1	1	-22,720103	166,851016	1
0	1	1	-22,720178	166,85107	1
0	0	0	nd	nd	1
0	0	0	nd	nd	1
0	0	0	nd	nd	1
0	0	0	nd	nd	1
0	0	0	nd	nd	1
0	0	0	-22,693986	166,85135	1
0	0	0	-22,69381	166,851381	1
0	0	0	-22,693596	166,851524	1
0	0	0	-22,721914	166,850327	1

0	0	0	-22,723778	166,852779	1
0	0	0	-22,723582	166,852719	1
0	0	0	-22,720745	166,851296	1
0	0	0	-22,720401	166,851126	1
0	0	0	nd	nd	1
0	0	0	nd	nd	1
0	0	0	nd	nd	1
0	0	0	-22,577437	166,74925	1
0	0	0	nd	nd	1
0	0	0	nd	nd	1
1	1	1	-22,520462	166,555868	1
0	1	1	-22,52126	166,556842	1
1	1	1	-22,521182	166,557378	1
1	1	1	-22,520674	166,557553	1
0	0	0	nd	nd	1
0	1	1	-22,577458	166,749308	1
0	1	1	-22,577458	166,749381	1
0	0	0	nd	nd	1
1	1	1	-22,847024	166,876714	1
1	1	1	-22,847342	166,877033	1
0	1	1	-22,845515	166,878351	1
1	1	1	-22,845425	166,877945	1
1	1	0	-22,845365	166,877657	1
0	1	0	-22,845399	166,877378	1
0	0	0	-22,775356	166,799497	1
0	0	0	-22,720291	166,85096	1
0	0	0	-22,721092	166,851442	1
1	0	0	-22,720921	166,851369	1
1	0	0	-22,678534	166,809763	1
0	0	0	-22,6802	166,812715	1
1	1	1	-22,678856	166,812676	1
0	0	0	-22,709471	166,794787	1
1	1	1	-22,711317	166,794732	1
0	0	0	-22,711311	166,794695	1
1	0	1	-22,711185	166,796537	1
0	1	1	-22,710999	166,796817	1
0	1	1	-22,71092	166,797066	1
1	1	1	-22,710903	166,797107	1
1	1	1	-22,71087	166,797188	1
0	0	0	-22,707516	166,811903	1
0	0	0	-22,692619	166,849978	1
0	0	0	-22,693636	166,849766	1
0	1	1	-22,602204	166,827473	1
1	1	1	-22,602305	166,82721	1
0	1	0	-22,604836	166,826313	1
0	0	0	-22,602203	166,828005	1
1	0	1	-22,37815	167,062104	0
0	0	0	-22,375909	167,065498	0
0	1	1	-22,375677	167,065528	0
1	0	0	-22,375584	167,065571	0

0	0	0	-22,375522	167,065572	0
0	1	1	-22,375471	167,065578	0
1	0	0	-22,375377	167,064606	0
0	0	0	-22,444461	167,094316	0
1	0	0	-22,444642	167,094094	0
0	0	0	-22,444678	167,094065	0
0	0	1	-22,444101	167,098446	0
0	0	0	-22,443799	167,098432	0
0	0	0	-22,443633	167,098349	0
0	0	0	nd	nd	0
0	1	1	-22,576069	166,745509	1
0	1	1	-22,576848	166,745105	1
0	1	1	-22,577229	166,745593	1
1	1	1	-22,577693	166,749066	1
1	1	1	-22,576474	166,748223	1
0	0	0	-22,575887	166,746856	1
0	1	1	-22,575893	166,746713	1
0	1	1	-22,575896	166,74665	1
1	1	1	-22,575845	166,746639	1
1	1	1	-22,709814	166,796375	1
1	1	1	-22,709381	166,795437	1
1	1	0	-22,709568	166,7946	1
1	1	0	-22,709727	166,794433	1
1	1	1	-22,710251	166,794163	1
1	1	1	-22,710961	166,796877	1
0	1	1	-22,710805	166,797293	1
1	1	1	-22,710807	166,797323	1
1	1	1	-22,778193	166,802562	1
0	1	1	-22,777726	166,80279	1
0	1	1	-22,775323	166,799884	1
0	1	1	-22,775837	166,799782	1
0	1	1	-22,775996	166,799881	1
0	0	0	-22,776113	166,799989	1
1	1	1	-22,776263	166,800137	1
0	0	0	-22,77791	166,80137	1
1	1	1	-22,778268	166,80175	1
1	0	0	-22,779007	166,802498	1
1	0	0	-22,84712	166,876784	1
0	0	0	-22,847125	166,879149	1
1	1	1	-22,846993	166,879153	1
1	1	1	-22,846915	166,879168	1
1	1	1	-22,846108	166,879097	1
1	1	1	-22,845825	166,878874	1
1	1	1	-22,845792	166,87881	1
1	1	1	-22,845622	166,878596	1
1	1	1	-22,845571	166,878451	1
0	0	0	-22,845438	166,877488	1
1	1	1	-22,84539	166,877425	1
1	1	1	-22,84541	166,877339	1
0	1	1	-22,845497	166,87723	1

0	0	0	-22,845595	166,877163	1
1	1	1	-22,845621	166,877163	1
1	1	1	-22,845831	166,877013	1
1	1	1	-22,846673	166,876813	1
0	1	1	-22,692479	166,850075	1
1	0	0	-22,69349	166,849795	1
0	0	0	-22,693891	166,85138	1
1	1	1	-22,693556	166,851496	1
0	0	0	-22,693024	166,851629	1
1	1	1	-22,692318	166,851355	1
0	0	0	-22,692008	166,850423	1
1	1	1	-22,692263	166,850206	1
1	1	0	-22,720336	166,850916	1
0	0	0	-22,721315	166,850521	1
1	1	1	-22,709085	166,810152	1
0	0	0	-22,706733	166,812801	1
1	1	1	-22,707295	166,812215	1
0	1	0	-22,708267	166,810928	1
1	0	0	-22,708316	166,810853	1
1	0	0	-22,70833	166,810771	1
0	0	0	-22,708401	166,810537	1
1	0	0	-22,708511	166,810274	1
1	1	0	-22,708764	166,809971	1
0	0	1	-22,602462	166,826957	1
1	1	1	-22,604775	166,826322	1
0	1	1	-22,605025	166,82639	1
0	0	0	-22,603373	166,828067	1
1	0	0	-22,602683	166,828179	1
1	0	0	-22,60264	166,828197	1
0	0	1	-22,60239	166,828135	1
0	0	1	-22,602216	166,827991	1
1	0	0	-22,516224	166,769891	0
0	1	1	-22,684418	166,969049	0
1	1	0	-22,684463	166,968501	0
1	0	0	-22,684395	166,966805	0
0	0	0	-22,684043	166,967122	0
0	1	1	-22,683394	166,967766	0
0	0	0	-22,682969	166,96832	0
1	0	0	-22,682984	166,968602	0
0	0	0	-22,720746	166,97285	0
1	1	1	-22,721044	166,973072	0
1	1	1	-22,721097	166,973429	0
1	1	1	-22,72084	166,973965	0
0	0	0	-22,720711	166,97409	0
1	1	1	-22,720537	166,974211	0
0	1	1	-22,720471	166,974232	0
1	0	0	-22,720223	166,974321	0
1	0	0	-22,719896	166,973768	0
1	0	0	-22,719916	166,973709	0
0	0	0	-22,719964	166,973685	0

0	0	0	-22,720031	166,973609	0
1	0	0	-22,720355	166,973279	0
0	0	0	-22,72037	166,973299	0
0	0	0	nd	nd	0
0	1	1	-22,803394	166,916509	0
0	0	0	-22,803157	166,915808	0
1	0	0	-22,802502	166,916481	0
0	0	0	-22,858165	166,943767	0
0	0	0	nd	nd	1
0	0	0	-22,679241	166,810205	1
1	1	1	-22,679466	166,810332	1
1	0	0	-22,680138	166,811126	1
0	0	0	-22,680243	166,811397	1
1	0	0	-22,68025	166,81142	1
0	1	1	-22,680395	166,811875	1
1	1	1	-22,680414	166,811957	1
1	1	1	-22,680423	166,812086	1
1	1	1	-22,680308	166,812559	1
1	0	0	-22,680276	166,812607	1
0	1	1	-22,680156	166,81271	1
1	1	1	-22,679716	166,812929	1
1	1	1	-22,679186	166,812813	1
1	1	0	-22,677598	166,810564	1
1	1	1	-22,677531	166,810071	1
1	1	1	-22,67758	166,809975	1
0	0	0	nd	nd	0
0	0	0	nd	nd	0
0	0	0	-22,532908	166,828355	0
0	0	0	-22,532934	166,828394	0
0	0	0	-22,532424	166,831636	0
0	0	0	-22,53217	166,83189	0
0	0	0	-22,532114	166,831917	0
0	0	0	nd	nd	0
0	0	0	-22,602201	166,827446	1
1	0	0	-22,604758	166,826313	1
0	0	0	-22,603807	166,827913	1
1	1	1	-22,603673	166,827968	1
1	1	1	-22,603248	166,828066	1
1	1	1	-22,602538	166,828184	1
1	0	0	-22,777821	166,801306	1
0	0	0	-22,777069	166,800746	1
0	0	0	-22,775851	166,799768	1
0	0	0	-22,845536	166,877104	1
0	0	0	-22,845389	166,877254	1
0	1	1	-22,845329	166,877377	1
1	1	1	-22,845314	166,877505	1
0	1	1	-22,845362	166,87791	1
1	1	1	-22,845373	166,87799	1
1	1	1	-22,84553	166,878443	1
1	1	1	-22,845803	166,878797	1

0	0	0	-22,846107	166,879048	1
1	1	1	-22,846243	166,879114	1
1	0	0	-22,846406	166,879146	1
1	0	0	-22,846534	166,879165	1
1	0	0	-22,847645	166,878517	1
1	1	1	-22,84736	166,877139	1
0	0	0	-22,847108	166,876643	1
1	1	1	-22,846508	166,876699	1
1	1	0	-22,576751	166,745191	1
1	0	1	-22,576902	166,745191	1
0	1	0	-22,577239	166,745581	1
0	0	0	-22,577338	166,745828	1
0	0	0	-22,577323	166,745804	1
1	0	0	-22,577302	166,745749	1
1	1	1	-22,577068	166,748841	1
1	0	0	-22,576895	166,748647	1
0	0	0	-22,576111	166,747619	1
1	1	0	-22,575915	166,746075	1
1	0	0	-22,521241	166,556814	1
0	0	0	-22,521263	166,556828	1
0	0	0	-22,521271	166,556988	1
0	0	0	-22,521246	166,55704	1
0	0	0	-22,521222	166,557043	1
0	0	0	-22,521265	166,557167	1
0	0	0	-22,521173	166,557359	1
1	0	0	-22,52117	166,557467	1
1	1	1	-22,521119	166,557637	1
0	1	1	-22,520683	166,557806	1
1	1	1	-22,520548	166,557322	1
0	0	0	-22,520237	166,556732	1
0	0	0	-22,520188	166,556663	1
0	1	1	-22,378077	167,061141	0
0	0	0	-22,377828	167,064503	0
1	1	1	-22,377743	167,064577	0
0	0	0	-22,376186	167,065416	0
1	1	1	-22,376062	167,065451	0
0	0	0	-22,375814	167,065539	0
0	1	1	-22,375751	167,065551	0
1	1	0	-22,375331	167,065589	0
0	1	1	-22,375197	167,065342	0
0	1	1	-22,37523	167,065201	0
0	1	1	-22,375274	167,065045	0
0	0	0	-22,3753	167,064773	0
0	0	0	-22,375713	167,064167	0
0	0	0	-22,444942	167,093785	0
1	1	0	-22,444044	167,098423	0
1	0	0	-22,443862	167,098447	0
1	0	0	-22,443588	167,098335	0
1	1	1	-22,443474	167,098254	0
0	0	0	-22,443217	167,097955	0

0	0	0	nd	nd	0
0	0	0	nd	nd	0
0	1	1	-22,684814	166,966655	0
0	1	1	-22,684818	166,966569	0
0	0	0	-22,684652	166,96619	0
0	1	0	-22,684028	166,967127	0
0	0	0	-22,683981	166,967175	0
0	0	0	-22,683826	166,967292	0
0	1	1	-22,683349	166,967551	0
0	1	1	-22,683338	166,967564	0
0	0	0	-22,68322	166,969325	0
0	1	1	-22,72065	166,972948	0
0	1	1	-22,720721	166,972873	0
0	0	0	-22,721108	166,973651	0
0	0	0	-22,720497	166,97426	0
1	0	0	-22,720241	166,974352	0
1	1	1	-22,71986	166,973831	0
1	1	1	-22,719934	166,973725	0
1	1	1	-22,720009	166,973638	0
1	1	1	-22,720067	166,97357	0
0	0	0	-22,720388	166,973315	0
1	1	1	-22,7205	166,973196	0
0	0	0	nd	nd	0
0	1	1	-22,802898	166,916968	0
1	0	0	-22,802591	166,916001	0
0	1	1	-22,858154	166,943839	0
0	0	0	nd	nd	1
0	0	0	-22,691938	166,850779	1
1	0	0	-22,691899	166,850704	1
0	0	0	-22,691864	166,850701	1
1	1	1	-22,692605	166,850012	1
1	1	1	-22,69279	166,849941	1
0	0	0	-22,693954	166,85135	1
0	0	0	-22,691885	166,850998	1
1	0	0	-22,551654	166,791273	0
0	0	0	nd	nd	0
0	0	0	-22,532673	166,828166	0
0	0	0	-22,533106	166,828794	0
0	0	0	nd	nd	0
1	0	0	-22,707843	166,811464	1
0	1	1	-22,70807	166,811204	1
1	1	1	-22,708235	166,810884	1
0	0	0	-22,708242	166,810868	1
1	0	0	-22,708277	166,810749	1
0	1	1	-22,679417	166,810356	1
0	0	0	-22,680332	166,811791	1
0	0	0	-22,68036	166,811943	1
1	0	0	-22,680373	166,811995	1
1	0	0	-22,680401	166,812241	1
1	0	0	-22,680367	166,812558	1

0	0	0	-22,679472	166,81298	1
1	1	1	-22,679151	166,812831	1
1	0	0	-22,679027	166,812806	1
1	0	0	-22,677512	166,810298	1
0	0	0	-22,67761	166,80999	1
0	0	0	-22,720075	166,850977	1
1	1	1	-22,720929	166,851359	1
1	0	0	-22,721169	166,851507	1
0	0	0	-22,721296	166,851571	1
1	0	0	-22,723459	166,852669	1
0	0	0	-22,723754	166,852846	1
0	0	0	-22,720287	166,851109	1
1	1	1	-22,720534	166,850748	1
0	0	0	-22,709426	166,795163	1
1	0	0	-22,7095	166,794681	1
0	0	0	-22,709518	166,794609	1
1	1	0	-22,709571	166,794561	1
0	0	0	-22,709749	166,794373	1
0	1	0	-22,710358	166,794114	1
0	1	0	-22,710418	166,794094	1
1	0	0	-22,710964	166,794183	1
1	1	1	-22,711379	166,794949	1
0	1	1	-22,710998	166,79678	1
1	0	0	-22,604784	166,826355	1
0	0	0	-22,60328	166,82808	1
0	0	0	-22,602206	166,827985	1
0	0	0	-22,691873	166,850845	1
1	1	1	-22,691946	166,851047	1
1	1	1	-22,69205	166,851194	1
1	1	1	-22,692274	166,851618	1
1	1	1	-22,692518	166,851471	1
0	1	1	-22,69263	166,851518	1
1	1	0	-22,692925	166,85166	1
0	0	0	-22,69426	166,851218	1
1	0	0	-22,69265	166,850007	1
0	0	0	-22,691929	166,850676	1
0	0	0	-22,691948	166,850736	1
0	0	0	-22,723773	166,852838	1
1	1	1	-22,72358	166,852765	1
1	0	0	-22,723515	166,852667	1
0	0	0	-22,723012	166,852521	1
0	0	0	-22,72151	166,85171	1
0	1	1	-22,721006	166,851425	1
0	0	0	nd	nd	1
0	1	1	-22,84547	166,87824	1
0	0	0	-22,845426	166,878145	1
0	0	0	-22,845376	166,877538	1
0	0	0	-22,845417	166,87751	1
0	1	1	-22,845464	166,877278	1
1	0	0	-22,845604	166,877162	1

1	0	0	-22,845708	166,877068	1
1	0	0	-22,84578	166,877058	1
0	0	0	-22,845828	166,877038	1
1	0	0	-22,846769	166,876771	1
0	0	0	-22,847051	166,876709	1
0	0	0	-22,847333	166,87708	1
1	1	1	-22,847266	166,879121	1
1	1	1	-22,847066	166,879171	1
1	1	1	-22,84649	166,87924	1
1	1	1	-22,84628	166,879167	1
0	1	1	-22,845817	166,87887	1
1	0	0	-22,845524	166,878336	1
1	1	1	-22,777932	166,802694	1
1	1	1	-22,77844	166,802337	1
0	0	0	-22,777193	166,800819	1
0	0	0	-22,776356	166,800245	1
0	0	0	-22,776064	166,800002	1
1	0	0	-22,775321	166,799957	1
1	0	0	-22,775317	166,799906	1
1	1	1	-22,7755	166,800316	1
0	1	1	-22,709343	166,795078	1
1	1	1	-22,709508	166,794677	1
0	0	0	-22,709578	166,794559	1
1	0	0	-22,679384	166,810383	1
0	0	0	-22,680202	166,811463	1
0	0	0	-22,680386	166,811971	1
0	0	0	-22,575839	166,74636	1
1	0	0	-22,575855	166,746256	1
0	0	0	-22,575933	166,74586	1
0	0	0	-22,575957	166,745823	1
1	0	0	-22,576048	166,745607	1
1	0	0	-22,577616	166,749144	1
0	1	1	-22,577539	166,749316	1
0	0	0	-22,577379	166,749166	1
1	0	0	-22,576	166,747174	1
1	0	0	-22,575913	166,746826	1
0	0	0	-22,575846	166,746557	1
0	0	0	-22,514076	166,610855	1
0	0	0	-22,514103	166,610814	1
1	0	0	-22,514473	166,610343	1
1	0	0	-22,517379	166,611168	1
1	0	0	-22,51736	166,611286	1
1	1	1	nd	nd	1
0	0	0	-22,514096	166,611837	1
1	1	1	-22,520751	166,557602	1
1	0	0	-22,520707	166,557578	1
1	0	0	-22,520668	166,557469	1
1	1	1	-22,520618	166,557495	1
0	0	0	-22,520614	166,557302	1
0	1	1	-22,520288	166,556697	1

1	1	1	-22,521245	166,556536	1
0	1	1	-22,521311	166,556764	1
0	1	1	-22,521328	166,556819	1
1	1	1	-22,521313	166,556968	1
0	1	1	-22,521196	166,557352	1
0	1	1	-22,521223	166,557551	1
0	0	0	-22,521195	166,557613	1
0	0	0	-22,52089	166,557908	1
0	0	0	-22,520748	166,557752	1
0	0	0	-22,60234	166,827129	1
1	0	0	-22,60243	166,826917	1
1	1	1	-22,679779	166,812924	1
0	0	0	-22,680202	166,811463	1
1	0	0	-22,709573	166,794685	1
0	0	0	-22,709533	166,794805	1
0	0	0	-22,709729	166,796082	1
0	1	1	-22,720559	166,850831	1
0	0	0	-22,723638	166,852719	1
0	1	1	-22,789422	166,847825	1
1	1	1	-22,789288	166,847372	1
0	1	0	-22,845725	166,878737	1
1	1	1	-22,846403	166,879164	1
1	0	0	-22,8473	166,877102	1
1	1	1	-22,847272	166,877019	1
0	0	0	-22,846577	166,876801	1
1	0	0	-22,846508	166,876805	1
0	1	1	-22,845735	166,877052	1
0	1	1	-22,692715	166,851528	1
0	0	0	nd	nd	1
0	0	0	nd	nd	1
0	0	0	-22,575939	166,745973	1
1	1	1	-22,577706	166,748973	1
0	1	1	-22,577515	166,74942	1
0	1	1	-22,577363	166,749196	1
1	0	0	-22,577328	166,749079	1
0	1	1	-22,577282	166,749022	1
0	1	1	-22,520526	166,557203	1
1	0	0	-22,521321	166,556743	1
0	0	0	-22,521319	166,556882	1
0	0	0	nd	nd	1
1	1	1	-22,680469	166,812295	1
0	0	0	-22,720123	166,850945	1
0	0	0	-22,788055	166,848188	1
0	0	0	-22,846013	166,878969	1
0	0	0	-22,846685	166,876806	1
0	0	0	nd	nd	1
0	0	0	nd	nd	1
0	0	0	-22,692715	166,851528	1
0	0	0	nd	nd	1
