# Supplementary material for: Innovative monitoring scheme adapted to remote, scattered nesting aggregation reveals a major loggerhead turtle rookery in New Caledonia, South Pacific
Source: PLoS One. 2024 Jun 18;19(6):e0299748. doi: 10.1371/journal.pone.0299748 (PMC11185463; doi:10.1371/journal.pone.0299748)
Supplement: S3 File — (PDF) [file pone.0299748.s003.pdf]

season	date	mission	islet	nest_tag		discovery_dat	activity_moda
2019_2020	04/12/2019	2	atire		2	27/11/2019	successful nes
2019_2020	05/12/2019	2	uaterembi		16	27/11/2019	successful nes
2019_2020	05/12/2019	2	uaterembi		35	27/11/2019	successful nes
2019_2020	05/12/2019	2	uaterembi		14	27/11/2019	successful nes
2019_2020	05/12/2019	2	uaterembi	32B		27/11/2019	successful nes
2019_2020	05/12/2019	2	gi		18	28/11/2019	successful nes
2019_2020	05/12/2019	2	gi		33	28/11/2019	successful nes
2019_2020	05/12/2019	2	gi		1	28/11/2019	successful nes
2019_2020	05/12/2019	2	gi	29B		28/11/2019	successful nes
2019_2020	05/12/2019	2	gi	12B		28/11/2019	successful nes
2019_2020	05/12/2019	2	gi	33B		28/11/2019	successful nes
2019_2020	05/12/2019	2	gi		44	28/11/2019	successful nes
2019_2020	05/12/2019	2	gi		98	28/11/2019	successful nes
2019_2020	05/12/2019	2	gi		12	28/11/2019	successful nes
2019_2020	05/12/2019	2	gi		3	28/11/2019	successful nes
2019_2020	05/12/2019	2	uatio		26	28/11/2019	successful nes
2019_2020	05/12/2019	2	uatio		17	28/11/2019	successful nes
2019_2020	05/12/2019	2	uatio	8B		28/11/2019	successful nes
2019_2020	05/12/2019	2	uatio	23B		28/11/2019	successful nes
2019_2020	04/12/2019	2	redika	31B		28/11/2019	successful nes
2019_2020	10/12/2019	3	atire		2	27/11/2019	successful nes
2019_2020	10/12/2019	3	atire	31B		04/12/2019	successful nes
2019_2020	10/12/2019	3	atire	9B		04/12/2019	successful nes
2019_2020	10/12/2019	3	atire	25B		04/12/2019	successful nes
2019_2020	10/12/2019	3	atire		4	04/12/2019	successful nes
2019_2020	10/12/2019	3	atire		12	04/12/2019	successful nes
2019_2020	10/12/2019	3	atire		6	04/12/2019	successful nes
2019_2020	10/12/2019	3	atire		7	04/12/2019	successful nes
2019_2020	10/12/2019	3	atire		8	04/12/2019	successful nes
2019_2020	23/12/2019	4	atire	31B		04/12/2019	successful nes
2019_2020	23/12/2019	4	atire	9B		04/12/2019	successful nes
2019_2020	23/12/2019	4	atire	25B		04/12/2019	successful nes
2019_2020	23/12/2019	4	atire		4	04/12/2019	successful nes
2019_2020	23/12/2019	4	atire		12	04/12/2019	successful nes
2019_2020	23/12/2019	4	atire		6	04/12/2019	successful nes
2019_2020	23/12/2019	4	atire		7	04/12/2019	successful nes
2019_2020	23/12/2019	4	atire		8	04/12/2019	successful nes
2019_2020	23/12/2019	4	atire		18	10/12/2019	successful nes
2019_2020	23/12/2019	4	atire		12	10/12/2019	successful nes
2019_2020	23/12/2019	4	atire		16	10/12/2019	successful nes
2019_2020	23/12/2019	4	atire		10	10/12/2019	successful nes
2019_2020	23/12/2019	4	atire		13	10/12/2019	successful nes
2019_2020	23/12/2019	4	atire		17	10/12/2019	successful nes
2019_2020	23/12/2019	4	atire		19	10/12/2019	successful nes
2019_2020	23/12/2019	4	atire		20	10/12/2019	successful nes
2019_2020	23/12/2019	4	atire		21	10/12/2019	successful nes
2019_2020	23/12/2019	4	atire		39	10/12/2019	successful nes
2019_2020	23/12/2019	4	atire	27B		10/12/2019	successful nes
2019_2020	23/12/2019	4	atire		37	10/12/2019	successful nes

2019_2020	23/12/2019	4	atire		1	10/12/2019	successful nes
2019_2020	23/12/2019	4	atire	17B		10/12/2019	successful nes
2019_2020	03/01/2020	5	kouare		22	05/12/2019	successful nes
2019_2020	03/01/2020	5	kouare		23	05/12/2019	successful nes
2019_2020	03/01/2020	5	kouare		24	05/12/2019	successful nes
2019_2020	03/01/2020	5	kouare		25	05/12/2019	successful nes
2019_2020	02/01/2020	5	nge		1	04/12/2019	successful nes
2019_2020	02/01/2020	5	nge		2	04/12/2019	successful nes
2019_2020	02/01/2020	5	nge		3	04/12/2019	successful nes
2019_2020	02/01/2020	5	nge		4	04/12/2019	successful nes
2019_2020	02/01/2020	5	nge		5	04/12/2019	successful nes
2019_2020	02/01/2020	5	nge		6	04/12/2019	successful nes
2019_2020	02/01/2020	5	nge		7	04/12/2019	successful nes
2019_2020	02/01/2020	5	nge		8	05/12/2019	successful nes
2019_2020	02/01/2020	5	gi		18	28/11/2019	successful nes
2019_2020	02/01/2020	5	gi		33	28/11/2019	successful nes
2019_2020	02/01/2020	5	gi		1	28/11/2019	successful nes
2019_2020	02/01/2020	5	gi	29B		28/11/2019	successful nes
2019_2020	02/01/2020	5	gi	12B		28/11/2019	successful nes
2019_2020	02/01/2020	5	gi	33B		28/11/2019	successful nes
2019_2020	02/01/2020	5	gi		44	28/11/2019	successful nes
2019_2020	02/01/2020	5	gi		98	28/11/2019	successful nes
2019_2020	02/01/2020	5	gi		12	28/11/2019	successful nes
2019_2020	02/01/2020	5	gi		3	28/11/2019	successful nes
2019_2020	02/01/2020	5	gi		6	05/12/2019	successful nes
2019_2020	02/01/2020	5	uatio		17	28/11/2019	successful nes
2019_2020	02/01/2020	5	uatio	8B		28/11/2019	successful nes
2019_2020	02/01/2020	5	uatio	23B		28/11/2019	successful nes
2019_2020	02/01/2020	5	uatio		9	05/12/2019	successful nes
2019_2020	02/01/2020	5	uatio		37	05/12/2019	successful nes
2019_2020	02/01/2020	5	uaterembi		16	27/11/2019	successful nes
2019_2020	02/01/2020	5	uaterembi		35	27/11/2019	successful nes
2019_2020	02/01/2020	5	uaterembi		14	27/11/2019	successful nes
2019_2020	02/01/2020	5	uaterembi	32B		27/11/2019	successful nes
2019_2020	02/01/2020	5	uaterembi		20	05/12/2019	successful nes
2019_2020	02/01/2020	5	uaterembi		26	05/12/2019	successful nes
2019_2020	02/01/2020	5	uaterembi		27	05/12/2019	successful nes
2019_2020	02/01/2020	5	uaterembi		28	05/12/2019	successful nes
2019_2020	02/01/2020	5	uaterembi		29	05/12/2019	successful nes
2019_2020	02/01/2020	5	uaterembi		30	05/12/2019	successful nes
2019_2020	02/01/2020	5	uaterembi		31	05/12/2019	successful nes
2019_2020	02/01/2020	5	uaterembi		32	05/12/2019	successful nes
2019_2020	02/01/2020	5	uaterembi		24	05/12/2019	successful nes
2019_2020	02/01/2020	5	uaterembi		33	05/12/2019	successful nes
2019_2020	02/01/2020	5	uaterembi		34	05/12/2019	successful nes
2019_2020	02/01/2020	5	uaterembi		35	05/12/2019	successful nes
2019_2020	02/01/2020	5	redika		15	04/12/2019	successful nes
2019_2020	02/01/2020	5	redika		10	04/12/2019	successful nes
2019_2020	02/01/2020	5	redika		1	04/12/2019	successful nes
2019_2020	09/01/2020	6	ieroue		51	04/12/2019	successful nes

2019_2020	09/01/2020	6	ieroue		52	04/12/2019	successful nes
2019_2020	16/01/2020	7	redika		1	02/01/2020	successful nes
2019_2020	16/01/2020	7	redika		2	02/01/2020	successful nes
2019_2020	16/01/2020	7	redika		3	02/01/2020	successful nes
2019_2020	16/01/2020	7	redika		4	02/01/2020	successful nes
2019_2020	16/01/2020	7	redika		5	02/01/2020	successful nes
2019_2020	16/01/2020	7	redika		6	02/01/2020	successful nes
2019_2020	16/01/2020	7	uatio		7	02/01/2020	successful nes
2019_2020	16/01/2020	7	uatio		8	02/01/2020	successful nes
2019_2020	16/01/2020	7	uatio		9	02/01/2020	successful nes
2019_2020	16/01/2020	7	uatio		10	02/01/2020	successful nes
2019_2020	16/01/2020	7	kouare		20	03/01/2020	successful nes
2019_2020	16/01/2020	7	kouare		21	03/01/2020	successful nes
2019_2020	16/01/2020	7	kouare		23	05/12/2019	successful nes
2019_2020	16/01/2020	7	kouare		24	05/12/2019	successful nes
2019_2020	16/01/2020	7	kouare		25	05/12/2019	successful nes
2019_2020	16/01/2020	7	gi		11	02/01/2020	successful nes
2019_2020	16/01/2020	7	gi		12	02/01/2020	successful nes
2019_2020	16/01/2020	7	gi		13	02/01/2020	successful nes
2019_2020	16/01/2020	7	gi		14	02/01/2020	successful nes
2019_2020	16/01/2020	7	gi		15	02/01/2020	successful nes
2019_2020	16/01/2020	7	gi		18	28/11/2019	successful nes
2019_2020	16/01/2020	7	gi		33	28/11/2019	successful nes
2019_2020	16/01/2020	7	gi		1	28/11/2019	successful nes
2019_2020	16/01/2020	7	gi		12	28/11/2019	successful nes
2019_2020	16/01/2020	7	nge		16	02/01/2020	successful nes
2019_2020	16/01/2020	7	nge		17	02/01/2020	successful nes
2019_2020	16/01/2020	7	nge		18	02/01/2020	successful nes
2019_2020	16/01/2020	7	nge		19	02/01/2020	successful nes
2019_2020	16/01/2020	7	nge		3	04/12/2019	successful nes
2019_2020	16/01/2020	7	nge		6	04/12/2019	successful nes
2019_2020	16/01/2020	7	nge		8	05/12/2019	successful nes
2019_2020	17/01/2020	7	uie	18B		03/01/2020	successful nes
2019_2020	17/01/2020	7	uie		37	03/01/2020	successful nes
2019_2020	17/01/2020	7	uie		33	03/01/2020	successful nes
2019_2020	17/01/2020	7	ieroue		51	09/01/2020	successful nes
2019_2020	17/01/2020	7	puemba		22	03/01/2020	successful nes
2019_2020	17/01/2020	7	puemba		23	03/01/2020	successful nes
2019_2020	17/01/2020	7	puemba		24	03/01/2020	successful nes
2019_2020	17/01/2020	7	puemba		25	03/01/2020	successful nes
2019_2020	17/01/2020	7	uo		26	03/01/2020	successful nes
2019_2020	17/01/2020	7	uo		4	17/01/2020	successful nes
2019_2020	17/01/2020	7	atire	29B		23/12/2019	successful nes
2019_2020	17/01/2020	7	atire	31B		04/12/2019	successful nes
2019_2020	17/01/2020	7	atire	9B		04/12/2019	successful nes
2019_2020	17/01/2020	7	atire	22B		23/12/2019	successful nes
2019_2020	17/01/2020	7	atire		18	10/12/2019	successful nes
2019_2020	17/01/2020	7	atire		12	04/12/2019	successful nes
2019_2020	17/01/2020	7	atire		16	10/12/2019	successful nes
2019_2020	17/01/2020	7	atire		25	23/12/2019	successful nes

2019_2020	17/01/2020	7	atire	11B		24/12/2019	successful nes
2019_2020	17/01/2020	7	atire		10	10/12/2019	successful nes
2019_2020	17/01/2020	7	atire		13	10/12/2019	successful nes
2019_2020	17/01/2020	7	atire		17	10/12/2019	successful nes
2019_2020	17/01/2020	7	atire	28B		24/12/2019	successful nes
2019_2020	17/01/2020	7	atire		6	04/12/2019	successful nes
2019_2020	17/01/2020	7	atire		19	10/12/2019	successful nes
2019_2020	17/01/2020	7	atire		20	10/12/2019	successful nes
2019_2020	17/01/2020	7	atire		5	24/12/2019	successful nes
2019_2020	17/01/2020	7	atire		21	10/12/2019	successful nes
2019_2020	17/01/2020	7	atire		11	24/12/2019	successful nes
2019_2020	17/01/2020	7	atire	19B		24/12/2019	successful nes
2019_2020	17/01/2020	7	atire		8	04/12/2019	successful nes
2019_2020	17/01/2020	7	atire		39	10/12/2019	successful nes
2019_2020	17/01/2020	7	atire	17B		10/12/2019	successful nes
2019_2020	17/01/2020	7	atire		55	24/12/2019	successful nes
2019_2020	17/01/2020	7	atire	27B		10/12/2019	successful nes
2019_2020	17/01/2020	7	atire		37	10/12/2019	successful nes
2019_2020	17/01/2020	7	atire	3B		24/12/2019	successful nes
2019_2020	17/01/2020	7	atire		22	24/12/2019	successful nes
2019_2020	17/01/2020	7	atire	25B		04/12/2019	successful nes
2019_2020	17/01/2020	7	atire		66	24/12/2019	successful nes
2019_2020	17/01/2020	7	atire		23	23/12/2019	successful nes
2019_2020	17/01/2020	7	atire	4B		23/12/2019	successful nes
2019_2020	17/01/2020	7	atire		45	23/12/2019	successful nes
2019_2020	28/01/2020	9	atire		18	10/12/2019	successful nes
2019_2020	28/01/2020	9	atire		12	04/12/2019	successful nes
2019_2020	28/01/2020	9	atire	11B		24/12/2019	successful nes
2019_2020	28/01/2020	9	atire		16	10/12/2019	successful nes
2019_2020	28/01/2020	9	atire		25	23/12/2019	successful nes
2019_2020	28/01/2020	9	atire	17B		10/12/2019	successful nes
2019_2020	28/01/2020	9	kouare		21	03/01/2020	successful nes
2021_2022	17/11/2021	1	atire		62	17/11/2021	successful nes
2021_2022	17/11/2021	1	vua		55	17/11/2021	successful nes
2021_2022	04/11/2021	0	nge		45	17/11/2021	successful nes
2021_2022	17/11/2021	1	nge		45	17/11/2021	successful nes
2021_2022	18/11/2021	1	gi		43	18/11/2021	successful nes
2021_2022	18/11/2021	1	gi		51	18/11/2021	successful nes
2021_2022	18/11/2021	1	gi		100	18/11/2021	successful nes
2021_2022	07/12/2021	2	vua		55	17/11/2021	successful nes
2021_2022	07/12/2021	2	vua		40	07/12/2021	successful nes
2021_2022	07/12/2021	2	vua		19	07/12/2021	successful nes
2021_2022	07/12/2021	2	vua		56	07/12/2021	successful nes
2021_2022	07/12/2021	2	vua		53	07/12/2021	successful nes
2021_2022	07/12/2021	2	vua		21	07/12/2021	successful nes
2021_2022	07/12/2021	2	vua		60	07/12/2021	successful nes
2021_2022	07/12/2021	2	vua		32	07/12/2021	aborted bodyp
2021_2022	07/12/2021	2	vua		57	07/12/2021	aborted bodyp
2021_2022	07/12/2021	2	uaterembi		50	07/12/2021	successful nes
2021_2022	07/12/2021	2	uaterembi		66	07/12/2021	successful nes

2021_2022	07/12/2021	2	uaterembi	36	07/12/2021	successful nes
2021_2022	07/12/2021	2	uaterembi	34	07/12/2021	successful nes
2021_2022	07/12/2021	2	uaterembi	22	07/12/2021	successful nes
2021_2022	07/12/2021	2	uaterembi	58	07/12/2021	successful nes
2021_2022	07/12/2021	2	uaterembi	36	07/12/2021	successful nes
2021_2022	07/12/2021	2	gi	14	07/12/2021	successful nes
2021_2022	07/12/2021	2	gi	7	07/12/2021	successful nes
2021_2022	07/12/2021	2	gi	3	07/12/2021	successful nes
2021_2022	07/12/2021	2	gi	23	07/12/2021	successful nes
2021_2022	07/12/2021	2	gi	54	07/12/2021	successful nes
2021_2022	07/12/2021	2	gi	49	07/12/2021	successful nes
2021_2022	07/12/2021	2	gi	3	07/12/2021	successful nes
2021_2022	07/12/2021	2	gi	43	18/11/2021	successful nes
2021_2022	07/12/2021	2	gi	51	18/11/2021	successful nes
2021_2022	07/12/2021	2	gi	100	18/11/2021	successful nes
2021_2022	07/12/2021	2	kouare	29	07/12/2021	successful nes
2021_2022	07/12/2021	2	nda	63	07/12/2021	successful nes
2021_2022	07/12/2021	2	nda	20	07/12/2021	successful nes
2021_2022	07/12/2021	2	nda	10	07/12/2021	successful nes
2021_2022	07/12/2021	2	nge	27	07/12/2021	successful nes
2021_2022	07/12/2021	2	nge	4	07/12/2021	successful nes
2021_2022	07/12/2021	2	nge	18	07/12/2021	successful nes
2021_2022	07/12/2021	2	nge	51	07/12/2021	successful nes
2021_2022	07/12/2021	2	nge	24	07/12/2021	successful nes
2021_2022	07/12/2021	2	nge	50	07/12/2021	successful nes
2021_2022	07/12/2021	2	nge	65	07/12/2021	successful nes
2021_2022	07/12/2021	2	nge	13	07/12/2021	successful nes
2021_2022	07/12/2021	2	nge	15	07/12/2021	successful nes
2021_2022	07/12/2021	2	nge	"poteau méta	07/12/2021	successful nes
2021_2022	07/12/2021	2	nge	45	17/11/2021	successful nes
2021_2022	08/12/2021	2	atire	62	17/11/2021	successful nes
2021_2022	08/12/2021	2	redika	62	08/12/2021	successful nes
2021_2022	08/12/2021	2	redika	12	08/12/2021	successful nes
2021_2022	08/12/2021	2	redika	17	08/12/2021	successful nes
2021_2022	08/12/2021	2	redika	48	08/12/2021	successful nes
2021_2022	21/12/2021	4	vua	55	17/11/2021	successful nes
2021_2022	21/12/2021	4	vua	40	07/12/2021	successful nes
2021_2022	21/12/2021	4	vua	19	07/12/2021	successful nes
2021_2022	21/12/2021	4	vua	56	07/12/2021	successful nes
2021_2022	21/12/2021	4	vua	53	07/12/2021	successful nes
2021_2022	21/12/2021	4	vua	21	07/12/2021	successful nes
2021_2022	21/12/2021	4	vua	60	07/12/2021	successful nes
2021_2022	21/12/2021	4	vua	32	07/12/2021	aborted body
2021_2022	21/12/2021	4	vua	57	07/12/2021	aborted body
2021_2022	21/12/2021	4	uaterembi	50	07/12/2021	successful nes
2021_2022	21/12/2021	4	uaterembi	66	07/12/2021	successful nes
2021_2022	21/12/2021	4	uaterembi	36	07/12/2021	successful nes
2021_2022	21/12/2021	4	uaterembi	34	07/12/2021	successful nes
2021_2022	21/12/2021	4	uaterembi	22	07/12/2021	successful nes
2021_2022	21/12/2021	4	uaterembi	58	07/12/2021	successful nes

2021_2022	21/12/2021	4	uaterembi	36	07/12/2021	successful nes
2021_2022	21/12/2021	4	kouare	29	07/12/2021	successful nes
2021_2022	21/12/2021	4	nda	63	07/12/2021	successful nes
2021_2022	21/12/2021	4	nda	20	07/12/2021	successful nes
2021_2022	21/12/2021	4	nda	10	07/12/2021	successful nes
2021_2022	22/12/2021	4	nge	45	17/11/2021	successful nes
2021_2022	22/12/2021	4	nge	27	07/12/2021	successful nes
2021_2022	22/12/2021	4	nge	4	07/12/2021	successful nes
2021_2022	22/12/2021	4	nge	18	07/12/2021	successful nes
2021_2022	22/12/2021	4	nge	51	07/12/2021	successful nes
2021_2022	22/12/2021	4	nge	24	07/12/2021	successful nes
2021_2022	22/12/2021	4	nge	50	07/12/2021	successful nes
2021_2022	22/12/2021	4	nge	65	07/12/2021	successful nes
2021_2022	22/12/2021	4	nge	13	07/12/2021	successful nes
2021_2022	22/12/2021	4	nge	15	07/12/2021	successful nes
2021_2022	22/12/2021	4	nge	"poteau méta	07/12/2021	successful nes
2021_2022	22/12/2021	4	gi	43	18/11/2021	successful nes
2021_2022	22/12/2021	4	gi	100	18/11/2021	successful nes
2021_2022	22/12/2021	4	gi	14	07/12/2021	successful nes
2021_2022	22/12/2021	4	gi	7	07/12/2021	successful nes
2021_2022	22/12/2021	4	gi	3	07/12/2021	successful nes
2021_2022	22/12/2021	4	gi	23	07/12/2021	successful nes
2021_2022	22/12/2021	4	gi	54	07/12/2021	successful nes
2021_2022	22/12/2021	4	gi	49	07/12/2021	successful nes
2021_2022	22/12/2021	4	gi	3	07/12/2021	successful nes
2021_2022	22/12/2021	4	redika	62	08/12/2021	successful nes
2021_2022	22/12/2021	4	redika	12	08/12/2021	successful nes
2021_2022	22/12/2021	4	redika	17	08/12/2021	successful nes
2021_2022	22/12/2021	4	redika	48	08/12/2021	successful nes
2021_2022	21/12/2021	4	atire	1	21/12/2021	successful nes
2021_2022	21/12/2021	4	atire	46	21/12/2021	successful nes
2021_2022	21/12/2021	4	ua	25	21/12/2021	successful nes
2021_2022	21/12/2021	4	uaterembi	9	21/12/2021	successful nes
2021_2022	21/12/2021	4	uaterembi	56	21/12/2021	successful nes
2021_2022	21/12/2021	4	uaterembi	55	21/12/2021	successful nes
2021_2022	21/12/2021	4	uaterembi	34	21/12/2021	successful nes
2021_2022	21/12/2021	4	uaterembi	41	21/12/2021	successful nes
2021_2022	21/12/2021	4	kouare	21	21/12/2021	successful nes
2021_2022	21/12/2021	4	kouare	66	21/12/2021	successful nes
2021_2022	21/12/2021	4	nda	11	21/12/2021	successful nes
2021_2022	22/12/2021	4	nge	70	22/12/2021	successful nes
2021_2022	22/12/2021	4	nge	42	22/12/2021	successful nes
2021_2022	22/12/2021	4	nge	52	22/12/2021	successful nes
2021_2022	22/12/2021	4	nge	25	22/12/2021	successful nes
2021_2022	22/12/2021	4	nge	30	22/12/2021	successful nes
2021_2022	22/12/2021	4	nge	67	22/12/2021	successful nes
2021_2022	22/12/2021	4	nge	23	22/12/2021	successful nes
2021_2022	22/12/2021	4	nge	7	22/12/2021	successful nes
2021_2022	22/12/2021	4	nge	11	22/12/2021	successful nes
2021_2022	22/12/2021	4	gi	67	22/12/2021	successful nes

2021_2022	22/12/2021	4	gi	4	22/12/2021	successful nes
2021_2022	22/12/2021	4	gi	35	22/12/2021	successful nes
2021_2022	22/12/2021	4	gi	68	22/12/2021	successful nes
2021_2022	22/12/2021	4	gi	95	22/12/2021	successful nes
2021_2022	22/12/2021	4	uatio	23	22/12/2021	successful nes
2021_2022	22/12/2021	4	redika	31	22/12/2021	successful nes
2021_2022	22/12/2021	4	redika	22	22/12/2021	successful nes
2021_2022	22/12/2021	4	redika	99	22/12/2021	successful nes
2021_2022	21/12/2021	4	uaterembi	52	21/12/2021	aborted body[illegible]
2021_2022	21/12/2021	4	uaterembi	51	21/12/2021	aborted body[illegible]
2021_2022	22/12/2021	4	gi	21	22/12/2021	aborted body[illegible]
2021_2022	04/01/2022	6	atire	1	21/12/2021	successful nes
2021_2022	04/01/2022	6	atire	46	21/12/2021	successful nes
2021_2022	04/01/2022	6	atire	31	04/01/2022	successful nes
2021_2022	04/01/2022	6	atire	34	04/01/2022	successful nes
2021_2022	04/01/2022	6	atire	30	04/01/2022	successful nes
2021_2022	04/01/2022	6	atire	41	04/01/2022	successful nes
2021_2022	04/01/2022	6	atire	5	04/01/2022	successful nes
2021_2022	04/01/2022	6	atire	9	04/01/2022	successful nes
2021_2022	04/01/2022	6	atire	"bambou"	04/01/2022	successful nes
2021_2022	04/01/2022	6	vua	1	04/01/2022	successful nes
2021_2022	04/01/2022	6	vua	47	04/01/2022	successful nes
2021_2022	04/01/2022	6	vua	"sternes"	04/01/2022	successful nes
2021_2022	04/01/2022	6	vua	32	04/01/2022	successful nes
2021_2022	04/01/2022	6	vua	38	04/01/2022	aborted body[illegible]
2021_2022	04/01/2022	6	vua	42	04/01/2022	aborted body[illegible]
2021_2022	04/01/2022	6	vua	19	07/12/2021	successful nes
2021_2022	04/01/2022	6	vua	53	07/12/2021	successful nes
2021_2022	04/01/2022	6	vua	60	07/12/2021	successful nes
2021_2022	04/01/2022	6	vua	32	07/12/2021	aborted body[illegible]
2021_2022	04/01/2022	6	uaterembi	22	07/12/2021	successful nes
2021_2022	04/01/2022	6	uaterembi	36	07/12/2021	successful nes
2021_2022	04/01/2022	6	uaterembi	58	07/12/2021	successful nes
2021_2022	04/01/2022	6	uaterembi	9	21/12/2021	successful nes
2021_2022	04/01/2022	6	uaterembi	56	21/12/2021	successful nes
2021_2022	04/01/2022	6	uaterembi	55	21/12/2021	successful nes
2021_2022	04/01/2022	6	uaterembi	34	21/12/2021	successful nes
2021_2022	04/01/2022	6	uaterembi	41	21/12/2021	successful nes
2021_2022	04/01/2022	6	uaterembi	52	21/12/2021	aborted body[illegible]
2021_2022	04/01/2022	6	uaterembi	51	21/12/2021	aborted body[illegible]
2021_2022	04/01/2022	6	uaterembi	6	04/01/2022	successful nes
2021_2022	04/01/2022	6	uaterembi	61	04/01/2022	successful nes
2021_2022	04/01/2022	6	uaterembi	68	04/01/2022	successful nes
2021_2022	04/01/2022	6	uaterembi	2	04/01/2022	successful nes
2021_2022	04/01/2022	6	uaterembi	11	04/01/2022	successful nes
2021_2022	04/01/2022	6	kouare	58	04/01/2022	successful nes
2021_2022	04/01/2022	6	kouare	51	04/01/2022	successful nes
2021_2022	04/01/2022	6	kouare	4	04/01/2022	aborted body[illegible]
2021_2022	04/01/2022	6	kouare	29	07/12/2021	successful nes
2021_2022	04/01/2022	6	kouare	21	21/12/2021	successful nes

2021_2022	04/01/2022	6	kouare	66	21/12/2021	successful nes
2021_2022	04/01/2022	6	nda	22	04/01/2022	successful nes
2021_2022	04/01/2022	6	nda	63	07/12/2021	successful nes
2021_2022	04/01/2022	6	nda	20	07/12/2021	successful nes
2021_2022	04/01/2022	6	nda	10	07/12/2021	successful nes
2021_2022	04/01/2022	6	nda	11	21/12/2021	successful nes
2021_2022	04/01/2022	6	nge	27	07/12/2021	successful nes
2021_2022	04/01/2022	6	nge	4	07/12/2021	successful nes
2021_2022	04/01/2022	6	nge	18	07/12/2021	successful nes
2021_2022	04/01/2022	6	nge	51	07/12/2021	successful nes
2021_2022	04/01/2022	6	nge	24	07/12/2021	successful nes
2021_2022	04/01/2022	6	nge	50	07/12/2021	successful nes
2021_2022	04/01/2022	6	nge	65	07/12/2021	successful nes
2021_2022	04/01/2022	6	nge	13	07/12/2021	successful nes
2021_2022	04/01/2022	6	nge	15	07/12/2021	successful nes
2021_2022	04/01/2022	6	nge	"poteau méta	07/12/2021	successful nes
2021_2022	04/01/2022	6	nge	70	22/12/2021	successful nes
2021_2022	04/01/2022	6	nge	42	22/12/2021	successful nes
2021_2022	04/01/2022	6	nge	52	22/12/2021	successful nes
2021_2022	04/01/2022	6	nge	25	22/12/2021	successful nes
2021_2022	04/01/2022	6	nge	30	22/12/2021	successful nes
2021_2022	04/01/2022	6	nge	67	22/12/2021	successful nes
2021_2022	04/01/2022	6	nge	23	22/12/2021	successful nes
2021_2022	04/01/2022	6	nge	7	22/12/2021	successful nes
2021_2022	04/01/2022	6	nge	11	22/12/2021	successful nes
2021_2022	04/01/2022	6	nge	5	04/01/2022	successful nes
2021_2022	04/01/2022	6	nge	37	04/01/2022	successful nes
2021_2022	04/01/2022	6	nge	8	04/01/2022	successful nes
2021_2022	04/01/2022	6	nge	3	04/01/2022	successful nes
2021_2022	04/01/2022	6	nge	59	04/01/2022	successful nes
2021_2022	04/01/2022	6	nge	20	04/01/2022	successful nes
2021_2022	04/01/2022	6	nge	24	04/01/2022	aborted bodyp
2021_2022	04/01/2022	6	nge	56	04/01/2022	successful nes
2021_2022	05/01/2022	6	gi	43	18/11/2021	successful nes
2021_2022	05/01/2022	6	gi	100	18/11/2021	successful nes
2021_2022	05/01/2022	6	gi	14	07/12/2021	successful nes
2021_2022	05/01/2022	6	gi	7	07/12/2021	successful nes
2021_2022	05/01/2022	6	gi	3	07/12/2021	successful nes
2021_2022	05/01/2022	6	gi	54	07/12/2021	successful nes
2021_2022	05/01/2022	6	gi	49	07/12/2021	successful nes
2021_2022	05/01/2022	6	gi	33	07/12/2021	successful nes
2021_2022	05/01/2022	6	gi	67	22/12/2021	successful nes
2021_2022	05/01/2022	6	gi	4	22/12/2021	successful nes
2021_2022	05/01/2022	6	gi	35	22/12/2021	successful nes
2021_2022	05/01/2022	6	gi	68	22/12/2021	successful nes
2021_2022	05/01/2022	6	gi	95	22/12/2021	successful nes
2021_2022	05/01/2022	6	gi	21	22/12/2021	aborted bodyp
2021_2022	05/01/2022	6	gi	50	05/01/2022	successful nes
2021_2022	05/01/2022	6	gi	52	05/01/2022	successful nes
2021_2022	05/01/2022	6	gi	9	05/01/2022	aborted bodyp

2021_2022	05/01/2022	6	gi		36	05/01/2022	successful nes
2021_2022	05/01/2022	6	gi		55	05/01/2022	successful nes
2021_2022	05/01/2022	6	gi		21	05/01/2022	successful nes
2021_2022	05/01/2022	6	gi		32	05/01/2022	successful nes
2021_2022	05/01/2022	6	gi		52	05/01/2022	successful nes
2021_2022	05/01/2022	6	gi	21B		05/01/2022	successful nes
2021_2022	05/01/2022	6	gi		51	05/01/2022	successful nes
2021_2022	05/01/2022	6	uatio		23	22/12/2021	successful nes
2021_2022	05/01/2022	6	uatio		46	05/01/2022	successful nes
2021_2022	05/01/2022	6	uatio		28	05/01/2022	successful nes
2021_2022	05/01/2022	6	uatio		7	05/01/2022	aborted bodyp
2021_2022	05/01/2022	6	ua		25	21/12/2021	successful nes
2021_2022	05/01/2022	6	ua		27	05/01/2022	successful nes
2021_2022	22/12/2021	4	ieroue	nd		22/12/2021	successful nes
2021_2022	05/01/2022	6	ieroue	nd		22/12/2021	successful nes
2021_2022	05/01/2022	6	ieroue		25	05/01/2022	successful nes
2021_2022	05/01/2022	6	redika		62	08/12/2021	successful nes
2021_2022	05/01/2022	6	redika		12	08/12/2021	successful nes
2021_2022	05/01/2022	6	redika		17	08/12/2021	successful nes
2021_2022	05/01/2022	6	redika		48	08/12/2021	successful nes
2021_2022	05/01/2022	6	redika		31	22/12/2021	successful nes
2021_2022	05/01/2022	6	redika		22	22/12/2021	successful nes
2021_2022	05/01/2022	6	redika		99	22/12/2021	successful nes
2021_2022	05/01/2022	6	redika		63	05/01/2022	aborted bodyp
2021_2022	05/01/2022	6	redika		35	05/01/2022	successful nes
2021_2022	05/01/2022	6	redika		67	05/01/2022	successful nes
2021_2022	05/01/2022	6	redika		70	05/01/2022	successful nes
2021_2022	26/01/2022	7	vua		60	26/01/2022	successful nes
2021_2022	26/01/2022	7	vua		38	26/01/2022	successful nes
2021_2022	26/01/2022	7	vua		42	26/01/2022	successful nes
2021_2022	26/01/2022	7	vua		19	26/01/2022	successful nes
2021_2022	26/01/2022	7	vua		53	26/01/2022	successful nes
2021_2022	26/01/2022	7	vua		32	26/01/2022	successful nes
2021_2022	26/01/2022	7	vua		19	07/12/2021	successful nes
2021_2022	26/01/2022	7	vua		53	07/12/2021	successful nes
2021_2022	26/01/2022	7	vua		60	07/12/2021	successful nes
2021_2022	26/01/2022	7	vua		1	04/01/2022	successful nes
2021_2022	26/01/2022	7	vua		47	04/01/2022	successful nes
2021_2022	26/01/2022	7	vua	"sternes"		04/01/2022	successful nes
2021_2022	26/01/2022	7	vua		32	04/01/2022	successful nes
2021_2022	26/01/2022	7	vua		38	04/01/2022	aborted bodyp
2021_2022	26/01/2022	7	vua		42	04/01/2022	aborted bodyp
2021_2022	26/01/2022	7	ieroue	nd		26/01/2022	successful nes
2021_2022	26/01/2022	7	ieroue		25	26/01/2022	successful nes
2021_2022	26/01/2022	7	ieroue		25	05/01/2022	successful nes
2021_2022	26/01/2022	7	uaterembi	"bois"		26/01/2022	successful nes
2021_2022	26/01/2022	7	uaterembi		6	26/01/2022	successful nes
2021_2022	26/01/2022	7	uaterembi		2	26/01/2022	successful nes
2021_2022	26/01/2022	7	uaterembi		41	26/01/2022	successful nes
2021_2022	26/01/2022	7	uaterembi		9	21/12/2021	successful nes

2021_2022	26/01/2022	7	uaterembi	34	21/12/2021	successful nes
2021_2022	26/01/2022	7	uaterembi	41	21/12/2021	successful nes
2021_2022	26/01/2022	7	uaterembi	6	04/01/2022	successful nes
2021_2022	26/01/2022	7	uaterembi	61	04/01/2022	successful nes
2021_2022	26/01/2022	7	uaterembi	68	04/01/2022	successful nes
2021_2022	26/01/2022	7	uaterembi	2	04/01/2022	successful nes
2021_2022	26/01/2022	7	uaterembi	11	04/01/2022	successful nes
2021_2022	26/01/2022	7	uatio	11	26/01/2022	successful nes
2021_2022	26/01/2022	7	uatio	23	22/12/2021	successful nes
2021_2022	26/01/2022	7	uatio	46	05/01/2022	successful nes
2021_2022	26/01/2022	7	uatio	28	05/01/2022	successful nes
2021_2022	26/01/2022	7	uatio	7	05/01/2022	aborted bodyp
2021_2022	26/01/2022	7	ua	27	05/01/2022	successful nes
2021_2022	26/01/2022	7	ua	25	21/12/2021	successful nes
2021_2022	26/01/2022	7	gi	27	26/01/2022	successful nes
2021_2022	26/01/2022	7	gi	21	26/01/2022	successful nes
2021_2022	26/01/2022	7	gi	55	26/01/2022	successful nes
2021_2022	26/01/2022	7	gi	21	26/01/2022	successful nes
2021_2022	26/01/2022	7	gi	21	26/01/2022	successful nes
2021_2022	26/01/2022	7	gi	35	26/01/2022	successful nes
2021_2022	26/01/2022	7	gi	36	26/01/2022	successful nes
2021_2022	26/01/2022	7	gi	"bois"	26/01/2022	successful nes
2021_2022	26/01/2022	7	gi	"bambou"	26/01/2022	successful nes
2021_2022	26/01/2022	7	gi	"big bambou e	26/01/2022	successful nes
2021_2022	26/01/2022	7	gi	7	26/01/2022	successful nes
2021_2022	26/01/2022	7	gi	43	18/11/2021	successful nes
2021_2022	26/01/2022	7	gi	14	07/12/2021	successful nes
2021_2022	26/01/2022	7	gi	7	07/12/2021	successful nes
2021_2022	26/01/2022	7	gi	3	07/12/2021	successful nes
2021_2022	26/01/2022	7	gi	54	07/12/2021	successful nes
2021_2022	26/01/2022	7	gi	49	07/12/2021	successful nes
2021_2022	26/01/2022	7	gi	33	07/12/2021	successful nes
2021_2022	26/01/2022	7	gi	67	22/12/2021	successful nes
2021_2022	26/01/2022	7	gi	35	22/12/2021	successful nes
2021_2022	26/01/2022	7	gi	68	22/12/2021	successful nes
2021_2022	26/01/2022	7	gi	95	22/12/2021	successful nes
2021_2022	26/01/2022	7	gi	21	22/12/2021	aborted bodyp
2021_2022	26/01/2022	7	gi	50	05/01/2022	successful nes
2021_2022	26/01/2022	7	gi	52	05/01/2022	successful nes
2021_2022	26/01/2022	7	gi	9	05/01/2022	aborted bodyp
2021_2022	26/01/2022	7	gi	36	05/01/2022	successful nes
2021_2022	26/01/2022	7	gi	55	05/01/2022	successful nes
2021_2022	26/01/2022	7	gi	21	05/01/2022	successful nes
2021_2022	26/01/2022	7	gi	32	05/01/2022	successful nes
2021_2022	26/01/2022	7	gi	52	05/01/2022	successful nes
2021_2022	26/01/2022	7	gi	21B	05/01/2022	successful nes
2021_2022	26/01/2022	7	gi	51	05/01/2022	successful nes
2021_2022	27/01/2022	7	nge	15	27/01/2022	successful nes
2021_2022	27/01/2022	7	nge	39	27/01/2022	successful nes
2021_2022	27/01/2022	7	nge	67	27/01/2022	successful nes

2021_2022	27/01/2022	7	nge	40	27/01/2022	successful nes
2021_2022	27/01/2022	7	nge	25	27/01/2022	successful nes
2021_2022	27/01/2022	7	nge	13	27/01/2022	successful nes
2021_2022	27/01/2022	7	nge	27	07/12/2021	successful nes
2021_2022	27/01/2022	7	nge	4	07/12/2021	successful nes
2021_2022	27/01/2022	7	nge	18	07/12/2021	successful nes
2021_2022	27/01/2022	7	nge	65	07/12/2021	successful nes
2021_2022	27/01/2022	7	nge	13	07/12/2021	successful nes
2021_2022	27/01/2022	7	nge	15	07/12/2021	successful nes
2021_2022	27/01/2022	7	nge	70	22/12/2021	successful nes
2021_2022	27/01/2022	7	nge	42	22/12/2021	successful nes
2021_2022	27/01/2022	7	nge	25	22/12/2021	successful nes
2021_2022	27/01/2022	7	nge	30	22/12/2021	successful nes
2021_2022	27/01/2022	7	nge	67	22/12/2021	successful nes
2021_2022	27/01/2022	7	nge	23	22/12/2021	successful nes
2021_2022	27/01/2022	7	nge	11	22/12/2021	successful nes
2021_2022	27/01/2022	7	nge	37	04/01/2022	successful nes
2021_2022	27/01/2022	7	nge	8	04/01/2022	successful nes
2021_2022	27/01/2022	7	nge	3	04/01/2022	successful nes
2021_2022	27/01/2022	7	nge	59	04/01/2022	successful nes
2021_2022	27/01/2022	7	nge	20	04/01/2022	successful nes
2021_2022	27/01/2022	7	nge	24	04/01/2022	aborted bodyp
2021_2022	27/01/2022	7	nge	56	04/01/2022	successful nes
2021_2022	27/01/2022	7	kouare	24	27/01/2022	successful nes
2021_2022	27/01/2022	7	kouare	44	27/01/2022	successful nes
2021_2022	27/01/2022	7	kouare	"panneau kite	27/01/2022	successful nes
2021_2022	27/01/2022	7	kouare	66	21/12/2021	successful nes
2021_2022	27/01/2022	7	kouare	58	04/01/2022	successful nes
2021_2022	27/01/2022	7	kouare	51	04/01/2022	successful nes
2021_2022	27/01/2022	7	kouare	4	04/01/2022	aborted bodyp
2021_2022	27/01/2022	7	nda	58	27/01/2022	successful nes
2021_2022	27/01/2022	7	nda	10	07/12/2021	successful nes
2021_2022	27/01/2022	7	nda	11	21/12/2021	successful nes
2021_2022	27/01/2022	7	nda	22	04/01/2022	successful nes
2021_2022	27/01/2022	7	redika	62	27/01/2022	successful nes
2021_2022	27/01/2022	7	redika	62	08/12/2021	successful nes
2021_2022	27/01/2022	7	redika	12	08/12/2021	successful nes
2021_2022	27/01/2022	7	redika	17	08/12/2021	successful nes
2021_2022	27/01/2022	7	redika	31	22/12/2021	successful nes
2021_2022	27/01/2022	7	redika	22	22/12/2021	successful nes
2021_2022	27/01/2022	7	redika	63	05/01/2022	aborted bodyp
2021_2022	27/01/2022	7	redika	35	05/01/2022	successful nes
2021_2022	27/01/2022	7	redika	67	05/01/2022	successful nes
2021_2022	27/01/2022	7	redika	70	05/01/2022	successful nes
2021_2022	27/01/2022	7	atire	12	27/01/2022	successful nes
2021_2022	27/01/2022	7	atire	17	27/01/2022	successful nes
2021_2022	27/01/2022	7	atire	31	27/01/2022	successful nes
2021_2022	27/01/2022	7	atire	10	27/01/2022	successful nes
2021_2022	27/01/2022	7	atire	41	27/01/2022	successful nes
2021_2022	27/01/2022	7	atire	"bois"	27/01/2022	successful nes

2021_2022	27/01/2022	7	atire	"bois"	27/01/2022	successful nes
2021_2022	27/01/2022	7	atire	64	27/01/2022	successful nes
2021_2022	27/01/2022	7	atire	"bois"	27/01/2022	successful nes
2021_2022	27/01/2022	7	atire	"bois"	27/01/2022	successful nes
2021_2022	27/01/2022	7	atire	1	21/12/2021	successful nes
2021_2022	27/01/2022	7	atire	46	21/12/2021	successful nes
2021_2022	27/01/2022	7	atire	31	04/01/2022	successful nes
2021_2022	27/01/2022	7	atire	34	04/01/2022	successful nes
2021_2022	27/01/2022	7	atire	30	04/01/2022	successful nes
2021_2022	27/01/2022	7	atire	41	04/01/2022	successful nes
2021_2022	27/01/2022	7	atire	5	04/01/2022	successful nes
2021_2022	27/01/2022	7	atire	9	04/01/2022	successful nes
2021_2022	27/01/2022	7	atire	"bambou"	04/01/2022	successful nes
2021_2022	14/02/2022	8	atire	64	14/02/2022	successful nes
2021_2022	14/02/2022	8	atire	41	14/02/2022	successful nes
2021_2022	14/02/2022	8	atire	30	14/02/2022	successful nes
2021_2022	14/02/2022	8	atire	10	14/02/2022	successful nes
2021_2022	14/02/2022	8	atire	12	27/01/2022	successful nes
2021_2022	14/02/2022	8	atire	17	27/01/2022	successful nes
2021_2022	14/02/2022	8	atire	31	27/01/2022	successful nes
2021_2022	14/02/2022	8	atire	10	27/01/2022	successful nes
2021_2022	14/02/2022	8	atire	41	27/01/2022	successful nes
2021_2022	14/02/2022	8	atire	"bois"	27/01/2022	successful nes
2021_2022	14/02/2022	8	atire	"bois"	27/01/2022	successful nes
2021_2022	14/02/2022	8	atire	64	27/01/2022	successful nes
2021_2022	14/02/2022	8	atire	"bois"	27/01/2022	successful nes
2021_2022	14/02/2022	8	atire	"bois"	27/01/2022	successful nes
2021_2022	14/02/2022	8	atire	1	21/12/2021	successful nes
2021_2022	14/02/2022	8	atire	46	21/12/2021	successful nes
2021_2022	14/02/2022	8	atire	31	04/01/2022	successful nes
2021_2022	14/02/2022	8	atire	34	04/01/2022	successful nes
2021_2022	14/02/2022	8	atire	30	04/01/2022	successful nes
2021_2022	14/02/2022	8	atire	5	04/01/2022	successful nes
2021_2022	14/02/2022	8	atire	9	04/01/2022	successful nes
2021_2022	14/02/2022	8	atire	"bambou"	04/01/2022	successful nes
2021_2022	14/02/2022	8	vua	459	14/02/2022	aborted bodyp
2021_2022	14/02/2022	8	vua	1	04/01/2022	successful nes
2021_2022	14/02/2022	8	vua	47	04/01/2022	successful nes
2021_2022	14/02/2022	8	vua	"sternes"	04/01/2022	successful nes
2021_2022	14/02/2022	8	vua	60	26/01/2022	successful nes
2021_2022	14/02/2022	8	vua	38	26/01/2022	successful nes
2021_2022	14/02/2022	8	vua	42	26/01/2022	successful nes
2021_2022	14/02/2022	8	vua	19	26/01/2022	successful nes
2021_2022	14/02/2022	8	vua	53	26/01/2022	successful nes
2021_2022	14/02/2022	8	vua	32	26/01/2022	successful nes
2021_2022	14/02/2022	8	ieroue	25	26/01/2022	successful nes
2021_2022	14/02/2022	8	ieroue	356	26/01/2022	successful nes
2021_2022	14/02/2022	8	kouare	24	27/01/2022	successful nes
2021_2022	14/02/2022	8	kouare	44	27/01/2022	successful nes
2021_2022	14/02/2022	8	kouare	"panneau kite	27/01/2022	successful nes

2021_2022	14/02/2022	8	kouare	66	21/12/2021	successful nes
2021_2022	14/02/2022	8	kouare	51	04/01/2022	successful nes
2021_2022	14/02/2022	8	kouare	4	04/01/2022	aborted bodyp
2021_2022	14/02/2022	8	nda	58	27/01/2022	successful nes
2021_2022	14/02/2022	8	nda	11	21/12/2021	successful nes
2021_2022	14/02/2022	8	nda	22	04/01/2022	successful nes
2021_2022	15/02/2022	8	gi	9	15/02/2022	successful nes
2021_2022	15/02/2022	8	gi	7	15/02/2022	successful nes
2021_2022	15/02/2022	8	gi	43	18/11/2021	successful nes
2021_2022	15/02/2022	8	gi	14	07/12/2021	successful nes
2021_2022	15/02/2022	8	gi	3	07/12/2021	successful nes
2021_2022	15/02/2022	8	gi	54	07/12/2021	successful nes
2021_2022	15/02/2022	8	gi	49	07/12/2021	successful nes
2021_2022	15/02/2022	8	gi	33	07/12/2021	successful nes
2021_2022	15/02/2022	8	gi	68	22/12/2021	successful nes
2021_2022	15/02/2022	8	gi	95	22/12/2021	successful nes
2021_2022	15/02/2022	8	gi	9	05/01/2022	aborted bodyp
2021_2022	15/02/2022	8	gi	32	05/01/2022	successful nes
2021_2022	15/02/2022	8	gi	52	05/01/2022	successful nes
2021_2022	15/02/2022	8	gi	21B	05/01/2022	successful nes
2021_2022	15/02/2022	8	gi	51	05/01/2022	successful nes
2021_2022	15/02/2022	8	gi	27	26/01/2022	successful nes
2021_2022	15/02/2022	8	gi	21	26/01/2022	successful nes
2021_2022	15/02/2022	8	gi	55	26/01/2022	successful nes
2021_2022	15/02/2022	8	gi	21	26/01/2022	successful nes
2021_2022	15/02/2022	8	gi	21	26/01/2022	successful nes
2021_2022	15/02/2022	8	gi	35	26/01/2022	successful nes
2021_2022	15/02/2022	8	gi	36	26/01/2022	successful nes
2021_2022	15/02/2022	8	gi	"bois"	26/01/2022	successful nes
2021_2022	15/02/2022	8	gi	"bambou"	26/01/2022	successful nes
2021_2022	15/02/2022	8	gi	"big bambou e	26/01/2022	successful nes
2021_2022	15/02/2022	8	gi	7	26/01/2022	successful nes
2021_2022	15/02/2022	8	uaterembi	9	21/12/2021	successful nes
2021_2022	15/02/2022	8	uaterembi	34	21/12/2021	successful nes
2021_2022	15/02/2022	8	uaterembi	61	04/01/2022	successful nes
2021_2022	15/02/2022	8	uaterembi	68	04/01/2022	successful nes
2021_2022	15/02/2022	8	uaterembi	"bois"	26/01/2022	successful nes
2021_2022	15/02/2022	8	uaterembi	6	26/01/2022	successful nes
2021_2022	15/02/2022	8	uaterembi	2	26/01/2022	successful nes
2021_2022	15/02/2022	8	uaterembi	41	26/01/2022	successful nes
2021_2022	15/02/2022	8	uatio	23	22/12/2021	successful nes
2021_2022	15/02/2022	8	uatio	46	05/01/2022	successful nes
2021_2022	15/02/2022	8	uatio	28	05/01/2022	successful nes
2021_2022	15/02/2022	8	uatio	7	05/01/2022	aborted bodyp
2021_2022	15/02/2022	8	uatio	11	26/01/2022	successful nes
2021_2022	15/02/2022	8	ua	464	15/02/2022	successful nes
2021_2022	15/02/2022	8	ua	25	21/12/2021	successful nes
2021_2022	16/02/2022	9	nge	466	16/02/2022	successful nes
2021_2022	16/02/2022	9	nge	467	16/02/2022	successful nes
2021_2022	16/02/2022	9	nge	468	16/02/2022	successful nes

2021_2022	16/02/2022	9	nge	470	16/02/2022	successful nes
2021_2022	16/02/2022	9	nge	471	16/02/2022	successful nes
2021_2022	16/02/2022	9	nge	474	16/02/2022	successful nes
2021_2022	16/02/2022	9	nge	15	27/01/2022	successful nes
2021_2022	16/02/2022	9	nge	39	27/01/2022	successful nes
2021_2022	16/02/2022	9	nge	67	27/01/2022	successful nes
2021_2022	16/02/2022	9	nge	40	27/01/2022	successful nes
2021_2022	16/02/2022	9	nge	25	27/01/2022	successful nes
2021_2022	16/02/2022	9	nge	13	27/01/2022	successful nes
2021_2022	16/02/2022	9	nge	27	07/12/2021	successful nes
2021_2022	16/02/2022	9	nge	4	07/12/2021	successful nes
2021_2022	16/02/2022	9	nge	18	07/12/2021	successful nes
2021_2022	16/02/2022	9	nge	65	07/12/2021	successful nes
2021_2022	16/02/2022	9	nge	30	22/12/2021	successful nes
2021_2022	16/02/2022	9	nge	67	22/12/2021	successful nes
2021_2022	16/02/2022	9	nge	70	22/12/2021	successful nes
2021_2022	16/02/2022	9	nge	42	22/12/2021	successful nes
2021_2022	16/02/2022	9	nge	23	22/12/2021	successful nes
2021_2022	16/02/2022	9	nge	11	22/12/2021	successful nes
2021_2022	16/02/2022	9	nge	37	04/01/2022	successful nes
2021_2022	16/02/2022	9	nge	8	04/01/2022	successful nes
2021_2022	16/02/2022	9	nge	3	04/01/2022	successful nes
2021_2022	16/02/2022	9	nge	59	04/01/2022	successful nes
2021_2022	16/02/2022	9	nge	20	04/01/2022	successful nes
2021_2022	16/02/2022	9	nge	56	04/01/2022	successful nes
2021_2022	16/02/2022	9	nge	5	04/01/2022	successful nes
2021_2022	18/02/2022	9	redika	22	22/12/2021	successful nes
2021_2022	18/02/2022	9	redika	63	05/01/2022	aborted bodyp
2021_2022	18/02/2022	9	redika	35	05/01/2022	successful nes
2021_2022	18/02/2022	9	redika	67	05/01/2022	successful nes
2021_2022	18/02/2022	9	redika	70	05/01/2022	successful nes
2021_2022	18/02/2022	9	redika	62	27/01/2022	successful nes
2021_2022	23/02/2022	10	gi	54	07/12/2021	successful nes
2021_2022	23/02/2022	10	gi	49	07/12/2021	successful nes
2021_2022	23/02/2022	10	gi	33	07/12/2021	successful nes
2021_2022	23/02/2022	10	gi	68	22/12/2021	successful nes
2021_2022	23/02/2022	10	gi	21	26/01/2022	successful nes
2021_2022	23/02/2022	10	gi	55	26/01/2022	successful nes
2021_2022	23/02/2022	10	gi	21	26/01/2022	successful nes
2021_2022	23/02/2022	10	gi	21	26/01/2022	successful nes
2021_2022	23/02/2022	10	gi	36	26/01/2022	successful nes
2021_2022	23/02/2022	10	gi	"bambou"	26/01/2022	successful nes
2021_2022	23/02/2022	10	gi	9	15/02/2022	successful nes
2021_2022	23/02/2022	10	gi	7	15/02/2022	successful nes
2021_2022	23/02/2022	10	nge	25	27/01/2022	successful nes
2021_2022	23/02/2022	10	nge	65	07/12/2021	successful nes
2021_2022	10/03/2022	11	ieroue	25	26/01/2022	successful nes
2021_2022	10/03/2022	11	ua	464	15/02/2022	successful nes
2021_2022	10/03/2022	11	nge	25	27/01/2022	successful nes
2021_2022	10/03/2022	11	nge	65	07/12/2021	successful nes

2021_2022	23/02/2022	10	gi		486	23/02/2022	successful nes
2021_2022	23/02/2022	10	gi		487	23/02/2022	successful nes
2021_2022	10/03/2022	11	gi		486	23/02/2022	successful nes
2021_2022	10/03/2022	11	gi		487	23/02/2022	successful nes
2021_2022	11/03/2022	11	kouare		24	27/01/2022	successful nes
2021_2022	11/03/2022	11	kouare		4	04/01/2022	aborted bodyp
2021_2022	11/03/2022	11	vua		459	14/02/2022	aborted bodyp
2021_2022	11/03/2022	11	vua		53	26/01/2022	successful nes
2021_2022	11/03/2022	11	redika		22	22/12/2021	successful nes
2021_2022	11/03/2022	11	redika		67	05/01/2022	successful nes
2021_2022	11/03/2022	11	redika		70	05/01/2022	successful nes
2021_2022	11/03/2022	11	atire	"bois"		27/01/2022	successful nes
2021_2022	11/03/2022	11	atire	"bois"		27/01/2022	successful nes
2021_2022	11/03/2022	11	atire		64	27/01/2022	successful nes
2021_2022	11/03/2022	11	atire		41	27/01/2022	successful nes
2021_2022	11/03/2022	11	atire		30	04/01/2022	successful nes
2021_2022	11/03/2022	11	atire		10	27/01/2022	successful nes
2022_2023	22/11/2022	1	atire		29	22/11/2022	successful nes
2022_2023	22/11/2022	1	vua		21	22/11/2022	successful nes
2022_2023	22/11/2022	1	vua		26	22/11/2022	successful nes
2022_2023	22/11/2022	1	nda		20	22/11/2022	successful nes
2022_2023	22/11/2022	1	nda		34	22/11/2022	aborted bodyp
2022_2023	22/11/2022	1	nda		18	22/11/2022	successful nes
2022_2023	22/11/2022	1	kouare		4	22/11/2022	successful nes
2022_2023	22/11/2022	1	gi		9	22/11/2022	successful nes
2022_2023	22/11/2022	1	gi		3	22/11/2022	successful nes
2022_2023	23/11/2022	1	uaterembi		22	23/11/2022	successful nes
2022_2023	23/11/2022	1	uatio		1	23/11/2022	successful nes
2022_2023	23/11/2022	1	uatio		24	23/11/2022	successful nes
2022_2023	23/11/2022	1	uatio		25	23/11/2022	successful nes
2022_2023	23/11/2022	1	uatio		36	23/11/2022	successful nes
2022_2023	23/11/2022	1	ua		15	23/11/2022	successful nes
2022_2023	23/11/2022	1	nge		12	23/11/2022	successful nes
2022_2023	23/11/2022	1	nge		23	23/11/2022	successful nes
2022_2023	23/11/2022	1	ieroue		7	23/11/2022	successful nes
2022_2023	23/11/2022	1	ieroue		5	23/11/2022	successful nes
2022_2023	23/11/2022	1	ieroue		35	23/11/2022	successful nes
2022_2023	05/12/2022	2	kie	nd		05/12/2022	successful nes
2022_2023	05/12/2022	2	kie	nd		05/12/2022	successful nes
2022_2023	05/12/2022	2	kie	nd		05/12/2022	successful nes
2022_2023	05/12/2022	2	kie	nd		05/12/2022	successful nes
2022_2023	05/12/2022	2	amere	nd		05/12/2022	successful nes
2022_2023	05/12/2022	2	amere	nd		05/12/2022	successful nes
2022_2023	05/12/2022	2	amere	nd		05/12/2022	successful nes
2022_2023	05/12/2022	2	amere	nd		05/12/2022	successful nes
2022_2023	05/12/2022	2	amere	nd		05/12/2022	successful nes
2022_2023	07/12/2022	3	vua		33	07/12/2022	successful nes
2022_2023	07/12/2022	3	vua		48	07/12/2022	successful nes
2022_2023	07/12/2022	3	vua		64	07/12/2022	successful nes
2022_2023	07/12/2022	3	vua		63	07/12/2022	successful nes

2022_2023	07/12/2022	3	vua	59	07/12/2022 successful nes
2022_2023	07/12/2022	3	vua	21	22/11/2022 successful nes
2022_2023	07/12/2022	3	vua	26	22/11/2022 successful nes
2022_2023	07/12/2022	3	uatio	49	07/12/2022 successful nes
2022_2023	07/12/2022	3	uatio	1	23/11/2022 successful nes
2022_2023	07/12/2022	3	uatio	24	23/11/2022 successful nes
2022_2023	07/12/2022	3	uatio	25	23/11/2022 successful nes
2022_2023	07/12/2022	3	uatio	36	23/11/2022 successful nes
2022_2023	07/12/2022	3	kouare	53	07/12/2022 successful nes
2022_2023	07/12/2022	3	kouare	41	07/12/2022 successful nes
2022_2023	07/12/2022	3	kouare	62	07/12/2022 successful nes
2022_2023	07/12/2022	3	kouare	28	07/12/2022 successful nes
2022_2023	07/12/2022	3	kouare	54	07/12/2022 aborted bodyp
2022_2023	07/12/2022	3	kouare	58	07/12/2022 successful nes
2022_2023	07/12/2022	3	kouare	4	22/11/2022 successful nes
2022_2023	07/12/2022	3	nda	72	07/12/2022 aborted bodyp
2022_2023	07/12/2022	3	nda	17	07/12/2022 successful nes
2022_2023	07/12/2022	3	nda	24	07/12/2022 aborted bodyp
2022_2023	07/12/2022	3	nda	10	07/12/2022 successful nes
2022_2023	07/12/2022	3	nda	32	07/12/2022 successful nes
2022_2023	07/12/2022	3	nda	52	07/12/2022 successful nes
2022_2023	07/12/2022	3	nda	20	22/11/2022 successful nes
2022_2023	07/12/2022	3	nda	34	22/11/2022 aborted bodyp
2022_2023	07/12/2022	3	nda	18	22/11/2022 successful nes
2022_2023	08/12/2022	3	nge	51	08/12/2022 successful nes
2022_2023	08/12/2022	3	nge	2	08/12/2022 successful nes
2022_2023	08/12/2022	3	nge	46	08/12/2022 successful nes
2022_2023	08/12/2022	3	nge	12	23/11/2022 successful nes
2022_2023	08/12/2022	3	nge	23	23/11/2022 successful nes
2022_2023	08/12/2022	3	gi	16	08/12/2022 successful nes
2022_2023	08/12/2022	3	gi	9	22/11/2022 successful nes
2022_2023	08/12/2022	3	gi	3	22/11/2022 successful nes
2022_2023	08/12/2022	3	ua	76	08/12/2022 successful nes
2022_2023	08/12/2022	3	ua	12	08/12/2022 successful nes
2022_2023	08/12/2022	3	ua	8	08/12/2022 successful nes
2022_2023	08/12/2022	3	ua	15	23/11/2022 successful nes
2022_2023	08/12/2022	3	ieroue	57	08/12/2022 successful nes
2022_2023	08/12/2022	3	ieroue	50	08/12/2022 successful nes
2022_2023	08/12/2022	3	ieroue	67	08/12/2022 successful nes
2022_2023	08/12/2022	3	ieroue	7	23/11/2022 successful nes
2022_2023	08/12/2022	3	ieroue	5	23/11/2022 successful nes
2022_2023	08/12/2022	3	ieroue	35	23/11/2022 successful nes
2022_2023	15/12/2022	4	uaterembi	22	15/12/2022 successful nes
2022_2023	15/12/2022	4	uaterembi	22	23/11/2022 successful nes
2022_2023	19/12/2022	5	ieroue	74	19/12/2022 successful nes
2022_2023	19/12/2022	5	ieroue	66	19/12/2022 successful nes
2022_2023	19/12/2022	5	ieroue	7	23/11/2022 successful nes
2022_2023	19/12/2022	5	ieroue	5	23/11/2022 successful nes
2022_2023	19/12/2022	5	ieroue	35	23/11/2022 successful nes
2022_2023	19/12/2022	5	ieroue	57	08/12/2022 successful nes

2022_2023	19/12/2022	5	ieroue		50	08/12/2022	successful nes
2022_2023	19/12/2022	5	ieroue		67	08/12/2022	successful nes
2022_2023	19/12/2022	5	kouare		61	19/12/2022	aborted bodyp
2022_2023	19/12/2022	5	kouare		31	19/12/2022	successful nes
2022_2023	19/12/2022	5	kouare		4	22/11/2022	successful nes
2022_2023	19/12/2022	5	kouare		53	07/12/2022	successful nes
2022_2023	19/12/2022	5	kouare		41	07/12/2022	successful nes
2022_2023	19/12/2022	5	kouare		62	07/12/2022	successful nes
2022_2023	19/12/2022	5	kouare		28	07/12/2022	successful nes
2022_2023	19/12/2022	5	kouare		54	07/12/2022	aborted bodyp
2022_2023	19/12/2022	5	kouare		58	07/12/2022	successful nes
2022_2023	19/12/2022	5	nda		19	19/12/2022	successful nes
2022_2023	19/12/2022	5	nda		40	19/12/2022	successful nes
2022_2023	19/12/2022	5	nda		20	22/11/2022	successful nes
2022_2023	19/12/2022	5	nda		34	22/11/2022	aborted bodyp
2022_2023	19/12/2022	5	nda		18	22/11/2022	successful nes
2022_2023	19/12/2022	5	nda		72	07/12/2022	aborted bodyp
2022_2023	19/12/2022	5	nda		17	07/12/2022	successful nes
2022_2023	19/12/2022	5	nda		24	07/12/2022	aborted bodyp
2022_2023	19/12/2022	5	nda		10	07/12/2022	successful nes
2022_2023	19/12/2022	5	nda		32	07/12/2022	successful nes
2022_2023	19/12/2022	5	nda		52	07/12/2022	successful nes
2022_2023	19/12/2022	5	vua		56	19/12/2022	successful nes
2022_2023	19/12/2022	5	vua		13	19/12/2022	successful nes
2022_2023	19/12/2022	5	vua		60	19/12/2022	successful nes
2022_2023	19/12/2022	5	vua		21	22/11/2022	successful nes
2022_2023	19/12/2022	5	vua		26	22/11/2022	successful nes
2022_2023	19/12/2022	5	vua		33	07/12/2022	successful nes
2022_2023	19/12/2022	5	vua		48	07/12/2022	successful nes
2022_2023	19/12/2022	5	vua		64	07/12/2022	successful nes
2022_2023	19/12/2022	5	vua		63	07/12/2022	successful nes
2022_2023	19/12/2022	5	vua		59	07/12/2022	successful nes
2022_2023	19/12/2022	5	atire		83	19/12/2022	successful nes
2022_2023	19/12/2022	5	atire		85	19/12/2022	successful nes
2022_2023	19/12/2022	5	atire		64	19/12/2022	successful nes
2022_2023	19/12/2022	5	atire		30	19/12/2022	successful nes
2022_2023	19/12/2022	5	atire		39	19/12/2022	successful nes
2022_2023	19/12/2022	5	atire		75	19/12/2022	successful nes
2022_2023	19/12/2022	5	atire		73	19/12/2022	successful nes
2022_2023	19/12/2022	5	atire		55	19/12/2022	successful nes
2022_2023	19/12/2022	5	atire		38	19/12/2022	successful nes
2022_2023	19/12/2022	5	atire		29	22/11/2022	successful nes
2022_2023	22/12/2022	6	kie	nd		05/12/2022	successful nes
2022_2023	22/12/2022	6	kie	nd		05/12/2022	successful nes
2022_2023	22/12/2022	6	kie	nd		05/12/2022	successful nes
2022_2023	22/12/2022	6	kie	nd		05/12/2022	successful nes
2022_2023	22/12/2022	6	amere	nd		05/12/2022	successful nes
2022_2023	22/12/2022	6	amere	nd		05/12/2022	successful nes
2022_2023	22/12/2022	6	amere	nd		05/12/2022	successful nes
2022_2023	22/12/2022	6	amere	nd		05/12/2022	successful nes

2022_2023	22/12/2022	6	amere	nd		05/12/2022	successful nes
2022_2023	29/12/2022	7	nge		51	08/12/2022	successful nes
2022_2023	29/12/2022	7	nge		2	08/12/2022	successful nes
2022_2023	29/12/2022	7	nge		46	08/12/2022	successful nes
2022_2023	29/12/2022	7	nge		12	23/11/2022	successful nes
2022_2023	03/01/2023	8	ua		15	23/11/2022	successful nes
2022_2023	03/01/2023	8	ua		76	08/12/2022	successful nes
2022_2023	03/01/2023	8	ua		12	08/12/2022	successful nes
2022_2023	03/01/2023	8	ua		8	08/12/2022	successful nes
2022_2023	03/01/2023	8	uaterembi		22	15/12/2022	successful nes
2022_2023	04/01/2023	8	gi		9	22/11/2022	successful nes
2022_2023	04/01/2023	8	gi		3	22/11/2022	successful nes
2022_2023	04/01/2023	8	uatio		1	23/11/2022	successful nes
2022_2023	04/01/2023	8	uatio		25	23/11/2022	successful nes
2022_2023	04/01/2023	8	uatio		36	23/11/2022	successful nes
2022_2023	04/01/2023	8	uatio		49	07/12/2022	successful nes
2022_2023	11/01/2023	9	ieroue		74	19/12/2022	successful nes
2022_2023	11/01/2023	9	ieroue		66	19/12/2022	successful nes
2022_2023	11/01/2023	9	ieroue		7	23/11/2022	successful nes
2022_2023	11/01/2023	9	ieroue		5	23/11/2022	successful nes
2022_2023	11/01/2023	9	ieroue		35	23/11/2022	successful nes
2022_2023	11/01/2023	9	ieroue		57	08/12/2022	successful nes
2022_2023	11/01/2023	9	ieroue		50	08/12/2022	successful nes
2022_2023	11/01/2023	9	ieroue		67	08/12/2022	successful nes
2022_2023	11/01/2023	9	nge		51	08/12/2022	successful nes
2022_2023	11/01/2023	9	nge		2	08/12/2022	successful nes
2022_2023	11/01/2023	9	nge		46	08/12/2022	successful nes
2022_2023	11/01/2023	9	nge		12	23/11/2022	successful nes
2022_2023	11/01/2023	9	nge		23	23/11/2022	successful nes
2022_2023	11/01/2023	9	gi		9	22/11/2022	successful nes
2022_2023	11/01/2023	9	gi		16	08/12/2022	successful nes
2022_2023	11/01/2023	9	gi		66	11/01/2023	successful nes
2022_2023	11/01/2023	9	gi		23	11/01/2023	successful nes
2022_2023	11/01/2023	9	gi		35	11/01/2023	successful nes
2022_2023	11/01/2023	9	gi		3	11/01/2023	successful nes
2022_2023	11/01/2023	9	nge		69	11/01/2023	successful nes
2022_2023	11/01/2023	9	nge		45	11/01/2023	successful nes
2022_2023	11/01/2023	9	nge		50	11/01/2023	successful nes
2022_2023	11/01/2023	9	nge		68	11/01/2023	successful nes
2022_2023	11/01/2023	9	nge		42	11/01/2023	successful nes
2022_2023	11/01/2023	9	nge		46	11/01/2023	successful nes
2022_2023	11/01/2023	9	ieroue		67	11/01/2023	successful nes
2022_2023	11/01/2023	9	nda		76	11/01/2023	successful nes
2022_2023	11/01/2023	9	nda		15	11/01/2023	successful nes
2022_2023	11/01/2023	9	nda		28	11/01/2023	successful nes
2022_2023	11/01/2023	9	nda		19	11/01/2023	successful nes
2022_2023	11/01/2023	9	nda		11	11/01/2023	successful nes
2022_2023	11/01/2023	9	nda		32	11/01/2023	successful nes
2022_2023	11/01/2023	9	nda		17	11/01/2023	successful nes
2022_2023	11/01/2023	9	nda		19	19/12/2022	successful nes

2022_2023	11/01/2023	9	nda	40	19/12/2022	successful nes
2022_2023	11/01/2023	9	nda	20	22/11/2022	successful nes
2022_2023	11/01/2023	9	nda	34	22/11/2022	aborted bodyp
2022_2023	11/01/2023	9	nda	18	22/11/2022	successful nes
2022_2023	11/01/2023	9	nda	72	07/12/2022	aborted bodyp
2022_2023	11/01/2023	9	nda	17	07/12/2022	successful nes
2022_2023	11/01/2023	9	nda	24	07/12/2022	aborted bodyp
2022_2023	11/01/2023	9	nda	10	07/12/2022	successful nes
2022_2023	11/01/2023	9	nda	32	07/12/2022	successful nes
2022_2023	11/01/2023	9	nda	52	07/12/2022	successful nes
2022_2023	11/01/2023	9	kouare	44	11/01/2023	successful nes
2022_2023	11/01/2023	9	kouare	18	11/01/2023	successful nes
2022_2023	11/01/2023	9	kouare	40	11/01/2023	successful nes
2022_2023	11/01/2023	9	kouare	61	19/12/2022	aborted bodyp
2022_2023	11/01/2023	9	kouare	31	19/12/2022	successful nes
2022_2023	11/01/2023	9	kouare	4	22/11/2022	successful nes
2022_2023	11/01/2023	9	kouare	53	07/12/2022	successful nes
2022_2023	11/01/2023	9	kouare	41	07/12/2022	successful nes
2022_2023	11/01/2023	9	kouare	62	07/12/2022	successful nes
2022_2023	11/01/2023	9	kouare	54	07/12/2022	aborted bodyp
2022_2023	11/01/2023	9	kouare	58	07/12/2022	successful nes
2022_2023	12/01/2023	9	uatio	6	12/01/2023	successful nes
2022_2023	12/01/2023	9	uatio	58	12/01/2023	successful nes
2022_2023	12/01/2023	9	uatio	1	23/11/2022	successful nes
2022_2023	12/01/2023	9	uatio	25	23/11/2022	successful nes
2022_2023	12/01/2023	9	uatio	36	23/11/2022	successful nes
2022_2023	12/01/2023	9	uatio	49	07/12/2022	successful nes
2022_2023	12/01/2023	9	uaterembi	22	15/12/2022	successful nes
2022_2023	12/01/2023	9	uaterembi	54	03/01/2023	successful nes
2022_2023	12/01/2023	9	uaterembi	62	12/01/2023	successful nes
2022_2023	12/01/2023	9	uaterembi	4	12/01/2023	successful nes
2022_2023	12/01/2023	9	vua	41	12/01/2023	successful nes
2022_2023	12/01/2023	9	vua	14	12/01/2023	successful nes
2022_2023	12/01/2023	9	vua	14	12/01/2023	successful nes
2022_2023	12/01/2023	9	vua	48	12/01/2023	successful nes
2022_2023	12/01/2023	9	vua	56	12/01/2023	successful nes
2022_2023	12/01/2023	9	vua	26	12/01/2023	successful nes
2022_2023	12/01/2023	9	vua	56	19/12/2022	successful nes
2022_2023	12/01/2023	9	vua	13	19/12/2022	successful nes
2022_2023	12/01/2023	9	vua	60	19/12/2022	successful nes
2022_2023	12/01/2023	9	vua	21	22/11/2022	successful nes
2022_2023	12/01/2023	9	vua	26	22/11/2022	successful nes
2022_2023	12/01/2023	9	vua	33	07/12/2022	successful nes
2022_2023	12/01/2023	9	vua	48	07/12/2022	successful nes
2022_2023	12/01/2023	9	vua	63	07/12/2022	successful nes
2022_2023	12/01/2023	9	vua	59	07/12/2022	successful nes
2022_2023	12/01/2023	9	atire	21	12/01/2023	successful nes
2022_2023	12/01/2023	9	atire	13	12/01/2023	successful nes
2022_2023	12/01/2023	9	atire	60	12/01/2023	successful nes
2022_2023	12/01/2023	9	atire	83	12/01/2023	successful nes

2022_2023	12/01/2023	9	atire	29	12/01/2023	successful nes
2022_2023	12/01/2023	9	atire	55	12/01/2023	successful nes
2022_2023	12/01/2023	9	atire	29	22/11/2022	successful nes
2022_2023	12/01/2023	9	atire	83	19/12/2022	successful nes
2022_2023	12/01/2023	9	atire	85	19/12/2022	successful nes
2022_2023	12/01/2023	9	atire	64	19/12/2022	successful nes
2022_2023	12/01/2023	9	atire	30	19/12/2022	successful nes
2022_2023	12/01/2023	9	atire	39	19/12/2022	successful nes
2022_2023	12/01/2023	9	atire	75	19/12/2022	successful nes
2022_2023	12/01/2023	9	atire	73	19/12/2022	successful nes
2022_2023	12/01/2023	9	atire	55	19/12/2022	successful nes
2022_2023	12/01/2023	9	atire	38	19/12/2022	successful nes
2022_2023	25/01/2023	10	ieroue	47	25/01/2023	successful nes
2022_2023	25/01/2023	10	ieroue	74	19/12/2022	successful nes
2022_2023	25/01/2023	10	ieroue	7	23/11/2022	successful nes
2022_2023	25/01/2023	10	ieroue	5	23/11/2022	successful nes
2022_2023	25/01/2023	10	ieroue	57	08/12/2022	successful nes
2022_2023	25/01/2023	10	ieroue	67	11/01/2023	successful nes
2022_2023	25/01/2023	10	uaterembi	22	15/12/2022	successful nes
2022_2023	25/01/2023	10	uaterembi	54	03/01/2023	successful nes
2022_2023	25/01/2023	10	uaterembi	62	12/01/2023	successful nes
2022_2023	25/01/2023	10	uaterembi	4	12/01/2023	successful nes
2022_2023	25/01/2023	10	uatio	6	12/01/2023	successful nes
2022_2023	25/01/2023	10	uatio	58	12/01/2023	successful nes
2022_2023	25/01/2023	10	uatio	1	23/11/2022	successful nes
2022_2023	25/01/2023	10	uatio	25	23/11/2022	successful nes
2022_2023	25/01/2023	10	uatio	36	23/11/2022	successful nes
2022_2023	25/01/2023	10	uatio	49	07/12/2022	successful nes
2022_2023	25/01/2023	10	uatio	81	25/01/2023	successful nes
2022_2023	25/01/2023	10	uatio	82	25/01/2023	successful nes
2022_2023	25/01/2023	10	gi	76	25/01/2023	successful nes
2022_2023	25/01/2023	10	gi	43	25/01/2023	successful nes
2022_2023	25/01/2023	10	gi	9	22/11/2022	successful nes
2022_2023	25/01/2023	10	gi	16	08/12/2022	successful nes
2022_2023	25/01/2023	10	gi	66	11/01/2023	successful nes
2022_2023	25/01/2023	10	gi	23	11/01/2023	successful nes
2022_2023	25/01/2023	10	gi	35	11/01/2023	successful nes
2022_2023	25/01/2023	10	gi	3	11/01/2023	successful nes
2022_2023	25/01/2023	10	tere	66	25/01/2023	successful nes
2022_2023	25/01/2023	10	nda	86	25/01/2023	successful nes
2022_2023	25/01/2023	10	nda	27	25/01/2023	successful nes
2022_2023	25/01/2023	10	nda	76	11/01/2023	successful nes
2022_2023	25/01/2023	10	nda	15	11/01/2023	successful nes
2022_2023	25/01/2023	10	nda	28	11/01/2023	successful nes
2022_2023	25/01/2023	10	nda	19	11/01/2023	successful nes
2022_2023	25/01/2023	10	nda	11	11/01/2023	successful nes
2022_2023	25/01/2023	10	nda	32	11/01/2023	successful nes
2022_2023	25/01/2023	10	nda	17	11/01/2023	successful nes
2022_2023	25/01/2023	10	nda	20	22/11/2022	successful nes
2022_2023	25/01/2023	10	nda	34	22/11/2022	aborted bodyp

2022_2023	25/01/2023	10	nda	72	07/12/2022	aborted bodyp
2022_2023	25/01/2023	10	nda	24	07/12/2022	aborted bodyp
2022_2023	25/01/2023	10	nda	52	07/12/2022	successful nes
2022_2023	26/01/2023	10	nge	10	26/01/2023	successful nes
2022_2023	26/01/2023	10	nge	51	08/12/2022	successful nes
2022_2023	26/01/2023	10	nge	2	08/12/2022	successful nes
2022_2023	26/01/2023	10	nge	12	23/11/2022	successful nes
2022_2023	26/01/2023	10	nge	69	11/01/2023	successful nes
2022_2023	26/01/2023	10	nge	45	11/01/2023	successful nes
2022_2023	26/01/2023	10	nge	50	11/01/2023	successful nes
2022_2023	26/01/2023	10	nge	68	11/01/2023	successful nes
2022_2023	26/01/2023	10	nge	42	11/01/2023	successful nes
2022_2023	26/01/2023	10	nge	46	11/01/2023	successful nes
2022_2023	26/01/2023	10	kouare	44	11/01/2023	successful nes
2022_2023	26/01/2023	10	kouare	18	11/01/2023	successful nes
2022_2023	26/01/2023	10	kouare	40	11/01/2023	successful nes
2022_2023	26/01/2023	10	kouare	61	19/12/2022	aborted bodyp
2022_2023	26/01/2023	10	kouare	31	19/12/2022	successful nes
2022_2023	26/01/2023	10	kouare	53	07/12/2022	successful nes
2022_2023	26/01/2023	10	ua	12	08/12/2022	successful nes
2022_2023	26/01/2023	10	ua	8	08/12/2022	successful nes
2022_2023	26/01/2023	10	vua	8	26/01/2023	successful nes
2022_2023	26/01/2023	10	vua	11	26/01/2023	successful nes
2022_2023	26/01/2023	10	vua	41	12/01/2023	successful nes
2022_2023	26/01/2023	10	vua	14	12/01/2023	successful nes
2022_2023	26/01/2023	10	vua	14	12/01/2023	successful nes
2022_2023	26/01/2023	10	vua	48	12/01/2023	successful nes
2022_2023	26/01/2023	10	vua	56	12/01/2023	successful nes
2022_2023	26/01/2023	10	vua	26	12/01/2023	successful nes
2022_2023	26/01/2023	10	vua	33	07/12/2022	successful nes
2022_2023	26/01/2023	10	vua	63	07/12/2022	successful nes
2022_2023	26/01/2023	10	vua	59	07/12/2022	successful nes
2022_2023	26/01/2023	10	atire	19	26/01/2023	successful nes
2022_2023	26/01/2023	10	atire	77	26/01/2023	successful nes
2022_2023	26/01/2023	10	atire	21	12/01/2023	successful nes
2022_2023	26/01/2023	10	atire	13	12/01/2023	successful nes
2022_2023	26/01/2023	10	atire	60	12/01/2023	successful nes
2022_2023	26/01/2023	10	atire	83	12/01/2023	successful nes
2022_2023	26/01/2023	10	atire	29	12/01/2023	successful nes
2022_2023	26/01/2023	10	atire	55	12/01/2023	successful nes
2022_2023	26/01/2023	10	atire	85	19/12/2022	successful nes
2022_2023	26/01/2023	10	atire	64	19/12/2022	successful nes
2022_2023	26/01/2023	10	atire	30	19/12/2022	successful nes
2022_2023	26/01/2023	10	atire	39	19/12/2022	successful nes
2022_2023	26/01/2023	10	atire	75	19/12/2022	successful nes
2022_2023	26/01/2023	10	atire	73	19/12/2022	successful nes
2022_2023	26/01/2023	10	atire	38	19/12/2022	successful nes
2022_2023	08/02/2023	11	ieroue	47	25/01/2023	successful nes
2022_2023	08/02/2023	11	ieroue	74	19/12/2022	successful nes
2022_2023	08/02/2023	11	ieroue	7	23/11/2022	successful nes

2022_2023	08/02/2023	11	ieroue	5	23/11/2022	successful nes
2022_2023	08/02/2023	11	ieroue	57	08/12/2022	successful nes
2022_2023	08/02/2023	11	ieroue	67	11/01/2023	successful nes
2022_2023	08/02/2023	11	uaterembi	22	15/12/2022	successful nes
2022_2023	08/02/2023	11	uaterembi	62	12/01/2023	successful nes
2022_2023	08/02/2023	11	uaterembi	4	12/01/2023	successful nes
2022_2023	08/02/2023	11	gi	9	22/11/2022	successful nes
2022_2023	08/02/2023	11	gi	23	11/01/2023	successful nes
2022_2023	08/02/2023	11	gi	35	11/01/2023	successful nes
2022_2023	08/02/2023	11	gi	3	11/01/2023	successful nes
2022_2023	08/02/2023	11	gi	76	25/01/2023	successful nes
2022_2023	08/02/2023	11	gi	43	25/01/2023	successful nes
2022_2023	08/02/2023	11	tere	66	25/01/2023	successful nes
2022_2023	08/02/2023	11	nda	20	22/11/2022	successful nes
2022_2023	08/02/2023	11	nda	34	22/11/2022	aborted bodyp
2022_2023	08/02/2023	11	nda	72	07/12/2022	aborted bodyp
2022_2023	08/02/2023	11	nda	24	07/12/2022	aborted bodyp
2022_2023	08/02/2023	11	nda	52	07/12/2022	successful nes
2022_2023	08/02/2023	11	nda	76	11/01/2023	successful nes
2022_2023	08/02/2023	11	nda	15	11/01/2023	successful nes
2022_2023	08/02/2023	11	nda	32	11/01/2023	successful nes
2022_2023	08/02/2023	11	nda	17	11/01/2023	successful nes
2022_2023	08/02/2023	11	nda	86	25/01/2023	successful nes
2022_2023	08/02/2023	11	nda	27	25/01/2023	successful nes
2022_2023	08/02/2023	11	kouare	61	19/12/2022	aborted bodyp
2022_2023	08/02/2023	11	kouare	31	19/12/2022	successful nes
2022_2023	08/02/2023	11	kouare	53	07/12/2022	successful nes
2022_2023	08/02/2023	11	kouare	44	11/01/2023	successful nes
2022_2023	08/02/2023	11	kouare	18	11/01/2023	successful nes
2022_2023	08/02/2023	11	kouare	40	11/01/2023	successful nes
2022_2023	15/02/2023	12	nge	10	11/01/2023	successful nes
2022_2023	15/02/2023	12	nge	51	11/01/2023	successful nes
2022_2023	15/02/2023	12	nge	2	11/01/2023	successful nes
2022_2023	15/02/2023	12	nge	12	11/01/2023	successful nes
2022_2023	15/02/2023	12	nge	69	11/01/2023	successful nes
2022_2023	15/02/2023	12	nge	45	11/01/2023	successful nes
2022_2023	15/02/2023	12	nge	50	11/01/2023	successful nes
2022_2023	15/02/2023	12	nge	68	11/01/2023	successful nes
2022_2023	15/02/2023	12	nge	42	11/01/2023	successful nes
2022_2023	15/02/2023	12	nge	46	11/01/2023	successful nes
2022_2023	15/02/2023	12	vua	8	11/01/2023	successful nes
2022_2023	15/02/2023	12	vua	11	11/01/2023	successful nes
2022_2023	15/02/2023	12	vua	41	11/01/2023	successful nes
2022_2023	15/02/2023	12	vua	14	11/01/2023	successful nes
2022_2023	15/02/2023	12	vua	14	11/01/2023	successful nes
2022_2023	15/02/2023	12	vua	48	11/01/2023	successful nes
2022_2023	15/02/2023	12	vua	56	11/01/2023	successful nes
2022_2023	15/02/2023	12	vua	26	11/01/2023	successful nes
2022_2023	15/02/2023	12	vua	33	11/01/2023	successful nes
2022_2023	15/02/2023	12	vua	63	11/01/2023	successful nes

new_activity	good_detectic	poor_detectic	no_detection	tag_left	tag_removed	tag_lost
nd	1			1	0	0
nd	1			1	0	0
nd	1			1	0	0
nd	1			1	0	0
nd	1			1	0	0
nd	1			1	0	0
nd	1			1	0	0
nd	1			1	0	0
nd	1			1	0	0
nd	1			1	0	0
nd	1			1	0	0
nd	1			1	0	0
nd		1		1	0	0
nd	1			1	0	0
nd	1			1	0	0
nd		1		0	1	0
nd	1			1	0	0
nd	1			1	0	0
nd	nd	nd	nd	0	0	1
nd		1		0	1	0
nd		1		0	1	0
nd	1			1	0	0
nd	1			1	0	0
nd	1			1	0	0
nd	1			1	0	0
nd		1		0	1	0
nd	1			1	0	0
nd	1			1	0	0
nd	1			1	0	0
nd	1			1	0	0
nd	nd	nd	nd	0	0	1
nd		1		0	1	0
nd		1		0	1	0
nd	nd	nd	nd	0	0	1
nd	1			1	0	0
nd		1		0	1	0
nd		1		1	0	0
nd		1		0	1	0
nd	1			1	0	0
nd	1			1	0	0
nd	1			1	0	0
nd	1			1	0	0
nd	1			1	0	0
nd	1			1	0	0
nd	nd	nd	nd	0	0	1
nd	nd	nd	nd	0	0	1
nd		1		0	1	0
nd	1			1	0	0
nd		1		1	0	0

nd		1			1	0	0
nd		1			1	0	0
nd	nd	nd	nd		0	0	1
nd		1			1	0	0
nd		1			1	0	0
nd		1			1	0	0
nd			1		0	1	0
nd				1	0	1	0
nd			1		1	0	0
nd				1	0	1	0
nd				1	0	1	0
nd		1			1	0	0
nd				1	0	1	0
nd			1		1	0	0
nd		1			1	0	0
nd		1			1	0	0
nd		1			1	0	0
nd				1	0	1	0
nd				1	0	1	0
nd		1			0	1	0
nd			1		0	1	0
nd	nd	nd	nd		0	0	1
nd			1		1	0	0
nd	nd	nd	nd		0	0	1
nd			1		0	1	0
nd			1		0	1	0
nd			1		0	1	0
nd				1	0	1	0
nd		1			0	1	0
nd				1	0	1	0
nd			1		0	1	0
nd		1			0	1	0
nd		1			0	1	0
nd		1			0	1	0
nd			1		0	1	0
nd			1		0	1	0
nd			1		0	1	0
nd		1			0	1	0
nd		1			0	1	0
nd		1			0	1	0
nd		1			0	1	0
nd		1			0	1	0
nd			1		0	1	0
nd			1		0	1	0
nd	nd	nd	nd		0	0	1
nd				1	0	1	0
nd			1		0	1	0
nd		1			0	1	0
nd			1		0	1	0
nd		1			0	1	0

nd		1		0	1	0
nd	1			0	1	0
nd		1		0	1	0
nd	1			0	1	0
nd			1	0	1	0
nd		1		0	1	0
nd	1			0	1	0
nd	1			0	1	0
nd		1		0	1	0
nd	1			0	1	0
nd	1			0	1	0
nd		1		0	1	0
nd		1		0	1	0
nd		1		0	1	0
nd		1		0	1	0
nd	1			0	1	0
nd	1			0	1	0
nd		1		0	1	0
nd	1			0	1	0
nd	1			0	1	0
nd		1		0	1	0
nd	1			0	1	0
nd		1		0	1	0
nd		1		0	1	0
nd		1		1	0	0
nd		1		0	1	0
nd	1			0	1	0
nd		1		0	1	0
nd		1		0	1	0
nd		1		0	1	0
nd	1			0	1	0
nd		1		0	1	0
nd		1		0	1	0
nd		1		0	1	0
nd		1		0	1	0
nd	1			0	1	0
nd		1		0	1	0
nd	1			0	1	0
nd		1		0	1	0
nd		1		0	1	0
nd		1		0	1	0
nd	1			1	0	0
nd	1			0	1	0
nd	1			0	1	0
nd	1			0	1	0
nd			1	0	1	0
nd	1			1	0	0
nd		1		1	0	0
nd		1		1	0	0
nd	1			1	0	0

nd		1		1	0	0
nd	1			0	1	0
nd	1			0	1	0
nd	1			0	1	0
nd	1			0	1	0
nd	1			0	1	0
nd	1			0	1	0
nd			1	0	1	0
nd	1			0	1	0
nd			1	0	1	0
nd	1			0	1	0
nd	1			0	1	0
nd		1		0	1	0
nd	1			0	1	0
nd			1	1	0	0
nd			1	0	1	0
nd	1			0	1	0
nd	1			0	1	0
nd	1			0	1	0
nd		1		0	1	0
nd	1			0	1	0
nd			1	0	1	0
nd			1	0	1	0
nd	1			0	1	0
nd	1			0	1	0
nd		1		1	0	0
nd	1			1	0	0
nd		1		1	0	0
nd		1		1	0	0
nd		1		1	0	0
nd			1	1	0	0
nd			1	0	1	0
1		1		0	0	0
1	1			0	0	0
1	1			0	0	0
0		1		1	0	0
1		1		0	0	0
1	1			0	0	0
1		1		0	0	0
0		1		1	0	0
1	nd	nd	nd	0	0	0
1	1			0	0	0
1		1		0	0	0
1		1		0	0	0
1		1		0	0	0
1	1			0	0	0
1	1			0	0	0
1	1			0	0	0
1	1			0	0	0
1	1			0	0	0

1	1			0	0	0
1	1			0	0	0
1		1		0	0	0
1		1		0	0	0
1		1		0	0	0
1	nd	nd	nd	0	0	0
1	nd	nd	nd	0	0	0
1	nd	nd	nd	0	0	0
1	nd	nd	nd	0	0	0
1	nd	nd	nd	0	0	0
1	nd	nd	nd	0	0	0
1	nd	nd	nd	0	0	0
0		1		1	0	0
0		1		0	1	0
0		1		1	0	0
1	nd	nd	nd	0	0	0
1	nd	nd	nd	0	0	0
1	nd	nd	nd	0	0	0
1	nd	nd	nd	0	0	0
1	nd	nd	nd	0	0	0
1	nd	nd	nd	0	0	0
1	nd	nd	nd	0	0	0
1	nd	nd	nd	0	0	0
1	nd	nd	nd	0	0	0
1	nd	nd	nd	0	0	0
1	nd	nd	nd	0	0	0
1	nd	nd	nd	0	0	0
1	nd	nd	nd	0	0	0
1	nd	nd	nd	0	0	0
0		1		1	0	0
0			1	0	1	0
1	1			0	0	0
1		1		0	0	0
1	1			0	0	0
1		1		0	0	0
0		1		0	1	0
0			1	0	1	0
0	1			1	0	0
0			1	0	1	0
0		1		1	0	0
0			1	0	1	0
0		1		1	0	0
0		1		1	0	0
0		1		0	1	0
0			1	0	1	0
0			1	0	1	0
0		1		0	1	0
0			1	0	1	0
0	nd	nd	nd	0	0	1
0		1		1	0	0

0		1		1	0	0
0		1		1	0	0
0		1		1	0	0
0	1			1	0	0
0		1		1	0	0
0			1	0	1	0
0		1		1	0	0
0		1		1	0	0
0		1		1	0	0
0		1		1	0	0
0		1		1	0	0
0		1		1	0	0
0		1		1	0	0
0		1		1	0	0
0		1		1	0	0
0		1		1	0	0
0		1		1	0	0
0	1			1	0	0
0	1			1	0	0
0		1		1	0	0
0	1			1	0	0
0			1	0	1	0
0		1		1	0	0
0	1			1	0	0
0	1			1	0	0
0	1			1	0	0
0		1		1	0	0
0		1		1	0	0
0		1		1	0	0
1	nd	nd	nd	0	0	0
1	nd	nd	nd	0	0	0
1	nd	nd	nd	0	0	0
1	nd	nd	nd	0	0	0
1	nd	nd	nd	0	0	0
1	nd	nd	nd	0	0	0
1	nd	nd	nd	0	0	0
1	nd	nd	nd	0	0	0
1	nd	nd	nd	0	0	0
1	nd	nd	nd	0	0	0
1	nd	nd	nd	0	0	0
1		1		0	0	0
1	1			0	0	0
1	1			0	0	0
1		1		0	0	0
1		1		0	0	0
1		1		0	0	0
1	1			0	0	0
1	nd	nd	nd	0	0	0
1	nd	nd	nd	0	0	0
1	nd	nd	nd	0	0	0

1	nd	nd	nd	0	0	0
1	nd	nd	nd	0	0	0
1	nd	nd	nd	0	0	0
1	nd	nd	nd	0	0	0
1	nd	nd	nd	0	0	0
1	nd	nd	nd	0	0	0
1	nd	nd	nd	0	0	0
1	nd	nd	nd	0	0	0
1	nd	nd	nd	0	0	0
1	nd	nd	nd	0	0	0
1	nd	nd	nd	0	0	0
0		1		1	0	0
0	1			1	0	0
1		1		0	0	0
1	1			0	0	0
1		1		0	0	0
1		1		0	0	0
1		1		0	0	0
1	1			0	0	0
1	nd	nd	nd	0	0	0
1	1			0	0	0
1		1		0	0	0
1	nd	nd	nd	0	0	0
1	1			0	0	0
1	1			0	0	0
1		1		0	0	0
0		1		1	0	0
0	1			1	0	0
0		1		1	0	0
0			1	0	1	0
0			1	0	1	0
0			1	0	1	0
0		1		0	0	0
0	1			1	0	0
0			1	0	1	0
0			1	0	1	0
0		1		1	0	0
0		1		1	0	0
0			1	0	1	0
0			1	0	1	0
1		1		0	0	0
1	1			0	0	0
1		1		0	0	0
1	1			0	0	0
1		1		0	0	0
1		1		0	0	0
1	1			0	0	0
1		1		0	0	0
0			1	0	1	0
0			1	0	1	0

0	1			1	0	0
1		1		0	0	0
0	nd	nd	nd	0	0	1
0			1	0	1	0
0		1		1	0	0
0	1			1	0	0
0		1		1	0	0
0		1		1	0	0
0	1			1	0	0
0			1	0	1	0
0			1	0	1	0
0			1	0	1	0
0		1		1	0	0
0		1		1	0	0
0		1		1	0	0
0			1	0	1	0
0	1			1	0	0
0		1		1	0	0
0			1	0	1	0
0	1			1	0	0
0		1		1	0	0
0		1		1	0	0
0		1		1	0	0
0	nd	nd	nd	0	1	0
0	1			1	0	0
1	1			0	0	0
1		1		0	0	0
1		1		0	0	0
1	1			0	0	0
1	1			0	0	0
1		1		0	0	0
1		1		0	0	0
1		1		0	0	0
0		1		1	0	0
0			1	0	1	0
0		1		1	0	0
0		1		1	0	0
0		1		1	0	0
0		1		1	0	0
0		1		1	0	0
0		1		1	0	0
0	1			1	0	0
0			1	0	1	0
0		1		1	0	0
0		1		1	0	0
0		1		1	0	0
0		1		1	0	0
1		1		0	0	0
1	1			0	0	0
1	1			0	0	0

1		1		0	0	0
1		1		0	0	0
1	1			0	0	0
1		1		0	0	0
1		1		0	0	0
1		1		0	0	0
1	1			0	0	0
0	1			1	0	0
1	nd	nd	nd	0	0	0
1		1		0	0	0
1		1		0	0	0
0	1			1	0	0
1	1			0	0	0
1	1			0	0	0
0			1	0	1	0
1		1		0	0	0
0		1		1	0	0
0		1		1	0	0
0		1		1	0	0
0	nd	nd	nd	0	0	1
0		1		1	0	0
0		1		1	0	0
0	nd	nd	nd	0	0	1
1	1			0	0	0
1	1			0	0	0
1		1		0	0	0
1	1			0	0	0
1	1			0	0	0
1		1		0	0	0
1		1		0	0	0
1		1		0	0	0
1		1		0	0	0
1	1			0	0	0
0			1	0	1	0
0		1	2	0	1	0
0			1	0	1	0
0		1		1	0	0
0		1		1	0	0
0		1		1	0	0
0		1	2	0	1	0
0		1		0	1	0
0			1	0	1	0
1	1	2		0	0	0
1		1		0	0	0
0			1	0	1	0
1	1			0	0	0
1	1			0	0	0
1	nd	nd	nd	0	0	0
1	1	2		0	0	0
0	1			1	0	0

0	1	2		1	0	0
0			1	0	1	0
0		1		0	1	0
0		1		1	0	0
0	nd	nd	nd	0	0	1
0		1		0	0	0
0		1	2	0	1	0
1	1			0	0	0
0	1			1	0	0
0	nd	nd	nd	0	1	0
0	1	2		1	0	0
0		1		1	0	0
0		1		0	0	0
0		1		1	0	0
1	1			0	0	0
1	1			0	0	0
1	1			0	0	0
1	1			0	0	0
1	1			0	0	0
1		1		0	0	0
1	1			0	0	0
1	1			0	0	0
1		1		0	0	0
1		1		0	0	0
1		1		0	0	0
0		1		1	0	0
0		1		1	0	0
0		1		0	1	0
0		1		1	0	0
0		1		1	0	0
0	1			1	0	0
0	1			1	0	0
0			1	0	1	0
0			1	0	1	0
0		1		1	0	0
0		1		1	0	0
0		1		0	1	0
0		1		0	1	0
0		1		0	1	0
0		1		1	0	0
0			1	0	1	0
0		1		0	1	0
0			1	0	1	0
0	1	2		1	0	0
0		1		1	0	0
0		1		1	0	0
0		1		1	0	0
1	1	2		0	0	0
1	1			0	0	0
1	1			0	0	0

1	1			0	0	0
1		1		0	0	0
1		1		0	0	0
0	nd	nd	nd	0	0	1
0		1		1	0	0
0		1		1	0	0
0		1		1	0	0
0		1	2	0	1	0
0		1	2	0	1	0
0		1		1	0	0
0		1	2	1	0	0
0		1	2	0	1	0
0		1		1	0	0
0		1		1	0	0
0		1		1	0	0
0	nd	nd	nd	0	0	1
0		1		1	0	0
0		1		1	0	0
0		1		1	0	0
0		1	2	1	0	0
0		1		1	0	0
0		1		0	1	0
0		1		1	0	0
1	1			0	0	0
1		1		0	0	0
1		1		0	0	0
0		1		1	0	0
0			1	0	1	0
0		1		1	0	0
0	nd	nd	nd	0	0	1
1	1	2		0	0	0
0			1	0	1	0
0		1		1	0	0
0		1	2	1	0	0
1	1			0	0	0
0		1	2	0	1	0
0		1	2	0	1	0
0		1		0	0	0
0			1	0	1	0
0		1		1	0	0
0	1	2		1	0	0
0		1		1	0	0
0	nd	nd	nd	0	0	1
0	nd	nd	nd	0	0	1
1	1			0	0	0
1	1			0	0	0
1	1			0	0	0
1		1		0	0	0
1		1		0	0	0
1		1		0	0	0

1		1		0	0	0
1		1		0	0	0
1	1			0	0	0
1		1		0	0	0
0		1		1	0	0
0		1		1	0	0
0		1		1	0	0
0		1		1	0	0
0		1		1	0	0
0			1	0	1	0
0	nd	nd	nd	0	0	1
0		1		1	0	0
0	nd	nd	nd	0	1	0
1		1		0	0	0
1		1		0	0	0
1		1		0	0	0
1		1		0	0	0
0			1	0	1	0
0			1	0	1	0
0			1	0	1	0
0		1		1	0	0
0		1		1	0	0
0			1	0	1	0
0			1	0	1	0
0		1		0	1	0
0		1		1	0	0
0		1		1	0	0
0			1	0	1	0
0			1	0	1	0
0			1	0	1	0
0			1	0	1	0
0		1	2	1	0	0
0			1	0	1	0
0			1	0	1	0
0	nd	nd	nd	0	1	0
1	nd	nd	nd	0	0	0
0			1	0	1	0
0			1	0	1	0
0	nd	nd	nd	0	1	0
0			1	0	1	0
0			1	0	1	0
0			1	0	1	0
0			1	0	1	0
0		1		1	0	0
0			1	0	1	0
0	nd	nd	nd	0	0	1
0	nd	nd	nd	0	0	1
0	nd	nd	nd	0	0	1
0			1	0	1	0
0			1	0	1	0

0			1	0	1	0
0			1	0	1	0
0	nd	nd	nd	0	0	1
0			1	0	1	0
0			1	0	1	0
0			1	0	1	0
1		1		0	0	0
1	1			0	0	0
0			1	0	1	0
0		1	2	0	1	0
0			1	0	1	0
0		1		1	0	0
0		1		1	0	0
0		1		1	0	0
0		1	2	1	0	0
0			1	0	1	0
0		1	2	0	1	0
0		1	2	0	1	0
0			1	0	1	0
0			1	0	1	0
0		1	2	0	1	0
0			1	0	1	0
0	1	2		1	0	0
0		1	2	1	0	0
0		1		1	0	0
0		1		1	0	0
0			1	0	1	0
0		1	2	1	0	0
0			1	0	1	0
0		1	2	1	0	0
0			1	0	1	0
0			1	0	1	0
0			1	0	1	0
0		1	2	0	1	0
0			1	0	1	0
0			1	0	1	0
0			1	0	1	0
0		1	2	0	1	0
0			1	0	1	0
0		1	2	0	1	0
0		1	2	0	1	0
0	nd	nd	nd	0	1	0
0		1	2	0	1	0
0			1	0	1	0
0			1	0	1	0
1	1			0	0	0
0			1	0	1	0
1		1		0	0	0
1		1		0	0	0
1		1		0	0	0

1		1		0	0	0
1		1		0	0	0
1		1		0	0	0
0			1	0	1	0
0		1		0	1	0
0		1	2	0	1	0
0		1		0	1	0
0	nd	nd	nd	0	0	1
0			1	0	1	0
0	nd	nd	nd	0	0	1
0		1	2	0	1	0
0		1	2	0	1	0
0	nd	nd	nd	0	0	1
0			1	0	1	0
0			1	0	1	0
0			1	0	1	0
0			1	0	1	0
0		1	2	0	1	0
0			1	0	1	0
0			1	0	1	0
0			1	0	1	0
0			1	0	1	0
0			1	0	1	0
0			1	0	1	0
0			1	0	1	0
0		1	2	0	1	0
0	nd	nd	nd	0	0	1
0		1	2	0	1	0
0		1	2	0	1	0
0	nd	nd	nd	0	0	1
0	nd	nd	nd	0	0	1
0		1		0	1	0
0		1	2	0	1	0
0		1	2	0	1	0
0		1		0	1	0
0		1		0	1	0
0	1	2		0	1	0
0		1		0	1	0
0		1		0	1	0
0		1		0	1	0
0			1	0	1	0
0		1		0	1	0
0		1		0	1	0
0		1		0	1	0
0	nd	nd	nd	0	0	1
0	nd	nd	nd	0	0	1
0	nd	nd	nd	0	0	1
0		1		0	1	0
0			1	0	1	0
0	nd	nd	nd	0	0	1

1	nd	nd	nd	0	0	0
1	nd	nd	nd	0	0	0
0	1	2		0	1	0
0	1	2		0	1	0
0			1	0	1	0
0			1	0	0	1
0		1		0	1	0
0		1	2	0	1	0
0			1	0	1	0
0			1	0	1	0
0	nd	nd	nd	0	0	1
0		1		0	1	0
0		1		0	1	0
0		1		0	1	0
0		1	2	0	1	0
0			1	0	1	0
0		1	2	0	1	0
1		1		0	0	0
1	nd	nd	nd	0	0	0
1	nd	nd	nd	0	0	0
1	nd	nd	nd	0	0	0
1	nd	nd	nd	0	0	0
1	nd	nd	nd	0	0	0
1	nd	nd	nd	0	0	0
1		1		0	0	0
1		1		0	0	0
1		1		0	0	0
1		1		0	0	0
1		1		0	0	0
1	1			0	0	0
1	1			0	0	0
1	nd	nd	nd	0	0	0
1		1		0	0	0
1		1		0	0	0
1	nd	nd	nd	0	0	0
1	nd	nd	nd	0	0	0
1	nd	nd	nd	0	0	0
1		1		0	0	0
1	1			0	0	0
1		1		0	0	0
1		1		0	0	0
1	1			0	0	0
1	1			0	0	0
1	1			0	0	0
1		1		0	0	0
1		1		0	0	0
1	nd	nd	nd	0	0	0
1	nd	nd	nd	0	0	0
1	nd	nd	nd	0	0	0
1	nd	nd	nd	0	0	0

1	nd	nd	nd	0	0	0
0		1		1	0	0
0		1		1	0	0
1	nd	nd	nd	0	0	0
0		1		1	0	0
0			1	0	1	0
0	1			1	0	0
0	1			1	0	0
1	nd	nd	nd	0	0	0
1	nd	nd	nd	0	0	0
1	nd	nd	nd	0	0	0
1	nd	nd	nd	0	0	0
1	nd	nd	nd	0	0	0
1	nd	nd	nd	0	0	0
0		1		1	0	0
1	nd	nd	nd	0	0	0
1		1		0	0	0
1	nd	nd	nd	0	0	0
1	nd	nd	nd	0	0	0
1	nd	nd	nd	0	0	0
1	nd	nd	nd	0	0	0
0	1			1	0	0
0	1	2		1	0	0
0		1		1	0	0
1	nd	nd	nd	0	0	0
1	nd	nd	nd	0	0	0
1	nd	nd	nd	0	0	0
0		1	2	1	0	0
0	nd	nd	nd	0	0	1
1	nd	nd	nd	0	0	0
0		1		1	0	0
0		1		1	0	0
1	nd	nd	nd	0	0	0
1	nd	nd	nd	0	0	0
1	nd	nd	nd	0	0	0
0		1		1	0	0
1	nd	nd	nd	0	0	0
1	nd	nd	nd	0	0	0
1	nd	nd	nd	0	0	0
0		1		1	0	0
0		1		1	0	0
0		1		1	0	0
1	nd	nd	nd	0	0	0
0		1	2	0	1	0
1	nd	nd	nd	0	0	0
1	nd	nd	nd	0	0	0
0		1		1	0	0
0	1			1	0	0
0		1		1	0	0
0	1	2		1	0	0

0		1		1	0	0
0	1	2		1	0	0
1	nd	nd	nd	0	0	0
1	nd	nd	nd	0	0	0
0		1		1	0	0
0		1		1	0	0
0		1		1	0	0
0	1	2		1	0	0
0			1	0	1	0
0		1		1	0	0
0		1		1	0	0
1	nd	nd	nd	0	0	0
1	nd	nd	nd	0	0	0
0		1		1	0	0
0		1		1	0	0
0	1	2		1	0	0
0	1			1	0	0
0		1		1	0	0
0	1	2		1	0	0
0		1		1	0	0
0		1		1	0	0
0		1		1	0	0
1	nd	nd	nd	0	0	0
1	nd	nd	nd	0	0	0
1	nd	nd	nd	0	0	0
0		1		1	0	0
0		1		1	0	0
0	1			1	0	0
0	1			1	0	0
0		1		0	0	0
0	1			1	0	0
0		1		1	0	0
1	nd	nd	nd	0	0	0
1	nd	nd	nd	0	0	0
1	nd	nd	nd	0	0	0
1	nd	nd	nd	0	0	0
1	nd	nd	nd	0	0	0
1	nd	nd	nd	0	0	0
1	nd	nd	nd	0	0	0
1	nd	nd	nd	0	0	0
1	nd	nd	nd	0	0	0
0		1		1	0	0
0		1		0	0	0
0		1		0	0	0
0		1		0	0	0
0		1		0	0	0
0		1		0	0	0
0		1		0	0	0
0		1		0	0	0
0		1		0	0	0

0		1		0	0	0
0		1		1	0	0
0		1		1	0	0
0		1		1	0	0
0	nd	nd	nd	0	0	1
0		1	2	0	1	0
0			1	0	1	0
0		1		1	0	0
0		1		1	0	0
0	1	2		1	0	0
0	nd	nd	nd	0	0	1
0			1	0	1	0
0		1		1	0	0
0		1		1	0	0
0		1		1	0	0
0		1		1	0	0
0	nd	nd	nd	0	0	1
0			1	0	1	0
0		1		1	0	0
0		1		1	0	0
0			1	0	1	0
0		1		1	0	0
0			1	0	1	0
0			1	0	1	0
0		1		1	0	0
0		1		1	0	0
0			1	0	1	0
0	nd	nd	nd	0	0	1
0			1	0	1	0
0		1		1	0	0
0	nd	nd	nd	0	0	1
1	nd	nd	nd	0	0	0
1	nd	nd	nd	0	0	0
1	nd	nd	nd	0	0	0
1	nd	nd	nd	0	0	0
1	nd	nd	nd	0	0	0
1	nd	nd	nd	0	0	0
0			1	0	0	1
1	nd	nd	nd	0	0	0
1	nd	nd	nd	0	0	0
1	nd	nd	nd	0	0	0
1	nd	nd	nd	0	0	0
1	nd	nd	nd	0	0	0
1	nd	nd	nd	0	0	0
1	nd	nd	nd	0	0	0
1	nd	nd	nd	0	0	0
1	nd	nd	nd	0	0	0
1	nd	nd	nd	0	0	0
1	nd	nd	nd	0	0	0
0		1	2	0	1	0

0			1	0	1	0
0		1		1	0	0
0		1		1	0	0
0			1	0	1	0
0			1	0	0	1
0			1	0	1	0
0		1		1	0	0
0			1	0	1	0
0			1	0	1	0
0	1	2		1	0	0
1	nd	nd	nd	0	0	0
1	nd	nd	nd	0	0	0
1	nd	nd	nd	0	0	0
0	1	2		1	0	0
0		1		1	0	0
0			1	0	1	0
0		1		1	0	0
0			1	0	1	0
0			1	0	1	0
0		1	2	0	1	0
0			1	0	1	0
1	nd	nd	nd	0	0	0
1	nd	nd	nd	0	0	0
0	1	2		1	0	0
0		1		1	0	0
0	1	2		1	0	0
0	1	2		1	0	0
0	1	2		1	0	0
0	1	2		1	0	0
1	nd	nd	nd	0	0	0
1	nd	nd	nd	0	0	0
1	nd	nd	nd	0	0	0
1	nd	nd	nd	0	0	0
1	nd	nd	nd	0	0	0
1	nd	nd	nd	0	0	0
1	nd	nd	nd	0	0	0
1	nd	nd	nd	0	0	0
0		1	2	0	1	0
0		1	2	0	1	0
0		1	2	0	1	0
0		1	2	0	1	0
0			1	0	1	0
0	1	2		1	0	0
0			1	0	1	0
0		1		1	0	0
0		1		1	0	0
1	nd	nd	nd	0	0	0
1	nd	nd	nd	0	0	0
1	nd	nd	nd	0	0	0
1	nd	nd	nd	0	0	0

1	nd	nd	nd	0	0	0
1	nd	nd	nd	0	0	0
0			1	0	1	0
0			1	0	1	0
0		1		1	0	0
0		1		1	0	0
0		1		1	0	0
0		1		1	0	0
0	1	2		1	0	0
0	nd	nd	nd	0	0	1
0		1	2	0	1	0
0		1		1	0	0
1	nd	nd	nd	0	0	0
0		1		1	0	0
0		1		1	0	0
0	nd	nd	nd	0	0	1
0		1	2	1	0	0
0		1	2	1	0	0
0		1		1	0	0
0			1	0	1	0
0		1	2	1	0	0
0	1			1	0	0
0		1		1	0	0
0		1	2	1	0	0
0		1		1	0	0
0		1		1	0	0
0		1		1	0	0
0		1	2	1	0	0
1	nd	nd	nd	0	0	0
1	nd	nd	nd	0	0	0
1	nd	nd	nd	0	0	0
1	nd	nd	nd	0	0	0
0		1		1	0	0
0			1	0	1	0
0		1	2	0	1	0
0	nd	nd	nd	0	0	1
0		1		1	0	0
0	1			1	0	0
1	nd	nd	nd	0	0	0
1	nd	nd	nd	0	0	0
1	nd	nd	nd	0	0	0
0	1			1	0	0
0	1			1	0	0
0			1	0	1	0
0		1	2	0	1	0
0			1	0	1	0
0	1	2		1	0	0
0		1		1	0	0
0		1		1	0	0
0		1		1	0	0

0		1		1	0	0
0		1		1	0	0
0		1		1	0	0
1	nd	nd	nd	0	0	0
0		1		1	0	0
0		1		1	0	0
0	nd	nd	nd	0	0	1
0	nd	nd	nd	0	0	1
0		1		1	0	0
0		1		1	0	0
0		1		1	0	0
0		1		1	0	0
0		1		1	0	0
0		1		1	0	0
0		1		1	0	0
0	nd	nd	nd	0	0	1
0	1	2		1	0	0
0		1		1	0	0
0		1		1	0	0
0			1	0	1	0
0			1	0	1	0
1	nd	nd	nd	0	0	0
1	nd	nd	nd	0	0	0
0	1			1	0	0
0	1			1	0	0
0	1			1	0	0
0		1		1	0	0
0		1		1	0	0
0		1		1	0	0
0		1		1	0	0
0		1		1	0	0
0			1	0	1	0
1	nd	nd	nd	0	0	0
1	nd	nd	nd	0	0	0
0		1	2	1	0	0
0		1	2	1	0	0
0		1	2	0	1	0
0	1	2		1	0	0
0		1	2	1	0	0
0		1		1	0	0
0	1	2		1	0	0
0		1		1	0	0
0		1	2	1	0	0
0		1	2	0	1	0
0		1		1	0	0
0	nd	nd	nd	0	0	1
0		1	2	1	0	0
0			1	0	1	0
0			1	0	1	0
0		1	2	0	1	0

0	nd	nd	nd	0	0	1
0		1	2	0	1	0
0		1	2	0	1	0
0		1		0	1	0
0		1	2	0	1	0
0	1	2		0	1	0
0		1		0	1	0
0			1	0	1	0
0		1	2	0	1	0
0		1		0	1	0
0		1	2	0	1	0
0		1		0	1	0
0	1			0	1	0
0		1	2	0	1	0
0		1	2	0	1	0
0			1	0	1	0
0		1		0	1	0
0		1	2	0	1	0
0	1	2		0	1	0
0		1	2	0	1	0
0		1	2	0	1	0
0			1	0	1	0
0		1	2	0	1	0
0		1	2	0	1	0
0		1	2	0	1	0
0		1		0	1	0
0		1		0	1	0
0		1	2	0	1	0
0		1		0	1	0
0			1	0	1	0
0		1		0	1	0
0			1	0	1	0
0			1	0	1	0
0	nd	nd	nd	0	0	1
0			1	0	1	0
0			1	0	1	0
0		1	1	0	1	0
0			1	0	1	0
0			1	0	1	0
0			1	0	1	0
0			1	1	0	0
0			1	1	0	0
0	nd	nd	nd	0	0	1
0			1	1	0	0
0			1	1	0	0
0			1	1	0	0
0			1	1	0	0
0	nd	nd	nd	0	0	1
0	nd	nd	nd	0	0	1
0	nd	nd	nd	0	0	1
