# Supplementary material for: Innovative monitoring scheme adapted to remote, scattered nesting aggregation reveals a major loggerhead turtle rookery in New Caledonia, South Pacific
Source: PLoS One. 2024 Jun 18;19(6):e0299748. doi: 10.1371/journal.pone.0299748 (PMC11185463; doi:10.1371/journal.pone.0299748)
Supplement: S1 Table — (PDF) [file pone.0299748.s004.pdf]

S1 Table. Seasonal number of nests and 95% CI for each considered islet. 'Nests' refers to the median projection of the seasonal total number of nests for each considered islet.

	2017-2018		2018-2019		2019-2020		2020-2021		2021-2022		2022-2023	
**islet**	**Nests**	**95% CI**	**Nests**	**95% CI**	**Nests**	**95% CI**	**Nests**	**95% CI**	**Nests**	**95% CI**	**Nests**	**95% CI**
amere	35	19-52	41	34-50	31	18-45	24	13-35	33	19-47	17	13-23
atire	46	43-49	46	40-51	28	25-32	45	43-47	32	32-32	25	23-26
du ami	9	4-16	5	1-15	8	4-14	6	3-11	9	4-14	7	3-11
gi	35	32-39	43	38-47	23	20-27	18	16-20	47	47-47	15	14-15
ieroue	9	8-11	4	2-6	5	3-7	6	5-6	5	5-5	14	13-14
kie	65	43-91	77	69-87	58	40-78	44	31-60	61	43-82	36	30-43
koko	30	14-47	30	23-38	26	13-40	20	10-31	28	13-42	21	10-31
kouare	11	9-13	26	21-31	6	5-7	27	25-28	8	8-8	13	12-13
mato	11	6-17	6	1-16	10	4-17	3	1-6	8	5-13	1	0-4
mbore	12	6-19	13	7-19	11	5-16	6	3-9	3	1-8	8	5-13
nda	28	25-31	7	5-10	18	5-44	11	11-12	11	11-11	28	27-29
ndo	14	7-21	6	1-15	45	37-54	7	4-11	8	5-13	33	27-40
nge	16	13-19	47	42-53	23	20-25	19	18-20	50	50-50	18	17-18
noe	9	5-14	2	0-10	5	3-8	1	0-4	5	2-11	1	0-4
nouare	9	5-14	10	6-14	2	0-9	2	0-10	3	1-8	1	0-4
petit koko	11	6-17	26	19-35	10	5-14	1	0-4	3	1-8	5	2-10
puemba	16	9-25	18	10-26	11	8-14	7	4-11	8	4-14	18	13-24
pumbo	9	5-15	10	5-15	2	0-10	3	1-6	3	1-8	6	3-10
redika	37	35-40	16	13-19	20	18-22	1	1-2	12	12-12	5	4-5
tere	3	2-5	6	4-9	4	0-22	0	0-0	0	0-0	2	2-2
totea	19	11-29	13	7-22	17	10-25	7	4-11	15	11-21	27	21-33
ua	6	4-8	5	1-12	24	18-31	1	1-1	3	3-3	7	6-7
uaterembi	30	26-33	15	12-18	8	6-10	13	13-14	22	22-22	14	14-15
uatio	17	10-25	20	17-24	17	15-19	4	3-4	8	8-8	17	17-19
ugo	8	4-12	8	5-12	2	0-9	1	0-4	1	0-5	1	0-4
uie	13	7-20	6	1-15	15	12-18	7	4-11	19	14-25	1	0-4
uo	10	6-16	10	5-17	4	2-6	1	0-4	10	5-16	1	0-4
vua	14	13-16	16	13-20	24	16-34	20	20-21	21	21-21	22	22-23
