# Supplementary material for: Innovative monitoring scheme adapted to remote, scattered nesting aggregation reveals a major loggerhead turtle rookery in New Caledonia, South Pacific
Source: PLoS One. 2024 Jun 18;19(6):e0299748. doi: 10.1371/journal.pone.0299748 (PMC11185463; doi:10.1371/journal.pone.0299748)
Supplement: Attachment — Submitted filename: PONE-D-24-06149.pdf [file pone.0299748.s005.pdf]

PLOS ONE

Innovative monitoring scheme at remote, scattered nesting aggregation reveals a major loggerhead turtle rookery in New Caledonia, South Pacific

--Manuscript Draft--

Manuscript Number:	PONE-D-24-06149
Article Type:	Research Article
Full Title:	Innovative monitoring scheme at remote, scattered nesting aggregation reveals a major loggerhead turtle rookery in New Caledonia, South Pacific
Short Title:	Innovative monitoring scheme reveals a major, scattered loggerhead turtle rookery in New Caledonia
Corresponding Author:	Hugo Bourgogne, Ph.D.<br>IRD: Institut de recherche pour le developpement<br>Noumea, NEW CALEDONIA
Keywords:	Marine turtle; loggerhead turtle; endangered species; nesting rookery; coral-reef island; nests counts; monitoring protocol; phenology modelling; science-based conservation; New Caledonia; South Pacific
Abstract:	The loggerhead turtle Caretta caretta is a large marine turtle with a cosmopolitan repartition in warm and temperate waters of the planet. The South Pacific subpopulation is classified as 'Critically Endangered' on the IUCN Red List, based on the estimated demographic decline. This precarious situation engages an urgent need to monitor nesting populations in order to highlight conservation priorities and to ensure their efficiency over time. New Caledonia encompasses a large number of micro and distant nesting sites, localized on coral islets widely distributed across its large lagoon. Adequately surveying nesting activities on those hard-to-reach beaches can prove to be challenging. As a result, important knowledge gaps prevail in those high-potential nesting habitats. For the first time, an innovative monitoring scheme was conducted to assess the intensity of nesting activities, considered as a proxy of the population size, on an exhaustive set of islets located in the 'Grand Lagon Sud' area. These data were analyzed using a set of statistical methods specially designed to produce phenology and nesting activity estimates using Bayesian methods. This analysis revealed that this rookery hosts a large nesting colony, with a mean annual estimate of 437 nests (95% Credible Interval=328-582). These numbers exceed that of the previous estimated annual number of loggerhead turtle nests in New Caledonia, highlighting the exceptional nature of this area. Considering the fact that similar high-potential aggregations have been identified in other parts of New Caledonia, but failed to be comprehensively assessed to this day, we recommend carrying out this replicable monitoring scheme to other locations. It could allow a significant re-evaluation of the New Caledonian nesting population importance and, ultimately, of its prevailing responsibility for the protection of this patrimonial yet endangered species.
Order of Authors:	Hugo Bourgogne, Ph.D.
	Marc Oremus, Doctor
	Morgan Mangeas, Doctor
	Eric Vidal, Doctor
	Marc Girondot, Doctor
Additional Information:	
Question	Response
**Financial Disclosure**<br><br>Enter a financial disclosure statement that describes the sources of funding for the work included in this submission. Review	Yes

Powered by Editorial Manager® and ProduXion Manager® from Aries Systems Corporation

the submission guidelines for detailed requirements. View published research articles from *PLOS ONE* for specific examples.<br><br>This statement is required for submission and **will appear in the published article** if the submission is accepted. Please make sure it is accurate.<br><br>**Funded studies**<br>Enter a statement with the following details:<br>• Initials of the authors who received each award<br>• Grant numbers awarded to each author<br>• The full name of each funder<br>• URL of each funder website<br>• Did the sponsors or funders play any role in the study design, data collection and analysis, decision to publish, or preparation of the manuscript?<br><br>Did you receive funding for this work?	
Please add funding details.<br>as follow-up to "**Financial Disclosure**<br><br>Enter a financial disclosure statement that describes the sources of funding for the work included in this submission. Review the submission guidelines for detailed requirements. View published research articles from *PLOS ONE* for specific examples.<br><br>This statement is required for submission and **will appear in the published article** if the submission is accepted. Please make sure it is accurate.	This study was supported and funded by the French Research Institute for Sustainable Development IRD (https://www.ird.fr/) for salary considerations for HB (through a ANRT CIFRE grand)/EV/MM, and the World Wide Fund for Nature international non-governmental organization WWF-France (https://www.wwf.fr/) for salary considerations for HB/MO and operating expenses. It takes place in the framework of a PhD program jointly supervised by the susnamed funders, which naturally had a role in the study design, data collection and analysis, decision to publish and preparation of the manuscript as the executive project owners.

Powered by Editorial Manager® and ProduXion Manager® from Aries Systems Corporation

**Funded studies**<br>Enter a statement with the following details:<br>• Initials of the authors who received each award<br>• Grant numbers awarded to each author<br>• The full name of each funder<br>• URL of each funder website<br>• Did the sponsors or funders play any role in the study design, data collection and analysis, decision to publish, or preparation of the manuscript?<br><br>Did you receive funding for this work?"	
Please select the country of your main research funder (please select carefully as in some cases this is used in fee calculation).<br>as follow-up to "**Financial Disclosure**<br><br>Enter a financial disclosure statement that describes the sources of funding for the work included in this submission. Review the submission guidelines for detailed requirements. View published research articles from *PLOS ONE* for specific examples.<br><br>This statement is required for submission and **will appear in the published article** if the submission is accepted. Please make sure it is accurate.<br><br>**Funded studies**<br>Enter a statement with the following details:<br>• Initials of the authors who received each award<br>• Grant numbers awarded to each author<br>• The full name of each funder<br>• URL of each funder website<br>• Did the sponsors or funders play any role in the study design, data collection and analysis, decision to publish, or preparation of the manuscript?<br><br>Did you receive funding for this work?"	FRANCE - FR
**Competing Interests**	The authors have declared that no competing interests exist.

Use the instructions below to enter a competing interest statement for this submission. On behalf of all authors, disclose any competing interests that could be perceived to bias this work—acknowledging all financial support and any other relevant financial or non-financial competing interests.<br><br>This statement is required for submission and **will appear in the published article** if the submission is accepted. Please make sure it is accurate and that any funding sources listed in your Funding Information later in the submission form are also declared in your Financial Disclosure statement.<br><br>View published research articles from *PLOS ONE* for specific examples.<br><br>**NO authors have competing interests**<br><br>Enter: *The authors have declared that no competing interests exist.*<br><br>**Authors with competing interests**<br><br>Enter competing interest details beginning with this statement:<br><br>*I have read the journal's policy and the authors of this manuscript have the following competing interests: [insert competing interests here]*<br><br>* typeset	
**Ethics Statement**<br><br>Enter an ethics statement for this submission. This statement is required if the study involved:<br><br>• Human participants<br>• Human specimens or tissue<br>• Vertebrate animals or cephalopods<br>• Vertebrate embryos or tissues	This work has been supported in its field research phases by the South Province of New Caledonia (permit #3245-2020/ARR/DDDT).

• Field research<br><br>Write "N/A" if the submission does not require an ethics statement.<br><br>General guidance is provided below. Consult the submission guidelines for detailed instructions. **Make sure that all information entered here is included in the Methods section of the manuscript.**	

**Format for specific study types**<br><br>**Human Subject Research (involving human participants and/or tissue)**<br>• Give the name of the institutional review board or ethics committee that approved the study<br>• Include the approval number and/or a statement indicating approval of this research<br>• Indicate the form of consent obtained (written/oral) or the reason that consent was not obtained (e.g. the data were analyzed anonymously)<br><br>**Animal Research (involving vertebrate animals, embryos or tissues)**<br>• Provide the name of the Institutional Animal Care and Use Committee (IACUC) or other relevant ethics board that reviewed the study protocol, and indicate whether they approved this research or granted a formal waiver of ethical approval<br>• Include an approval number if one was obtained<br>• If the study involved *non-human primates*, add *additional details* about animal welfare and steps taken to ameliorate suffering<br>• If anesthesia, euthanasia, or any kind of animal sacrifice is part of the study, include briefly which substances and/or methods were applied<br><br>**Field Research**<br><br>Include the following details if this study involves the collection of plant, animal, or other materials from a natural setting:<br>• Field permit number<br>• Name of the institution or relevant body that granted permission	
**Data Availability**<br><br>Authors are required to make all data underlying the findings described fully available, without restriction, and from the time of publication. PLOS allows rare exceptions to address legal and ethical concerns. See the PLOS Data Policy and FAQ for detailed information.	Yes - all data are fully available without restriction

A Data Availability Statement describing where the data can be found is required at submission. Your answers to this question constitute the Data Availability Statement and **will be published in the article**, if accepted.<br><br>**Important:** Stating 'data available on request from the author' is not sufficient. If your data are only available upon request, select 'No' for the first question and explain your exceptional situation in the text box.<br><br>Do the authors confirm that all data underlying the findings described in their manuscript are fully available without restriction?	
**Describe where the data may be found in full sentences. If you are copying our sample text, replace any instances of XXX with the appropriate details.**<br><br>• If the data are **held or will be held in a public repository**, include URLs, accession numbers or DOIs. If this information will only be available after acceptance, indicate this by ticking the box below. For example: *All XXX files are available from the XXX database (accession number(s) XXX, XXX.).*<br>• If the data are all contained **within the manuscript and/or Supporting Information files**, enter the following: *All relevant data are within the manuscript and its Supporting Information files.*<br>• If neither of these applies but you are able to provide **details of access elsewhere**, with or without limitations, please do so. For example:<br><br>*Data cannot be shared publicly because of [XXX]. Data are available from the XXX Institutional Data Access / Ethics Committee (contact via XXX) for researchers who meet the criteria for access to confidential data.*<br><br>*The data underlying the results presented in the study are available from (include the name of the third party*	All relevant data are within the manuscript and its Supporting Information files.

and contact information or URL).<br>• This text is appropriate if the data are owned by a third party and authors do not have permission to share the data.<br><br>* typeset	
Additional data availability information:	

Manuscript Click here to access/download;Manuscript;manuscript.docx

Full title

Innovative monitoring scheme at remote, scattered nesting aggregation reveals a major loggerhead turtle rookery in New Caledonia, South Pacific

Short title

Innovative monitoring scheme reveals a major, scattered loggerhead turtle rookery in New Caledonia

Hugo Bourgogne[1,2*], Marc Oremus[2], Morgan Mangeas[1], Eric Vidal[1], Marc Girondot[3]

[1] UMR 250 ENTROPIE, Centre IRD de Nouméa, IRD, CNRS, Ifremer, Université de la Réunion, Université de la Nouvelle-Calédonie, Nouméa, New Caledonia, France

[2] WWF-France bureau Nouvelle-Calédonie, World Wide Fund for Nature (WWF), Nouméa, New Caledonia, France

[3] Laboratoire Ecologie, Systématique et Evolution, Université Paris-Saclay, AgroParisTech, Centre National de la Recherche Scientifique (CNRS), Gif-sur-Yvette, France

* Corresponding author

E-mail: hugo.bourgogne@ird.fr

Abstract

The loggerhead turtle *Caretta caretta* is a large marine turtle with a cosmopolitan repartition in warm and temperate waters of the planet. The South Pacific subpopulation is classified as 'Critically Endangered' on the IUCN Red List, based on the estimated demographic decline. This precarious situation engages an urgent need to monitor nesting populations in order to highlight conservation priorities and to ensure their efficiency over time. New Caledonia encompasses a large number of micro and distant nesting sites, localized on coral islets widely distributed across its large lagoon. Adequately surveying nesting activities on those hard-to-reach beaches can prove to be challenging. As a result, important knowledge gaps prevail in those high-potential nesting habitats. For the first time, an innovative monitoring scheme was conducted to assess the intensity of nesting activities, considered as a proxy of the population size, on an exhaustive set of islets located in the 'Grand Lagon Sud' area. These data were analyzed using a set of statistical methods specially designed to produce phenology and nesting activity estimates using Bayesian methods. This analysis revealed that this rookery hosts a large nesting colony, with a mean annual estimate of 437 nests (95% Credible Interval=328-582). These numbers exceed that of the previous estimated annual number of loggerhead turtle nests in New Caledonia, highlighting the exceptional nature of this area. Considering the fact that similar high-potential aggregations have been identified in other parts of New Caledonia, but failed to be comprehensively assessed to this day, we recommend carrying out this replicable monitoring scheme to other locations. It could allow a significant re-evaluation of the New Caledonian nesting population importance and, ultimately, of its prevailing responsibility for the protection of this patrimonial yet endangered species.

1 Introduction

Assessing population abundance and demographic trends is key to develop and evaluate conservation actions for threatened species [1–3]. Marine megafaunal species, such as sea

turtles, face major conservation issues worldwide [4–7]. Yet, long-term monitoring programs can prove to be particularly challenging, due to ocean-scale distributions and migratory nature [8,9]. Onshore quantifications of sea turtle nesting activities have developed into the most common indicator to evaluate the demographic trends, as it is often used as an index of population size [9,10]. Several monitoring schemes has been developed to assess long-term population trends according to nesting site conformations and specificities [11]. Monitoring scattered insular nesting areas with high precision is a statistical challenge due to the detrimental scarcity of data because of remoteness constraints. The high financial costs associated with field presence act indeed as a strong hindrance to the collection of core data. This may lead to important knowledge gaps and significant misunderstanding of demographic processes in high-potential habitats [12–14]. The development of efficient and resource-optimized tools is therefore a priority as it could reveal unsuspected major nesting rookeries [13], especially in the South Pacific region that encompasses a multitude of island-rich geographies [15,16].

The loggerhead turtle *Caretta caretta* (Linnaeus, 1758) is classified as 'Vulnerable' on the International Union for Conservation of Nature's (IUCN) Red List on a global scale. However, the South Pacific subpopulation suffered a strong demographic collapse, showing a loss of over 80% of nesting females over the past three generations. It was therefore classified as 'Critically Endangered' [17]. Nesting aggregations of this distinct Regional Management Unit [7] are restricted to the western part of the ocean basin and are reported almost exclusively in eastern Australia and New Caledonia [18,19]. In Australia, approximately 80% of the loggerhead turtle nesting activities are concentrated at five major areas, each supporting hundreds of nesting females every year [17]. They combine for the greatest concentration of all reproductive individuals in the South Pacific region, as the remainder of the nesting population is dispersed at a large number of small aggregations with 10s or <10 nesting females per year. In New Caledonia, only one important coastal nesting site with several 10s of nesting females every year has been identified on the west coast of the mainland, in the

‘Roche Percée’ beach, commune of Bourail [20,21]. Numerous other smaller nesting beaches have been inventoried on dispersed coral islets along the coastline, forming large aggregations off the northwestern coast of the mainland and in the ‘Grand Lagon Sud’ (GLS) provincial park, at the meridional end of the mainland. Despite the pre identified potential for hosting a significant number of nesting activities, information for those insular rookeries remained sparse and only based on expert opinion because they did not benefit censuses to this date. Given the reported lack of information on the loggerhead turtle demographic status at the national scale [17], the assessment of those nesting areas could prove critical to a re-evaluation of New Caledonia’s population and its importance within the South Pacific subpopulation.

In this study, we chose to give a specific focus on the ‘GLS’ area. It presents strong monitoring logistical constraints, with the requisite coverage of multiple, distant and scattered nesting beaches. We have therefore implemented an exploratory protocol, based on the seasonal survey of 28 islets and supported by an appropriate analytical tool to make up for the scarcity of data generated. Based on a 6 seasons time-series data collection, we have achieved the two main objectives that were initially set:

(1) establish a monitoring protocol well-adapted to the study area, allowing to deliver a baseline knowledge meeting the minimum threshold for data quality [11] while optimizing the resources required for its implementation ;

(2) produce the first estimates of the annual nesting intensity at the ‘GLS’ rookery-scale allowing to evaluate the status of this area in relation to the South Pacific loggerhead turtle population.

2 Materials and methods

2.1 Study area

Censuses were conducted in the 'Grand Lagon Sud' (S22°00, E167°00) of New Caledonia, a 314.500 ha marine protected park in the southern end of the mainland's coral lagoon (Fig 1). Its inscription as a UNESCO world heritage site in 2008 testifies to the exceptional nature of the area and its ecological interest. This region is characterized by a rich coral reef diversity, extensive seagrass and algal communities, a fractioned reef barrier, and 35 distinct coral reef islets distributed over the park.

Fig 1. Geographical location of the study area, in the 'Grand Lagon Sud' of New Caledonia. A: New Caledonia, South West Pacific. B: Position of the 28 surveyed sites within the 'Grand Lagon Sud' area, off the southern end of the main island of New Caledonia. Land is shown in light grey and shallow reefs are shown in blue (shapefile source: Millenium Coral Reef Mapping Project [22]).

The surveyed area requires the use of a motorboat to conduct field missions because of the large geographic scope to cover, with 32 km from Noumea city harbor to reach the first monitored islet, and 88 km to reach the most distant islet. All islets present fringing reefs and are difficult to access by low tide and/or poor weather conditions, leading to major planning constraints. Given the distance to the eastern part of the park (128 km from the departing harbor to the most eastern islet), the choice was made not to include the 7 islets surrounding the Isle of Pines (Fig 1) to the study area. The remaining 28 coral islets of the area were annually monitored over the 2017-18 to 2022-23 period, for a total of n=6 nesting seasons covered (Table 1). The islets are stable at the scale of this study.

Table 1. Designation and whereabouts of the 28 surveyed sites in the 'GLS' rookery.

islet #	name	latitude	longitude	set	islet #	name	latitude	longitude	set
1	atire	-22.5219	166.5603	A	15	nge	-22.6926	166.8507	A
2	redika	-22.5161	166.6130	A	16	gi	-22.7212	166.8516	A
3	vua	-22.5781	166.7475	A	17	nda	-22.8456	166.8773	A
4	uo	-22.5169	166.7696	B	18	uie	-22.7208	166.9125	B
5	mato	-22.5503	166.7923	B	19	mbore	-22.8030	166.9161	B
6	uatio	-22.7090	166.7953	A	20	ugo	-22.4417	166.9285	B
7	kouare	-22.7767	166.8009	A	21	koko	-22.8804	166.9329	B
8	ua	-22.7079	166.8112	A	22	petit koko	-22.8584	166.9439	B
9	uaterembi	-22.6795	166.8117	A	23	ndo	-22.6840	166.9653	B
10	noe	-22.5393	166.8218	B	24	totea	-22.7203	166.9735	B
11	ieroue	-22.6049	166.8277	A	25	nouare	-22.4238	167.0128	B
12	puemba	-22.5321	166.8289	B	26	kie	-22.3761	167.0646	B
13	pumbo	-22.5205	166.8355	B	27	amere	-22.4441	167.0961	B
14	tere	-22.7888	166.8475	A	28	du ami	-22.6252	167.2797	B

The geographic coordinates point the center of the islet.

2.2 Monitoring protocol

2.2.1 Nesting data count type

Seasonal survey events have been conducted during austral summer in the 'GLS' area to match the reproductive phenology of the loggerhead turtle in New Caledonia [20]. They consisted in a series of 1 to 2 days missions at sea, in which a set of islets were monitored by a minimum of two persons in order to reduce observer bias. All nesting activities observed on the perimeter of the islet were recorded, along with date, site, GPS localization and nature of the detected activity based on the available cues: crawls up and down the beach, aborted or successful nest as materialized by the characteristic body pit tracks. Knowing that only the loggerhead and the Green turtles *Chelonia mydas* (Linnaeus, 1758) are nesting in New Caledonia [23], species identification was assessed through crawling track observations. 99.9% of all tracks were confirmed as asymmetrical, thus assigned to loggerhead turtles [24].

We chose to use nest counts as the proxy for annual population abundance in this study. The selection of this count unit, rather than the more classical female emergence tracks, has been motivated by our incapacity to visit nesting beaches more than on a weekly basis at best. In this context, the higher persistence in time of body pits compared to crawl tracks makes it a better proxy to avoid detection bias. Moreover, body pits are considered the most accurate proxy to describe patterns in reproductive output after the direct count of individuals [11].

2.2.2 Monitoring strategy in space and time

The 'GLS' rookery presents a spatial configuration where numerous, non-contiguous, distant and hard-to-reach nesting beaches are presumably used by the same nesting population. Setting a well-adapted monitoring scheme is challenging, since no preconized protocol reflects the entire complexity of the present situation [11]. Given the logistic impossibility to monitor each islet with high intensity throughout the nesting season, the initial selection of 28 islets has been divided into two distinct sets, respectively designated as A- and B-set, that will differ in the total seasonal number of survey occurrences (Fig 2 and Table 1).

Fig 2. Monitoring strategy of the two sets of surveyed islets in the 'Grand Lagon Sud' rookery.

The A-set is composed of a panel of 12 islets showing various levels of nesting intensities. It benefits the highest monitoring effort, with a minimum of six survey events throughout the nesting season. Surveys were conducted with a two to three weeks interval in between them in order to (1) take into account the loggerhead inter-nesting interval, which varies between 12 to 25 days based on available sources [20,25], (2) efficiently assess the primary nesting phenology parameters, prerequisite key knowledge to the use of statistical methods to quantify total seasonal number of nests, and (3) provide valuable and whenever possible exhaustive counts from one survey event to another, considering the decrease of the nest detection over time. The 16 remaining islets, composing the B-set, were monitored only twice during the season, with a two to three weeks interval at the peak of the nesting season [26], *i.e.* from mid-

December to mid-January [20]. It would later benefit from the nesting phenology knowledge generated through the A-set survey protocol to produce robust estimations of the seasonal total number of nests on those sites. This monitoring strategy enables a reduced survey coverage while maintaining solid estimates of seasonal total nest counts [11].

2.3 Modeling nesting abundance

2.3.1 Nest tracks persistence over time

Although nesting trends can be detected from nest counts, an understanding of what proportion of the true abundance these counts represent is key to more accurately assess demographic progressions [11]. Without an estimate of detection probability, *i.e.* the proportion of nests recorded on a given survey event, a simple nest count is not as reliable an index of population status or trends [27]. In order to assess this parameter with high precision, we have developed an empiric protocol that allows us to determine the average duration for which a nest track remains detectable for an observer. It is based on the fact that the marks generated by females during nesting activities persist in the sand for a given period of time, which may vary according to weather conditions and to their wind, rain and wave exposure [9]. Nests were marked on the spot with wooden poles on the first detection, then systematically prospected in following survey events. A status of their detection state was set by the observers, from good (coded as 1) to poor (uncertain or no detection, coded as 0).

These data have been analyzed with a generalized linear mixed model using Penalized Quasi-Likelihood with a binomial distribution and a logit link using the function glmmPQL of the 'MASS' R package [28]. Age of the track in days and season (centered to December) were included as fixed factors and ID of the track has been included as a random factor with an autocorrelation structure of order 1 to take into account that the detection state (1 or 0) at time t is dependent on the detection state at time t-Δt. If the track was not detectable at time t, there ~~are more chance~~ that it is still not detectable at time t-Δt, except for false negative or positive

cases. Confidence interval is not available for GLMM-AR1 model and we used 9999 bootstraps to estimate the uncertainty of the predictions.

2.3.2 Model for nesting seasonality

Collected data were processed in a multiple-step pipe. The aim is to convert partial fieldwork data into an estimate of total seasonal nesting intensity through the establishment of the nesting phenology parameters and aggregation over multiple seasons. Nesting seasonality was modeled following the Girondot phenological model [29,30]. This model can be applied to any proxy of nesting such as nest or track counts. Assuming that *t* is an ordinal date (October 1 is 1 and September 30 in the following year is 365 or 366) and that N_t is the observed number of nests for this date, the number of nests deposited per night is modeled using the following set of equation:

$$n_t = \begin{cases} t < B \rightarrow \mathrm{PMin} \times \mathrm{Max} \\ t \in [B, P - F[\rightarrow \left(\left(1 + \cos\left(\pi(P - F - t)(P - F - B)\right)\right)/2\right)(\mathrm{Max} - \mathrm{PMin} \times \mathrm{Max}) + \mathrm{PMin} \times \mathrm{Max} \\ t \in [P - F, P + F[\rightarrow \mathrm{Max} \\ t \in [P + F, E] \rightarrow \left(\left(1 + \cos\left(\pi(t - P + F)(E - P + F)\right)\right)/2\right)(\mathrm{Max} - \mathrm{PMin} \times \mathrm{Max}) + \mathrm{PMin} \times \mathrm{Max} \\ t > E \rightarrow \mathrm{PMin} \times \mathrm{Max} \end{cases} \quad (1)$$

The model requires at most seven parameters, all of which have direct biological interpretations:

B and *E* are the ordinal dates for the start and end of the nesting season.

P is the ordinal date for the peak of the nesting season.

F is half of the number of days around *P* for which the curve flattens out.

Max is the mean number of nests at the peak of the nesting season.

PMin is the mean nightly nest numbers relative to *Max* before and after the nesting season.

The nesting season is described in segments, and all segments form one continuous function. The nesting season is defined as the interval [*B*, *E*]. If *F* is equal to 0, no flat portion is observed. Rather than fitting *B* and *E*, it is more convenient to fit LengthB=*P* – *B* and LengthE=*E* – *P* with LengthB > 0 and LengthE > 0 to ensure that *B* < *P* < *E*. The parameters *B*, *E*, *P*, *F*, LengthB, and LengthE are hereafter defined as shape parameters, and *PMin* and *Max* as scale parameters.

In such a situation when several nesting seasons are analyzed, it is also possible to implement a year effect for Peak (*P*) and/or for LengthB (length of the nesting season from the beginning to the peak) and LengthE (length of the nesting season from the peak to the end). Four categories of models were then fitted depending on the year effects for Peak and/or for LengthB and LengthE. These are defined as the Peak-Global or Peak-Year and Length-Global or Length-Year.

For the seasonality model, parameter fitting was performed using maximum likelihood with negative binomial (NB) daily nest distribution with values produced by Equation (1) ($m=n_t$) as theoretical values and the observed counts ($x=N_t$) as observations. In ecology, NB distribution is used to describe the distribution of an organism while taking into account the mean number of individuals *m* and an aggregation parameter *k* [31]. The probability mass function of NB distribution is:

$$NB(x;m;k) = Pr(X = x) = \frac{\Gamma(k+x)}{x!\,\Gamma(k)}\left(\frac{m}{m+k}\right)^{x}\left(\frac{k}{m+k}\right)^{k}, m > 0, k > 0 \qquad (2)$$

When the mean number of nests is low during all the season, a Poissonian distribution rather than a NB distribution can be used [30]. Poissonian distribution is a special case of NB distribution when *k* is +Inf. When N_t is equal to 0, it ~~was~~ replaced with 10^{-9} as the negative binomial and the Poissonian models are not defined for *m*=0.

When the count (N_t) represents the exact sum of activity during several nights, the probability mass function of the sum of NB is used [32]. When the count represents the minimum number

of activities because of the track loss in time, the likelihood is the integral of the probability mass function of NB for all the values between N_t and the maximal number of nests N_{max}. N_{max} is determined taking into account the probability of detection of a body pit after a known period on the beach (see section 3.1).

2.3.3 Interannual spatial and temporal trends

A model is used to estimate the number of nest tracks for an islet that is not surveyed, based on the relative frequency of tracks on the different sites and the total number of tracks for each nesting season. Let the total theoretical number of nest tracks be T_i for season i in the entire region where K islets were monitored during a range of Y years.

The distribution of the nests across the different sites is defined by the proportion p_j of T_i nests in the j beach. For a total of K islets, K–1 parameters p are necessary due to the relation:

$$\sum_{j=1}^{K} p_j = 1 \quad (3)$$

The values p_j can be modeled as constant (K–1 parameters) or first (2.K–2 parameters) or second (3.K–3 parameters) order as a function of time to represent situations with changes in the relative use of the different nesting sites. The expected number of nests for year i in the beach j is then:

$$E_{i,j} = T_i * p_j \quad (4)$$

Let $N_{i,j}$ be an observed number of nests with a standard deviation of $S_{i,j}$. The distribution of $N_{i,j}$ may be close to a Gaussian distribution when the number of nests and monitoring coverage are high, but it can also be a positive skew when the number of nests or monitoring coverage is low. For this reason, a gamma distribution was used to model the data; the gamma distribution is always positive and can show a positive skew when the standard deviation is high compared to the mean. The fit of the parameters was then completed using maximum likelihood with a gamma distribution. For the final estimate, the expected number of nests $E_{i,j}$

was only used when no observation was available; in other situations, the number of nests fitted using the phenology model was preferred.

The log likelihood of the observations is the sum of the log likelihood for each observation $N_{i,j}$ within the gamma model.

2.3.4 Strategy for parameter fitting

The strategy for the fitting parameters model is similar for the seasonality model and the interannual spatial and temporal trend model. First, the parameters are fitted using maximum likelihood, and then the models are selected using the Akaike Information Criterion (AIC) [33] and Akaike weight [34]. In short, AIC evaluates the quality of the fit that penalizes for overfitting too many parameters, while the Akaike weight gives the relative support of the different models, *i.e.* the probability for each model being the best one.

Finally, the distribution of parameters was assessed using the Metropolis-Hastings algorithm, which is a Markov chain Monte Carlo method for obtaining a sequence of random samples from a probability distribution [35,36]. The adaptive proposal distribution [37] as implemented in R package 'HelpersMG' [38] ensures that the acceptance rate is close to 0.234, which is the optimal acceptance rate [39]. A total of 20,000 iterations was run. Priors were all uniform with a range of proposals large enough to ensure that it does not constrain the limits of the parameters. From the 20,000 sets of parameters, we calculated the posterior predictive mean and standard deviation of the statistics of interest.

The adjustments were performed using the R package 'Phenology' available in the Comprehensive R Archive Network (https://cran.r-project.org) that implements these models [40]. Comparisons between the observed and modeled values were based on the adjusted coefficient of determination [41].

3 Results

Over the 6 nesting seasons covered by this study (2017-2018 to 2022-2023), 51 monitoring field trips were conducted, for a total effort of 98 days and over 300 hours of beach survey. This protocol led to the census of 2186 tracks, including 1105 nests based on the presence of body pits over the 28 prospected islets.

3.1 Nest detection probability

The loggerhead turtle nest tracks persistence estimated on the 'GLS' rookery was based on the survey of n=286 nests over the 2019-20 to 2022-23 period. Empirical data are presented in Fig 3(A). The confidence interval of predictions for each season overlaps nearly fully (not shown) and it confirms that season effect is not present in this dataset (Wald t-test for Season effect, Fisher combined p-value=0.11). Autocorrelation structure is very high with Phi=0.47. It can be seen in Fig 3(A) that very few false positive or negative detections are present in the dataset (a false positive or negative occurs when a non-detected track was seen again in the next field session). The fitted detection probability is shown in Fig 3(B) including its confidence interval based on 10^4 bootstrapping estimates. The fitted parameters of the logit model are: Intercept=1.82, Age=-0.08 which can be converted into *S* and *A* of the detection probability d_a:

$$d_a = 1/\left(1 + exp((-1/(4.S))(A - a)\right) \tag{5}$$

With *S*=-(1/(4*Age))=3.04 and *A*=4*S*Intercept=22.23. The half-time life of a body pit on these beaches is 22.23 days (95% confidence interval (CI) from 1 to 71 days, 50% CI from 5 to 29).

Fig 3. Evolution of nest detection over time. (A) Empirical data of detection of tracks according to their age for 3 seasons. (B) Model of probability of detection based on logistic function.

3.2 Nesting phenology of the loggerhead turtle in the 'Grand Lagon Sud' rookery

We fitted the 'GLS' 2017-2018 to 2022-2023 time series with Peak, LengthB, LengthE, Pmin and Max nesting phenology parameters using the R package phenology [40]. Flat parameter was not retained as it was fitted always close to 0. A Poisson distribution was preferred to a Negative Binomial since the mean number of nests per night is very low [30] and if NB was used, *k* fitted value was very large (>1000). We also tested different combinations of parameters to fit the more accurate phenological model and implement the possible seasonal for peak (P) and/or for LengthB and Length E (LBLE), based on AICc [33]. The selected model has a different seasonal peak date but a similar nesting season length for all 6-time series (Table 2).

Table 2. Model selection for the phenology of the number of nesting activities in the 'GLS' rookery during the 2017-2018 to 2022-2023 period.

Peak & LBLE model	AICc	ΔAICc	Akaike weight
Peak common + LBLE common	1640.46	5.95	0.05
**Peak seasonal + LBLE common**	**1634.51**	**0.00**	**0.95**
Peak common + LBLE seasonal	1646.28	11.77	0.00
Peak seasonal + LBLE seasonal	1649.32	14.80	0.00

If the Peak (*P*) and LengthB/LengthE (LBLE) are indicated as 'common', then this indicates that a single set of values is used for the 'GLS' rookery, otherwise a different set of values is used for each season, indicated as 'seasonal'. The selected model is indicated in bold. AICc: Akaike Information Criterion corrected for small sample sizes. ΔAICc is the difference in AICc from the best-performing model.

The fitted dates for the nesting seasonality in the 'GLS' rookery over the study period are shown in Table 3. The season starts in late October, and stretches for over 4 months into the austral summer. The peak of the activity occurs in late December/early January.

Table 3. Fitted nesting phenological parameters in the 'GLS' rookery and median seasonal number of nests with the 95% Credible Interval (CI).

Nesting season	2017-18	2018-19	2019-20	2020-21	2021-22	2022-23
**Phenology parameters**						
Beginning	02 Nov.	04 Nov.	31 Oct.	30 Oct.	27 Oct.	17 Oct.
Peak	30 Dec.	01 Jan.	28 Dec.	27 Dec.	24 Dec.	14 Dec.
End	27 Mar.	29 Mar.	24 Mar.	24 Mar.	21 Mar.	11 Mar.
Length (days)	145	145	145	145	145	145
**Seasonal number of nests**						
Lower 95% CI	377	402	312	232	347	299
median	532	531	455	303	437	362
Upper 95% CI	715	709	661	405	553	450

Fit for the selected nesting phenology model: date of the Beginning, Peak, End and Length of the loggerhead turtle nesting season.

3.3 Assessment of the overall seasonal number of loggerhead turtle nests on the 'GLS' rookery

For each prospected islets, time series of nest counts has been fitted using the phenological parameters *LengthB*, *LengthE* and *Peak* of the corresponding nesting season as fixed parameters, with only the *Max* parameter being specific to each islet time series [13,30]. We obtained the posterior predictive mean number of nests during the season, then we estimated the number of nesting activities for the islets not monitored during a season. Ultimately, an overall estimate of the nesting intensity at the rookery-scale was generated, as well as its

distribution based on 50,000 iterations using a Markov chain built with Monte Carlo sampling (Table 3). The overall seasonal number of nests in the 'GLS' rookery ranged from 303 (95% CI=232-405) during the 2020-2021 season to 532 (95% CI=377-715) during the 2017-2018 season, with a mean seasonal intensity of 437 nests (95% CI=328-582) over the 2017-2018 to 2022-2023 period.

4 Discussion

We have estimated the overall nesting activity of loggerhead turtles in the remote, scattered and hard-to-reach 'GLS' rookery for the first time. Based on an adapted monitoring protocol, we were able to highlight an important nesting intensity, ranging annually from 232 to 402 nests for the lowest limit of the 95% CI (Table 3). This result is quite exceptional considering both the precarious conservation status of the South Pacific subpopulation and available territorial knowledge prior to this study [17,20]. The mean number of nests 437 (95% CI=328-582) surpasses that of the estimated number of loggerhead turtle nesting activities in New Caledonia.

The assessment of nest detection dynamics over time on the islets of the 'GLS' rookery has allowed us to feed the models more accurately. The resulting nesting phenology parameters estimated are consistent with local reports in New Caledonia, with a season duration of 4.8 months, starting in late October/early November and with a peak date occurring in late December/early January [20].

Monitoring protocol replication potential and conservation implications.

The management of marine turtle species, with ocean-wide distributions and philopatric behaviors, can be facilitated by better understanding their reproductive population trends [9]. The lack of standardized data series in New Caledonia has been highlighted by the IUCN in 2015 as a source of uncertainty regarding the assessment of the South Pacific subpopulation [17]. A strong emphasis has thus been given to the need to develop robust estimates of nesting

intensity on high potential sites, such as the 'Grand Lagon Sud' area. Comprehensive monitoring on this important zone to the loggerhead turtle has been hindered by the financial and technical difficulties to adequately survey the numerous and distant coral islets inventoried in the area. Therefore, we have compiled a 6-year time-series to adapt a monitoring and analytic protocol in order to estimate the overall number of nesting activities. Resulting knowledge has allowed us to identify the 'GLS' rookery as the first nesting area in terms of number of nests for the Critically Endangered loggerhead turtle South Pacific subpopulation in New Caledonia. This study also establishes the preliminary basis in the perspective of assessing the demographic trends, which requires several years, if not decades, of survey effort due to the marine turtle life history traits [42]. The only conclusion that can be drawn at this stage is that the population is not facing an imminent crash or extremely rapid growth.

The late identification of this major area to the reproduction of loggerhead turtles in New Caledonia is very likely to be attributed to the lack of efficient tools to assess the nesting intensity in this geographical context. Considering that other important nesting aggregations had been identified in New Caledonia, especially in the north-western lagoon where both the number of nesting islets and the monitoring constraints are very similar to that found in the 'GLS' area, it is reasonable to assume that other nesting rookeries are yet to be confirmed at the territorial scale. The assessment of such areas could prove useful to highlight innovative conservation actions to undertake for the more efficient preservation of this emblematic species.

5 Conclusions

In the light of this study and available information on the South Pacific loggerhead turtle subpopulation [17,18,20], we have come to re-evaluate the estimated number of nesting females in the breeding population from a range of 1820-6370 to 2450-6950 individuals. The importance of New Caledonia to this nesting population has also been refined, with a mean estimation of 20% (14-39%) of all reproductive females breeding and nesting within the

territory. An important knowledge gap is still to be filled considering other high-potential insular rookeries in the South Pacific region, such as the north-western lagoon of New Caledonia that could prove to be as important as the ‘GLS’ rookery. The replication of this monitoring protocol could substantially improve both regional knowledge and management policies of this marine turtle species.

Acknowledgements

We would like to thank the South Province of New Caledonia for their substantial contribution to the study, by providing precious technical and fieldwork support and administrative authorizations to seasonally visit restricted-access sites in the ‘Grand Lagon Sud’ area. We extend our gratitude to all WWF-France volunteers, the French National Research Institute for Sustainable Development (IRD) and the Coastal Observatory of Geological Services of New Caledonia (OBLIC) members who contributed to field missions. Finally, we would like to thank all the local tribes in which geographies this study has taken place. This work has been supported by the South Province of New Caledonia (permit #3245-2020/ARR/DDDT).

References

1. Ceriani SA, Casale P, Brost M, Leone EH, Witherington BE. Conservation implications of sea turtle nesting trends: elusive recovery of a globally important loggerhead population. Ecosphere. 2019;10(11):19 p.

2. Reynolds JH, Thompson WL, Russell B. Planning for success: Identifying effective and efficient survey designs for monitoring. Biol Conserv. 2011;144(5):1278–1284.

3. Robinson NM, Scheele BC, Legge S, Southwell DM, Carter O, Lintermans M, et al. How to ensure threatened species monitoring leads to threatened species conservation. Ecol Manag Restor. 2018;19(3):222–229.

4. Alves F, Rosso M, Li S, Nowacek DP. A sea of possibilities for marine megafauna.

Science. 2022;375(6579):391–393.

5. Grémillet D, Chevallier D, Guinet C. Big data approaches to the spatial ecology and conservation of marine megafauna. ICES J Mar Sci. 2022;79(4):975–986.

6. Herbert-Read JE, Thornton A, Amon DJ, Birchenough SNR, Côté IM, Dias MP, et al. A global horizon scan of issues impacting marine and coastal biodiversity conservation. Nat Ecol Evol. 2022;6(9):1262–1270.

7. Wallace BP, DiMatteo AD, Bolten AB, Chaloupka MY, Hutchinson BJ, Abreu-Grobois FA, et al. Global conservation priorities for Marine turtles. PLoS One. 2011;6(9):14 p.

8. Casale P, Ceriani SA. Sea turtle populations are overestimated worldwide from remigration intervals: correction for bias. Endanger Species Res. 2020;41:141–151.

9. Metcalfe K, Agamboué PD, Augowet E, Boussamba F, Cardiec F, Fay JM, et al. Going the extra mile: Ground-based monitoring of olive ridley turtles reveals Gabon hosts the largest rookery in the Atlantic. Biol Conserv. 2015;190:14–22.

10. Omeyer LCM, McKinley TJ, Bréheret N, Bal G, Petchell Balchin G, Bitsindou A, et al. Missing Data in Sea Turtle Population Monitoring: A Bayesian Statistical Framework Accounting for Incomplete Sampling. Front Mar Sci. 2022;9(817014):15 p.

11. SWOT Scientific Advisory Board. The State of the World's Sea Turtles (SWOT) Minimim Data Standards for Nesting Beach Monitoring. Technical Report. 2011:24 p.

12. Delcroix E, Bédel S, Santelli G, Girondot M. Monitoring design for quantification of marine turtle nesting with limited effort: A test case in the Guadeloupe archipelago. Oryx. 2014;48(1):95–105.

13. Fretey J, Read TC, Carron L, Fontfreyde C, Fourdrain A, Kerandel JA, et al. From terra incognita to hotspot: The largest South Pacific green turtle nesting population in the forgotten reefs of New Caledonia. Oryx. 2023;57(5):626–636.

14. Shamblin BM, Hart KM, Martin KJ, Ceriani SA, Bagley DA, Mansfield KL, et al. Green

turtle mitochondrial microsatellites indicate finer-scale natal homing to isolated islands than to continental nesting sites. Mar Ecol Prog Ser. 2020;643:159–171.

15. Barnett J, Campbell J. Climate Change and Small Island States. Power, Knowledge and the South Pacific. 1st ed. London: Earthscan Routledge; 2010. 232 p.

16. Nunn PD, Mimura N. Vulnerability of Tonga to future sea-level rise. J Coast Res. 1997;(24):133–151.

17. Limpus CJ, Casale P. The IUCN Red List of Threatened Species: *Caretta caretta* (South Pacific subpopulation), Loggerhead Turtle. IUCN Red List Threat Species. 2015;8235:14 p.

18. Limpus CJ, Limpus DJ. Loggerhead turtles in the equatorial and southern Pacific Ocean: a species in decline. In: Bolten AB, Witherington BE, editors. Loggerhead Sea Turtles. Washington DC: Smithsonian Books; 2003. 199–209.

19. Read TC, Fitzsimmons NN, Wantiez L, Jensen MP, Keller F, Chateau O, et al. Mixed stock analysis of a resident green turtle, *Chelonia mydas*, population in New Caledonia links rookeries in the South Pacific. Wildl Res. 2015;42(6):488–499.

20. Barbier M, Lafage D, Bourgogne H, Read T, Attard M, Fournière K, et al. Assessment of the nesting population demography of loggerhead turtles (*Caretta caretta*) in La Roche Percée: First long-term monitoring in New Caledonia. Aquat Conserv Mar Freshw Ecosyst. 2023;33(6):579–591.

21. Limpus CJ, Boyle M, Sunderland T. New Caledonian Loggerhead Turtle Population Assessment : 2005 Pilot Study. Queensl Turt Res. 2005;(04):77–92.

22. Andréfouët S, Cabioch G, Flamand B, Pelletier B. A reappraisal of the diversity of geomorphological and genetic processes of New Caledonian coral reefs: a synthesis from optical remote sensing, coring and acoustic multibeam observations. Coral Reefs. 2009;28(3):691–707.

23. Pilcher NJ. Review of the Status of Sea Turtles in the Pacific Ocean. Secretariat of the Pacific Regional Environment Programme. Apia, Samoa; 2021. 139 p.

24. Pritchard PCH, Mortimer JA. Taxonomy, External Morphology, and Species Identification. Res Manag Tech Conserv Sea Turtles. 1999;4(4):18 p.

25. Robinson NJ, Sanfèlix MM, Blanco GS, Clyde-Brockway C, Hill JE, Paladino FV, et al. Effect of water temperature on the duration of the internesting interval across sea turtle species. J Therm Biol. 2022;110(103342):5 p.

26. Jackson AL, Broderick AC, Fuller WJ, Glen F, Ruxton GD, Godley BJ. Sampling design and its effect on population monitoring: How much monitoring do turtles really need? Biol Conserv. 2008;141(12):2932–2941.

27. Royle JA, Dorazio RM. Hierarchical modeling and inference in ecology: the analysis of data from populations, metapopulations and communities. San Diego: Academic Press, Elsevier; 2008. 444 p.

28. Venables WN, Ripley BD. Modern Applied Statistics with S-PLUS. Fourth edi. New York: Springer Science & Business Media; 2002. 495 p.

29. Girondot M. Estimating density of animals during migratory waves: a new model applied to marine turtles at nesting sites. Endanger Species Res. 2010;12(2):95–105.

30. Girondot M. Optimizing sampling design to infer the number of marine turtles nesting on low and high density sea turtle rookeries using convolution of negative binomial distribution. Ecol Indic. 2017;81:83–89.

31. Taylor LR, Woiwod IP, Perry JN. The Negative Binomial as a Dynamic Ecological Model for Aggregation, and the Density Dependence of k. J Anim Ecol. 1979;48(1):289–304.

32. Girondot M, Barry J. Computation of the Distribution of the Sum of Independent Negative Binomial Random Variables. Math Comput Appl. 2023;28(63):9 p.

33. Akaike H. A new look at the statistical model identification. IEEE Trans Automat Contr.

1974;19(6):716–723.

34. Burnham KP, Anderson DR. Practical use of the Information-Theoretic Approach. In: Model selection and inference. New York: Springer-Verlag; 1998. p. 75–117.

35. Hastings WK. Monte Carlo sampling methods using Markov chains and their applications. Biometrika. 1970;57(1):97–109.

36. Metropolis N, Rosenbluth AW, Rosenbluth MN, Teller AH, Teller E. Equation of state Calculations by Fast Computing Machines. J Chem Phys. 1953;21(6):1087–1092.

37. Rosenthal JS. Optimal proposal distributions and adaptive MCMC. In: Handbook of Markov Chain Monte Carlo. Boca Raton: Chapman and Hall/CRC Press; 2011. p. 93–112.

38. Girondot M. HelpersMG: Tools for Environmental Analyses, Ecotoxicology and Various R Functions. Indianapolis, USA: The Comprehensive R Archive Network; 2023.

39. Roberts GO, Rosenthal JS. Optimal Scaling for Various Metropolis-Hastings Algorithms. Stat Sci. 2001;16(4):351–367.

40. Girondot M. Phenology: Tools to manage a parametric function that describes phenology. Indianapolis, USA: The Comprehensive R Archive Network; 2023.

41. Nagelkerke NJD. A note on a general definition of the coefficient of determination. Biometrika. 1991;78(3):691–692.

42. Chaloupka M, Bjorndal KA, Balazs GH, Bolten AB, Ehrhart LM, Limpus CJ, et al. Encouraging outlook for recovery of a once severely exploited marine megaherbivore. Glob Ecol Biogeogr. 2008;17(2):297–304.

Fig1

Click here to access/download;Figure;Fig1.tif

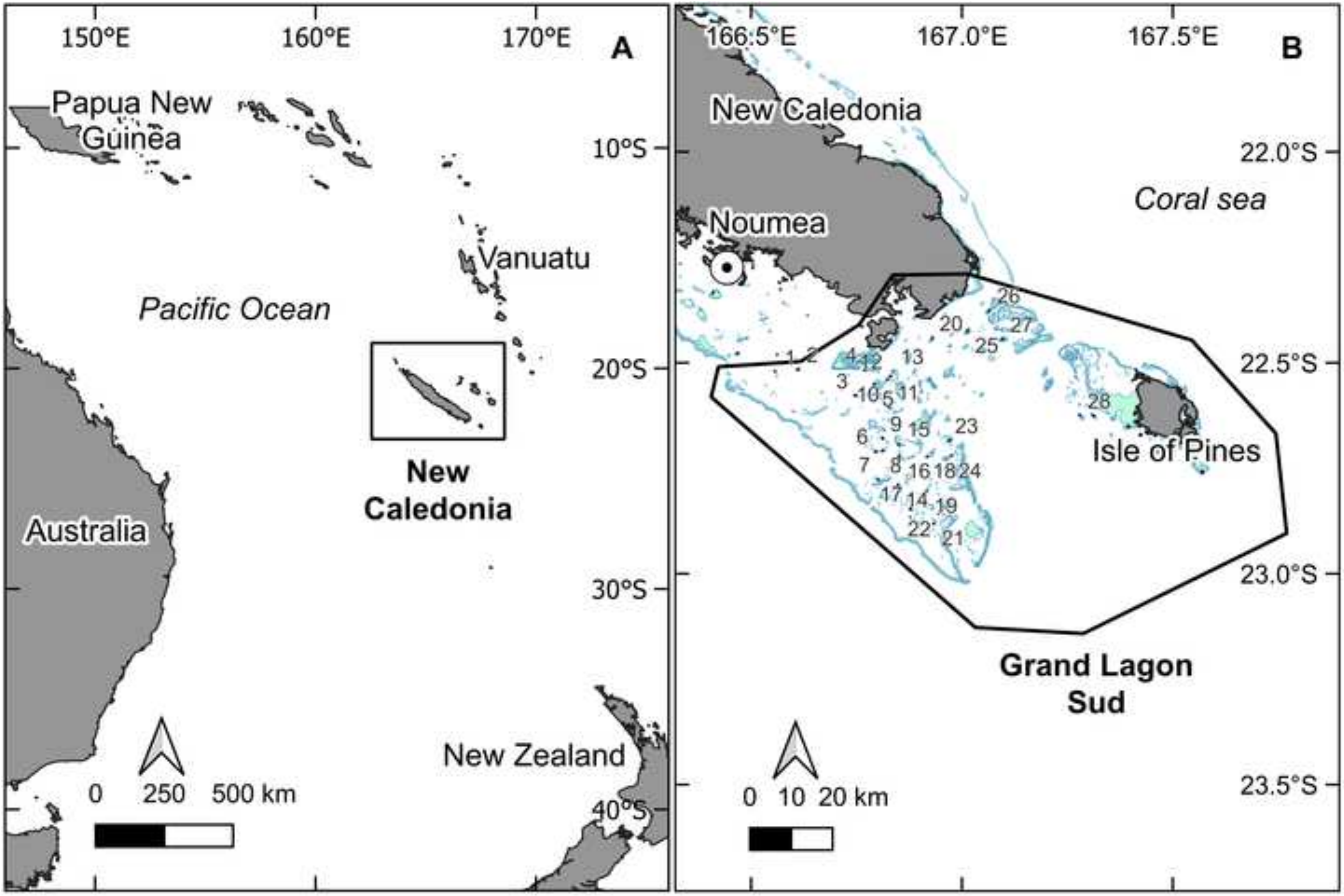

Click here to access/download;Figure;Fig2.tif

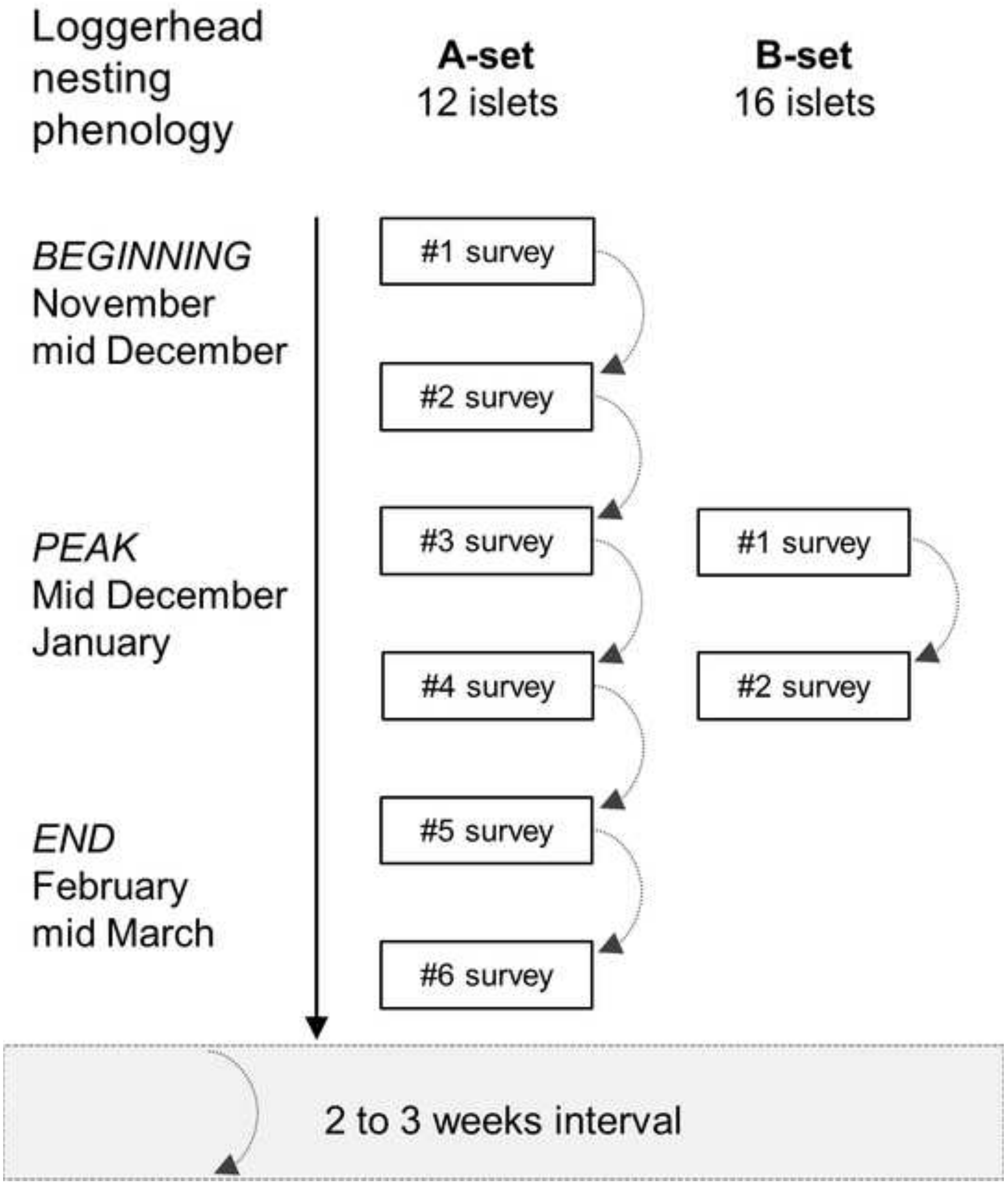

Fig3

Click here to access/download;Figure;Fig3.tif

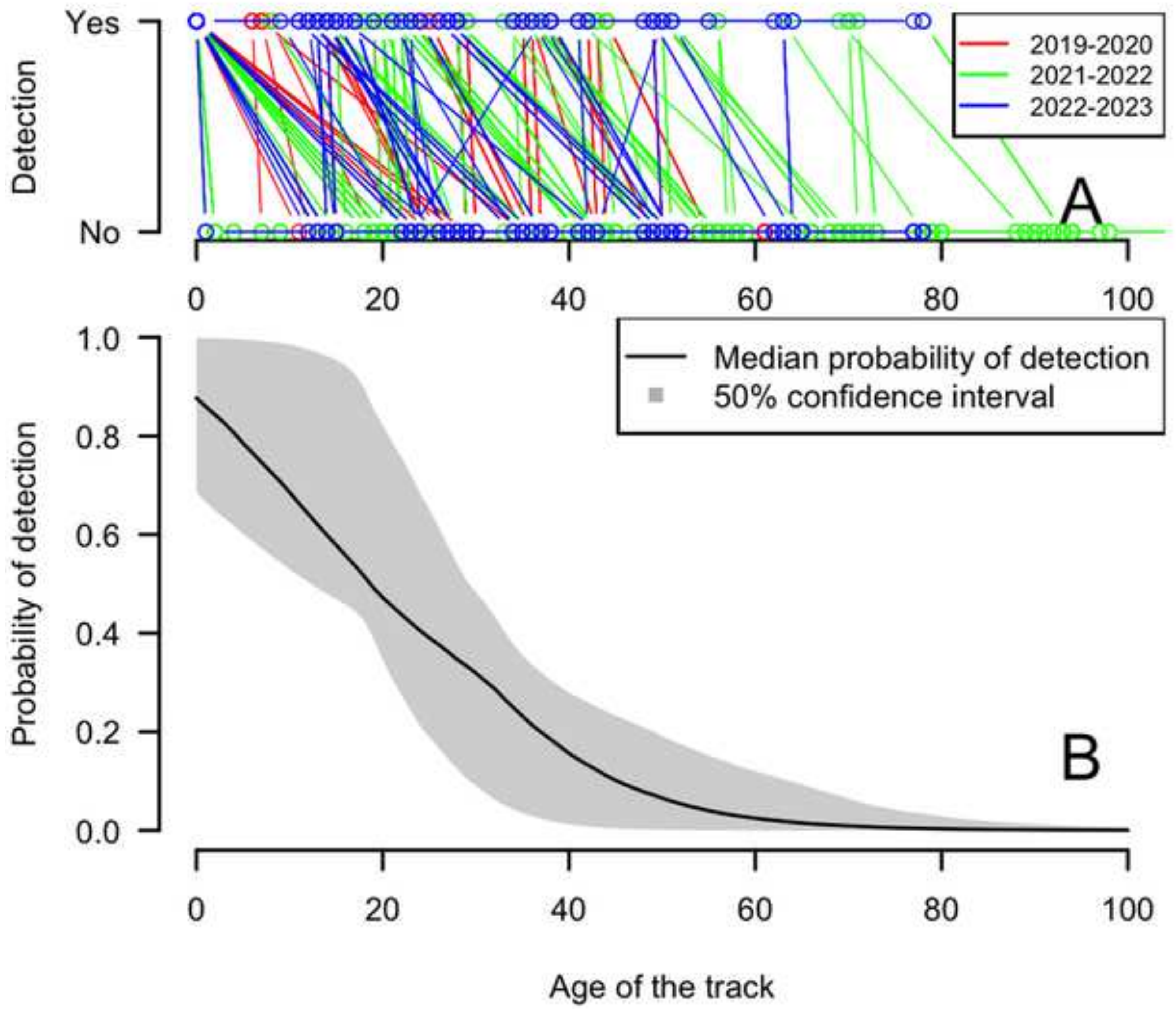

DATABASE_1

Click here to access/download
Supporting Information
DB_nestcounts_mission_20240129.csv

DATABASE_2

Click here to access/download
Supporting Information
DB_nestcounts_total_20240129.csv

DATABASE_3

Click here to access/download
Supporting Information
DB_nestdetectability_total_20240129.csv
